# Supplementary material for: Markovnikov hydroamination of terminal alkenes by phosphine redox catalysis
Source: Nature. 2026 Feb 23;652(8108):96–104. doi: 10.1038/s41586-026-10263-7 (PMC13043287; doi:10.1038/s41586-026-10263-7)
Supplement: Supplementary file 1 — This file contains the following sections: (1) general information; (2) control experiments and reaction optimization; (3) functional group robustness screen; (4) general procedure B and characterization; (5) other procedures and characterization; (6) X-ray crystal structure; (7) evaluation of by-products; (8) synthesis of alkenes; (9) mechanistic investigations; (10) DFT studies; (11) references; and (12) NMR characterization. [file 41586_2026_10263_MOESM1_ESM.pdf]

nature portfolio

https://doi.org/10.1038/s41586-026-10263-7

Supplementary information

Markovnikov hydroamination of terminal alkenes by phosphine redox catalysis

In the format provided by the authors and unedited

Markovnikov hydroamination of terminal alkenes by phosphine redox catalysis

Flora Fan[1], Kassandra F. Sedillo[2], Alexander J. Maertens[1], Abigail G. Doyle[1]*

[1]Department of Chemistry and Biochemistry, University of California, Los Angeles; California 90095, United States.

[2]Department of Chemistry, Princeton University; Princeton, New Jersey 08540, United States.

*Correspondence to: abigaildoyle@g.ucla.edu

Supporting Information

Table of Contents

1. General Information 3
2. Control Experiments and Reaction Optimization 4
3. Functional Group Robustness Screen 17
4. General Procedure B and Characterization 18
5. Other Procedures and Characterization 40
6. X-ray Crystal Structure 41
7. Evaluation of Byproducts 43
8. Synthesis of Alkenes 46
9. Mechanistic Investigations 48
   Stern-Volmer Quenching Experiments 48
   Light-on Light-off Cycling 53
   Quantum Yield Measurement 54
   Phosphorus Speciation Experiments 59
   Radical Cyclization Experiments 65
   Deuteration Study 72
   Competition Experiments for Hammett Study 77
   Kinetic Isotope Effect Experiments 79
   Competitive Intramolecular Reactivity 82
   Cyclic Voltammetry 83
   UV-Vis Study 84
   Internal Alkenes as Substrates 85
   Unsuccessful Substrates 86
10. DFT Studies 88
General Considerations 88
NCR Addition to 1-Hexene 88
C–N Bond Formation via Nucleophilic Amination 90
N-site Selectivity 92
Comparison of C–N Bond Formation between Phosphines 94
Comparison of Azole vs. Alkene Nucleophilic Addition 96
Alternative Product Formation Pathways 99
Reactivity from P–N Phosphoranyl Radical 99
Deprotonation via Ternary Complex 99
Selectivity in Indole Functionalization 100
11. References 101
12. NMR Characterization 104

1. General Information

Materials: Commercial reagents were acquired from Sigma-Aldrich, Ambeed, Strem, TCI, Combi-Blocks, or Oakwood and used as received, unless otherwise noted. Solvents were sparged with N_2 and brought into a N_2-filled glovebox prior to use. α,α,α-trifluorotoluene ($PhCF_3$) was purchased directly from Sigma Aldrich in a Sure Seal bottle and was used without further purification. All phosphines were used without further purification.

Methods: Organic solutions were concentrated under reduced pressure using a rotary evaporator unless otherwise noted. Automated column chromatography was performed using silica gel cartridges on a Biotage Selekt. Preparative column chromatography was performed using SiliCycle glass-backed 1000 μm silica gel plates.

Instrumentation: Proton and carbon nuclear magnetic resonance (^{1}H NMR) spectra were recorded on a Bruker 500 spectrometer (500 MHz). Chemical shifts for protons are reported in parts per million (ppm) and are referenced to residual protium in the NMR solvent ($CDCl_3$ = δ 7.26 ppm). Chemical shifts for carbon are reported in parts per million and are referenced to the carbon resonances of the solvent residual peak ($CDCl_3$ = δ 77.16 ppm). NMR data are represented as follows: chemical shift (δ ppm), multiplicity (s = singlet, bs = broad singlet, d = doublet, t = triplet, q = quartet, p = pentet, sx = sextet, m = multiplet), coupling constant in Hertz (Hz), integration. High-resolution mass spectra (HRMS) were obtained on a Thermo Exactive Plus MSD (DART-MS) equipped with an ID-CUBE ion source and a Vapur Interface (Ion Sense Inc.) (atmospheric-pressure chemical ionization, APCI). Cyclic voltammetry (CV) experiments were obtained with a Gamry Interface 1010 Potentiostat/Galvanostat/ZRA instrument and processed using Gamry Echem FrameworkTM and AnalystTM software. Ultraviolet-Visible spectroscopy (UV-Vis) was performed with a Shimadzu UV-3101PC spectrophotometer. Fluorescence emission spectra were obtained with a Photon Technologies International QuantaMaster Spectrofluorimeter. Fourier transform infrared (FT-IR) spectra were recorded on an Agilent Cary 630 FTIR spectrometer and are reported in terms of frequency of absorption (cm^{-1}).

Light Sources: Reactions were optimized in 1- and 2-dram vials using either a Penn PhD photoreactor M2 (blue LEDs, 450 nm) purchased from Sigma-Aldrich with the following settings: 100% intensity, 2800 rpm fan speed, 1500 rpm stir rate, or Kessil PR160L 427 nm lamps purchased from Kessil. Kessil lamps were placed 2 cm away from 20 mL reaction vials without the use of fans and set to 50% intensity. Reactions are reported to occur at room temperature (rt) since no additional heat source was employed, but the internal temperature of solutions in reaction vials in the photoreactor could rise to 40°C as measured by a connected temperature probe. The internal temperature of reactions in the Kessil set up could rise to 65°C as measured by infrared thermometer. The reaction vials were placed on a stir plate with a stir rate of 800 rpm, and an orange plexiglass shield was placed in front of the setup for reaction viewing.

2. Control Experiments and Reaction Optimization

General Procedure A for Reaction Optimization (0.10 mmol scale): A 1-dram glass vial equipped with a stir bar was charged with the N–H heteroaromatic substrate. The vial was loosely covered with a cap and brought into a N_2-filled glovebox. Phosphine (0.020 mmol, 0.2 equiv.) and photocatalyst (0.002 mmol, 0.020 equiv.) were then added to the vial, followed by 1.0 mL of α,α,α-trifluorotoluene (0.1 M), resulting in a suspension. The reaction vial was charged with 2,4,6-*tri*isopropylbenzenethiol (TRIP-SH) (2.3 μL, 0.010 mmol, 0.10 equiv.) and alkene (0.30 mmol, 3.0 equiv.). The vial was capped, sealed with electrical tape, and removed from the glovebox. The reaction was stirred at 1500 rpm for 18 hours while irradiating within the photoreactor (450 nm, 100% intensity, 2800 rpm fan speed). At the end of 18 hours, the vial was removed from the photoreactor (PR). 1,3,5-Trimethoxybenzene solid (~1 equiv.) was added to the vial as an internal standard for determining the yield by ^1H NMR; the exact amount of standard added was recorded to back-calculate the corresponding peak integration.

Control Experiments

All yields in Tables S1 to S28 were determined by ^1H NMR with 1,3,5-trimethoxybenzene as an internal standard unless noted otherwise.

Table S1. Deoptimization experiments.

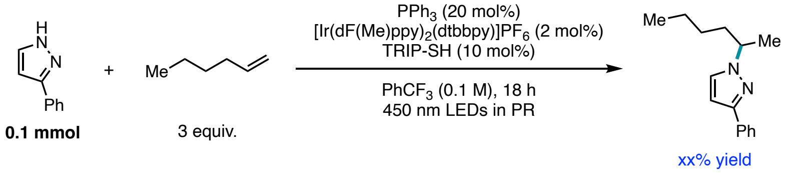

Modification	Yield
N/A	89%
No TRIP-SH	7%
No PPh_3	0%
No photocatalyst	0%
No light	0%
2,6-lutidine instead of TRIP-SH	7%
K_3PO_4 instead of TRIP-SH	5%
Setup in air, N_2 sparging for 5 min	55%
Setup in air, N_2 sparging for 5 min, P(*p*-OMePh)$_3$ as phosphine catalyst	76%

Table S2. Metal additive screen.

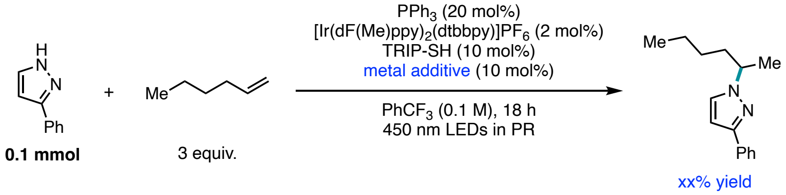

Additive	Yield
N/A	89%
$Pd(OAc)_2$	0%
$Fe(OTf)_3$	0%
Co(salen)	0%
$Cu(OAc)_2$	0%
$NiBr_2$	0%
$Fe(acac)_3$	0%

Reaction Optimization

Table S3. Phosphine catalyst screen.

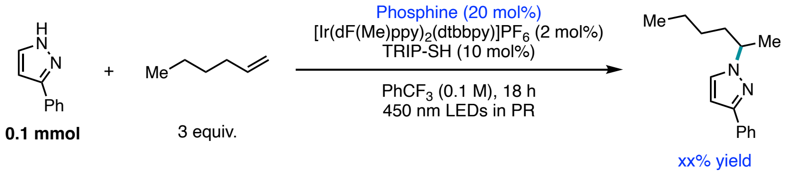

Phosphine	Yield
PPh_3	89%
PCy_3 (10 mol%)	0%
PPh_2Cy	4%
$PPhCy_2$	0%
PPh_2Me	0%
$PPhMe_2$	0%
P(*p*-OMe-Ph)$_3$	93%
P(*p*-tolyl)$_3$	100%
P(*p*-F-Ph)$_3$	0%
P(*p*-Cl-Ph)$_3$	0%
P(*p*-CF_3-Ph)$_3$	0%
P(2,4,6-Mes-Ph)$_3$	0%
P(*m*-OMe-Ph)$_3$	80%
P(*o*-OMe-Ph)$_3$	0%
rac-BINAP	0%
(*R*)-MOP	0%

Table S4. Phosphine loading screen.

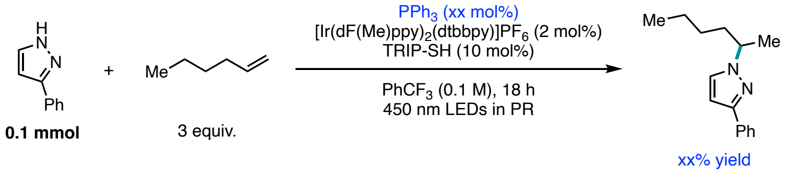

Phosphine loading (mol%)	Yield
10	9%
20	89%
100	100%
200	72%

Table S5. Photocatalyst screen.

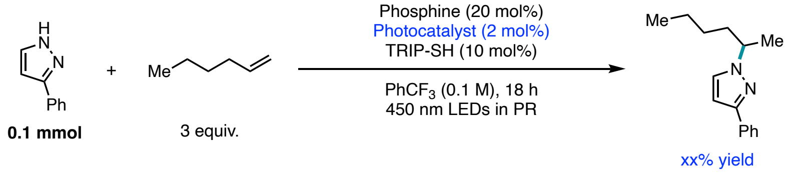

with PPh_3			
**Photocatalyst**	**PC*/PC•− (V)**	**$E_{1/2}$(M*/M−)(V)**	**Yield**
[Ir(dF(Me)ppy)$_2$(dtbbpy)]PF$_6$	-	0.97	89%
[Ir(dF(CF$_3$)ppy)$_2$(dtbbpy)]PF$_6$	-	1.21	18%
4DPAIPN	0.99	-	0%
3DPA2FBN	1.00	-	0%
3DPAFIPN	1.11	-	0%
4DPATPN	1.19	-	0%
4CzPN	1.30	-	0%

with P(*p*-OMePh)$_3$*			
**Photocatalyst**	**PC*/PC•− (V)**	**$E_{1/2}$(M*/M−)(V)**	**Yield**
[Ir(dF(CF$_3$)ppy)$_2$(dtbbpy)]PF$_6$	-	1.21	43%
4DPAIPN	0.99	-	0%
3DPA2FBN	1.00	-	0%
3DPAFIPN	1.11	-	0%
4DPATPN	1.19	-	6%
4CzIPN	1.43	-	0%
Mes-Acr-Ph$^+$	2.10	-	0%

* PhH (0.1 M) as solvent

with P(*p*-OMePh)$_3$*			
**Photocatalyst (20 mol%)**	**PC*/PC•− (V)**	**Kessil Lamp at 100% LI (nm)**	**Yield**
9,10-dicyanoanthracene	1.14	427	4%
benzophenone	1.28	370	0%
9-fluorenone	0.96	370	0%
thioxanthene	1.18	370	0%
acridine orange hydrochloride	0.95	565	0%
rhodamine 6G	0.95	565	0%
methylene blue	1.14	630	0%

* PhH (0.1 M) as solvent

The screened organic photocatalysts possess reduction potentials to match that of the iridium-based photocatalyst currently employed, but these were found to be ineffective. We attribute the poor performance of these organic photocatalysts to a poorly matched ability to reduce the thiyl radical; these photocatalysts are also known to be more susceptible to back-electron transfer, which we found to be a major limitation in our prior work on anti-Markovnikov hydroamination with phosphoranyl radicals[1].

Table S6. Photocatalyst loading screen.

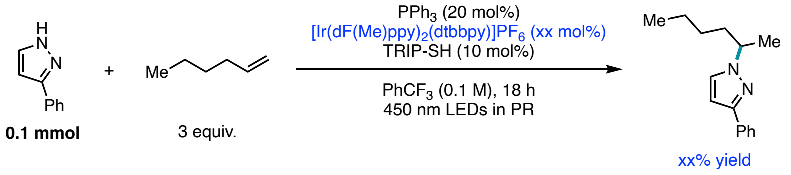

Photocatalyst loading (mol%)	Yield
0.1	46%
0.5	75%
1	70%
2	89%

Table S7. Alkene loading screen.

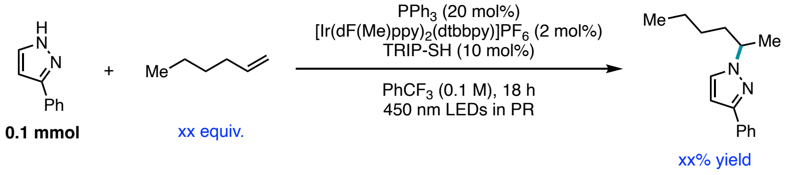

Alkene loading (equiv.)	Yield
1	64%
1.5	54%
2	74%
3	89%
1 equiv., 48 h	86%

Table S8. Thiol screen.

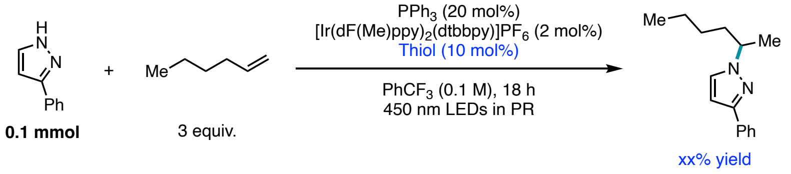

Thiol	Yield
TRIP-SH	89%
$TRIP_2S_2$	63%
2,4,6-trimethylbenzenethiol	44%

Table S9. Thiol loading screen.

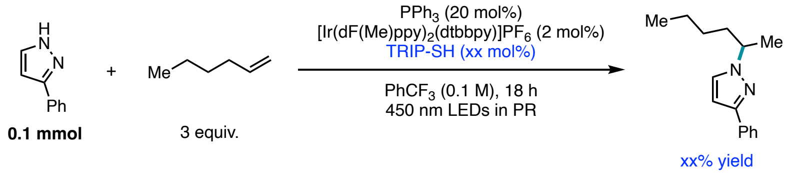

Thiol loading (mol%)	Yield
1	58%
2	93%
5	91%
10	89%
20	83%
50	44%
100	6%

Table S10. Solvent screen.

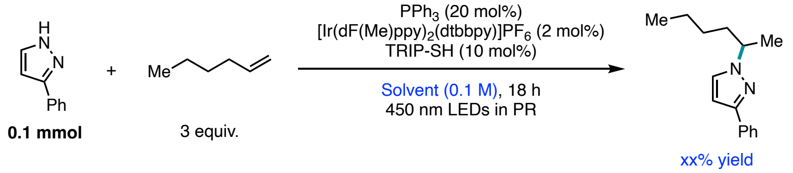

Solvent	Yield
$PhCF_3$	89%
PhMe	100%
PhH	100%
CPME	100%
MeCN	9%
MeCN, with 2 equiv. H_2O	9%*
1:1 MeCN:H_2O	4%*

*No Ritter amination product was observed.

Table S11. Concentration screen.

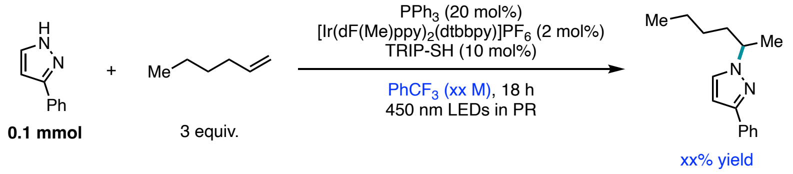

Concentration (M)	Yield
0.1	89%
0.2	68%

Table S12. Time course.

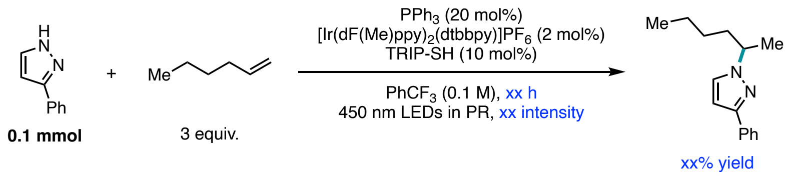

Time (h)	100% Intensity	75% Intensity	50% Intensity	25% Intensity
	Yield	Yield	Yield	Yield
0.5	0%	-	-	-
1	3%	-	-	-
2	11%	-	-	-
4	23%	16%	9%	4%
6	30%	-	-	-
8	47%	30%	19%	10%
10	52%	-	-	-
12	80%	54%	26%	13%
16	93%	54%	37%	33%

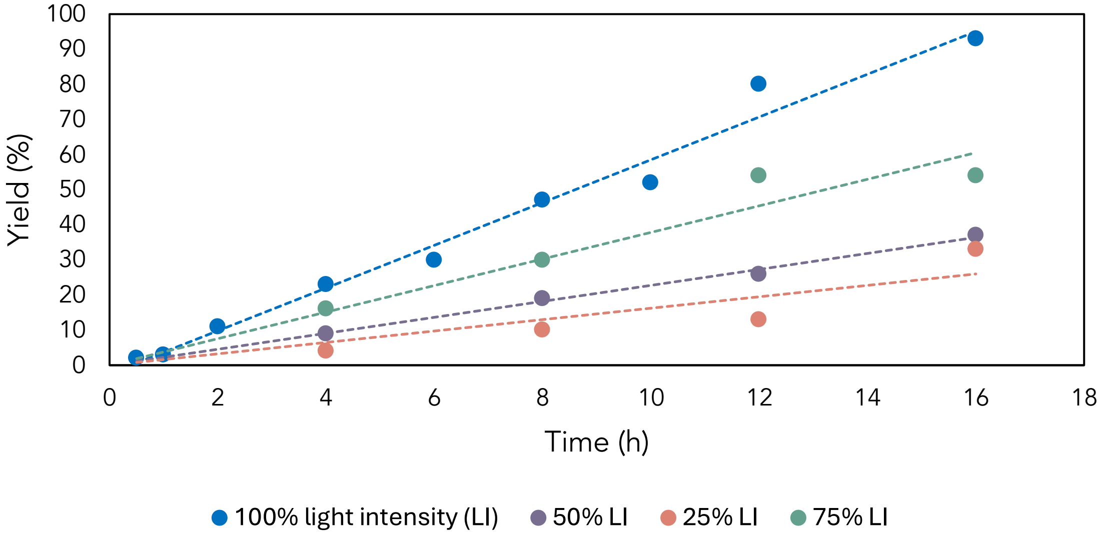

Fig. S1. Time course plot at varied light intensities; graphical representation of Table S12.

At all light intensities at which the reaction is run, the yield of the product increases linearly over time, indicating yield data could be a simpler readout for rate data. Since the slope increases at higher light intensities, the reaction rate is light limited.

Table S13. N–H substrate and phosphine screen.

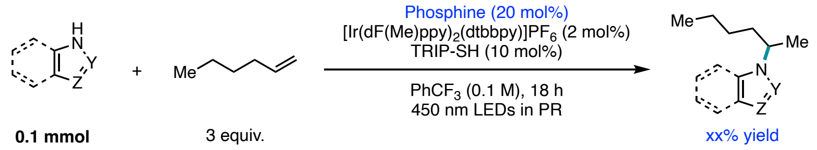

N–H substrate	Yield with PPh_3	Yield with P(*p*-OMePh)$_3$
3-Ph-pyrazole	89%	93%
Pyrazole	57%	63%
Indazole	49%	43%
4-Ph-imidazole	20%	92%
Benzimidazole	20%	77%
Triazole	96%	0%
Carbazole	0%	39%
Indole	0%	21%
Oxindole	0%	70%
Pyrrole	0%	0%
(*p*-*t*-Bu)aniline	0%	0%
Purine	0%	0%
Benzamide	0%	0%
Benzotriazole	0%	0%
Tetrazole	0%	0%

Optimization on other N–H substrates: Benzimidazole

Table S14. Thiol loading screen.

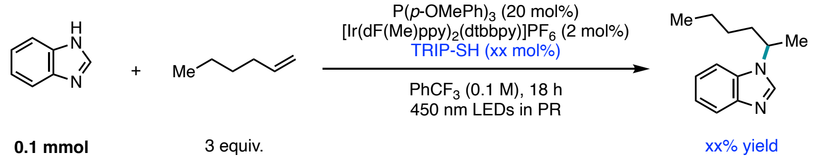

Thiol loading (mol%)	Yield
2	19%
5	36%
10	77%
20	66%
50	50%

Table S15. Phosphine loading screen.

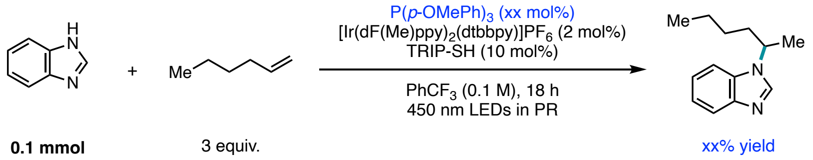

Phosphine loading (mol%)	Yield
5	0%
10	16%
20	77%
50	68%

Table S16. Cross-condition screen.

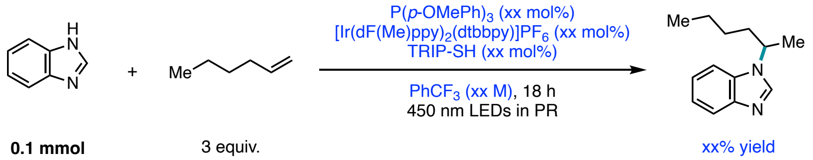

Solvent concentration (M)	Phosphine loading (mol%)	Photocatalyst loading (mol%)	Thiol loading (mol%)	Yield
0.02	35	0.05	50	58%
0.02	40	0.05	40	66%
0.02	40	0.05	50	58%
0.05	20	1	10	60%
0.05	40	0.05	50	64%
0.05	50	1	10	59%
0.05	50	1	20	58%
0.05	50	1	50	68%
0.05	50	2	50	64%
0.1	20	2	10	77%
0.1	25	2	10	65%
0.1	50	1	20	57%
0.1	50	1	50	67%
0.1	50	2	50	62%
0.4	50	1	20	56%
0.4	50	5	50	58%

Optimization on other N–H substrates: Indole

Table S17. Solvent screen.

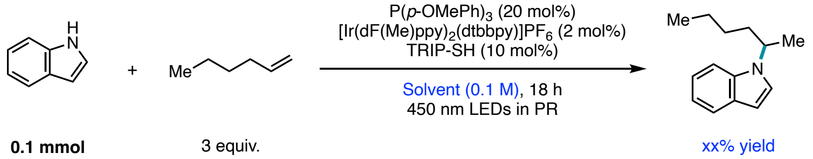

Solvent	Yield
$PhCF_3$	21%
PhMe	16%
CPME	0%
2-MeTHF	0%
(1,2-*di*-Cl)PhH	0%

Table S18. Thiol loading screen.

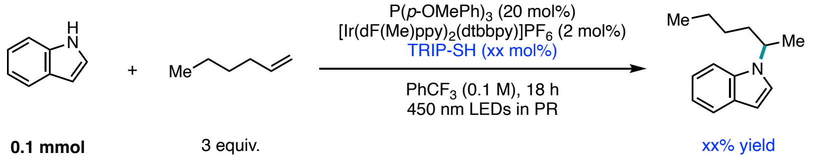

Thiol loading (mol%)	Yield
1	1%
5	6%
10	21%
20	30%
50	13%

Table S19. Phosphine loading screen.

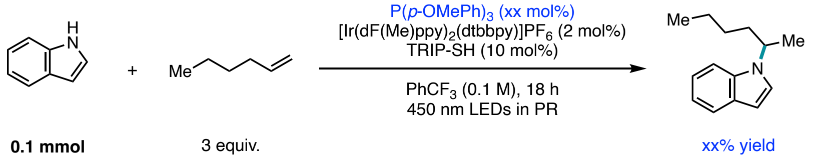

Phosphine loading (mol%)	Yield
5	4%
10	16%
20	21%
50	25%

Table S20. Cross-condition screen.

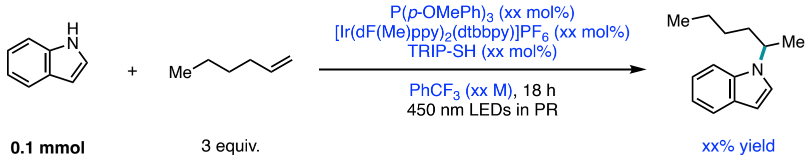

Solvent concentration (M)	Phosphine loading (mol%)	Photocatalyst loading (mol%)	Thiol loading (mol%)	Yield
0.05	20	1	10	8%
0.05	50	1	10	14%
0.05	50	1	50	4%
0.1	20	2	10	21%
0.1	50	1	50	22%
0.4	50	5	50	0%

Optimization on other N–H substrates: Carbazole

Table S21. Solvent screen.

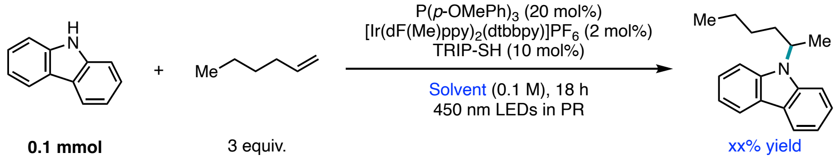

Solvent	Yield
$PhCF_3$	39%
PhMe	37%
CPME	36%

Table S22. Thiol loading screen.

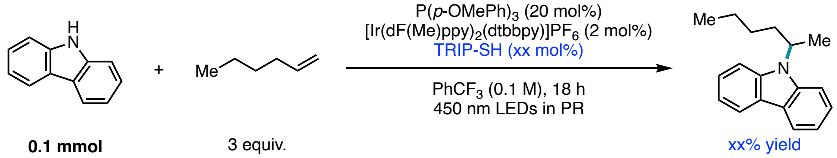

Thiol loading (mol%)	Yield
2	13%
5	11%
10	39%
20	15%
50	34%

Table S23. Phosphine screen.

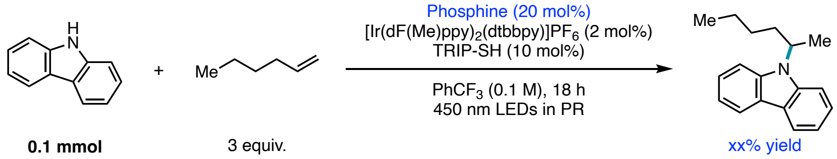

Phosphine	Yield
PPh_3	0%
(*p*-OMe-Ph)$_3$P	39%
(*p*-F-Ph)$_3$P	0%
(*p*-Cl-Ph)$_3$P	0%
(*p*-CF_3-Ph)$_3$P	0%

Table S24. Phosphine loading screen.

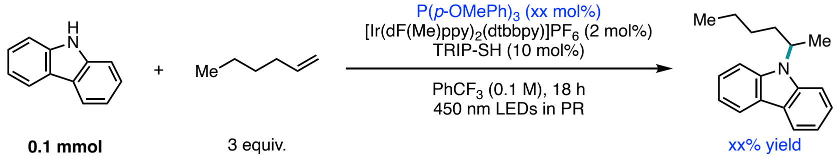

Phosphine loading (mol%)	Yield
5	0%
10	0%
20	39%
50	29%

Table S25. Cross-condition screen.

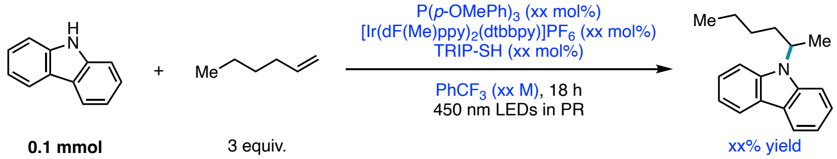

Solvent concentration (M)	Phosphine loading (mol%)	Photocatalyst loading (mol%)	Thiol loading (mol%)	Yield
0.1	20	2	10	39%
0.1	50	1	50	23%
0.05	50	1	50	11%
0.05	50	1	10	16%
0.05	20	1	10	14%

Optimization on other N–H substrates: Indazole

Table S26. Thiol loading screen.

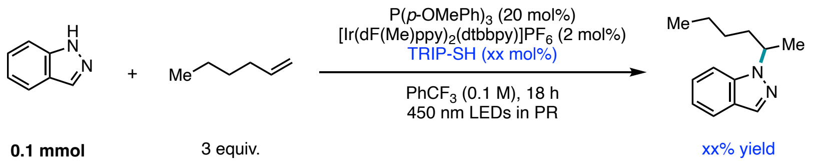

Thiol loading (mol%)	Yield
2	7%
5	20%
10	43%
20	15%
50	10%

Table S27. Phosphine loading screen.

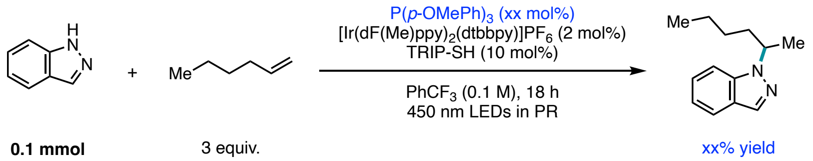

Phosphine loading (mol%)	Yield
5	0%
10	7%
20	43%
50	53%

Table S28. Cross-condition screen.

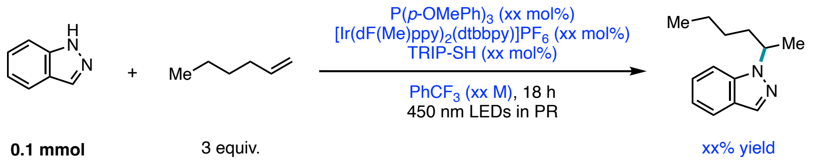

Solvent concentration (M)	Phosphine loading (mol%)	Photocatalyst loading (mol%)	Thiol loading (mol%)	Yield
0.1	20	2	10	43%
0.1	50	1	50	56%
0.05	50	1	50	65%
0.05	50	1	10	68%
0.4	50	5	50	20%
0.05	20	1	10	76%

3. Functional Group Robustness Screen

A functional group robustness screen[2] was performed following General Procedure A with the addition of 0.1 mmol (1 equiv.) of additive.

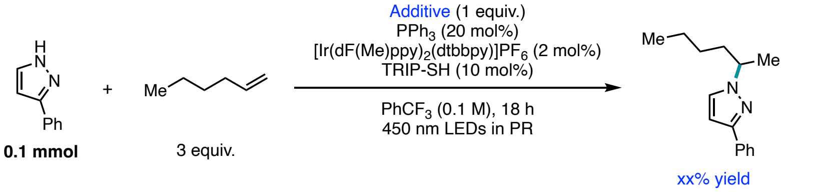

Table S29. Functional group robustness additive screen.

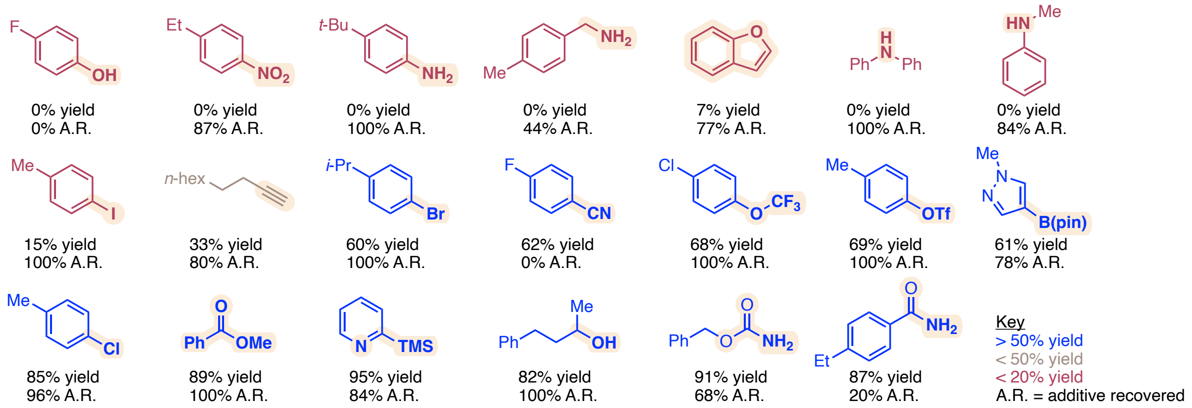

Yields and additive recovery determined by ^{1}H NMR in $CDCl_3$ with 1,3,5-TMB as internal standard.

Functional groups such as aniline, phenol, and nitroarene that can undergo competitive oxidation or reduction by the photocatalyst were deleterious to the standard reaction. Halides such as Br and I, which are often coupling partners in transition-metal catalyzed methods, resulted in decreased yield of the standard reaction, but could be fully recovered at the end of the reaction. Electron-withdrawing groups such as trifluoromethoxy arene and triflate had some moderate influence on the standard reaction yield but are compatible under these conditions. Benzoates, pyridines, alcohols, and carbamates were largely unreactive under these conditions, resulting in minor influence on the yield of the standard reaction.

4. General Procedure B and Characterization

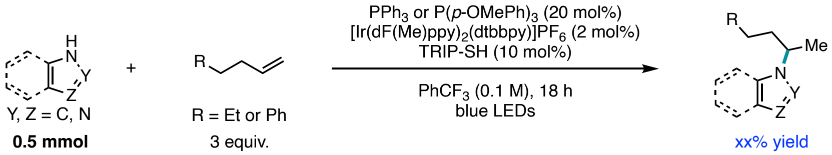

Procedure B1 (for all N–H substrates except indazoles) - Reaction setup (0.50 mmol scale): A 2-dram glass vial equipped with a stir bar was charged with the N–H heteroaromatic substrate. The vial was loosely covered with a cap and brought into a N_2-filled glovebox. Phosphine (0.10 mmol, 0.20 equiv.) and $[Ir(dF(Me)ppy)_2(dtbbpy)]PF_6$ (10.1 mg, 0.010 mmol, 0.020 equiv.) were then added to the vial, followed by 5.0 mL of α,α,α-trifluorotoluene (0.1 M), resulting in a suspension. The reaction vial was charged with TRIP-SH (11.8 µL, 0.050 mmol, 0.10 equiv.) and alkene (1.5 mmol, 3.0 equiv.). The vial was capped, sealed with electrical tape, and removed from the glovebox. The reaction was stirred at 1500 rpm for 18 hours while irradiating within the photoreactor (450 nm, 100% intensity, 2800 rpm fan speed). At the end of 18 hours, the vial was removed from the photoreactor. The reaction was then opened to air, and concentrated *in vacuo* to give the crude product which was purified by automated flash chromatography eluting with 0% to 100% ethyl acetate or diethyl ether in hexanes (unless otherwise stated) to provide the desired product. Regioisomeric ratios were determined by ^1H NMR ratios. Regioisomeric compounds were further characterized by 2D-NMR (HSQC and HMBC) unless otherwise unambiguously assigned by ^1H NMR.

Procedure B2 (for indazoles) - Reaction setup (0.50 mmol scale): A 20 mL glass vial equipped with a stir bar was charged with the N–H heteroaromatic substrate. The vial was loosely covered with a cap and brought into a N_2-filled glovebox. Phosphine (0.10 mmol, 0.2 equiv.) and $[Ir(dF(Me)ppy)_2(dtbbpy)]PF_6$ (10.1 mg, 0.010 mmol, 0.020 equiv.) were then added to the vial, followed by 5.0 mL of α,α,α-trifluorotoluene (0.1 M), resulting in a suspension. The reaction vial was charged with TRIP-SH (11.8 µL, 0.050 mmol, 0.10 equiv.) and alkene (1.5 mmol, 3.0 equiv.). The vial was capped, sealed with electrical tape, and removed from the glovebox. The reaction was stirred at 800 rpm for 16 hours while irradiating with two 427 nm PR160L Kessil Lamps at 50% intensity, placed horizontally opposite each other, 2 cm from the reaction vial. At the end of 16 hours, the lamps were turned off. The reaction was then opened to air, and concentrated *in vacuo* to give the crude product which was purified by automated flash chromatography eluting with 0 to 100% ethyl acetate or diethyl ether in hexanes (unless otherwise stated) to provide the desired product. Regioisomeric ratios were determined by ^1H NMR ratios. Regioisomeric compounds were further characterized by 2D-NMR (HSQC, HMBC, NOESY) unless otherwise unambiguously assigned by ^1H NMR.

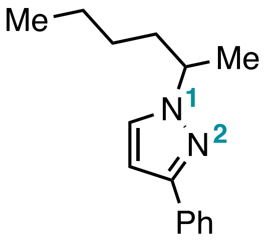

2

1-(hexan-2-yl)-3-phenyl-1*H*-pyrazole (2): The reaction was set up following general procedure B1 using 3-phenylpyrazole (72.1 mg, 0.500 mmol, 1.00 equiv.), 1-hexene (188 μL, 1.50 mmol, 3.00 equiv.), and triphenylphosphine (26.2 mg, 0.100 mmol, 0.200 equiv.). The crude product was purified using silica gel flash column chromatography eluting with Et_2O/Hex (0% → 10%, eluted 10%) to give the title compound as a pale-yellow oil.

Run 1: 95 mg (83% yield) **Run 2:** 98 mg (86% yield) **Average:** 85% yield

IR (FT-ATR, cm^{-1}, neat): 3063, 3034, 2956, 2930, 2859, 1606, 1496, 1457, 1415, 1375, 1357, 1304, 1281, 1260, 1216, 1073, 1046, 947, 744, 691.

^{1}H NMR (500 MHz, $CDCl_3$): δ 7.82 (d, *J* = 8.0, 2H), 7.41 (d, *J* = 2.4, 1H), 7.40 – 7.37 (m, 2H), 7.31 – 7.26 (m, 1H), 6.53 (d, *J* = 2.3 Hz, 1H), 4.40 – 4.29 (m, 1H), 1.98 – 1.91 (m, 1H), 1.79 – 1.72 (m, 1H), 1.53 (d, *J* = 6.7 Hz, 3H), 1.36 – 1.28 (m, 2H), 1.28 – 1.22 (m, 1H), 1.22 – 1.13 (m, 1H), 0.88 (t, *J* = 7.1 Hz, 3H).

^{13}C{^{1}H} NMR (126 MHz, $CDCl_3$): δ 150.7, 134.1, 128.7, 128.1, 127.4, 125.7, 102.3, 58.7, 37.1, 28.5, 22.5, 21.5, 14.1.

HRMS (APCI): Exact Mass calculated for $[C_{15}H_{20}N_2 + H]^+$ requires *m/z* = 229.1699. Found 229.1699.

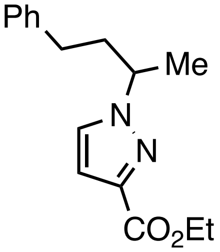

3

Ethyl 1-(4-phenylbutan-2-yl)-1*H*-pyrazole-3-carboxylate (3): The reaction was set up following general procedure B1 using ethyl 1H-pyrazole-3-carboxylate (90.1 mg, 0.500 mmol, 1.00 equiv.), but-3-en-1-ylbenzene (230 μL, 1.50 mmol, 3.00 equiv.), and triphenylphosphine (26.2 mg, 0.100 mmol, 0.200 equiv.). The crude product was purified using silica gel flash column chromatography eluting with EtOAc/Hex (0% → 30%, eluted 30%) to give the title compound as a pale-yellow oil.

Run 1: 100 mg (74% yield) **Run 2:** 112 mg (82% yield) **Average:** 78% yield

IR (FT-ATR, cm^{-1}, neat): 3025, 2978, 2932, 2858, 1714, 1469, 1454, 1418, 1375, 1344, 1283, 1215, 1172, 1145, 1114, 1080, 1051, 1023, 991, 759, 698.

^{1}H NMR (500 MHz, $CDCl_3$): δ 7.41 (d, *J* = 2.4 Hz, 1H), 7.30 – 7.25 (m, 2H), 7.20 – 7.15 (m, 1H), 7.13 – 7.08 (m, 2H), 6.82 (d, *J* = 2.3 Hz, 1H), 4.49 – 4.39 (m, 1H), 4.41 (q, *J* = 7.1 Hz, 2H), 2.54 – 2.44 (m, 2H), 2.34 – 2.26 (m, 1H), 2.13 – 2.02 (m, 1H), 1.54 (d, *J* = 6.8 Hz, 3H), 1.40 (t, *J* = 7.1 Hz, 3H).

^{13}C{^{1}H} NMR (126 MHz, $CDCl_3$): δ 162.7, 143.3, 140.9, 128.6, 128.5, 128.4, 126.2, 108.9, 61.0, 58.9, 38.6, 32.4, 21.5, 14.6.

HRMS (APCI): Exact Mass calculated for $[C_{16}H_{20}N_2O_2 + H]^+$ requires *m/z* = 273.1598. Found 273.1597.

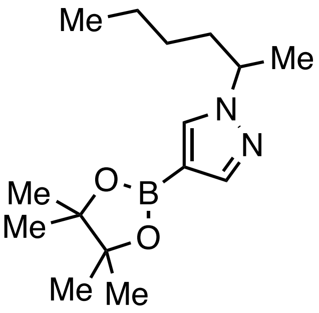

4

1-(hexan-2-yl)-4-(4,4,5,5-tetramethyl-1,3,2-dioxaborolan-2-yl)-1*H*-pyrazole (4): The reaction was set up following general procedure B1 using 4-(4,4,5,5-tetramethyl-1,3,2-dioxaborolan-2-yl)-1H-pyrazole (97.0 mg, 0.500 mmol, 1.00 equiv.), 1-hexene (188 μL, 1.50 mmol, 3.00 equiv.), and triphenylphosphine (26.2 mg, 0.100 mmol, 0.200 equiv.). The crude product was purified using silica gel flash column chromatography eluting with Et_2O/Hex (0% → 20%, eluted 20%) to give the title compound as a pale-yellow oil.

Run 1: 34 mg (24% yield) **Run 2:** 39 mg (28% yield) **Average:** 26% yield

IR (FT-ATR, cm^{-1}, neat): 2976, 2957, 2930, 2862, 1553, 1461, 1404, 1380, 1351, 1292, 1242, 1214, 1165, 1142, 1111, 1005, 985, 869, 857, 829, 790, 692.

^{1}H NMR (500 MHz, $CDCl_3$): δ 7.79 (s, 1H), 7.70 (d, *J* = 0.7 Hz, 1H), 4.37 – 4.26 (m, 1H), 1.93 – 1.86 (m, 1H), 1.75 – 1.68 (m, 1H), 1.48 (d, *J* = 6.8 Hz, 3H), 1.32 (s, 12H), 1.30 – 1.24 (m, 2H), 1.22 – 1.15 (m, 1H), 1.14 – 1.05 (m, 1H), 0.85 (t, *J* = 7.3 Hz, 3H).

^{13}C{^{1}H} NMR (126 MHz, $CDCl_3$): δ 145.1, 134.2, 83.4, 58.5, 37.0, 28.4, 25.0, 25.0, 22.5, 21.5, 14.1. The carbon atom bound to boron is not observed due to quadrupolar relaxation.

HRMS (APCI): Exact Mass calculated for $[C_{15}H_{27}N_2O_2B + H]^+$ requires *m/z* = 279.2238. Found 279.2238.

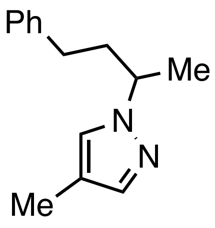

5

4-methyl-1-(4-phenylbutan-2-yl)-1*H*-pyrazole (5): The reaction was set up following general procedure B1 using 4-methyl-1H-pyrazole (41.1 mg, 0.500 mmol, 1.00 equiv.), but-3-en-1-ylbenzene (230 μL, 1.50 mmol, 3.00 equiv.), and triphenylphosphine (26.2 mg, 0.100 mmol, 0.200 equiv.). The crude product was purified using silica gel flash column chromatography eluting with EtOAc/Hex (0% → 20%, eluted 20%) to give the title compound as an orange oil.

Run 1: 60 mg (56% yield) **Run 2:** 62 mg (58% yield) **Average:** 57% yield

IR (FT-ATR, cm^{-1}, neat): 3026, 2972, 2930, 2864, 1603, 1495, 1454, 1429, 1388, 1355, 1343, 1309, 1193, 1160, 1014, 978, 841, 785, 743, 700.

^{1}H NMR (500 MHz, $CDCl_3$): δ 7.34 (s, 1H), 7.30 – 7.26 (m, 1H), 7.26 – 7.25 (m, 1H), 7.21 – 7.09 (m, 4H), 4.27 – 4.20 (m, 1H), 2.47 (dd, *J* = 8.7, 6.9 Hz, 2H), 2.30 – 2.19 (m, 1H), 2.09 (s, 3H), 2.06 – 1.95 (m, 1H), 1.48 (d, *J* = 6.8 Hz, 3H).

^{13}C{^{1}H} NMR (126 MHz, $CDCl_3$): δ 141.5, 139.3, 128.6, 128.5, 126.3, 126.1, 115.6, 57.4, 38.8, 32.5, 21.7, 9.1.

HRMS (APCI): Exact Mass calculated for $[C_{14}H_{18}N_2 + H]^+$ requires *m/z* = 215.1543. Found 215.1543.

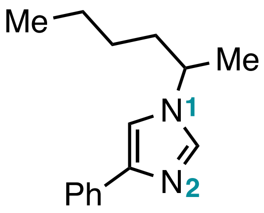

6

1-(hexan-2-yl)-4-phenyl-1*H*-imidazole (6): The reaction was set up following general procedure B1 using 4-phenylimidazole (72.1 mg, 0.500 mmol, 1.00 equiv.), 1-hexene (188 µL, 1.50 mmol, 3.00 equiv.), and tris(4-methoxyphenyl)phosphine (35.2 mg, 0.100 mmol, 0.200 equiv.). The crude product was purified using silica gel flash column chromatography eluting with Et_2O/Hex (0% → 50%, eluted 50%) to give the title compound as a red oil.

Run 1: 82 mg (72% yield) **Run 2:** 89 mg (78% yield) **Average:** 75% yield

IR (FT-ATR, cm^{-1}, neat): 2960, 2933, 2861, 1607, 1493, 1483, 1467, 1457, 1413, 1380, 1361, 1220, 1187, 1066, 904, 724.

^{1}H NMR (500 MHz, $CDCl_3$): δ 7.82 – 7.74 (m, 2H), 7.54 (d, *J* = 1.3 Hz, 1H), 7.40 – 7.33 (m, 2H), 7.25 – 7.20 (m, 2H), 4.18 – 4.07 (m, 1H), 1.84 – 1.69 (m, 2H), 1.51 (d, *J* = 6.8 Hz, 3H), 1.34 – 1.15 (m, 4H), 0.87 (t, *J* = 7.2 Hz, 3H).

$^{13}C\{^1H\}$ NMR (126 MHz, $CDCl_3$): δ 142.2, 136.2, 134.5, 128.7, 126.8, 124.8, 112.5, 54.2, 37.7, 28.4, 22.5, 22.4, 14.0.

HRMS (APCI): Exact Mass calculated for $[C_{15}H_{20}N_2 + H]^+$ requires *m/z* = 229.1699. Found 229.1699.

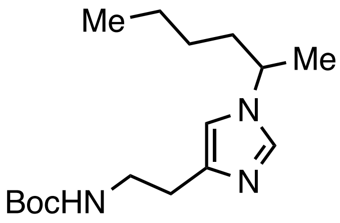

7

***tert*-butyl (2-(1-(hexan-2-yl)-1*H*-imidazol-4-yl)ethyl)carbamate (7):** The reaction was set up following general procedure B1 using *tert*-butyl (2-(1*H*-imidazol-4-yl)ethyl)carbamate (106 mg, 0.500 mmol, 1.00 equiv.), 1-hexene (188 µL, 1.50 mmol, 3.00 equiv.), and tris(4-methoxyphenyl)phosphine (35.2 mg, 0.100 mmol, 0.200 equiv.). The crude product was purified using silica gel flash column chromatography eluting with EtOAc/Hex (0% → 100%, eluted 100%) to give the title compound as a red oil.

Run 1: 93 mg (63% yield) **Run 2:** 94 mg (64% yield) **Average:** 64% yield

IR (FT-ATR, cm^{-1}, neat): 3341, 3229, 2966, 2932, 2931, 2862, 1702, 1493, 1455, 1416, 1390, 1364, 1268, 1251, 1171, 1055, 992, 956, 869, 818, 780, 756, 734.

^{1}H NMR (500 MHz, $CDCl_3$): δ 7.40 (d, *J* = 1.4 Hz, 1H), 6.72 – 6.67 (m, 1H), 5.14 (br, 1H), 4.03 (h, *J* = 6.8 Hz, 1H), 3.41 (q, *J* = 6.4 Hz, 2H), 2.73 (t, *J* = 6.6 Hz, 2H), 1.75 – 1.62 (m, 2H), 1.43 (d, *J* = 6.8 Hz, 3H), 1.43 (s, 9H), 1.37 – 1.04 (m, 4H), 0.86 (t, *J* = 7.2 Hz, 3H).

$^{13}C\{^1H\}$ NMR (126 MHz, $CDCl_3$): δ 156.2, 140.2, 135.4, 113.5, 79.0, 53.9, 40.5, 37.6, 28.6, 28.6, 28.4, 22.4, 22.3, 14.0.

HRMS (APCI): Exact Mass calculated for $[C_{16}H_{29}N_3O_2 + H]^+$ requires *m/z* = 296.2333. Found 296.2333.

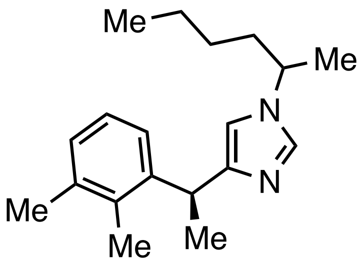

8

4-((*S*)-1-(2,3-dimethylphenyl)ethyl)-1-(hexan-2-yl)-1*H*-imidazole (8): The reaction was set up following general procedure B1 using (*S*)-4-(1-(2,3-dimethylphenyl)ethyl)-1*H*-imidazole (100 mg, 0.500 mmol, 1.00 equiv.), 1-hexene (188 µL, 1.50 mmol, 3.00 equiv.), and tris(4-methoxyphenyl)phosphine (35.2 mg, 0.100 mmol, 0.200 equiv.). The crude product was purified using silica gel flash column chromatography eluting with Et_2O/Hex (0% → 100%, eluted 100%) to give the title compound as a 1:1 mixture of diastereomers as a yellow oil. D.r. determined by ^1H NMR integration of the doublets at 1.41 and 1.40 ppm assigned as the diastereomeric benzylic methyl groups.

Run 1: 44 mg (31% yield) **Run 2:** 37 mg (26% yield) **Average:** 29% yield

IR (FT-ATR, cm^{-1}, neat): 2960, 2930, 2861, 1585, 1545, 1491, 1465, 1457, 1413, 1378, 1236, 1217, 1163, 1122, 1098, 987, 954, 910, 814, 787, 729.

^{1}H NMR (500 MHz, $CDCl_3$): δ 7.43 (d, *J* = 1.8 Hz, 1H), 7.10 – 6.98 (m, 3H), 6.47 – 6.42 (m, 1H), 4.37 (q, *J* = 7.1 Hz, 1H), 4.00 (h, *J* = 6.9 Hz, 1H), 2.29 (s, 3H), 2.24 (d, *J* = 3.7 Hz, 3H), 1.70 – 1.63 (m, 2H), 1.58 (d, *J* = 7.1 Hz, 3H), [diastereomeric: 1.41 (d, *J* = 6.9 Hz, 1.5H*), 1.40 (d, *J* = 6.7 Hz, 1.5H*)], 1.31 – 1.23 (m, 2H), 1.21 – 1.14 (m, 1H), 1.13 – 1.06 (m, 1H), 0.85 (t, *J* = 7.2 Hz, 3H).
*diastereomeric CH_3 groups

^{13}C{^{1}H} NMR (126 MHz, $CDCl_3$): δ 147.5, 147.4, 144.1, 144.0, 136.7, 135.2, 135.1, 134.4, 134.3, 127.8, 125.5, 124.7, 124.7, 112.9, 112.8, 53.8, 37.6, 37.5, 35.6, 35.6, 28.4, 28.4, 22.4, 22.2, 22.2, 21.1, 15.0, 14.0.
Carbon signals reported for the mixture of diastereomers.

HRMS (APCI): Exact Mass calculated for $[C_{19}H_{28}N_2 + H]^+$ requires *m/z* = 285.2325. Found 285.2325.

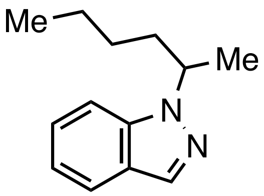

9

1-(hexan-2-yl)-1*H*-indazole (9): The reaction was set up following general procedure B2 using 1H-indazole (59.1 mg, 0.500 mmol, 1.00 equiv.), 1-hexene (188 µL, 1.50 mmol, 3.00 equiv.), and tris(4-methoxyphenyl)phosphine (35.2 mg, 0.100 mmol, 0.200 equiv.). The crude product was purified using silica gel flash column chromatography eluting with Et_2O/Hex (0% → 10%, eluted 8%) to give the title compound as a yellow oil.

Run 1: 61 mg (60% yield) **Run 2:** 61 mg (60% yield) **Average:** 60% yield

IR (FT-ATR, cm^{-1}, neat): 3059, 2957, 2931, 2859, 1615, 1497, 1464, 1424, 1370, 1223, 1192, 1177, 1121, 1008, 909, 844, 826, 768, 751, 738.

^{1}H NMR (500 MHz, $CDCl_3$): δ 8.02 (s, 1H), 7.73 (d, *J* = 8.1, 1H), 7.43 (d, *J* = 8.5, 1H), 7.35 (t, *J* = 6.6, 1H), 7.13 (t, *J* = 6.8, 1H), 4.70 – 4.59 (m, 1H), 2.15 – 2.08 (m, 1H), 1.89 – 1.82 (m, 1H), 1.57 (d, *J* = 6.7, 3H), 1.36 – 1.24 (m, 2H), 1.27 – 1.13 (m, 1H), 1.09 – 1.01 (m, 1H), 0.82 (t, *J* = 7.3, 3H).

$^{13}C\{^1H\}$ NMR (126 MHz, CDCl$_3$): δ 139.4, 132.8, 125.9, 124.0, 121.2, 120.4, 109.2, 54.8, 36.4, 28.8, 22.6, 21.0, 14.1.
HRMS (APCI): Exact Mass calculated for $[C_{13}H_{18}N_2 + H]^+$ requires *m/z* = 203.1543. Found 203.1543.

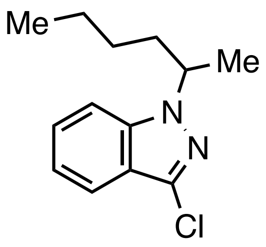

10

3-chloro-1-(hexan-2-yl)-1*H*-indazole (10): The reaction was set up following general procedure B2 using 3-chloro-1H-indazole (76.3 mg, 0.500 mmol, 1.00 equiv.), 1-hexene (188 μL, 1.50 mmol, 3.00 equiv.), and triphenylphosphine (26.23 mg, 0.100 mmol, 0.200 equiv.). The crude product was purified using silica gel flash column chromatography eluting with Et_2O/Hex (0% → 10%, eluted 10%) to give the title compound as an orange oil.
Run 1: 92 mg (78% yield) **Run 2:** 96 mg (81% yield) **Average:** 80% yield
IR (FT-ATR, cm^{-1}, neat): 3058, 2931, 2871, 2860, 1616, 1493, 1464, 1408, 1336, 1224, 1197, 1129, 1100, 1061, 1043, 1005, 968, 768, 742.
^1H NMR (500 MHz, CDCl$_3$): δ 7.67 (dt, *J* = 8.2, 1.0 Hz, 1H), 7.40 (t, *J* = 1.2 Hz, 1H), 7.39 (d, *J* = 1.0 Hz, 1H), 7.21 – 7.15 (m, 1H), 4.63 – 4.52 (m, 1H), 2.12 – 2.05 (m, 1H), 1.86 – 1.79 (m, 1H), 1.55 (d, *J* = 6.7 Hz, 3H), 1.32 – 1.25 (m, 2H), 1.22 – 1.15 (m, 1H), 1.11 – 1.03 (m, 1H), 0.82 (t, *J* = 7.3 Hz, 3H).
$^{13}C\{^1H\}$ NMR (126 MHz, CDCl$_3$): δ 140.7, 132.5, 127.2, 121.1, 120.9, 119.9, 109.5, 55.5, 36.3, 28.8, 22.5, 21.0, 14.1.
HRMS (APCI): Exact Mass calculated for $[C_{13}H_{17}N_2Cl + H]^+$ requires *m/z* = 237.1153. Found 237.1154.

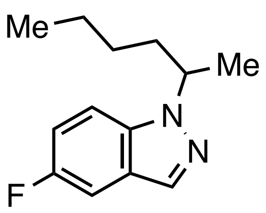

11

5-fluoro-1-(hexan-2-yl)-1*H*-indazole (11): The reaction was set up following general procedure B2 using 5-fluoro-1H-indazole (68.1 mg, 0.500 mmol, 1.00 equiv.), 1-hexene (188 μL, 1.50 mmol, 3.00 equiv.), and triphenylphosphine (26.2 mg, 0.100 mmol, 0.200 equiv.). The crude product was purified using silica gel flash column chromatography eluting with Et_2O/Hex (0% → 10%, eluted 10%) to give the title compound as a colorless oil.
Run 1: 75 mg (68% yield) **Run 2:** 68 mg (62% yield) **Average:** 65% yield
IR (FT-ATR, cm^{-1}, neat): 2956, 2933, 2859, 1611, 1582, 1502, 1449, 1419, 1377, 1358, 1344, 1297, 1264, 1247, 1215, 1188, 1136, 1120, 1074, 1034, 1019, 983, 954, 860, 829, 797, 749, 694.
^1H NMR (500 MHz, CDCl$_3$): δ 7.97 (s, 1H), 7.40 – 7.35 (m, 1H), 7.35 – 7.32 (m, 1H), 7.13 (td, *J* = 9.0, 2.4 Hz, 1H), 4.60 (m, 1H), 2.09 (m, 1H), 1.85 (m, 1H), 1.56 (d, *J* = 6.7 Hz, 3H), 1.36 – 1.23 (m, 2H), 1.22 – 1.13 (m, 1H), 1.09 – 0.96 (m, 1H), 0.82 (t, *J* = 7.3 Hz, 3H).

$^{13}C\{^1H\}$ NMR (126 MHz, $CDCl_3$): δ 157.8 (d, *J* = 237.5 Hz), 136.4, 132.5 (d, *J* = 5.7 Hz), 123.7 (d, *J* = 10.0 Hz), 115.7 (d, *J* = 27.7 Hz), 110.1 (d, *J* = 9.6 Hz), 105.0 (d, *J* = 23.3 Hz), 55.2, 36.4, 28.8, 22.5, 21.0, 14.1.
$^{19}F\{^1H\}$ NMR (376 MHz, $CDCl_3$) δ -123.79.
HRMS (APCI): Exact Mass calculated for $[C_{13}H_{17}N_2F + H]^+$ requires *m/z* = 221.1449. Found 221.1448.

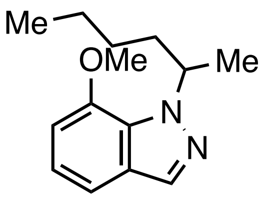

12

1-(hexan-2-yl)-7-methoxy-1*H*-indazole (12): The reaction was set up following general procedure B2 using 7-methoxy-1H-indazole (59.1 mg, 0.500 mmol, 1.00 equiv.), 1-hexene (188 µL, 1.50 mmol, 3.00 equiv.), and tris(4-methoxyphenyl)phosphine (35.2 mg, 0.100 mmol, 0.200 equiv.). The crude product was purified using silica gel flash column chromatography eluting with Et_2O/Hex (0% → 10%, eluted 6%) to give the title compound as a yellow oil.
Run 1: 59 mg (51% yield) **Run 2:** 54 mg (47% yield) **Average:** 49% yield
IR (FT-ATR, cm^{-1}, neat): 2959, 2933, 2859, 1580, 1517, 1466, 1452, 1415, 1392, 1375, 1336, 1321, 1265, 1231, 1102, 1018, 984, 905, 854, 832, 773, 726, 693.
^1H NMR (500 MHz, $CDCl_3$): δ 7.97 (s, 1H), 7.28 (d, *J* = 8.0 Hz, 1H), 7.01 (t, *J* = 7.8 Hz, 1H), 6.70 (d, *J* = 7.5 Hz, 1H), 5.37 – 5.30 (m, 1H), 3.97 (s, 3H), 2.15 – 2.03 (m, 1H), 1.81 – 1.76 (m, 1H), 1.53 (d, *J* = 6.6 Hz, 3H), 1.34 – 1.25 (m, 2H), 1.23 – 1.15 (m, 1H), 1.09 – 1.01 (m, 1H), 0.82 (t, *J* = 7.2 Hz, 3H).
$^{13}C\{^1H\}$ NMR (126 MHz, $CDCl_3$): δ 146.4, 133.1, 131.1, 125.9, 120.9, 113.2, 105.2, 56.5, 55.5, 37.1, 28.6, 22.6, 21.7, 14.1.
HRMS (APCI): Exact Mass calculated for $[C_{14}H_{20}N_2O + H]^+$ requires *m/z* = 233.1648. Found 233.1647.

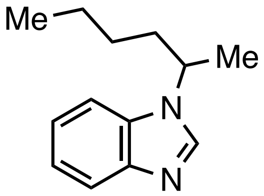

13

1-(hexan-2-yl)-1*H*-benzo[*d*]imidazole (13): The reaction was set up following general procedure B1 using 1H-benzo[d]imidazole (59.1 mg, 0.500 mmol, 1.00 equiv.), 1-hexene (188 µL, 1.50 mmol, 3.00 equiv.), and tris(4-methoxyphenyl)phosphine (35.2 mg, 0.100 mmol, 0.200 equiv.). The crude product was purified using silica gel flash column chromatography eluting with EtOAc/Hex (0% → 50%, eluted 50%) to give the title compound as a red oil.
Run 1: 54 mg (53% yield) **Run 2:** 58 mg (57% yield) **Average:** 55% yield
IR (FT-ATR, cm^{-1}, neat): 3081, 3053, 2956, 2931, 2859, 1613, 1487, 1457, 1402, 1379, 1368, 1328, 1312, 1284, 1231, 1204, 1008, 889, 779, 767, 741.
^1H NMR (500 MHz, $CDCl_3$): δ 7.95 (s, 1H), 7.86 – 7.77 (m, 1H), 7.46 – 7.38 (m, 1H), 7.30 – 7.27 (m, 2H), 4.48 – 4.41 (m, 1H), 2.04 – 1.96 (m, 1H), 1.91 – 1.84 (m, 1H), 1.60 (d, *J* = 6.8 Hz, 3H), 1.38 – 1.28 (m, 2H), 1.28 – 1.23 (m, 1H), 1.22 – 1.13 (m, 1H), 0.85 (t, *J* = 7.2 Hz, 3H).

$^{13}C\{^1H\}$ **NMR (126 MHz, CDCl$_3$):** δ 144.2, 141.1, 133.5, 122.7, 122.1, 120.6, 110.3, 52.5, 36.4, 28.5, 22.5, 21.3, 14.0.
HRMS (APCI): Exact Mass calculated for $[C_{13}H_{18}N_2 + H]^+$ requires *m/z* = 203.1543. Found 203.1543.

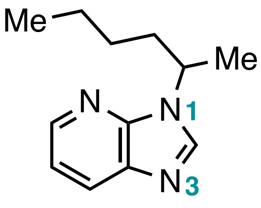

14

3-(hexan-2-yl)-3*H*-imidazo[4,5-*b*]pyridine (14): The reaction was set up following general procedure B1 using 1H-imidazo[4,5-b]pyridine (59.6 mg, 0.500 mmol, 1.00 equiv.), 1-hexene (188 μL, 1.50 mmol, 3.00 equiv.), and tris(4-methoxyphenyl)phosphine (35.2 mg, 0.100 mmol, 0.200 equiv.). The crude product was purified using silica gel flash column chromatography eluting with EtOAc/Hex (0% → 50%, eluted 50%) to give the title compound as an orange oil.
Run 1: 37 mg (36% yield) **Run 2:** 47 mg (46% yield) **Average:** 41% yield
IR (FT-ATR, cm^{-1}, neat): 3079, 3054, 2956, 2930, 2860, 1599, 1580, 1491, 1457, 1405, 1378, 1287, 1275, 1233, 1204, 800, 775.
^1H **NMR (500 MHz, CDCl$_3$):** δ 8.39 (dd, *J* = 4.7, 1.5 Hz, 1H), 8.10 (s, 1H), 8.07 (dd, *J* = 8.0, 1.5 Hz, 1H), 7.23 (dd, *J* = 8.0, 4.7 Hz, 1H), 4.86 – 4.79 (m, 1H), 2.07 – 2.00 (m, 1H), 1.94 – 1.87 (m, 1H), 1.61 (d, *J* = 6.9 Hz, 3H), 1.39 – 1.29 (m, 2H), 1.29 – 1.23 (m, 1H), 1.21 – 1.10 (m, 1H), 0.84 (t, *J* = 7.2 Hz, 3H).
$^{13}C\{^1H\}$ **NMR (126 MHz, CDCl$_3$):** δ 147.0, 144.1, 142.1, 135.7, 128.0, 118.3, 51.0, 36.5, 28.5, 22.4, 21.4, 14.0.
HRMS (APCI): Exact Mass calculated for $[C_{12}H_{17}N_3 + H]^+$ requires *m/z* = 204.1495. Found 204.1495.

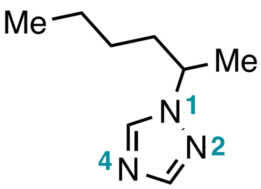

15

1-(hexan-2-yl)-1*H*-1,2,4-triazole (15): The reaction was set up following general procedure B1 using 1*H*-1,2,4-triazole (34.5 mg, 0.500 mmol, 1.00 equiv.), 1-hexene (188 μL, 1.50 mmol, 3.00 equiv.), and triphenylphosphine (26.2 mg, 0.100 mmol, 0.200 equiv.). The crude product was purified using silica gel flash column chromatography eluting with EtOAc/Hex (0% → 100%, eluted 100%) to give the title compound as a pale yellow oil.
Run 1: 58 mg (76% yield) **Run 2:** 46 mg (60% yield) **Average:** 68% yield
IR (FT-ATR, cm^{-1}, neat): 3118, 2958, 2933, 2863, 1465, 1457, 1436, 1383, 1274, 1200, 1140, 1006, 988, 958, 944, 908, 876, 844, 729, 682, 667.
^1H **NMR (500 MHz, CDCl$_3$):** δ 8.06 (s, 1H), 7.94 (s, 1H), 4.41 – 4.32 (m, 1H), 1.97 – 1.88 (m, 1H), 1.82 – 1.69 (m, 1H), 1.52 (d, *J* = 6.7 Hz, 3H), 1.36 – 1.24 (m, 2H), 1.22 – 1.01 (m, 2H), 0.86 (t, *J* = 7.2 Hz, 3H).
$^{13}C\{^1H\}$ **NMR (126 MHz, CDCl$_3$):** δ 151.8, 141.7, 56.8, 36.4, 28.2, 22.4, 21.1, 14.0.

HRMS (APCI): Exact Mass calculated for $[C_8H_{15}N_3 + H]^+$ requires m/z = 154.1339. Found 154.1336.

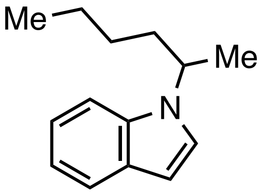

16

1-(hexan-2-yl)-1*H*-indole (16): The reaction was set up following general procedure B1 using 1H-indole (58.6 mg, 0.500 mmol, 1.00 equiv.), 1-hexene (188 µL, 1.50 mmol, 3.00 equiv.), and tris(4-methoxyphenyl)phosphine (35.2 mg, 0.100 mmol, 0.200 equiv.). The crude product was purified using silica gel flash column chromatography eluting with Hex (100%) to give the title compound as a colorless oil.

Run 1: 41 mg (41% yield) **Run 2:** 43 mg (43% yield) **Average:** 42% yield

IR (FT-ATR, cm^{-1}, neat): 2956, 2927, 2859, 1509, 1477, 1458, 1409, 1377, 1361, 1307, 1228, 1201, 1162, 1120, 1101, 1014, 883, 761, 735, 712.

^{1}H NMR (500 MHz, $CDCl_3$) δ 7.63 (d, J = 7.9, 1H), 7.38 (d, J = 8.3, 1H), 7.21 – 7.14 (m, 2H), 7.09 (m, 1H), 6.53 (d, J = 3.2, 1H), 4.52 – 4.45 (m, 1H), 1.95 – 1.88 (m, 1H), 1.85 – 1.76 (m, 1H), 1.50 (d, J = 6.8 Hz, 3H), 1.30 – 1.24 (m, 3H), 1.18 – 1.11 (m, 1H), 0.84 (t, J = 7.2 Hz, 3H).

^{13}C{^{1}H} NMR (126 MHz, $CDCl_3$) δ 136.0, 128.6, 124.1, 121.2, 121.0, 119.3, 109.6, 101.4, 51.7, 37.0, 28.7, 22.6, 21.5, 14.1.

HRMS (APCI): Exact Mass calculated for $[C_{14}H_{19}N + H]^+$ requires m/z = 202.1590. Found 202.1590.

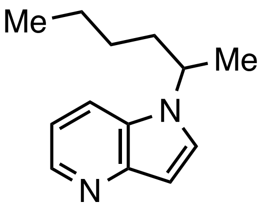

17

1-(hexan-2-yl)-1*H*-pyrrolo[3,2-*b*]pyridine (17): The reaction was set up following general procedure B1 using 1H-pyrrolo[3,2-b]pyridine (59.1 mg, 0.500 mmol, 1.00 equiv.), 1-hexene (188 µL, 1.500 mmol, 3.00 equiv.), and tris(4-methoxyphenyl)phosphine (35.2 mg, 0.100 mmol, 0.200 equiv.). The crude product was purified using silica gel flash column chromatography eluting with EtOAc/Hex (0% → 50%, eluted 50%) to give the title compound as a purple oil.

Run 1: 60 mg (59% yield) **Run 2:** 55 mg (54% yield) **Average:** 57% yield

IR (FT-ATR, cm^{-1}, neat): 3040, 2957, 2930, 2859, 1599, 1554, 1504, 1484, 1414, 1379, 1284, 1224, 1211, 1187, 1082, 772, 725.

^{1}H NMR (500 MHz, $CDCl_3$): δ 8.45 (d, J = 4.6 Hz, 1H), 7.65 (d, J = 8.5 Hz, 1H), 7.41 (d, J = 3.3 Hz, 1H), 7.09 (dd, J = 8.3, 4.6 Hz, 1H), 6.72 (d, J = 3.3 Hz, 1H), 4.48 – 4.37 (m, 1H), 1.95 – 1.86 (m, 1H), 1.86 – 1.77 (m, 1H), 1.52 (d, J = 6.8 Hz, 3H), 1.35 – 1.25 (m, 2H), 1.24 – 1.15 (m, 1H), 1.13 – 1.05 (m, 1H), 0.83 (t, J = 7.2 Hz, 3H).

^{13}C{^{1}H} NMR (126 MHz, $CDCl_3$): δ 146.9, 143.2, 128.9, 127.7, 116.7, 116.1, 102.6, 52.4, 37.0, 28.7, 22.6, 21.5, 14.0.

HRMS (APCI): Exact Mass calculated for $[C_{13}H_{18}N_2 + H]^+$ requires m/z = 203.1543. Found 203.1543.

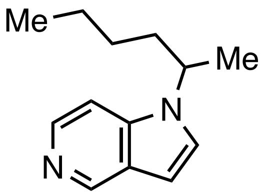

18

1-(hexan-2-yl)-1*H*-pyrrolo[3,2-*c*]pyridine (18): The reaction was set up following general procedure B1 using 1H-pyrrolo[3,2-c]pyridine (59.1 mg, 0.500 mmol, 1.00 equiv.), 1-hexene (188 μL, 1.50 mmol, 3.00 equiv.), and tris(4-methoxyphenyl)phosphine (35.2 mg, 0.100 mmol, 0.200 equiv.). The crude product was purified using silica gel flash column chromatography eluting with EtOAc (100%) to give the title compound as a yellow oil.

Run 1: 47 mg (47% yield) **Run 2:** 59 mg (59% yield) **Average:** 53% yield

IR (FT-ATR, cm^{-1}, neat): 3038, 2956, 2928, 2857, 1598, 1560, 1510, 1468, 1442, 1404, 1377, 1314, 1294, 1256, 1227, 1206, 1187, 1101, 1074, 1033, 889, 843, 723, 695.

^{1}H NMR (500 MHz, CDCl$_3$): δ 8.91 (d, *J* = 1.0 Hz, 1H), 8.29 (d, *J* = 5.9 Hz, 1H), 7.28 – 7.26 (m, 1H), 7.22 (d, *J* = 3.3 Hz, 1H), 6.64 (d, *J* = 3.2, 1H), 4.50 – 4.43 (m, 1H), 1.93 – 1.77 (m, 2H), 1.52 (d, *J* = 6.8 Hz, 3H), 1.32 – 1.25 (m, 2H), 1.24 – 1.17 (m, 1H), 1.13 – 1.02 (m, 1H), 0.83 (t, *J* = 7.2 Hz, 3H).

^{13}C{^{1}H} NMR (126 MHz, CDCl$_3$): δ 143.8, 140.1, 139.6, 125.6, 125.5, 105.1, 101.6, 52.2, 37.0, 28.6, 22.5, 21.5, 14.0.

HRMS (APCI): Exact Mass calculated for [$C_{13}H_{18}N_2$ + H]$^+$ requires *m/z* = 203.1543. Found 203.1543.

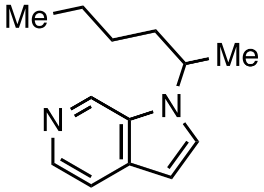

19

1-(hexan-2-yl)-1*H*-pyrrolo[2,3-*c*]pyridine (19): The reaction was set up following general procedure B1 using 1H-pyrrolo[2,3-c]pyridine (59.1 mg, 0.500 mmol, 1.00 equiv.), 1-hexene (188 μL, 1.500 mmol, 3.00 equiv.), and tris(4-methoxyphenyl)phosphine (35.2 mg, 0.100 mmol, 0.200 equiv.). The crude product was purified using silica gel flash column chromatography eluting with EtOAc/Hex (0% → 80%, eluted 80%) to give the title compound as an orange oil.

Run 1: 57 mg (56% yield) **Run 2:** 66 mg (65% yield) **Average:** 61% yield

IR (FT-ATR, cm^{-1}, neat): 3035, 2956, 2929, 2858, 1597, 1556, 1496, 1465, 1409, 1378, 1314, 1286, 1233, 1205, 1167, 1031, 891, 842, 814, 773, 731.

^{1}H NMR (500 MHz, CDCl$_3$): δ 8.83 (s, 1H), 8.22 (d, *J* = 5.5 Hz, 1H), 7.52 (dd, *J* = 5.5, 1.1 Hz, 1H), 7.32 (d, *J* = 3.2 Hz, 1H), 6.52 (dd, *J* = 3.1, 0.8 Hz, 1H), 4.60 – 4.53 (m, 1H), 1.98 – 1.79 (m, 2H), 1.56 (d, *J* = 6.8 Hz, 3H), 1.32 – 1.25 (m, 2H), 1.25 – 1.17 (m, 1H), 1.14 – 1.05 (m, 1H), 0.83 (t, *J* = 7.2 Hz, 3H).

^{13}C{^{1}H} NMR (126 MHz, CDCl$_3$): δ 138.3, 133.3, 133.1, 133.1, 128.2, 115.5, 101.0, 52.7, 37.1, 28.6, 22.5, 21.7, 14.0.

HRMS (APCI): Exact Mass calculated for [$C_{13}H_{18}N_2$ + H]$^+$ requires *m/z* = 203.1543. Found 203.1543.

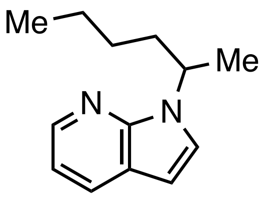

20

1-(hexan-2-yl)-1*H*-pyrrolo[2,3-*b*]pyridine (20): The reaction was set up following general procedure B1 using 1H-pyrrolo[2,3-b]pyridine (59.1 mg, 0.500 mmol, 1.00 equiv.), 1-hexene (188 µL, 1.50 mmol, 3.00 equiv.), and tris(4-methoxyphenyl)phosphine (35.2 mg, 0.100 mmol, 0.200 equiv.). The crude product was purified using silica gel flash column chromatography eluting with Et_2O/Hex (0% → 20%, eluted 15%) to give the title compound as an orange oil.

Run 1: 31 mg (31% yield) **Run 2:** 37 mg (37% yield) **Average:** 34% yield

IR (FT-ATR, cm^{-1}, neat): 3051, 2958, 2930, 2860, 1593, 1569, 1506, 1457, 1427, 1411, 1378, 1347, 1304, 1277, 1231, 1204, 905, 796, 773, 727.

^{1}H NMR (500 MHz, $CDCl_3$): δ 8.31 (dd, *J* = 4.7, 1.6 Hz, 1H), 7.90 (dd, *J* = 7.8, 1.6 Hz, 1H), 7.28 (d, *J* = 3.6 Hz, 1H), 7.04 (dd, *J* = 7.8, 4.6 Hz, 1H), 6.48 (d, *J* = 3.5 Hz, 1H), 5.09 – 5.02 (m, 1H), 1.94 – 1.77 (m, 2H), 1.49 (d, *J* = 6.8 Hz, 3H), 1.34 – 1.26 (m, 2H), 1.25 – 1.19 (m, 1H), 1.16 – 1.03 (m, 1H), 0.82 (t, *J* = 7.2 Hz, 3H).

^{13}C{^{1}H} NMR (126 MHz, $CDCl_3$): δ 147.5, 142.6, 128.8, 124.5, 120.7, 115.7, 99.7, 49.6, 37.0, 28.6, 22.6, 21.7, 14.1.

HRMS (APCI): Exact Mass calculated for $[C_{13}H_{18}N_2 + H]^+$ requires *m/z* = 203.1543. Found 203.1543.

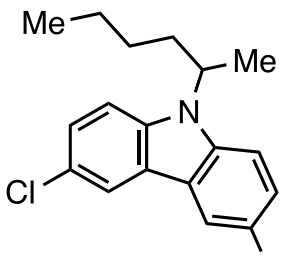

21

3,6-dichloro-9-(hexan-2-yl)-9*H*-carbazole (21): The reaction was set up following general procedure B1 using 3,6-dichloro-9H-carbazole (118.1 mg, 0.500 mmol, 1.00 equiv.), 1-hexene (188 µL, 1.50 mmol, 3.00 equiv.), and tris(4-methoxyphenyl)phosphine (35.2 mg, 0.100 mmol, 0.200 equiv.). The crude product was purified using silica gel flash column chromatography eluting with Hex (100%) to give the title compound as a colorless oil.

Run 1: 73 mg (46% yield) **Run 2:** 63 mg (39% yield) **Average:** 43% yield

IR (FT-ATR, cm^{-1}, neat): 2956, 2931, 2859, 1596, 1568, 1474, 1440, 1400, 1379, 1334, 1318, 1281, 1235, 1224, 1152, 1081, 1023, 866, 846, 800, 697.

^{1}H NMR (500 MHz, $CDCl_3$): δ 8.00 (s, 2H), 7.44 – 7.36 (m, 4H), 4.74 – 4.63 (m, 1H), 2.27 – 2.19 (m, 1H), 1.97 – 1.90 (m, 1H), 1.65 (d, *J* = 7.0 Hz, 3H), 1.29 – 1.19 (m, 3H), 1.03 – 0.95 (m, 1H), 0.77 (t, *J* = 7.1 Hz, 3H).

^{13}C{^{1}H} NMR (126 MHz, $CDCl_3$): δ 138.7, 126.3, 124.6, 123.5, 120.3, 111.4, 52.0, 34.8, 29.1, 22.5, 19.6, 14.0.

HRMS (APCI): Exact Mass calculated for $[C_{18}H_{19}NCl_2]^+$ requires *m/z* = 319.0895. Found 319.0906 (APCI).

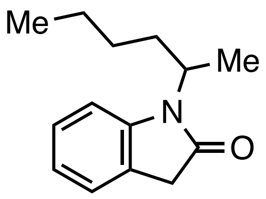

22

1-(hexan-2-yl)-indolin-2-one (22): The reaction was set up following general procedure B1 using indolin-2-one (66.6 mg, 0.500 mmol, 1.00 equiv.), 1-hexene (188 µL, 1.50 mmol, 3.00 equiv.), and tris(4-methoxyphenyl)phosphine (35.2 mg, 0.100 mmol, 0.200 equiv.). The crude product was purified using silica gel flash column chromatography eluting with Et_2O/Hex (0% → 30%, eluted 30%) to give the title compound as a yellow oil.

Run 1: 49 mg (45% yield) **Run 2:** 46 mg (42% yield) **Average:** 44% yield

IR (FT-ATR, cm^{-1}, neat): 2958, 2930, 2862, 1612, 1484, 1467, 1400, 1379, 1352, 1320, 1245, 1200, 1092, 904, 725.

^{1}H NMR (500 MHz, $CDCl_3$): δ 7.25 – 7.21 (m, 2H), 7.03 – 6.98 (m, 2H), 4.54 – 4.43 (m, 1H), 3.51 (d, *J* = 1.1 Hz, 2H), 2.05 – 1.96 (m, 1H), 1.77 – 1.69 (m, 1H), 1.44 (d, *J* = 7.1 Hz, 3H), 1.32 – 1.28 (m, 2H), 1.27 – 1.24 (m, 1H), 1.24 – 1.18 (m, 1H), 0.85 (t, *J* = 7.1 Hz, 3H).

^{13}C{^{1}H} NMR (126 MHz, $CDCl_3$): δ 175.1, 144.1, 127.7, 125.1, 124.7, 121.8, 110.0, 48.2, 36.1, 33.1, 29.1, 22.6, 18.0, 14.1.

HRMS (APCI): Exact Mass calculated for $[C_{14}H_{19}NO + H]^+$ requires *m/z* = 218.1539. Found 218.1537.

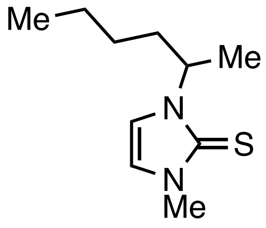

23

1-(hexan-2-yl)-3-methyl-1,3-dihydro-2*H*-imidazole-2-thione (23): The reaction was set up following general procedure B1 using 1-methyl-1,3-dihydro-2*H*-imidazole-2-thione (57.1 mg, 0.500 mmol, 1.00 equiv.), 1-hexene (188 µL, 1.50 mmol, 3.00 equiv.), and tris(4-methoxyphenyl)phosphine (35.2 mg, 0.100 mmol, 0.200 equiv.). The crude product was purified using silica gel flash column chromatography eluting with EtOAc/Hex (0% → 20%, eluted 20%) to give the title compound as a yellow oil.

Run 1: 52 mg (52% yield) **Run 2:** 52 mg (52% yield) **Average:** 52% yield

IR (FT-ATR, cm^{-1}, neat): 3118, 3087, 2955, 2927, 2858, 1568, 1452, 1407, 1377, 1359, 1323, 1278, 1224, 1146, 1101, 1033, 993, 976, 712, 675.

^{1}H NMR (500 MHz, $CDCl_3$): δ 6.72 – 6.66 (m, 2H), 5.02 – 4.95 (m, 1H), 3.63 (s, 3H), 1.74 – 1.63 (m, 2H), 1.60 – 1.59 (m, 1H), 1.37 – 1.25 (m, 1H), 1.32 (d, *J* = 6.8 Hz, 3H), 1.30 – 1.24 (m, 1H), 1.22 – 1.14 (m, 1H), 0.87 (t, *J* = 6.9 Hz, 3H).

^{13}C{^{1}H} NMR (126 MHz, $CDCl_3$): δ 118.2, 113.2, 53.2, 36.1, 35.1, 28.3, 22.6, 20.5, 14.1.

HRMS (APCI): Exact Mass calculated for $[C_{10}H_{18}N_2S + H]^+$ requires *m/z* = 199.1263. Found 199.1263.

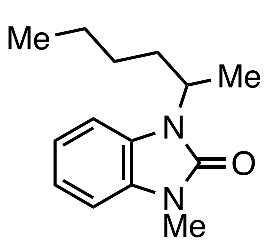

24

1-(hexan-2-yl)-3-methyl-1,3-dihydro-2*H*-benzo[*d*]imidazol-2-one (24): The reaction was set up following general procedure B1 using 1-methyl-1,3-dihydro-2*H*-benzo[*d*]imidazol-2-one (74.1 mg, 0.500 mmol, 1.00 equiv.), 1-hexene (188 μL, 1.50 mmol, 3.00 equiv.), and tris(4-methoxyphenyl)phosphine (35.2 mg, 0.100 mmol, 0.200 equiv.). The crude product was purified using silica gel flash column chromatography eluting with EtOAc/Hex (0% → 30%, eluted 30%) to give the title compound as a yellow oil.

Run 1: 81 mg (70% yield) **Run 2:** 93 mg (80% yield) **Average:** 85% yield

IR (FT-ATR, cm^{-1}, neat): 3060, 2955, 2930, 2858, 1697, 1618, 1605, 1495, 1457, 1431, 1407, 1388, 1362, 1319, 1250, 1224, 1186, 1126, 1088, 1019, 751, 731, 710.

^{1}H NMR (500 MHz, $CDCl_3$): δ 7.14 – 7.02 (m, 3H), 7.00 – 6.94 (m, 1H), 4.57 – 4.50 (m, 1H), 3.41 (s, 3H), 2.09 – 2.01 (m, 1H), 1.83 – 1.73 (m, 1H), 1.50 (d, *J* = 7.0 Hz, 3H), 1.35 – 1.22 (m, 3H), 1.22 – 1.10 (m, 1H), 0.84 (t, *J* = 7.2 Hz, 3H).

^{13}C{^{1}H} NMR (126 MHz, $CDCl_3$): δ 154.3, 130.4, 128.5, 121.0, 120.8, 109.1, 107.5, 49.6, 34.1, 29.0, 27.2, 22.5, 19.0, 14.1.

HRMS (APCI): Exact Mass calculated for $[C_{14}H_{21}N_2O + H]^+$ requires *m/z* = 233.1648. Found 233.1647.

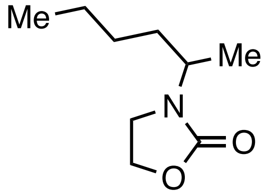

25

3-(hexan-2-yl)oxazolidin-2-one (25): The reaction was set up following general procedure B1 using oxazolidin-2-one (43.5 mg, 0.500 mmol, 1.00 equiv.), 1-hexene (188 μL, 1.50 mmol, 3.00 equiv.), and tris(4-methoxyphenyl)phosphine (35.2 mg, 0.100 mmol, 0.200 equiv.). The crude product was purified using silica gel flash column chromatography eluting with EtOAc/Hex (0% → 100%, eluted 50%) to give the title compound as a yellow oil.

Run 1: 47 mg (55% yield) **Run 2:** 36 mg (42% yield) **Average:** 49% yield

IR (FT-ATR, cm^{-1}, neat): 2957, 2928, 2859, 1735, 1483, 1457, 1420, 1384, 1253, 1163, 1122, 1092, 1057, 1037, 986, 967, 844, 762, 731, 693.

^{1}H NMR (500 MHz, $CDCl_3$): δ 4.32 (t, *J* = 8.0 Hz, 2H), 3.97 – 3.90 (m, 1H), 3.49 (q, *J* = 8.3 Hz, 1H)*, 3.43 (q, *J* = 8.0 Hz, 1H)*, 1.53 – 1.44 (m, 2H), 1.35 – 1.24 (m, 4H), 1.15 (d, *J* = 6.7 Hz, 3H), 0.90 (t, *J* = 6.8 Hz, 3H).

*diastereotopic

^{13}C{^{1}H} NMR (126 MHz, $CDCl_3$): δ 158.2, 62.0, 49.1, 39.7, 33.9, 28.7, 22.6, 18.2, 14.1.

HRMS (APCI): Exact Mass calculated for $[C_9H_{17}NO_2 + H]^+$ requires *m/z* = 172.1332. Found 172.1329.

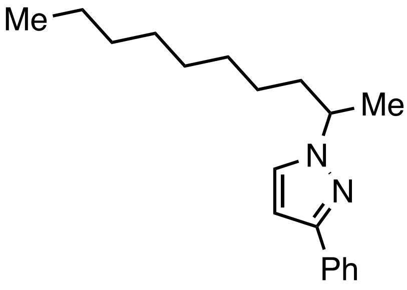

26

1-(decan-2-yl)-3-phenyl-1*H*-pyrazole (26): The reaction was set up following general procedure B1 using 3-phenylpyrazole (72.1 mg, 0.500 mmol, 1.00 equiv.), 1-decene (285 µL, 1.50 mmol, 3.00 equiv.), and triphenylphosphine (26.2 mg, 0.100 mmol, 0.200 equiv.). The crude product was purified using silica gel flash column chromatography eluting with Et_2O/Hex (0% → 10%, eluted 8%) to give the title compound as a yellow oil.

Run 1: 108 mg (76% yield) **Run 2:** 89 mg (63% yield) **Average:** 70% yield

IR (FT-ATR, cm^{-1}, neat): 3064, 3032, 2925, 2854, 1498, 1457, 1415, 1377, 1358, 1302, 1282, 1259, 1219, 1074, 1044, 1029, 982, 948, 912, 746, 692.

^{1}H NMR (500 MHz, $CDCl_3$): δ 7.84 – 7.78 (m, 2H), 7.43 – 7.34 (m, 3H), 7.32 – 7.24 (m, 1H), 6.53 (d, *J* = 2.3 Hz, 1H), 4.40 – 4.29 (m, 1H), 1.99 – 1.88 (m, 1H), 1.79 – 1.69 (m, 1H), 1.52 (d, *J* = 6.8 Hz, 3H), 1.34 – 1.21 (m, 12H), 0.86 (t, *J* = 7.0 Hz, 3H).

^{13}C{^{1}H} NMR (126 MHz, $CDCl_3$): δ 150.7, 134.1, 128.7, 128.1, 127.4, 125.7, 102.3, 58.7, 37.4, 32.0, 29.6, 29.45, 29.35, 26.3, 22.8, 21.5, 14.2.

HRMS (APCI): Exact Mass calculated for $[C_{19}H_{28}N_2 + H]^+$ requires *m/z* = 285.2325. Found 285.2324.

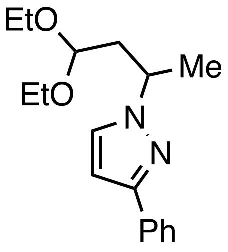

27

1-(4,4-diethoxybutan-2-yl)-3-phenyl-1*H*-pyrazole (27): The reaction was set up following general procedure B1 using 3-phenylpyrazole (72.1 mg, 0.500 mmol, 1.00 equiv.), 4,4-diethoxybut-1-ene (254 µL, 1.50 mmol, 3.00 equiv.), and triphenylphosphine (26.2 mg, 0.100 mmol, 0.200 equiv.). The crude product was purified using silica gel flash column chromatography eluting with EtOAc/Hex (0% → 15%, eluted 15%) to give the title compound as a yellow oil.

Run 1: 107 mg (74% yield) **Run 2:** 108 mg (75% yield) **Average:** 75% yield

IR (FT-ATR, cm^{-1}, neat): 3064, 3036, 2974, 2930, 2898, 2878, 1606, 1498, 1458, 1417, 1374, 1358, 1303, 1283, 1220, 1127, 1059, 1006, 990, 948, 749, 694.

^{1}H NMR (500 MHz, $CDCl_3$): δ 7.85 – 7.77 (m, 2H), 7.42 – 7.36 (m, 3H), 7.30 – 7.26 (m, 1H), 6.52 (d, *J* = 2.3 Hz, 1H), 4.56 – 4.45 (m, 1H), 4.20 (dd, *J* = 7.6, 4.0 Hz, 1H), 3.66 (dq, *J* = 9.4, 7.0 Hz, 1H), 3.55 (dq, *J* = 9.3, 7.1 Hz, 1H), 3.47 – 3.40 (m, 2H), 2.33 (ddd, *J* = 13.8, 9.6, 4.0 Hz, 1H), 2.04 (ddd, *J* = 14.1, 7.7, 4.9 Hz, 1H), 1.55 (d, *J* = 6.9 Hz, 3H), 1.22 (t, *J* = 7.0 Hz, 3H), 1.15 (t, *J* = 7.1 Hz, 3H).

^{13}C{^{1}H} NMR (126 MHz, $CDCl_3$): δ 151.2, 134.1, 129.1, 128.7, 127.5, 125.7, 102.2, 100.7, 62.4, 61.6, 55.1, 41.1, 21.9, 15.49, 15.47.

HRMS (APCI): Exact Mass calculated for $[C_{17}H_{24}N_2O_2 + H]^+$ requires m/z = 289.1911. Found 289.1911.

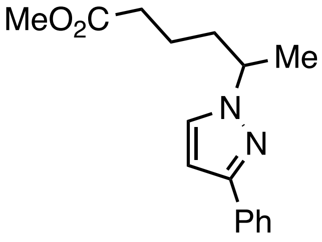

28

Methyl 5-(3-phenyl-1*H*-pyrazol-1-yl)hexanoate (28): The reaction was set up following general procedure B1 using 3-phenylpyrazole (72.1 mg, 0.500 mmol, 1.00 equiv.), methyl hex-5-enoate (211 µL, 1.50 mmol, 3.00 equiv.), and triphenylphosphine (26.2 mg, 0.100 mmol, 0.200 equiv.). The crude product was purified using silica gel flash column chromatography eluting with EtOAc/Hex (0% → 20%, eluted 20%) to give the title compound as a yellow oil.

Run 1: 109 mg (80% yield) **Run 2:** 100 mg (74% yield) **Average:** 77% yield

IR (FT-ATR, cm^{-1}, neat): 3032, 2974, 2950, 2870, 1734, 1606, 1498, 1457, 1436, 1416, 1358, 1256, 1217, 1167, 1109, 1074, 1045, 1016, 979, 948, 750, 695.

^{1}H NMR (500 MHz, $CDCl_3$): δ 7.83 – 7.77 (m, 2H), 7.41 (d, J = 2.4 Hz, 1H), 7.40 – 7.36 (m, 2H), 7.30 – 7.26 (m, 1H), 6.53 (d, J = 2.3 Hz, 1H), 4.39 – 4.32 (m, 1H), 3.65 (s, 3H), 2.30 (t, J = 7.4 Hz, 2H), 2.02 – 1.95 (m, 1H), 1.84 – 1.77 (m, 1H), 1.67 – 1.56 (m, 1H), 1.54 (d, J = 6.8 Hz, 3H), 1.53 – 1.45 (m, 1H).

^{13}C{^{1}H} NMR (126 MHz, $CDCl_3$): δ 173.9, 151.0, 134.0, 128.7, 128.4, 127.5, 125.7, 102.5, 58.3, 51.7, 36.6, 33.6, 21.7, 21.5.

HRMS (APCI): Exact Mass calculated for $[C_{16}H_{20}N_2O_2 + H]^+$ requires m/z = 273.1598. Found 273.1598.

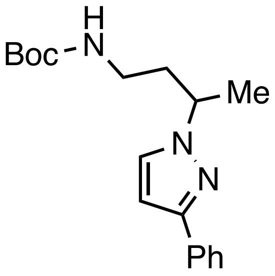

29

***tert*-butyl (3-(3-phenyl-1*H*-pyrazol-1-yl)butyl)carbamate (29):** The reaction was set up following general procedure B1 using 3-phenylpyrazole (72.1 mg, 0.500 mmol, 1.00 equiv.), *tert*-butyl but-3-en-1-ylcarbamate (276 µL, 1.50 mmol, 3.00 equiv.), and triphenylphosphine (26.2 mg, 0.100 mmol, 0.200 equiv.). The crude product was purified using silica gel flash column chromatography eluting with EtOAc/Hex (0% → 30%, eluted 30%) to give the title compound as an orange oil.

Run 1: 119 mg (76% yield) **Run 2:** 129 mg (82% yield) **Average:** 79% yield

IR (FT-ATR, cm^{-1}, neat): 3423, 3345, 3062, 3034, 2975, 2933, 1697, 1606, 1508, 1499, 1457, 1415, 1391, 1364, 1272, 1249, 1219, 1170, 1074, 1044, 1016, 948, 751, 694.

^{1}H NMR (500 MHz, $CDCl_3$): δ 7.80 (d, J = 7.3 Hz, 2H), 7.44 (d, J = 2.3 Hz, 1H), 7.39 (t, J = 7.7 Hz, 2H), 7.29 (t, J = 7.4 Hz, 1H), 6.55 (d, J = 2.3 Hz, 1H), 4.82 (br, 1H), 4.47 (m, 1H), 3.13 (m, 1H), 2.95 (m, 1H), 2.09 – 1.98 (m, 2H), 1.57 (d, J = 6.8 Hz, 3H), 1.42 (s, 9H).

^{13}C{^{1}H} NMR (126 MHz, $CDCl_3$): δ 156.1, 151.1, 133.8, 128.7, 128.5, 127.6, 125.7, 102.8, 79.3, 56.3, 37.7, 37.4, 28.5, 21.4.

HRMS (APCI): Exact Mass calculated for $[C_{18}H_{25}N_3O_2 + H]^+$ requires m/z = 316.2020. Found 316.2020.

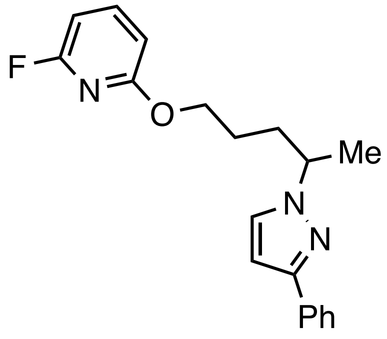

30

2-fluoro-6-((4-(3-phenyl-1*H*-pyrazol-1-yl)pentyl)oxy)pyridine (30): The reaction was set up following general procedure B1 using 3-phenylpyrazole (72.1 mg, 0.500 mmol, 1.00 equiv.), 2-fluoro-6-(pent-4-en-1-yloxy)pyridine (272 µL, 1.50 mmol, 3.00 equiv.), and triphenylphosphine (26.2 mg, 0.100 mmol, 0.200 equiv.). The crude product was purified using silica gel flash column chromatography eluting with Et_2O/Hex (0% → 15%, eluted 8%) to give the title compound as a yellow oil.
Run 1: 144 mg (88% yield) **Run 2:** 110 mg (67% yield) **Average:** 78% yield
IR (FT-ATR, cm^{-1}, neat): 2974, 2954, 2930, 2851, 1606, 1573, 1498, 1472, 1452, 1436, 1416, 1377, 1319, 1273, 1228, 1070, 1045, 1014, 993, 979, 947, 863, 788.
^{1}H NMR (500 MHz, $CDCl_3$): δ 7.81 – 7.79 (m, 2H), 7.63 – 7.58 (m, 1H), 7.43 (d, *J* = 2.3 Hz, 1H), 7.40 – 7.36 (m, 2H), 7.30 – 7.26 (m, 1H), 6.56 – 6.54 (m, 1H), 6.53 (d, *J* = 2.3 Hz, 1H), 6.44 – 6.42 (m, 1H), 4.46 – 4.39 (m, 1H), 4.25 – 4.22 (m, 2H), 2.14 – 2.06 (m, 1H), 1.97 – 1.90 (m, 1H), 1.77 – 1.69 (m, 1H), 1.68 – 1.61 (m, 1H), 1.56 (d, *J* = 6.8 Hz, 3H).
$^{13}C\{^1H\}$ NMR (126 MHz, $CDCl_3$): δ 163.1 (d, *J* = 13.8 Hz), 162.4 (d, *J* = 240.3 Hz), 151.0, 142.7 (d, *J* = 8.1 Hz), 134.0, 128.7, 128.4, 127.5, 125.8, 107.2 (d, *J* = 5.1 Hz), 102.5, 100.0 (d, *J* = 35.6 Hz), 66.2, 58.3, 33.8, 25.8, 21.6.
$^{19}F\{^1H\}$ NMR (282 MHz, $CDCl_3$): δ -70.18.
HRMS (APCI): Exact Mass calculated for $[C_{19}H_{20}N_3OF + H]^+$ requires *m/z* = 326.1663. Found 326.1663.

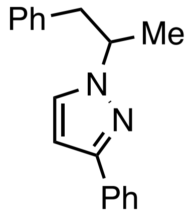

31

3-phenyl-1-(1-phenylpropan-2-yl)-1*H*-pyrazole (31): The reaction was set up following general procedure B1 using 3-phenylpyrazole (72.1 mg, 0.500 mmol, 1.00 equiv.), allylbenzene (199 µL, 1.50 mmol, 3.00 equiv.), and triphenylphosphine (26.2 mg, 0.100 mmol, 0.200 equiv.). The crude product was purified using silica gel flash column chromatography eluting with Et_2O/Hex (0% → 20%, eluted 15%) to give the title compound as a yellow oil.
Run 1: 79 mg (60% yield) **Run 2:** 76 mg (58% yield) **Average:** 59% yield
IR (FT-ATR, cm^{-1}, neat): 3062, 3029, 2976, 2933, 1605, 1497, 1457, 1415, 1376, 1357, 1283, 1265, 1221, 1074, 1047, 1030, 982, 948, 747, 695.
^{1}H NMR (500 MHz, $CDCl_3$): δ 7.88 – 7.81 (m, 2H), 7.44 – 7.38 (m, 2H), 7.33 – 7.28 (m, 1H), 7.25 – 7.19 (m, 3H), 7.18 (d, *J* = 2.3 Hz, 1H), 7.04 – 6.97 (m, 2H), 6.45 (d, *J* = 2.3 Hz, 1H), 4.57 (h, *J* = 6.8 Hz, 1H), 3.30 (dd, *J* = 13.5, 7.3 Hz, 1H), 3.05 (dd, *J* = 13.6, 6.6 Hz, 1H), 1.57 (d, *J* = 6.8 Hz, 3H).
$^{13}C\{^1H\}$ NMR (126 MHz, $CDCl_3$): δ 151.2, 138.1, 134.1, 129.4, 129.2, 128.7, 128.5, 127.5, 126.6, 125.8, 102.0, 59.8, 43.7, 20.5.
HRMS (APCI): Exact Mass calculated for $[C_{18}H_{18}N_2 + H]^+$ requires *m/z* = 263.1543. Found 263.1543.

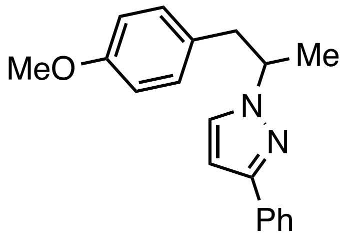

32

1-(1-(4-methoxyphenyl)propan-2-yl)-3-phenyl-1*H*-pyrazole (32): The reaction was set up following general procedure B1 using 3-phenylpyrazole (72.1 mg, 0.500 mmol, 1.00 equiv.), 1-allyl-4-methoxybenzene (235 µL, 1.50 mmol, 3.00 equiv.), and triphenylphosphine (26.2 mg, 0.100 mmol, 0.200 equiv.). The crude product was purified using silica gel flash column chromatography eluting with EtOAc/Hex (0% → 15%, eluted 15%) to give the title compound as a yellow oil.

Run 1: 103 mg (71% yield) **Run 2:** 98 mg (67% yield) **Average:** 69% yield

IR (FT-ATR, cm^{-1}, neat): 3063, 3031, 2976, 2950, 2933, 2835, 1611, 1583, 1512, 1498, 1458, 1416, 1375, 1357, 1301, 1246, 1221, 1178, 1115, 1074, 1033, 983, 948, 816, 749, 695.

^{1}H NMR (500 MHz, $CDCl_3$): δ 7.87 – 7.81 (m, 2H), 7.46 – 7.37 (m, 2H), 7.33 – 7.26 (m, 1H), 7.17 (d, *J* = 2.3 Hz, 1H), 6.96 – 6.86 (m, 2H), 6.80 – 6.72 (m, 2H), 6.45 (d, *J* = 2.3 Hz, 1H), 4.50 (h, *J* = 6.8 Hz, 1H), 3.76 (s, 3H), 3.22 (dd, *J* = 13.7, 7.3 Hz, 1H), 2.99 (dd, *J* = 13.7, 6.4 Hz, 1H), 1.55 (d, *J* = 6.8 Hz, 3H).

^{13}C{^{1}H} NMR (126 MHz, $CDCl_3$): δ 158.4, 151.2, 134.2, 130.3, 130.1, 129.2, 128.7, 127.5, 125.8, 113.9, 102.0, 60.0, 55.3, 42.8, 20.4.

HRMS (APCI): Exact Mass calculated for $[C_{19}H_{20}N_2O + H]^+$ requires *m/z* = 293.1648. Found 293.1648.

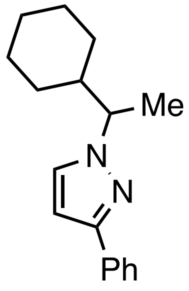

33

1-(1-cyclohexylethyl)-3-phenyl-1*H*-pyrazole (33): The reaction was set up following general procedure B1 using 3-phenylpyrazole (72.1 mg, 0.500 mmol, 1.00 equiv.), vinylcyclohexane (205 µL, 1.50 mmol, 3.00 equiv.), and triphenylphosphine (26.2 mg, 0.100 mmol, 0.200 equiv.). The crude product was purified using silica gel flash column chromatography eluting with Et_2O/Hex (0% → 10%, eluted 10%) to give the title compound as a yellow oil.

Run 1: 110 mg (87% yield) **Run 2:** 117 mg (92% yield) **Average:** 90% yield

IR (FT-ATR, cm^{-1}, neat): 3063, 3032, 2978, 2922, 2851, 1605, 1497, 1457, 1449, 1413, 1375, 1358, 1303, 1282, 1256, 1074, 1044, 992, 947, 912, 892, 746, 692.

^{1}H NMR (500 MHz, $CDCl_3$): δ 7.71 – 7.65 (m, 2H), 7.28 – 7.22 (m, 3H), 7.17 – 7.11 (m, 1H), 6.39 (d, *J* = 2.3 Hz, 1H), 3.97 – 3.91 (m, 1H), 1.70 – 1.58 (m, 3H), 1.57 – 1.47 (m, 2H), 1.38 (d, *J* = 6.9 Hz, 3H), 1.24 – 0.73 (m, 6H).

^{13}C{^{1}H} NMR (126 MHz, $CDCl_3$): δ 150.5, 134.2, 128.9, 128.7, 127.4, 125.7, 102.0, 63.5, 44.1, 29.9, 29.6, 26.4, 26.2, 26.1, 18.2.

HRMS (APCI): Exact Mass calculated for $[C_{17}H_{22}N_2 + H]^+$ requires *m/z* = 255.1856. Found 255.1856.

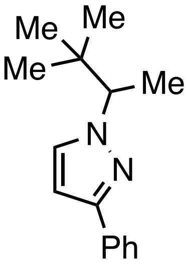
34

1-(3,3-dimethylbutan-2-yl)-3-phenyl-1*H*-pyrazole (34): The reaction was set up following general procedure B1 using 3-phenylpyrazole (72.1 mg, 0.500 mmol, 1.00 equiv.), 3,3-dimethylbut-1-ene (184 µL, 1.50 mmol, 3.00 equiv.), and triphenylphosphine (26.2 mg, 0.100 mmol, 0.200 equiv.). The crude product was purified using silica gel flash column chromatography eluting with Et_2O/Hex (0% → 10%, eluted 10%) to give the title compound as a pale yellow solid.

Run 1: 105 mg (92% yield) **Run 2:** 98 mg (86% yield) **Average:** 89% yield

IR (FT-ATR, cm^{-1}, neat): 3063, 2961, 2906, 2869, 1605, 1498, 1477, 1457, 1415, 1396, 1338, 1324, 1255, 1213, 1193, 1095, 1072, 1048, 993, 971, 947, 746, 692.

^{1}H NMR (500 MHz, $CDCl_3$): δ 7.84 – 7.78 (m, 2H), 7.42 – 7.34 (m, 3H), 7.29 – 7.25 (m, 1H), 6.52 (d, *J* = 2.3 Hz, 1H), 4.15 (q, *J* = 7.1 Hz, 1H), 1.53 (d, *J* = 7.1 Hz, 3H), 0.96 (s, 9H).

^{13}C{^{1}H} NMR (126 MHz, $CDCl_3$): δ 149.9, 134.2, 129.8, 128.7, 127.4, 125.7, 101.8, 67.0, 35.6, 27.0, 15.8.

HRMS (APCI): Exact Mass calculated for $[C_{15}H_{20}N_2 + H]^+$ requires *m/z* = 229.1699. Found 229.1699.

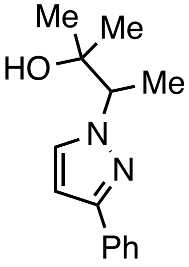
35

2-methyl-3-(3-phenyl-1*H*-pyrazol-1-yl)butan-2-ol (35): The reaction was set up following general procedure B1 using 3-phenylpyrazole (72.1 mg, 0.500 mmol, 1.00 equiv.), 2-methylbut-3-en-2-ol (149 µL, 1.50 mmol, 3.00 equiv.), and triphenylphosphine (26.2 mg, 0.100 mmol, 0.200 equiv.). The crude product was purified using silica gel flash column chromatography eluting with EtOAc/Hex (0% → 30%, eluted 30%) to give the title compound as a yellow oil.

Run 1: 103 mg (89% yield) **Run 2:** 104 mg (90% yield) **Average:** 90% yield

IR (FT-ATR, cm^{-1}, neat): 3413, 3133, 3065, 3036, 2977, 2932, 1606, 1525, 1498, 1459, 1413, 1374, 1359, 1344, 1305, 1247, 1221, 1176, 1155, 1105, 1074, 1049, 1028, 1013, 948, 915, 878, 753, 694.

^{1}H NMR (500 MHz, $CDCl_3$): δ 7.84 – 7.78 (m, 2H), 7.46 (d, *J* = 2.4 Hz, 1H), 7.42 – 7.37 (m, 2H), 7.34 – 7.28 (m, 1H), 6.57 (d, *J* = 2.4 Hz, 1H), 4.19 (q, *J* = 7.0 Hz, 1H), 1.60 (d, *J* = 6.9 Hz, 3H), 1.19 (s, 3H), 1.06 (s, 3H).

^{13}C{^{1}H} NMR (126 MHz, $CDCl_3$): δ 151.5, 133.1, 130.9, 128.8, 128.0, 125.8, 102.0, 72.8, 65.5, 28.0, 25.6, 15.7.

HRMS (APCI): Exact Mass calculated for $[C_{14}H_{18}N_2O + H]^+$ requires *m/z* = 231.1492. Found 231.1493.

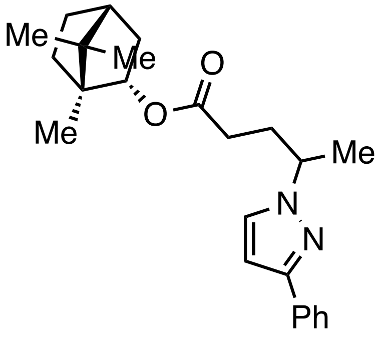

36

(1*R*,2*S*,4*R*)-1,7,7-trimethylbicyclo[2.2.1]heptan-2-yl 4-(3-phenyl-1*H*-pyrazol-1-yl)pentanoate (36): The reaction was set up following general procedure B1 using 3-phenylpyrazole (72.1 mg, 0.500 mmol, 1.00 equiv.), (1*R*,2*S*,4*R*)-1,7,7-trimethylbicyclo[2.2.1]heptan-2-yl pent-4-enoate (355 mg, 1.50 mmol, 3.00 equiv.), and triphenylphosphine (26.23 mg, 0.100 mmol, 0.200 equiv). The crude product was purified using silica gel flash column chromatography eluting with Et_2O/Hex (0% → 20%, eluted 20%) to give the title compound as a 1:1 mixture of diastereomers as a brown oil. D.r. determined by ^{1}H NMR integration of the singlets at 0.82 and 0.80 ppm assigned as the diastereomeric methyl groups.

Run 1: 170 mg (89% yield) **Run 2:** 170 mg (89% yield) **Average:** 89% yield

IR (FT-ATR, cm^{-1}, neat): 2953, 2873, 1726, 1498, 1457, 1385, 1375, 1357, 1334, 1303, 1265, 1240, 1217, 1177, 1160, 1114, 1074, 1044, 1027, 992, 980, 959, 915, 885, 736, 693.

^{1}H NMR (500 MHz, $CDCl_3$) δ 7.84 – 7.78 (m, 2H), 7.43 – 7.35 (m, 3H), 7.31 – 7.26 (m, 1H), 6.54 (d, *J* = 2.3 Hz, 1H), 4.90 – 4.85 (m, 1H), 4.48 – 4.38 (m, 1H), 2.38 – 2.30 (m, 1H), 2.29 – 2.20 (m, 3H), 2.19 – 2.10 (m, 1H), 1.91 – 1.85 (m, 1H), 1.76 – 1.69 (m, 1H), 1.66 (q, *J* = 4.7 Hz, 1H), 1.57 (d, *J* = 6.7 Hz, 3H), 1.30 – 1.18 (m, 2H), 0.96 – 0.91 (m, 1H), 0.90 (s, 3H), 0.86 (s, 3H), [diastereomeric: 0.82 (s, 1.5H*), 0.80 (s, 1.5H*)].

*diastereomeric CH_3 groups

^{13}C NMR (126 MHz, $CDCl_3$) δ 173.5, 151.2, 151.2, 133.9, 133.9, 128.8, 128.8, 128.7, 127.6, 125.8, 102.5, 102.5, 80.2, 80.2, 57.7, 57.6, 48.9, 48.9, 48.0, 47.9, 45.0, 37.0, 36.9, 32.4, 32.3, 31.2, 31.1, 28.2, 28.1, 27.2, 21.5, 21.5, 19.8, 19.0, 13.7, 13.7.

Carbon signals reported for the mixture of diastereomers.

HRMS: Exact Mass calculated for $[C_{24}H_{32}N_2O_2 + H]^+$ requires *m/z* = 381.2537. Found 381.2537 (APCI).

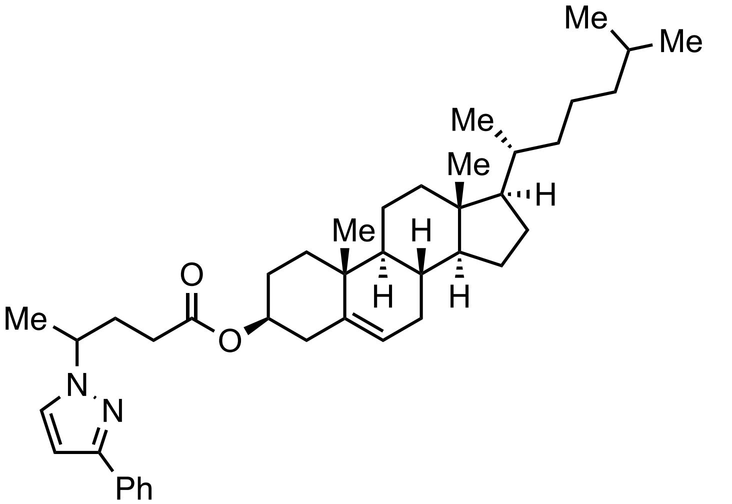

37

(1*R*,2*S*,4*R*)-1,7,7-trimethylbicyclo[2.2.1]heptan-2-yl 4-(3-phenyl-1*H*-pyrazol-1-yl)pentanoate (37): The reaction was set up following general procedure B1 using 3-phenylpyrazole (72.1 mg, 0.500 mmol, 1.00 equiv.), (3*S*,8*S*,9*S*,10*R*,13*R*,14*S*,17*R*)-10,13-dimethyl-17-(®-6-methylheptan-2-yl)-2,3,4,7,8,9,10,11,12,13,14,15,16,17-tetradecahydro-1*H*-cyclopenta[*a*]37henanthrene-3-yl pent-4-enoate (703 mg, 1.50 mmol, 3.00 equiv.), and triphenylphosphine (26.23 mg, 0.100 mmol, 0.200 equiv). The crude product was purified using silica gel flash column chromatography eluting with Et_2O/Hex (0% → 20%, eluted 20%) to give the title compound as a 1:1 mixture of diastereomers as a bubbly pink solid. D.r. determined by ^{1}H NMR integration of the doublets at 0.88 and 0.87 ppm assigned as the diastereomeric methyl groups.

Run 1: 253 mg (83% yield) **Run 2:** 223 mg (73% yield) **Average:** 78% yield

IR (FT-ATR, cm^{-1}, neat): 2933, 2866, 1728, 1498, 1457, 1416, 1375, 1357, 1333, 1301, 1265, 1218, 1171, 1120, 1074, 1043, 1028, 996, 984, 959, 948, 837, 800, 738, 692.

^{1}H NMR (500 MHz, $CDCl_3$) δ 7.84 – 7.78 (m, 2H), 7.42 – 7.36 (m, 3H), 7.30 – 7.26 (m, 1H), 6.54 (d, *J* = 2.3 Hz, 1H), 5.40 – 5.32 (m, 1H), 4.64 – 4.54 (m, 1H), 4.47 – 4.37 (m, 1H), 2.34 – 2.07 (m, 6H), 2.04 – 1.92 (m, 2H), 1.87 – 1.80 (m, 3H), 1.63 – 1.57 (m, 1H), 1.56 (d, *J* = 6.8 Hz, 3H), 1.55 – 1.42 (m, 5H), 1.41 – 1.20 (m, 5H), 1.19 – 1.04 (m, 7H), 1.04 – 1.01 (m, 1H), 1.00 (s, 3H), 1.00 – 0.93 (m, 2H), 0.92 (d, *J* = 6.5 Hz, 3H), [diastereomeric: 0.88 (d, *J* = 6.6 Hz, 1.5H*), 0.87 (d, *J* = 6.6 Hz, 1.5H*)], 0.68 (s, 3H).

*diastereomeric CH_3 groups

^{13}C NMR (126 MHz, $CDCl_3$) δ 172.5, 151.1, 139.7, 139.7, 134.0, 128.8, 128.7, 127.5, 125.7, 122.8, 102.5, 74.2, 57.6, 56.8, 56.3, 50.1, 42.4, 39.8, 39.6, 38.2, 38.2, 37.1, 36.7, 36.3, 35.9, 32.3, 32.0, 32.0, 31.2, 28.4, 28.1, 27.9, 27.9, 24.4, 24.0, 23.0, 22.7, 21.4, 21.1, 19.4, 18.8, 12.0. Carbon signals reported for the mixture of diastereomers.

HRMS: Exact Mass calculated for $[C_{41}H_{60}N_2O_2 + H]^+$ requires *m/z* = 613.4728. Found 613.4733 (APCI).

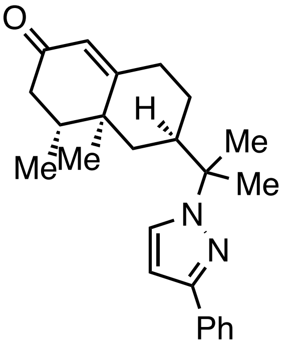

38

(4*R*,4a*S*,6*R*)-4,4a-dimethyl-6-(2-(3-phenyl-1*H*-pyrazol-1-yl)propan-2-yl)-4,4a,5,6,7,8-hexahydronaphthalen-2(3*H*)-one (38): The reaction was set up following general procedure B1 using 3-phenylpyrazole (72.1 mg, 0.500 mmol, 1.00 equiv.), (4*R*,4a*S*,6*R*)-4,4a-dimethyl-6-(prop-1-en-2-yl)-4,4a,5,6,7,8-hexahydronaphthalen-2(3*H*)-one (328 mg, 1.500 mmol, 3.00 equiv.), and triphenylphosphine (26.23 mg, 0.100 mmol, 0.200 equiv.). The crude product was purified using silica gel flash column chromatography eluting with Et_2O/Hex (0% → 50%, eluted 50%) to give the title compound as a Bubbly white solid.

Run 1: 50 mg (28% yield) **Run 2:** 44 mg (24% yield) **Average:** 26% yield

IR (FT-ATR, cm^{-1}, neat): 3062, 3029, 2969, 2941, 2897, 2879, 1663, 1618, 1496, 1457, 1433, 1415, 1378, 1362, 1314, 1301, 1286, 1227, 1211, 1201, 1182, 1104, 1074, 1049, 986, 948, 911, 873, 846, 774, 749, 693.

^{1}H NMR (500 MHz, CDCl$_3$) δ 7.85 – 7.79 (m, 2H), 7.50 (d, *J* = 2.4 Hz, 1H), 7.43 – 7.34 (m, 2H), 7.31 – 7.27 (m, 1H), 6.52 (d, *J* = 2.3 Hz, 1H), 5.73 (s, 1H), 2.58 – 2.41 (m, 2H), 2.33 (ddd, *J* = 15.2, 4.1, 2.6 Hz, 1H), 2.28 – 2.15 (m, 2H), 1.97 – 1.89 (m, 1H), 1.63 – 1.61 (m, 1H), 1.59 (d, *J* = 7.4 Hz, 6H), 1.51 (dt, *J* = 12.8, 2.8 Hz, 1H), 1.23 – 1.14 (m, 1H), 1.04 (s, 3H), 0.92 (t, *J* = 12.8 Hz, 1H), 0.84 (d, *J* = 6.8 Hz, 3H).
^{13}C NMR (126 MHz, CDCl$_3$) δ 199.7, 170.4, 150.9, 134.3, 128.7, 127.9, 127.5, 125.7, 124.7, 101.7, 63.3, 43.2, 42.1, 40.6, 39.6, 39.3, 33.1, 27.8, 25.3, 24.1, 16.9, 15.1.
HRMS: Exact Mass calculated for $[C_{24}H_{30}N_2O + H]^+$ requires *m/z* = 363.2431. Found 363.2430 (APCI).

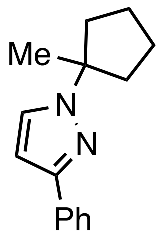

39

1-(1-methylcyclopentyl)-3-phenyl-1*H*-pyrazole (39): The reaction was set up following general procedure B1 using 3-phenylpyrazole (72.1 mg, 0.500 mmol, 1.00 equiv.), methylenecyclopentane (158 µL, 1.500 mmol, 3.00 equiv.), and tris(4-methoxyphenyl)phosphine (35.2 mg, 0.100 mmol, 0.200 equiv.). The crude product was purified using silica gel flash column chromatography eluting with Et_2O/Hex (0% → 10%, eluted 8%) to give the title compound as a colorless oil.
Run 1: 15 mg (13% yield) **Run 2:** 29 mg (26% yield) **Average:** 20% yield
IR (FT-ATR, cm^{-1}, neat): 2963, 2930, 2872, 1606, 1497, 1457, 1375, 1361, 1232, 1174, 1074, 1051, 983, 948, 773, 746, 693.
^{1}H NMR (500 MHz, CDCl$_3$): δ 7.86 – 7.79 (m, 2H), 7.52 (d, *J* = 2.3 Hz, 1H), 7.41 – 7.35 (m, 2H), 7.31 – 7.26 (m, 1H), 6.52 (d, *J* = 2.4 Hz, 1H), 2.51 – 2.40 (m, 2H), 1.94 – 1.87 (m, 2H), 1.80 (m, 4H), 1.62 (s, 3H).
^{13}C NMR (126 MHz, CDCl$_3$) δ 150.6, 134.3, 128.6, 127.6, 127.3, 125.7, 102.1, 69.3, 39.7, 28.0, 23.8.
HRMS: Exact Mass calculated for $[C_{15}H_{19}N_2 + H]^+$ requires *m/z* = 227.1543. Found 227.1536 (ESI+).

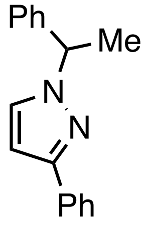

40

3-phenyl-1-(1-phenylethyl)-1*H*-pyrazole (40): The reaction was set up following general procedure B1 using 3-phenylpyrazole (72.1 mg, 0.500 mmol, 1.00 equiv.), styrene (69.0 µL, 0.600 mmol, 1.20 equiv.), and tris(4-methoxyphenyl)phosphine (35.2 mg, 0.100 mmol, 0.200 equiv.). The crude product was purified using silica gel flash column chromatography eluting with Et_2O/Hex (0% → 30%, eluted 30%), followed by reverse-phase C18 silica gel column chromatography eluting MeCN/H_2O (50% → 100%, eluted 70%) to give the title compound as a yellow oil.
Run 1: 77 mg (62% yield) **Run 2:** 84 mg (68% yield) **Average:** 65% yield
IR (FT-ATR, cm^{-1}, neat): 3060, 3029, 2979, 2933, 1604, 1496, 1456, 1405, 1375, 1356, 1326, 1301, 1280, 1250, 1216, 1096, 1074, 1043, 1029, 1005, 966, 946, 913, 747, 692.
^{1}H NMR (500 MHz, CDCl$_3$): δ 7.86 – 7.80 (m, 2H), 7.43 – 7.36 (m, 3H), 7.36 – 7.32 (m, 2H), 7.31 – 7.26 (m, 3H), 7.25 – 7.24 (m, 1H), 6.57 (d, *J* = 2.3 Hz, 1H), 5.60 (q, *J* = 7.1 Hz, 1H), 1.94 (d, *J* = 7.1 Hz, 3H).

$^{13}C\{^{1}H\}$ **NMR (126 MHz, $CDCl_3$):** δ 151.1, 142.1, 133.9, 129.1, 128.8, 128.7, 127.9, 127.6, 126.6, 125.8, 102.9, 61.4, 21.8.
HRMS (APCI): Exact Mass calculated for $[C_{17}H_{16}N_2 + H]^+$ requires *m/z* = 249.1386. Found 249.1386.

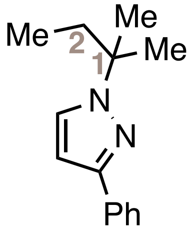

41

1-(*tert*-pentyl)-3-phenyl-1*H*-pyrazole (41): The reaction was set up following general procedure A using 3-phenylpyrazole (14.1 mg, 0.100 mmol, 1.00 equiv.), 2-methylbut-2-ene (31.8 µL, 0.300 mmol, 3.00 equiv.), and tris(4-methoxyphenyl)phosphine (7.05 mg, 0.100 mmol, 0.200 equiv.). After addition of 1,3,5-trimethoxybenzene (1 equiv.) as internal standard, the crude product was purified using preparative TLC eluting with 8% EtOAc/Hex to give the title compound as a colorless oil.

^{1}**H NMR yield:** 14% combined yield (*r.r.* 1 to 1.6 *C1:C2*)

C1 isomer:
IR (FT-ATR, cm^{-1}, neat): 2971, 2925, 2853, 1496, 1457, 1379, 1362, 1307, 1221, 1102, 1074, 1046, 1028, 949, 905, 812, 727, 693.
^{1}**H NMR (500 MHz, $CDCl_3$):** δ 7.87 – 7.77 (m, 2H), 7.50 (d, *J* = 2.4 Hz, 1H), 7.44 – 7.32 (m, 2H), 7.32 – 7.27 (m, 1H), 6.51 (d, *J* = 2.4 Hz, 1H), 1.94 (q, *J* = 7.4 Hz, 2H), 1.60 (s, 6H), 0.73 (t, *J* = 7.4 Hz, 3H).
^{13}**C NMR (126 MHz, $CDCl_3$)** δ 150.6, 134.4, 128.6, 127.7, 127.3, 125.7, 101.7, 61.4, 35.6, 27.3, 8.5.
HRMS: Exact Mass calculated for $[C_{14}H_{18}N_2 + H]^+$ requires *m/z* = 215.1543. Found 215.1541 (ESI+).

C2 isomer:
IR (FT-ATR, cm^{-1}, neat): 2963, 2926, 1497, 1457, 1378, 1357, 1243, 1215, 1102, 1090, 1073, 1046, 988, 948, 904, 725, 694.
^{1}**H NMR (500 MHz, $CDCl_3$):** δ 7.84 – 7.77 (m, 2H), 7.40 – 7.36 (m, 3H), 7.29 – 7.26 (m, 1H), 6.52 (d, *J* = 2.3 Hz, 1H), 4.11 – 4.02 (m, 1H), 2.15 (m, 1H), 1.51 (s, 3H), 0.96 (d, *J* = 6.7 Hz, 3H), 0.80 (d, *J* = 6.7 Hz, 3H).
^{13}**C NMR (126 MHz, $CDCl_3$)** δ 150.6, 128.8, 128.7, 127.4, 125.7, 102.1, 64.3, 34.6, 19.6, 19.1, 18.0.
HRMS: Exact Mass calculated for $[C_{14}H_{18}N_2 + H]^+$ requires *m/z* = 215.1543. Found 215.1544 (ESI+).

3-phenyl-1-(1-phenylpropyl)-1*H*-pyrazole (42): The reaction was set up following general procedure B1 using 3-phenylpyrazole (72.1 mg, 0.500 mmol, 1.00 equiv.), prop-1-en-1-ylbenzene (195 µL, 1.500 mmol, 3.00 equiv.), and tris(4-methoxyphenyl)phosphine (35.2 mg, 0.100 mmol, 0.200 equiv.). The crude product was purified using silica gel flash column chromatography eluting with EtOAc/Hex (0% → 10%, eluted 10%) to give the title compound as a colorless oil.

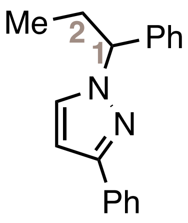

42

Run 1: 17 mg (13% yield) **Run 2:** 17 mg (13% yield) **Average:** 13% yield

IR (FT-ATR, cm^{-1}, neat): 3062, 3031, 2966, 2932, 2876, 1497, 1456, 1409, 1356, 1301, 1228, 1216, 1072, 1046, 1028, 906, 838, 727, 692.

^{1}H NMR (500 MHz, $CDCl_3$): δ 7.87 – 7.81 (m, 2H), 7.42 (d, *J* = 2.3 Hz, 1H), 7.41 – 7.37 (m, 2H), 7.35 – 7.33 (m, 4H), 7.31 – 7.26 (m, 2H), 6.56 (d, *J* = 2.3 Hz, 1H), 5.23 (dd, *J* = 8.8, 6.7 Hz, 1H), 2.54 – 2.45 (m, 1H), 2.28 – 2.19 (m, 1H), 0.96 (t, *J* = 7.3 Hz, 3H).

^{13}C NMR (126 MHz, $CDCl_3$) δ 151.1, 140.9, 134.0, 129.5, 128.7, 128.7, 127.9, 127.5, 127.1, 125.8, 102.8, 68.2, 28.9, 11.4.

HRMS: Exact Mass calculated for $[C_{18}H_{18}N_2 + H]^+$ requires *m/z* = 263.1543. Found 263.1541 (ESI+).

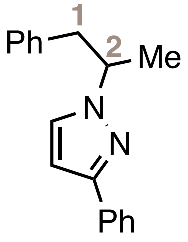

31

3-phenyl-1-(1-phenylpropan-2-yl)-1*H*-pyrazole (31): The reaction was set up following general procedure A using 3-phenylpyrazole (14.1 mg, 0.100 mmol, 1.00 equiv.), prop-1-en-1-ylbenzene (38.9 µL, 0.300 mmol, 3.00 equiv.), and tricyclohexylphosphine (2.80 mg, 0.100 mmol, 0.100 equiv.). After addition of 1,3,5-trimethoxybenzene (1 equiv.) as internal standard, the crude product was purified using preparative TLC eluting with 10% EtOAc/Hex to give the title compound as a colorless oil (co-eluted with 1,3,5-TMB). Characterization data aligned with that for compound **31**.

^{1}H NMR yield: 2% yield

5. Other Procedures and Characterization

Procedure C for reaction set-up using Schlenk technique (0.10 mmol scale): A 1.0-dram glass vial equipped with a stir bar was flame-dried and allowed to cool to room temperature under N_2. It was then charged with the N–H heteroaromatic substrate, phosphine (0.010 mmol, 0.2 equiv.), $[Ir(dF(Me)ppy)_2(dtbbpy)]PF_6$ (2.0 mg, 0.010 mmol, 0.020 equiv.), and TRIP-SH (2.3 µL, 0.010 mmol, 0.10 equiv.). The vial was capped with a septum cap and was evacuated and backfilled with N_2 three times. Sparged α,α,α-trifluorotoluene (1.0 mL, 0.1 M) and alkene (0.30 mmol, 3.0 equiv.) were added to the vial by syringe. The vial was sealed with electrical tape and placed in the photoreactor (450 nm, 100% intensity, 2800 rpm fan speed), and the reaction was stirred at

1500 rpm for 18 hours. At the end of 18 hours, the vial was removed from the photoreactor. 1,3,5-Trimethoxybenzene solid (~1 equiv.) was added to the vial as an internal standard for determining the yield by ^{1}H NMR; the exact amount of standard added was recorded to back-calculate the corresponding peak integration.

Procedure D for gram-scale reaction (5.0 mmol scale): A 50 mL tear drop shaped flask equipped with an egg-shaped stir bar was flame-dried and allowed to cool to room temperature under N_2, then charged with 3-phenylpyrazole (721 mg, 5.0 mmol, 1 equiv.). The flask was loosely covered with a white rubber septum and brought into a N_2-filled glovebox. Triphenylphosphine (262 mg, 1.00 mmol, 0.2 equiv.) and [Ir(dF(Me)ppy)$_2$(dtbbpy)]PF$_6$ (101 mg, 0.10 mmol, 0.020 equiv.) were added to the flask and suspended in 50 mL of toluene (0.1 M). The reaction flask was charged with TRIP-SH (118 µL, 0.50 mmol, 0.10 equiv.) and 1-hexene (1.88 mL, 15 mmol, 3.0 equiv.). The flask was capped with the rubber septum, sealed with electrical tape, and removed from the glovebox. The reaction was stirred at 800 rpm for 18 hours while irradiating with two Kessil PR160L 427 nm lamps at 100% intensity placed horizontally across from each other, 2 cm from the reaction flask. At the end of 18 hours, the flask was removed from the Kessil lamp setup. The reaction was then opened to air, and concentrated *in vacuo* to give the crude product which was purified by automated flash chromatography eluting with 8% diethyl ether in hexanes to provide the desired product as an orange oil (969 mg, 85% yield).

6. X-ray Crystal Structure

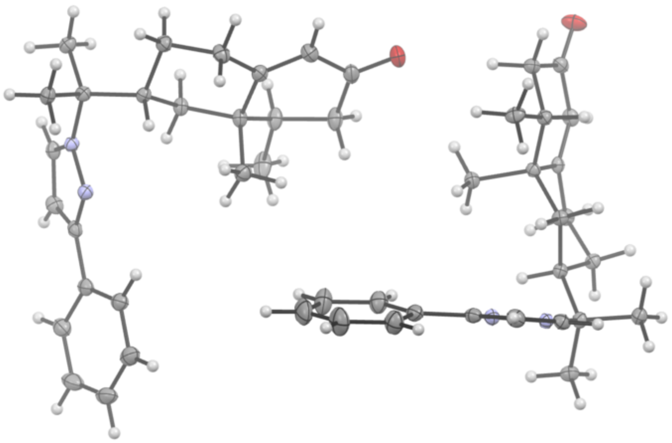

Experimental crystallographic data for (4*R*,4a*S*,6*R*)-4,4a-dimethyl-6-(2-(3-phenyl-1*H*-pyrazol-1-yl)propan-2-yl)-4,4a,5,6,7,8-hexahydronaphthalen-2(3*H*)-one (38) (CCDC Deposition # 2476334)

**Crystal data**	
Chemical formula	$C_{24}H_{30}N_2O$
M_r	362.50

Crystal system, space group	Monoclinic, $P2_1$
Temperature (K)	100
a, *b*, *c* (Å)	6.3358 (2), 20.5116 (8), 16.0478 (6)
β (°)	98.682 (2)
V (Å^3)	2061.63 (13)
Z	4
Radiation type	Cu $K\alpha$
μ (mm^{-1})	0.55
Crystal size (mm)	0.25 × 0.1 × 0.1

Data collection

Diffractometer	Bruker *APEX*-II CCD
Absorption correction	Multi-scan
T_{min}, T_{max}	0.691, 0.753
No. of measured, independent and observed [$I > 2\sigma(I)$] reflections	35480, 7309, 7218
R_{int}	0.027
$(\sin \theta/\lambda)_{max}$ (Å^{-1})	0.603

Refinement

$R[F^2 > 2\sigma(F^2)]$, $wR(F^2)$, S	0.027, 0.069, 1.05
No. of reflections	7309
No. of parameters	495
No. of restraints	1
H-atom treatment	H-atom parameters constrained
$\Delta\rho_{max}$, $\Delta\rho_{min}$ (e Å^{-3})	0.13, −0.24

Computer programs: SAINT V8.40B (2016), olex2.solve 1.5 (Bourhis et al., 2015), SHELXL 2019/2 (Sheldrick, 2015), Olex2 1.5 (Dolomanov et al., 2009).

7. Evaluation of Byproducts

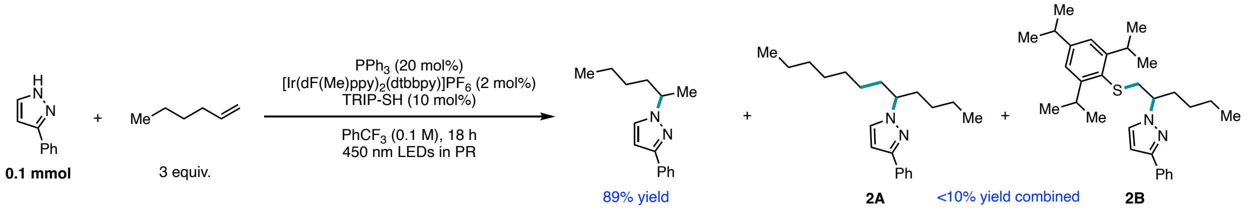

The reaction was set up following general procedure B1 using 3-phenylpyrazole (72.1 mg, 0.50 mmol, 1.0 equiv.), 1-hexene (188 µL, 1.50 mmol, 3.0 equiv.), and triphenylphosphine (26.2 mg, 0.10 mmol, 0.20 equiv.). The byproducts 1-(dodecan-5-yl)-3-phenyl-1*H*-pyrazole **2A** and 3-phenyl-1-(1-((2,4,6-triisopropylphenyl)thio)hexan-2-yl)-1*H*-pyrazole **2B** can be separated using reverse-phase C18 column chromatography eluting with 90% acetonitrile in water.

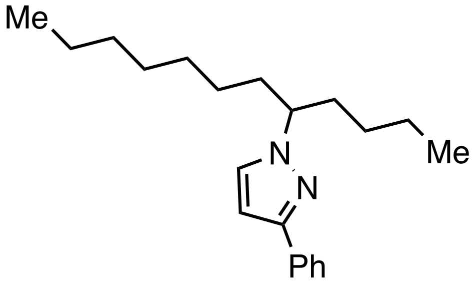

2A

1-(dodecan-5-yl)-3-phenyl-1*H*-pyrazole (2A):

IR (FT-ATR, cm^{-1}, neat): 2953, 2924, 2855, 1607, 1498, 1457, 1415, 1377, 1357, 1320, 1301, 1281, 1262, 1216, 1096, 1073, 1045, 1029, 948, 910, 744, 691.

^{1}H NMR (500 MHz, $CDCl_3$): δ 7.84 – 7.78 (m, 2H), 7.43 – 7.33 (m, 3H), 7.31 – 7.26 (m, 1H), 6.52 (d, *J* = 2.3 Hz, 1H), 4.11 (tt, *J* = 9.6, 4.9 Hz, 1H), 1.95 – 1.83 (m, 2H), 1.80 – 1.72 (m, 2H), 1.34 – 1.17 (m, 12H), 1.16 – 1.07 (m, 2H), 0.85 (t, *J* = 7.1 Hz, 6H).

^{13}C{^{1}H} NMR (126 MHz, $CDCl_3$): δ 150.7, 134.2, 129.1, 128.7, 127.4, 125.8, 102.1, 63.6, 35.8, 35.6, 31.9, 29.4, 29.3, 28.5, 26.3, 22.8, 22.6, 14.2, 14.1.

HRMS (APCI): Exact Mass calculated for $[C_{21}H_{32}N_2 + H]^+$ requires *m/z* = 313.2638. Found 313.2638.

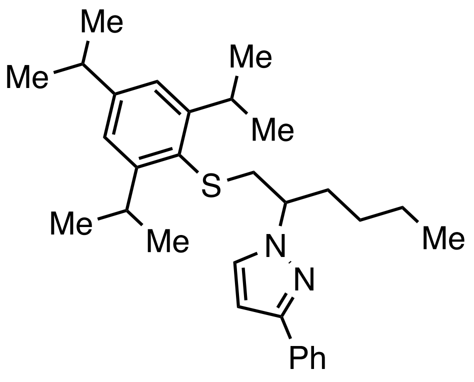

2B

3-phenyl-1-(1-((2,4,6-triisopropylphenyl)thio)hexan-2-yl)-1*H*-pyrazole (2B):

IR (FT-ATR, cm^{-1}, neat): 2959, 2927, 2866, 1598, 1560, 1526, 1498, 1458, 1419, 1382, 1360, 1309, 1218, 1167, 1156, 1100, 1074, 949, 939, 913, 877, 746, 693.

^{1}H NMR (500 MHz, $CDCl_3$): δ 7.84 – 7.79 (m, 2H), 7.43 (d, *J* = 2.3 Hz, 1H), 7.41 – 7.36 (m, 2H), 7.30 – 7.26 (m, 1H), 6.97 (s, 2H), 6.53 (d, *J* = 2.2 Hz, 1H), 4.28 – 4.22 (m, 1H), 3.76 (hept, *J* = 6.9 Hz, 2H), 3.23 (dd, *J* = 12.6, 7.9 Hz, 1H), 3.04 (dd, *J* = 12.5, 5.4 Hz, 1H), 2.85 (hept, *J* = 6.9 Hz, 1H), 2.08 – 2.00 (m, 1H), 1.98 – 1.87 (m, 1H), 1.33 – 1.26 (m, 2H), 1.23 (d, *J* = 6.9 Hz, 6H), 1.19 (d, *J* = 4.7 Hz, 6H), 1.17 (d, *J* = 4.8 Hz, 6H), 1.16 – 1.06 (m, 2H), 0.85 (t, *J* = 7.2 Hz, 3H).

^{13}C{^{1}H} NMR (126 MHz, $CDCl_3$): δ 153.1, 151.5, 149.8, 134.0, 130.4, 128.6, 128.3, 127.6, 125.8, 122.0, 102.1, 63.0, 43.4, 34.42, 34.37, 31.6, 28.5, 24.7, 24.6, 24.0, 22.4, 14.1.

HRMS (APCI): Exact Mass calculated for $[C_{30}H_{42}N_2S + H]^+$ requires *m/z* = 463.3141. Found 463.3144.

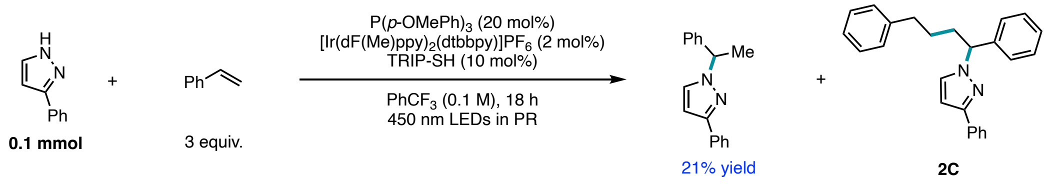

The reaction was set up following general Procedure A using 3-phenylpyrazole (14.1 mg, 0.10 mmol, 1.0 equiv.), styrene (34.4 µL, 0.60 mmol, 3.0 equiv.), and tris(4-methoxyphenyl)phosphine (7.1 mg, 0.020 mmol, 0.20 equiv.). The byproduct 1-(1,4-diphenylbutyl)-3-phenyl-1*H*-pyrazole **2C** can be separated using reverse-phase C18 column chromatography eluting with 75% acetonitrile in water.

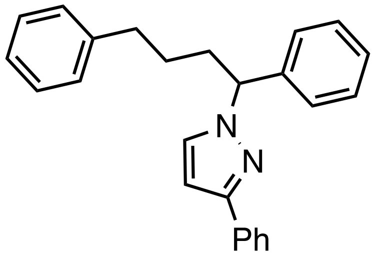

2C

1-(1,4-diphenylbutyl)-3-phenyl-1*H*-pyrazole (2C):

IR (FT-ATR, cm^{-1}, neat): 3060, 3026, 2926, 2857, 1603, 1496, 1453, 1405, 1355, 1303, 1281, 1241, 1215, 1179, 1156, 1099, 1074, 1046, 1029, 947, 912, 745, 692.

^{1}H NMR (500 MHz, CDCl$_3$): δ 7.86 – 7.80 (m, 2H), 7.42 – 7.35 (m, 3H), 7.35 – 7.26 (m, 6H), 7.25 (d, *J* = 1.7 Hz, 1H), 7.21 – 7.11 (m, 3H), 6.54 (d, *J* = 2.3 Hz, 1H), 5.33 (dd, *J* = 8.9, 6.6 Hz, 1H), 2.68 (t, *J* = 7.6 Hz, 2H), 2.55 – 2.48 (m, 1H), 2.26 – 2.18 (m, 1H), 1.70 – 1.62 (m, 2H).

^{13}C{^{1}H} NMR (126 MHz, CDCl$_3$): δ 151.1, 142.0, 140.9, 133.9, 129.5, 128.8, 128.7, 128.6, 128.5, 128.0, 127.6, 127.0, 126.0, 125.8, 102.9, 66.4, 35.6, 35.2, 28.3.

HRMS (APCI): Exact Mass calculated for $[C_{25}H_{24}N_2 + H]^+$ requires *m/z* = 353.2012. Found 353.2013.

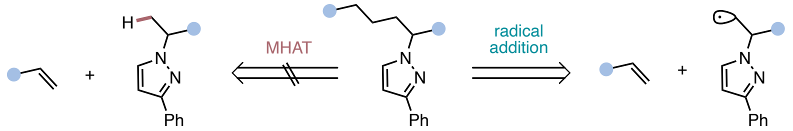

The structures of these byproducts are consistent with the proposed mechanism, as illustrated above to the right, wherein a terminal alkyl radical undergoes addition into alkene that is present in excess in the reaction. In contrast, the formation of these species is inconsistent with an MHAT mechanism, as shown above to the left, wherein the terminal carbon will first undergo HAT.

8. Synthesis of Alkenes

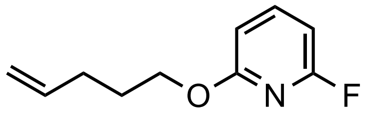

A1

2-fluoro-6-(pent-4-en-1-yloxy)pyridine (A1): Prepared according to literature procedure[3] from 6-fluoropyridin-2-ol and pent-4-en-1-ol (77% yield).

IR (FT-ATR, cm^{-1}, neat): 2942, 1618, 1607, 1571, 1474, 1452, 1437, 1379, 1320, 1273, 1228, 1142, 1070, 1018, 992, 977, 912, 787, 748, 727.

^{1}H NMR (500 MHz, $CDCl_3$): δ 7.63 (q, *J* = 8.1 Hz, 1H), 6.58 (dd, *J* = 8.0, 1.7 Hz, 1H), 6.44 (dd, *J* = 7.8, 2.5 Hz, 1H), 5.85 (ddt, *J* = 16.9, 10.2, 6.6 Hz, 1H), 5.06 (dq, *J* = 17.1, 1.7 Hz, 1H), 4.99 (dq, *J* = 10.1, 1.4 Hz, 1H), 4.27 (t, *J* = 6.6 Hz, 2H), 2.25 – 2.17 (m, 2H), 1.91 – 1.82 (m, 2H).

^{13}C{^{1}H} NMR (126 MHz, $CDCl_3$): δ 163.3, 162.3 (d, *J* = 226.5 Hz), 142.6 (d, *J* = 8.0 Hz), 137.9, 115.3, 107.3 (d, *J* = 5.1 Hz), 99.9 (d, *J* = 35.5 Hz), 66.1, 30.2, 28.2.

^{19}F{^{1}H} NMR (376 MHz, $CDCl_3$) δ -70.23.

HRMS (APCI): Exact Mass calculated for $[C_{10}H_{12}NOF + H]^+$ requires *m/z* = 182.0976. Found 182.0976.

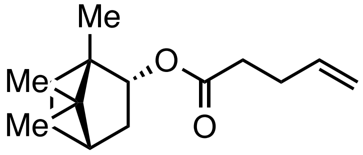

A2

(1*S*,2*R*,4*S*)-1,7,7-trimethylbicyclo[2.2.1]heptan-2-yl pent-4-enoate (A2): A flame-dried round-bottom flask equipped with a magnetic stir bar was charged with (1*S*,2*R*,4*S*)-1,7,7-trimethylbicyclo[2.2.1]heptan-2-ol (6.48 mmol, 1 equiv.), EDC (1.2 equiv.), DMAP (0.1 equiv.), pent-4-enoic acid (1.2 equiv.), and triethylamine (3 equiv.). Dichloromethane (20 mL) was added to the flask and the reaction allowed to stir overnight. The reaction was filtered and the filtrate was washed with 1M HCl, aq. Saturated $NaHCO_3$, then water. The organic layer was dried over Na_2SO_4 then concentrated to provide the desired product as a colorless oil (799 mg, 52% yield).

IR (FT-ATR, cm^{-1}, neat): 2878, 2952, 1731, 1641, 1473, 1452, 1419, 1353, 1301, 1254, 1233, 1176, 1158, 1140, 1113, 1025, 991, 980, 911, 824, 786, 746.

^{1}H NMR (500 MHz, $CDCl_3$): δ 5.89 – 5.78 (m, 1H), 5.07 (dq, *J* = 17.2, 1.6 Hz, 1H), 5.04 – 4.98 (m, 1H), 4.89 (ddd, *J* = 10.0, 3.6, 2.2 Hz, 1H), 2.46 – 2.36 (m, 4H), 2.37 – 2.31 (m, 1H), 1.94 (ddd, *J* = 12.7, 9.4, 4.5 Hz, 1H), 1.78 – 1.71 (m, 1H), 1.67 (t, *J* = 4.5 Hz, 1H), 1.33 – 1.27 (m, 1H), 1.26 – 1.20 (m, 1H), 0.96 (dd, *J* = 13.8, 3.5 Hz, 1H), 0.90 (s, 3H), 0.87 (s, 3H), 0.82 (s, 3H).

^{13}C{^{1}H} NMR (126 MHz, $CDCl_3$): δ 173.5, 137.0, 115.6, 80.0, 48.9, 47.9, 45.0, 37.0, 34.1, 29.2, 28.2, 27.3, 19.9, 19.0, 13.6.

HRMS (APCI): Exact Mass calculated for $[C_{15}H_{24}O_2 + H]^+$ requires *m/z* = 237.1849. Found 237.1837.

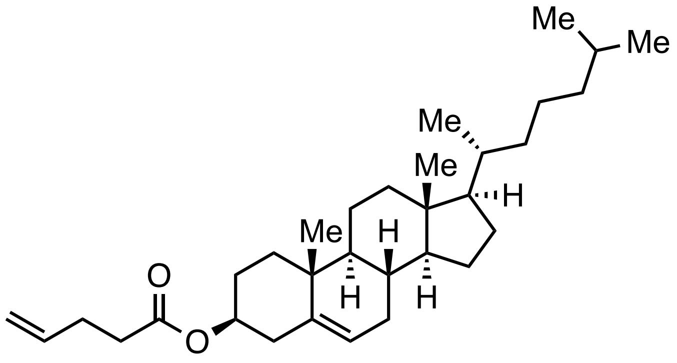

A3

(3*S*,8*S*,9*S*,10*R*,13*R*,14*S*,17*R*)-10,13-dimethyl-17-((*R*)-6-methylheptan-2-yl)-2,3,4,7,8,9,10,11,12,13,14,15,16,17-tetradecahydro-1*H*-cyclopenta[*a*]47henanthrene-3-yl pent-4-enoate (A3): A flame-dried round-bottom flask equipped with a magnetic stir bar was charged with (3*S*,8*S*,9*S*,10*R*,13*R*,14*S*,17*R*)-10,13-dimethyl-17-(®-6-methylheptan-2-yl)-2,3,4,7,8,9,10,11,12,13,14,15,16,17-tetradecahydro-1*H*-cyclopenta[*a*]47henanthrene-3-ol (6.48 mmol, 1 equiv.), EDC (1.2 equiv.), DMAP (0.1 equiv.), pent-4-enoic acid (1.2 equiv.), and triethylamine (3 equiv.). Dichloromethane (20 mL) was added to the flask and the reaction allowed to stir overnight. The reaction was filtered and the filtrate was washed with 1M HCl, aq. Saturated $NaHCO_3$, then water. The organic layer was dried over Na_2SO_4 then concentrated to provide the desired product as a white solid (771 mg, 64% yield).

IR (FT-ATR, cm^{-1}, neat): 2945, 2867, 1734, 1467, 1437, 1375, 1364, 1297, 1272, 1254, 1172, 1137, 1119, 1086, 1027, 996, 980, 958, 912, 800.

^{1}H NMR (500 MHz, $CDCl_3$): δ 5.88 – 5.76 (m, 1H), 5.37 (dq, *J* = 5.1, 1.6 Hz, 1H), 5.06 (dq, *J* = 17.5, 1.6 Hz, 1H), 5.02 – 4.97 (m, 1H), 4.67 – 4.57 (m, 1H), 2.38 – 2.36 (m, 4H), 2.31 (d, *J* = 8.8, 2H), 2.04 – 1.92 (m, 2H), 1.89 – 1.77 (m, 3H), 1.64 – 1.41 (m, 8H), 1.39 – 1.30 (m, 3H), 1.28 – 1.20 (m, 1H), 1.20 – 1.05 (m, 7H), 1.02 (s, 3H), 1.00 – 0.95 (m, 2H), 0.91 (d, *J* = 6.5 Hz, 3H), 0.87 (d, *J* = 2.3 Hz, 3H), 0.86 (d, *J* = 2.3 Hz, 3H), 0.67 (s, 3H).

^{13}C{^{1}H} NMR (126 MHz, $CDCl_3$): δ 172.6, 139.8, 136.9, 122.8, 115.6, 74.1, 56.8, 56.3, 50.2, 42.5, 39.9, 39.7, 38.3, 37.1, 36.7, 36.3, 35.9, 34.0, 32.1, 32.0, 29.1, 28.4, 28.2, 28.0, 24.4, 24.0, 23.0, 22.7, 21.2, 19.5, 18.9, 12.0.

HRMS (APCI): Exact Mass calculated for $[M – C_5H_8O_2]^+$ requires *m/z* = 369.3516. Found 369.3519 (APCI).

9. Mechanistic Investigations

Stern-Volmer Quenching Experiments

Absorption and Emission experiments for monitoring quenching of the iridium photocatalyst were conducted by using an excitation wavelength of 415 nm and an emission wavelength of 515 nm. Fluorescence emission spectra were obtained with excitation and emissions slit widths of 0.5 and 1.0 nm, respectively. Reported intensities are the average value of three separate scans.

All reagents were prepared as stock solutions inside a N_2-filled glove box. Reagents were diluted in acetonitrile and sealed in a screw-top 1.0 cm quartz cuvette. A blank composed of acetonitrile was used in absorbance measurements. Samples for quenching experiment were dispensed from a stock solution of Ir[dF(Me)ppy]$_2$(dtbbpy)PF$_6$ (0.45 mM in MeCN, amount dispensed: 1000 μL, 0.45 μmol, 0.15 M after dilution) followed by addition of the indicated species that were also prepared from stock solutions in MeCN. N–H azole employed in these studies is indicated in each experiment.

Quenching with Phosphines

Table S30. Photocatalyst and phosphine quenching species.

Species	Concentration
[Ir(dF(Me)ppy)$_2$(dtbbpy)]PF$_6$	0.15 mM
(*p*-OMePh)$_3$P	0.0 – 4.0 mM
PPh$_3$	0.0 – 4.0 mM

Table S31. Photocatalyst emission data with P(*p*-OMePh)$_3$ quencher.

[(*p*-OMePh)$_3$P] (mM)	Intensity	I_0/I
0.0	126430	1
1.0	33420	3.783064
2.0	19393	6.519251
3.0	13926	9.078267
4.0	10953	11.542605

Table S32. Photocatalyst emission data with PPh$_3$ quencher.

[PPh$_3$] (mM)	Intensity	I_0/I
0.0	98870	1
1.0	91946	1.075297
2.0	83206	1.188246
3.0	76543	1.291687
4.0	70606	1.400293

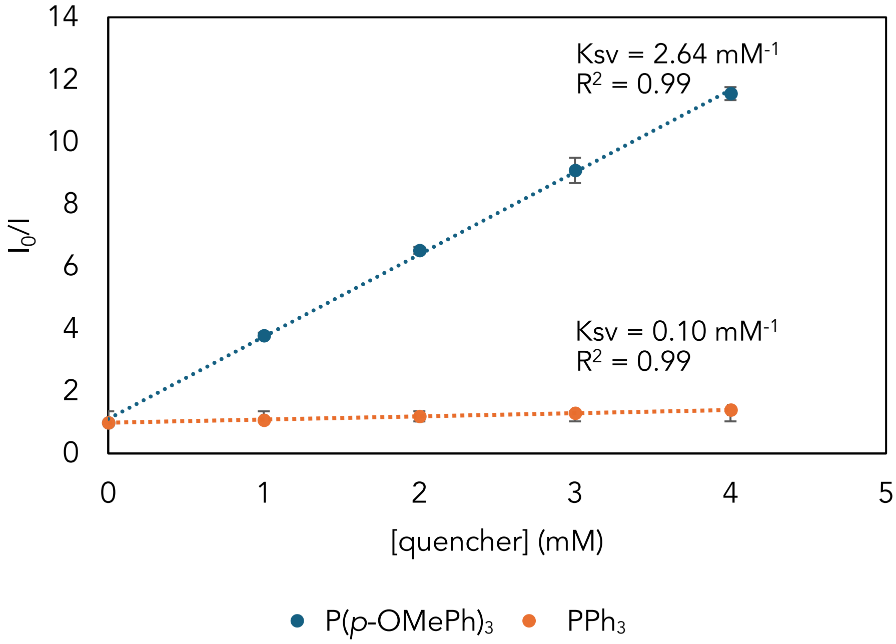

Fig. S2. Plot of I_0/I with (*p*-OMePh)$_3$P and PPh_3 quenchers.

Both phosphines quench the photocatalyst; P(*p*-OMePh)$_3$ is a more efficient quencher since it is more electron-rich.

Quenching with PPh_3 and standard substrates

The following studies were performed to evaluate if the combination of phosphine and N–H azole, or phosphine and alkene, would be a more efficient quencher than phosphine alone, probing the possibility of a PCET mechanism for the former[4], and indicating if the alkene is activated by the phosphine for the latter.

Table S33. Photocatalyst and quenching species.

Species	Concentration
[Ir(dF(Me)ppy)$_2$(dtbbpy)]PF_6	0.15 mM
3-phenylpyrazole	10 mM
PPh_3	0.0 – 4.0 mM

Table S34. Photocatalyst emission data with PPh_3 and 3-phenylpyrazole quenchers.

[PPh_3] (mM))	[3-phenylpyrazole] (mM)	Intensity	I_0/I
0.0	10	65623	1
1.0	10	61600	1.065314
2.0	10	57330	1.144660
3.0	10	54083	1.213374
4.0	10	51073	1.284884

Table S35. Photocatalyst and quenching species.

Species	Concentration
$[Ir(dF(Me)ppy)_2(dtbbpy)]PF_6$	0.15 mM
1-hexene	10 mM
PPh_3	0.0 – 4.0 mM

Table S36. Photocatalyst emission data with PPh_3 and 1-hexene quenchers.

$[PPh_3]$ (mM))	[1-hexene] (mM)	Intensity	I_0/I
0.0	10	85903	1
1.0	10	77183	1.112978
2.0	10	71866	1.195315
3.0	10	65126	1.319019
4.0	10	60003	1.431643

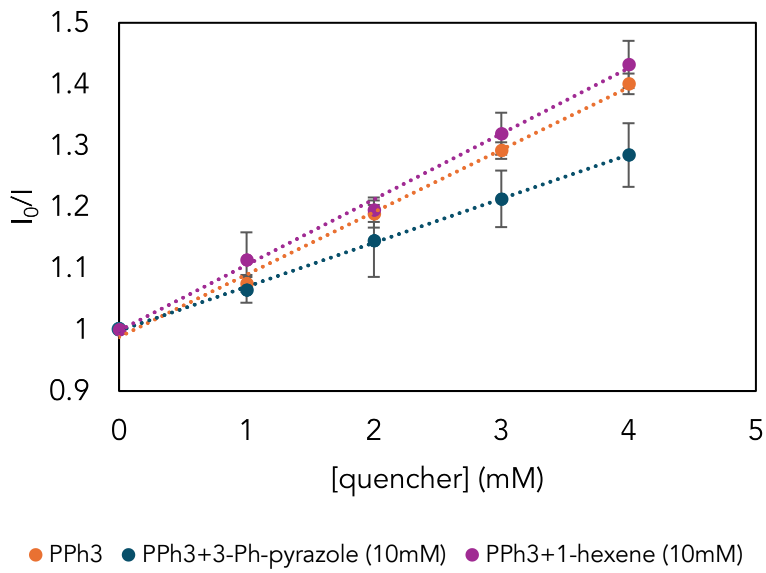

Fig. S3. Plot of I_0/I with combination of PPh_3 and standard substrate quenchers.

The combination of PPh_3 with either standard azole or alkene quenches the photocatalyst similarly to PPh_3 alone, supporting the proposed mechanism that a phosphine radical cation is independently generated by the excited state photocatalyst. The slightly reduced quenching for PPh_3 + 3-phenylpyrazole (purple) is consistent with an H-bond interaction between the two components that reduces electron density of the phosphine and will consequently depress quenching.

Quenching with 3-phenylpyrazole

Table S37. Photocatalyst and N–H azole quenching species.

Species	Concentration
$[Ir(dF(Me)ppy)_2(dtbbpy)]PF_6$	0.15 mM
3-phenylpyrazole	0.0 – 4.0 mM

Table S38. Photocatalyst emission data with 3-phenylpyrazole quencher.

[3-phenylpyrazole] (mM)	Intensity	I_0/I
0.0	93196	1
1.0	93946	0.992017
2.0	89826	1.037517
3.0	91006	1.024064
4.0	88763	1.049946

Quenching with 1-hexene

Table S39. Photocatalyst and alkene quenching species.

Species	Concentration
$[Ir(dF(Me)ppy)_2(dtbbpy)]PF_6$	0.15 mM
1-hexene	0.0 – 4.0 mM

Table S40. Photocatalyst emission data with 1-hexene quencher.

[1-hexene] (mM)	Intensity	I_0/I
0.0	129680	1
1.0	128140	1.012018
2.0	127840	1.014393
3.0	126903	1.02188
4.0	131753	0.984264

Quenching with 4-phenylimidazole

Table S41. Photocatalyst and N–H azole quenching species.

Species	Concentration
$[Ir(dF(Me)ppy)_2(dtbbpy)]PF_6$	0.15 mM
4-phenylimidazole	0.0 – 4.0 mM

Table S42. Photocatalyst emission data with 4-phenylimidazole quencher.

[4-phenylimidazole] (mM)	Intensity	I_0/I
0.0	173160	1
1.0	176243	0.982505
2.0	173390	0.998674
3.0	173080	1.000462
4.0	173620	0.997351

Quenching with indole

Table S43. Photocatalyst and N–H azole quenching species.

Species	Concentration
$[Ir(dF(Me)ppy)_2(dtbbpy)]PF_6$	0.15 mM
Indole	0.0 – 4.0 mM

Table S44. Photocatalyst emission data with indole quencher.

[Indole] (mM)	Intensity	I_0/I
0.0	169160	1
1.0	146723	1.152918
2.0	127253	1.329317
3.0	110080	1.536701
4.0	98960	1.709378

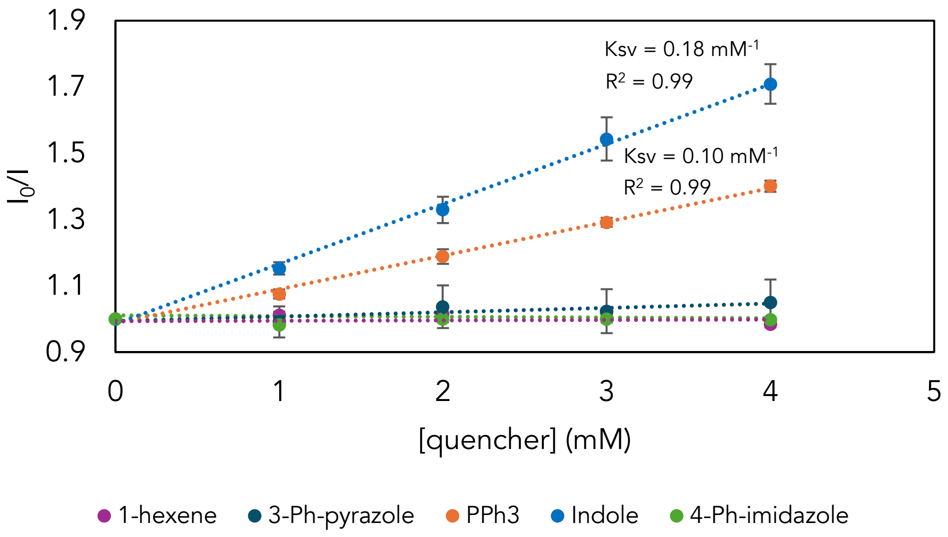

Fig. S4. Comparison of I0/I vs. [quencher] for PPh_3 and N–H azole substrates.

PPh_3 is a more efficient quencher than standard N–H azoles 3-phenylpyrazole and 4-phenylimidazole, supporting the mechanism where a phosphine radical cation is first generated. Indole is a substrate that is a more efficient quencher than PPh_3, thus an even more efficient phosphine quencher, such as P(*p*-OMePh)$_3$, is required to enable reactivity.

Light-on Light-off Cycling

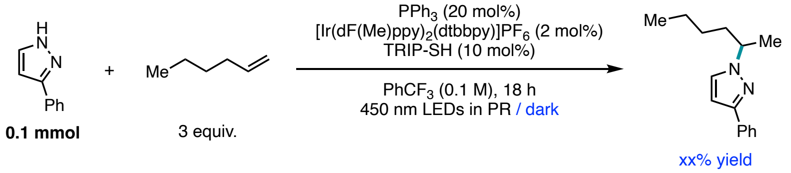

These reactions were performed according to General Procedure A. During the dark periods, the reaction vials were removed from the photoreactor, wrapped in aluminum foil, and left to stir on a stir plate at 1500 rpm for the indicated time interval.

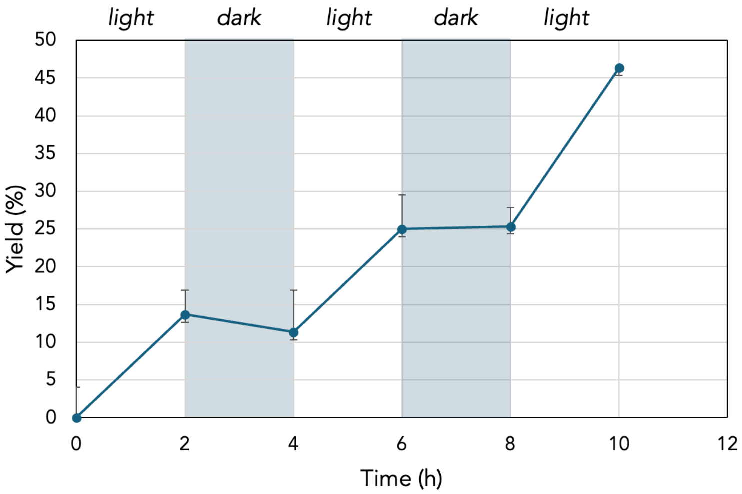

Fig. S5. Light-on light-off experiments.

Yields are an average of three runs and determined by ^1H NMR in $CDCl_3$ with 1,3,5-TMB as internal standard. The absence of reactivity during dark periods suggests there to be insignificant propagation, with the caveat that light-dark experiments cannot fully rule out short chain propagation[5].

Quantum Yield Measurement

The quantum yield was determined using the protocol by Ruos, M. et al.[6]

Table S45. Absorption data of $Fe(phen)_3^{2+}$ at 510nm.

	Run 1		*Run 2*		*Run 3*	
Time of irradiation (s)	Measured absorbance (a.u.)	ΔAbsorbance (a.u.)	Measured absorbance (a.u.)	ΔAbsorbance (a.u.)	Measured absorbance (a.u.)	ΔAbsorbance (a.u.)
0	0.299	0	0.208	0	0.164	0
10	0.410	0.111	0.355	0.146	0.398	0.234
20	0.644	0.344	0.463	0.255	0.546	0.382
30	0.868	0.568	0.784	0.576	0.811	0.646
40	1.124	0.824	1.412	1.204	0.955	0.791
R^2	–	0.990	–	0.925	–	0.997

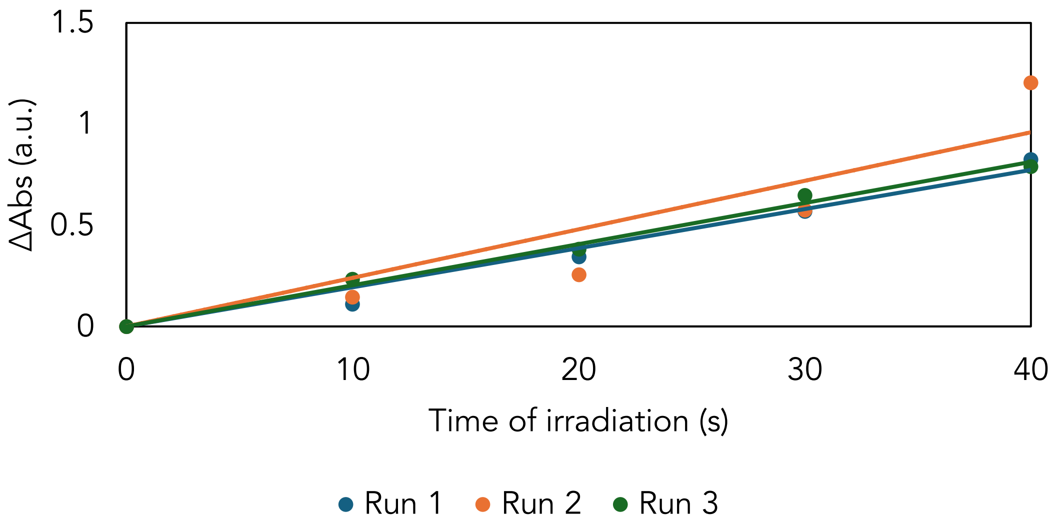

Fig. S6. Change in relative absorbance over irradiation of $Fe(phen)_3^{2+}$ at 510nm.

From Table S45, the number of iron ions produced by ferrioxalate photodegradation as a function of time can be determined by:

$$mol\ of\ Fe^{2+} = \frac{\Delta Abs_{510nm} V_1 V_3}{\varepsilon_{510nm} l V_2}$$

where

ΔAbs_{510nm} = difference in absorbance at 510 nm between sample and sample at time = 0
l = path length of the cuvette (1 cm)
ε_{510nm} = extinction coefficient of $Fe(phen)_3^{2+}$ complex at 510 nm (ε = 11,100 $M^{-1}cm^{-1}$)
V_1 = total volume of irradiated solution (1 mL)
V_2 = volume of aliquot taken from V_1 (20 µL)
V_3 = the volume that V_2 is diluted into (10 mL)

Table S46. $Fe(phen)_3^{2+}$ photochemical production.

	Run 1	*Run 2*	*Run 3*
Time of irradiation (s)	Fe^{2+} produced ($\times 10^{-5}$ mol)	Fe^{2+} produced ($\times 10^{-5}$ mol)	Fe^{2+} produced ($\times 10^{-5}$ mol)
0	0	0	0
10	0.500	1.147	1.053
20	1.550	0.658	1.719
30	2.560	2.592	2.912
40	3.712	5.421	3.562

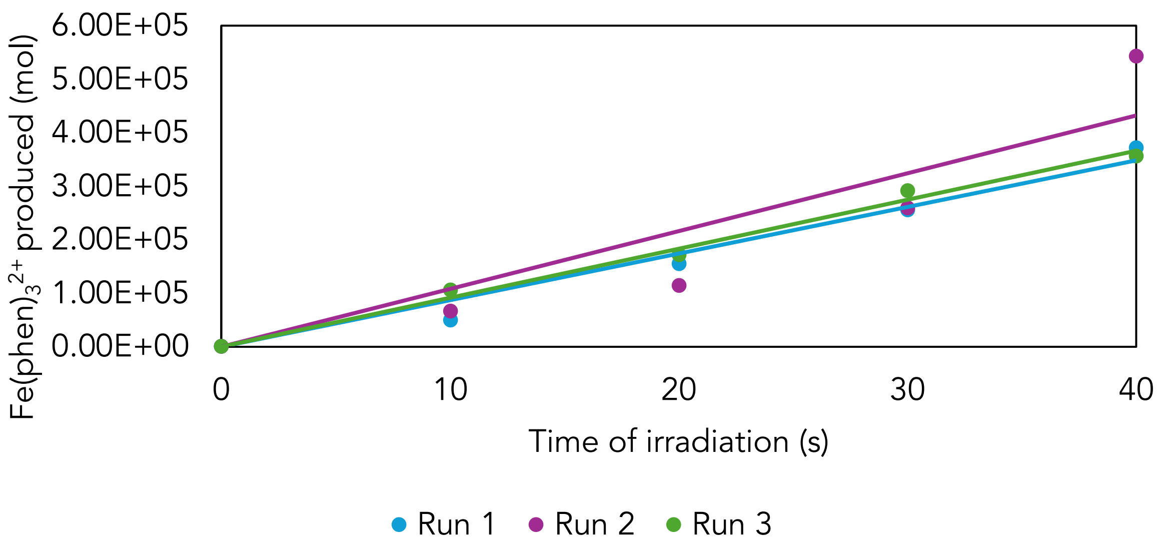

Fig. S7. $Fe(phen)_3^{2+}$ photochemical production over time.

From Table S46, the total number of photons to pass through the cuvette as a function of time can be determined by:

$$photons\ absorbed = \frac{mol\ of\ Fe^{2+}}{\emptyset_{436nm} \times F}$$

where

$\emptyset_{436nm}$ = quantum efficiency for wavelength and concentration (1.012 at 0.15 M ferrioxalate solution[7])

F = fraction of light absorbed by the ferrioxalate solution (absorbance of a 0.15 M potassium ferrioxalate solution is 1.7 a.u. at 450 nm (from absorption spectrum), implying that F = 0.98 ~ 1 at 450 nm)

$$F = 1 - 10^{-Abs_{450nm}}$$
$$F = 1 - 10^{-1.7} = 0.98$$

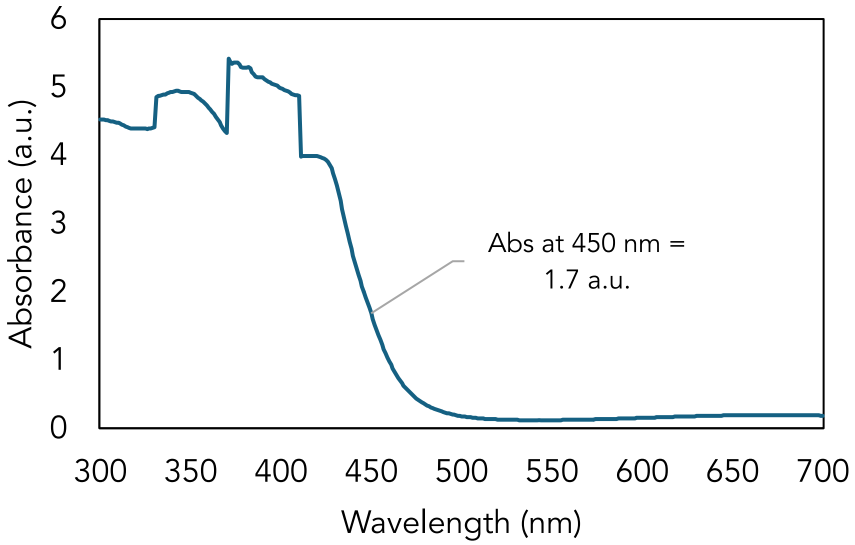

Fig. S8. Absorption spectrum for 0.15 M potassium ferrioxalate solution.

Table S47. Determination of photon flux.

	Run 1	*Run 2*	*Run 3*
**Time of irradiation (s)**	**Photons absorbed ($\times10^{-5}$ Einstein)**	**Photons absorbed ($\times10^{-5}$ Einstein)**	**Photons absorbed ($\times10^{-5}$ Einstein)**
0	0	0	0
10	0.504	0.664	1.063
20	1.563	1.158	1.734
30	2.582	2.616	2.938
40	3.745	5.470	3.594
Slope = photon flux ($\times10^{-6}$ Einstein/s)	0.088 ± 0.004	0.109 ± 0.016	0.092 ± 0.002
R^2	0.990	0.925	0.997

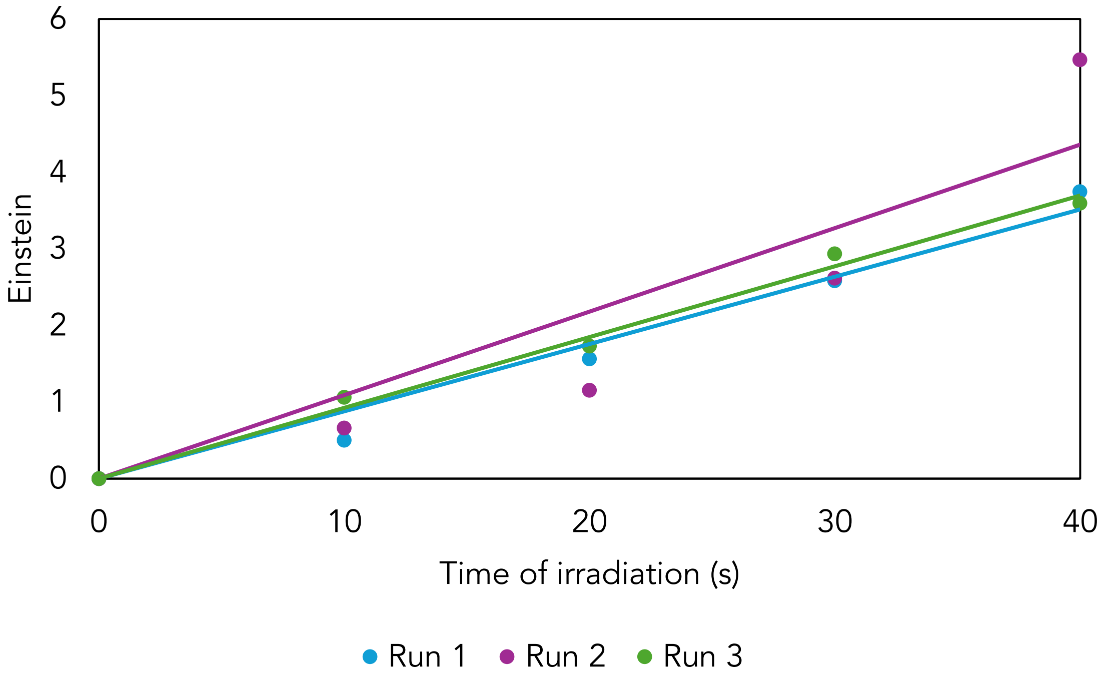

Fig. S9. Photon absorption over time.

The average photon flux is 0.096 ± 0.007 x 10^{-6} Einstein/s.

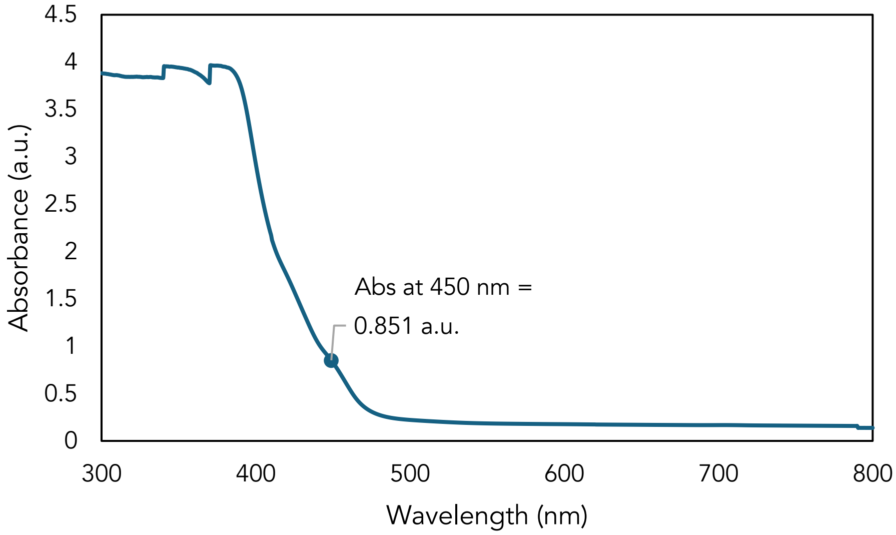

Fig. S10. Absorption spectrum of $[Ir(dF(Me)ppy)_2(dtbbpy)]PF_6$ (catalytically relevant concentration of 0.004 M) in $PhCF_3$.

$F = 1 - 10^{-\text{Abs at 450 nm}}$

$F = 1 - 10^{-0.851} = 0.859$

This value indicates majority of the photon flux determined by actinometry is absorbed by the photocatalyst at catalytically relevant concentrations. The remaining photon flux not accounted for (F ~ 0.14) is likely due to the photocatalyst having poor solubility in the solvent at the beginning of the reaction, which the UV-vis spectrum is representative of.

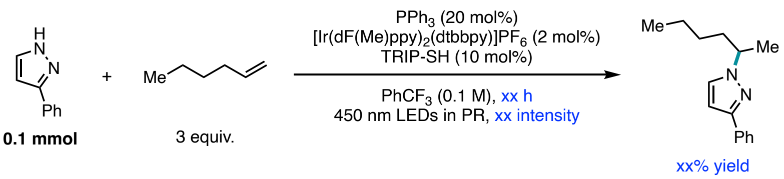

Table S48. Initial rate of product formation (first few entries from Table 12).

Time of irradiation (s)	Product yield in % (x10^{-4} mmol)
0	0
3600	3
7200	11
14400	23
Slope = initial rate (x10^{-9})	0.016
R^2	0.985

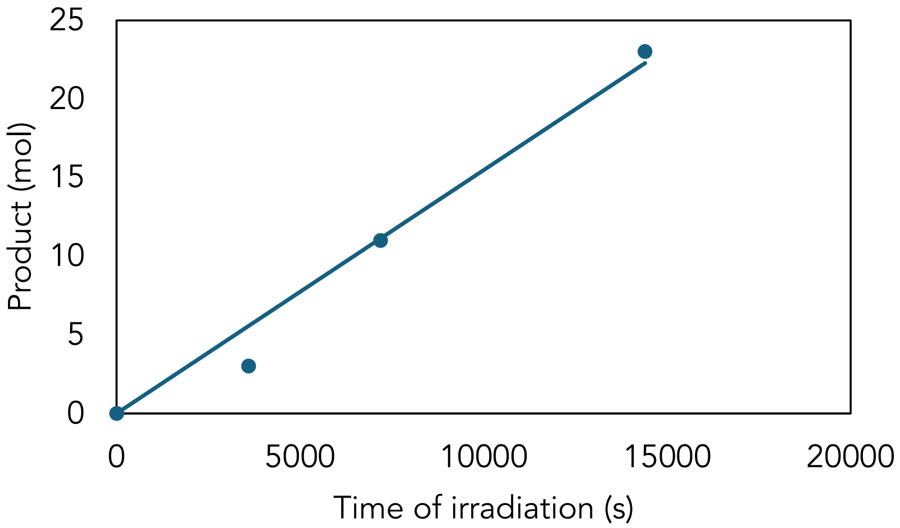

Fig. S11. Initial rate for product formation.

Using the initial rate of product formation of 0.016 x 10^{-9} mol/s, the quantum yield was determined:

$$Quantum\ yield = \frac{Product\ rate}{Photon\ flux\ rate} = \frac{0.016\ \times\ 10^{-9}\ mol/s}{0.096\ \times\ 10^{-6}\ Einstein/s} = 0.00017$$

This value indicates that the reaction is not proceeding via a chain mechanism.

Phosphorus Speciation Experiments

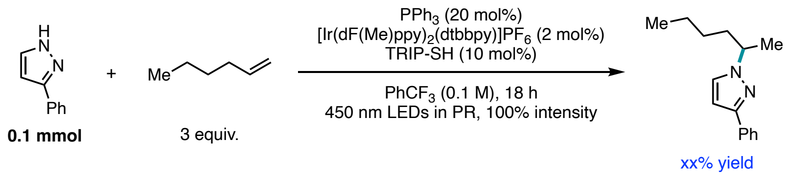

^{31}P NMR studies were conducted to evaluate the phosphorus species formed during the standard reaction shown above. To avoid potential quenching of intermediary phosphine species by exposure to air during sample preparation, a few modifications were made to the setup. The reaction was prepared according to Procedure A, omitting the stir bar and using toluene-d8 as the solvent instead. The reaction was then transferred to a J-Young tube and sealed. The J-Young tube was placed 2 cm away from a 427 nm PR160L Kessil Lamp, and was irradiated at 100% intensity for 18 h. The tube was inverted to mix the reaction every 30 mins for the first and last two hours. The J-Young tube was directly subject to NMR analysis and the spectra referenced to triphenylphosphine (-6 ppm).

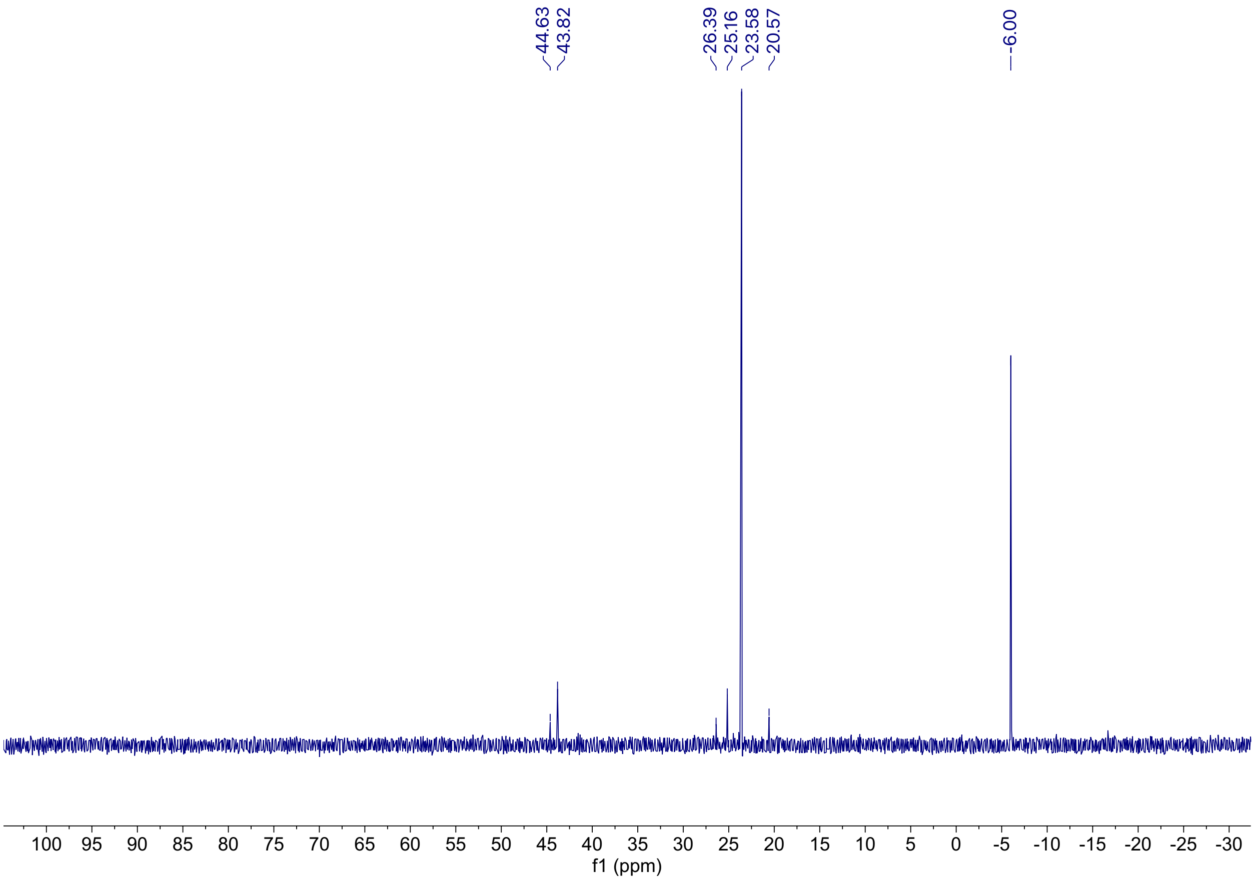

Fig. S12. ^{31}P NMR spectra of standard reaction run in a J-Young tube.

The absence of triphenylphosphine oxide (expected δ ~28 ppm) as well as presence of triphenyl phosphine (δ -6 ppm) supports the catalytic nature of the phosphine as proposed. We attribute the tallest peak at δ 23.58 to **B1**, and the smaller peaks at δ ~20-26 ppm, as well as the peaks at δ ~44 ppm, to side products resulting from alkene and azole addition into the phosphine radical cation [4,8,9]. The unproductive formation of **B1** and other side products likely necessitates a higher phosphine catalyst loading (20 mol%) for high yield of desired product.

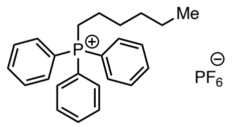

B1

To determine the origin of these peaks, exclusion experiments were run with the listed change and prepared according to Procedure A. After the reaction was completed, the vial was uncapped and the solvent evaporated in vacuo. An NMR sample of the crude was made with $CDCl_3$. All reactions are referenced to triphenylphosphine (δ -6 ppm).

No heterocycle, 1 equiv. alkene:

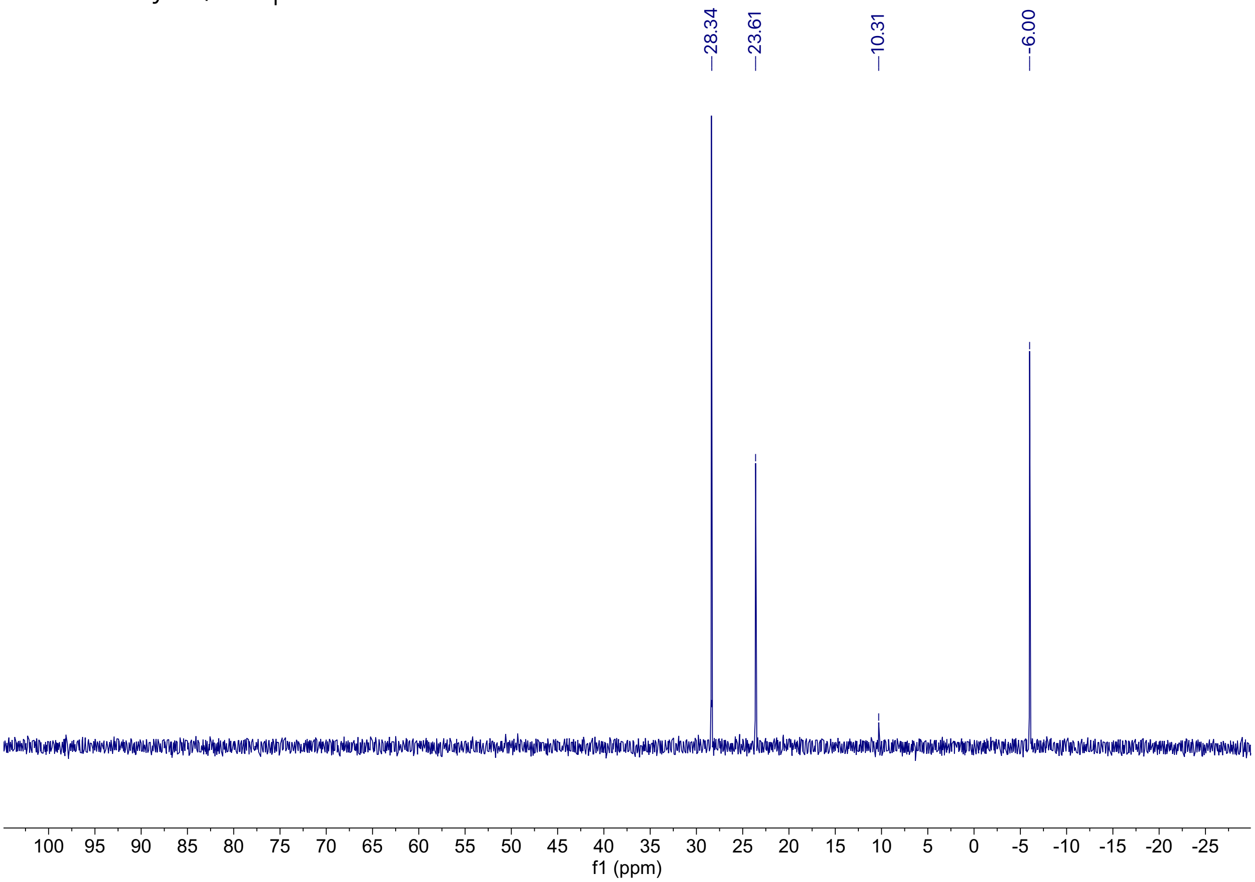

Fig. S13. ^{31}P NMR spectra of standard reaction with no heterocycle and 1 equiv. alkene.

Other than phosphine (δ -6 ppm) and phosphine oxide (δ 28.34 ppm), the latter of which likely arises when the vial is opened to air to make the NMR sample, the major phosphorus species in the reaction after completion is assigned to be phosphonium species **B1** (δ 23.61 ppm) by comparison to independently synthesized material **B2** (δ 24.51 ppm). This species is expected to form from alkene addition into the phosphine radical cation, followed by subsequent HAT by the thiol catalyst.

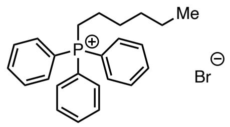

B2

No alkene:

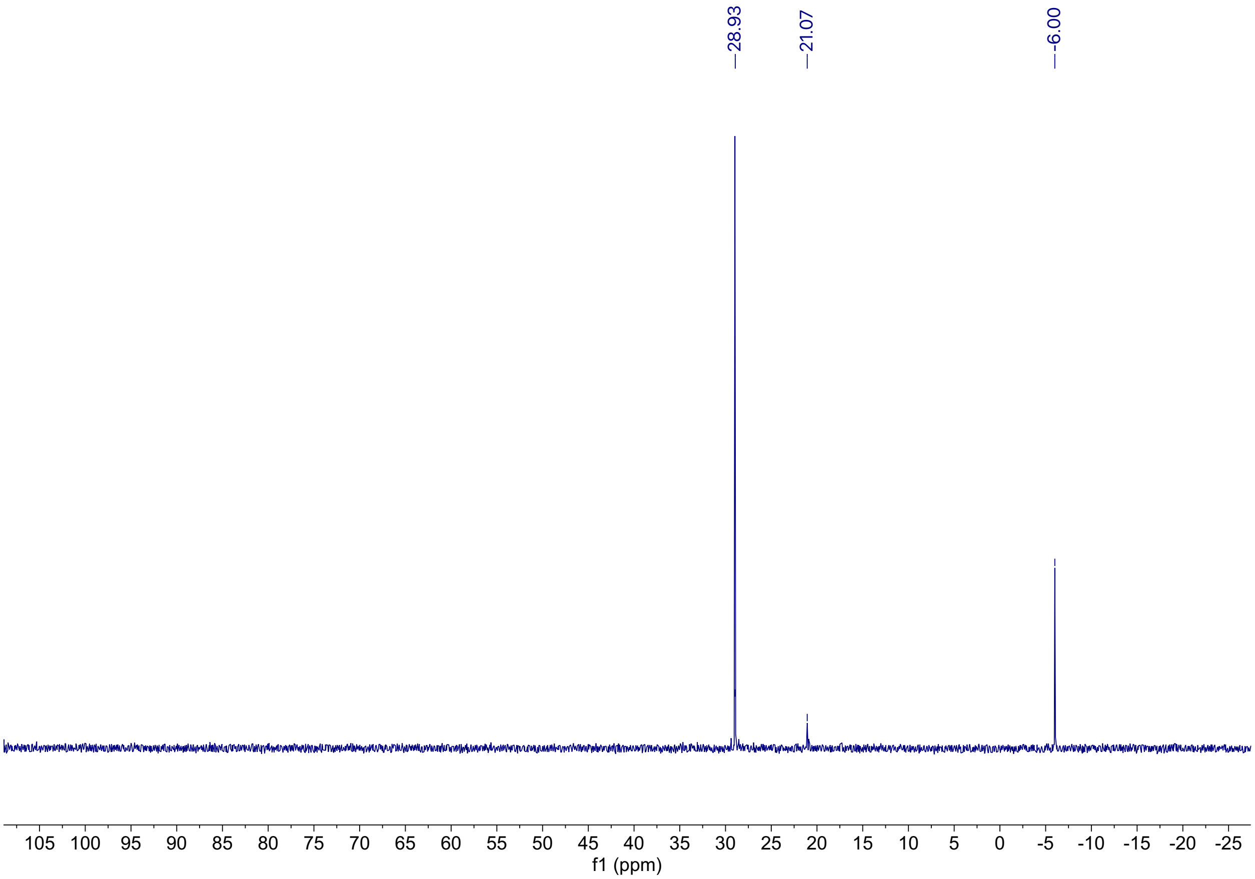

Fig. S14. ^{31}P NMR spectra of standard reaction with no alkene.

The major phosphine species are unreacted phosphine (δ -6 ppm) and phosphine oxide (δ 28.93 ppm). The small peak at δ 21.07 ppm could be a decomposition product resulting from azole or thiol addition into the phosphine radical cation[8,9].

No thiol:

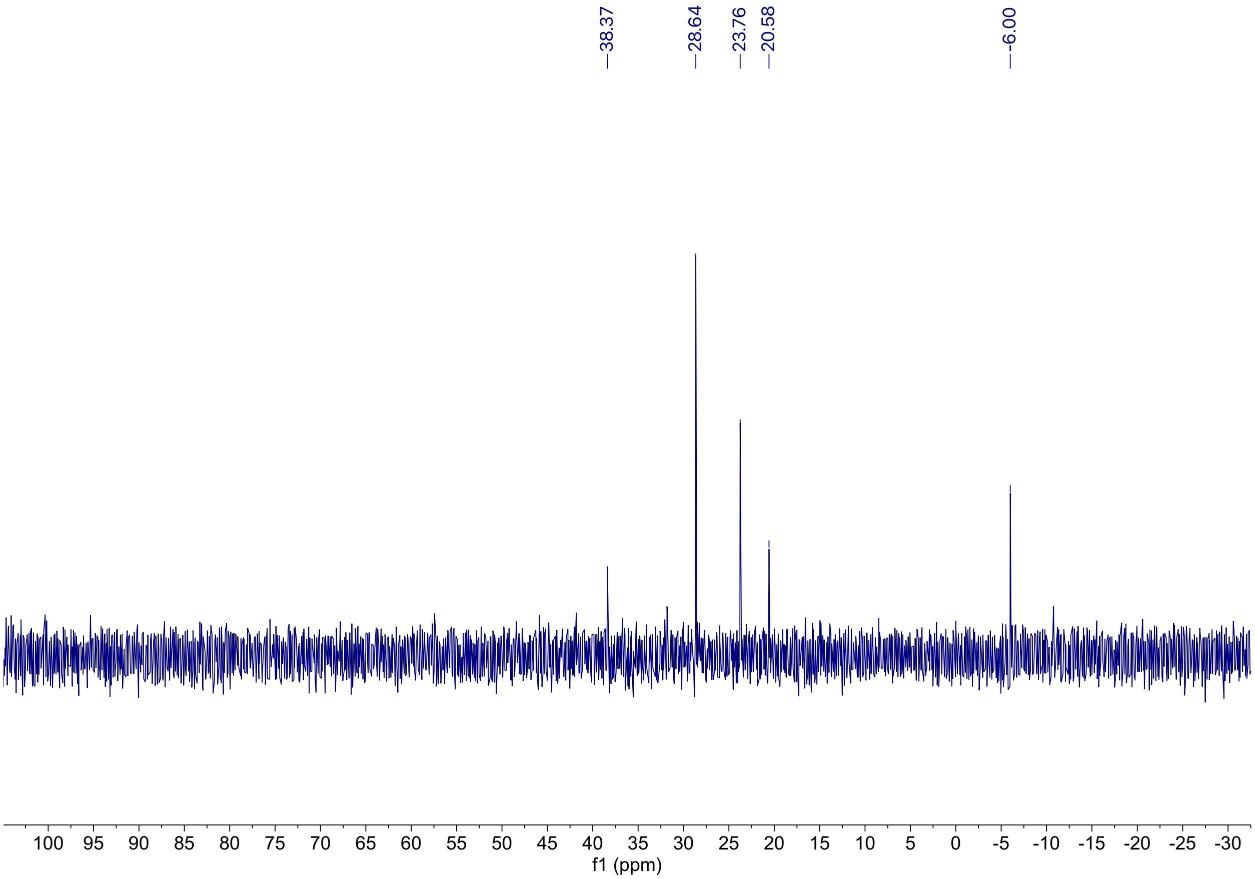

Fig. S15. ^{31}P NMR spectra of standard reaction with no thiol.

Compared to the other exclusion experiments, two new peaks appear at δ 20.56 ppm and δ 38.37 ppm when the reaction is run without thiol catalyst. These peaks may arise from azole or thiol addition into the phosphine radical cation and subsequent downstream side reactivity[8,9].

Synthesis of phosphonium bromide

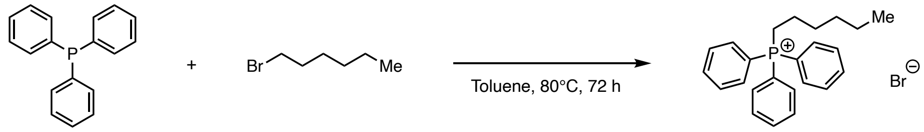

(1-Hexyl)triphenylphosphonium bromide (**B2**). In a round bottom flask equipped with a Teflon-coated magnetic stirbar under an atmosphere of N_2, triphenylphosphine (0.65 g, 2.48 mmol, 1 equiv.) in dry toluene (1.0 M) was heated to 80°C. Bromohexane (0.88 uL, 6.20 mmol, 2.5 equiv.) was added dropwise. The reaction was allowed to stir for 72 hours, and conversion was observed by ^{31}P NMR. After cooling to room temperature, the crude reaction was filtered over a glass frit and **S4** was collected as a white solid (0.99 g, 99% yield).

IR (FT-ATR, cm^{-1}, neat): 3410, 3054, 2955, 2926, 2859, 1618, 1586, 1484, 1466, 1438, 1406, 1339, 1318, 1189, 1162, 1114, 995, 749, 723, 691.
^{1}H NMR (500 MHz, CDCl$_3$) δ 7.89 – 7.82 (m, 6H), 7.81 – 7.74 (m, 3H), 7.74 – 7.67 (m, 6H), 3.87 – 3.73 (m, 2H), 1.65 – 1.59 (m, 4H), 1.26 – 1.20 (m, 4H), 0.86 – 0.77 (m, 3H).
^{13}C{^{1}H} NMR (126 MHz, CDCl$_3$) δ 135.1, 135.0, 133.9, 133.8, 130.7, 130.6, 119.0, 118.3, 31.51, 31.50, 30.3, 30.2, 23.1, 22.81, 22.77, 22.7, 22.4, 14.1.
^{31}P NMR (162 MHz, CDCl$_3$) δ 24.51.
HRMS (APCI): Exact Mass calculated for $[C_{24}H_{28}P]^+$ requires *m/z* = 347.1923. Found 347.1923.

Assessing catalytic relevance of phosphonium

Given the observation of phosphonium salt in the crude reaction mixture, we conducted the following experiments with phosphonium salt **B2** as a substitute for **B1** to assess if this species is catalytically competent.

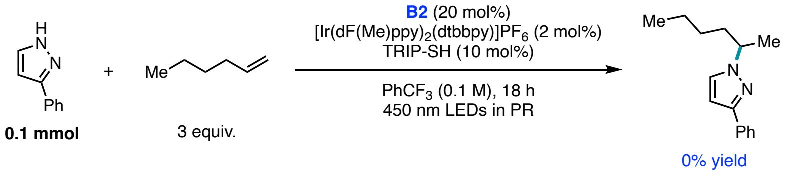

Fig. S16. Scheme showing reaction with catalytic phosphonium **B2** instead of triphenylphosphine.

The reaction was prepared according to procedure A, substituting the phosphine catalyst for 1-hexyl)triphenylphosphonium bromide **B2**. No desired Markovnikov product was observed by ^{1}H NMR of the reaction mixture, indicating **B2** to be catalytically inactive.

Crossover experiment with phosphonium

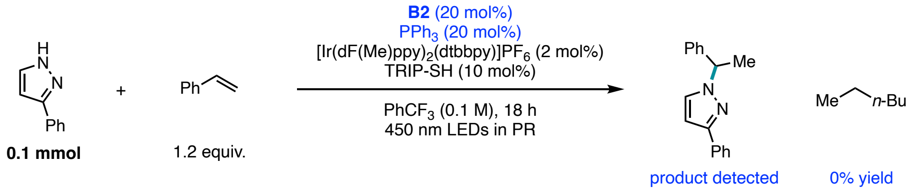

Fig. S17. Crossover experiment with catalytic phosphonium **B2** and triphenylphosphine.

The reaction was prepared according to Procedure A, adding 20 mol% of **B2** in addition to triphenylphosphine catalyst. As expected with the presence of phosphine, Markovnikov product was observed by ^{1}H NMR of the reaction mixture. Hexane, which would arise from reduction and subsequent P–C α-scission of **B2**, was not observed.

Radical Cyclization Experiments

Radical cyclization with 1,5-hexadiene

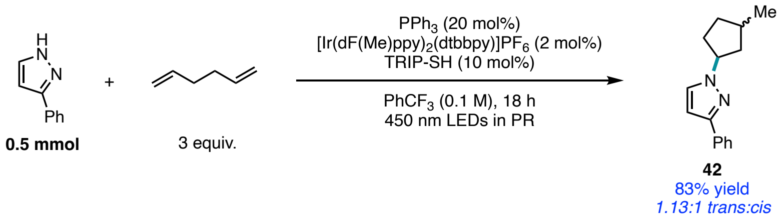

1-(3-methylcyclopentyl)-3-phenyl-1*H*-pyrazole (48): The reaction was set up following general procedure B1 using 3-phenylpyrazole (72.1 mg, 0.500 mmol, 1.00 equiv.), 1,5-hexadiene (178 μL, 1.50 mmol, 3.00 equiv.), and triphenylphosphine (26.2 mg, 0.100 mmol, 0.200 equiv.). The crude product was purified using silica gel flash column chromatography eluting with Et_2O/Hex (0% → 15%, eluted 15%) to give the title compound as a yellow oil and a mixture of diastereomers (94 mg, 83% yield, *d.r.* 1.13:1 trans:cis).

IR (FT-ATR, cm^{-1}, neat): 3064, 3036, 2952, 2867, 1605, 1498, 1458, 1418, 1375, 1358, 1323, 1302, 1283, 1262, 1221, 1098, 1073, 1046, 1029, 1008, 948, 913, 774, 747, 693.

HRMS (APCI): Exact Mass calculated for $[C_{15}H_{19}N_2 + H]^+$ requires m/z = 227.1543. Found 227.1542.

Major diastereomer (*trans*):

^{1}H NMR (500 MHz, $CDCl_3$): δ 7.81 – 7.78 (m, 2H), 7.44 (d, J = 2.3 Hz, 1H), 7.37 (d, J = 6.2 Hz, 2H), 7.28 (d, J = 1.9 Hz, 1H), 6.53 (d, J = 2.5 Hz, 1H), 4.81 (dt, J = 13.6, 7.8 Hz, 1H), 2.35 – 2.28 (m, 1H), 2.27 – 2.17 (m, 2H), 2.08 – 2.02 (m, 2H), 1.80 – 1.72 (m, 1H), 1.27 – 1.23 (m, 1H), 1.06 (d, J = 6.6 Hz, 3H).

^{13}C{^{1}H} NMR (126 MHz, $CDCl_3$): δ 150.9, 133.9, 128.7, 128.6, 127.5, 125.7, 102.4, 62.9, 41.7, 33.6, 32.9, 32.8, 20.9.

Minor diastereomer (*cis*):

^{1}H NMR (500 MHz, $CDCl_3$): δ 7.84 – 7.79 (m, 2H), 7.45 (d, J = 2.3 Hz, 1H), 7.39 (d, J = 7.5 Hz, 2H), 7.32 – 7.27 (m, 1H), 6.53 (d, J = 2.5 Hz, 1H), 4.77 – 4.64 (m, 1H), 2.45 – 2.37 (m, 1H), 2.14 – 2.08 (m, 2H), 1.97 – 1.86 (m, 1H), 1.72 – 1.63 (m, 1H), 1.54 – 1.44 (m, 1H), 1.34 – 1.28 (m, 1H), 1.13 (d, J = 6.6 Hz, 3H).

^{13}C{^{1}H} NMR (126 MHz, $CDCl_3$): δ 150.9, 133.9, 128.7, 128.6, 127.5, 125.7, 102.5, 63.2, 42.2, 33.6, 33.5, 32.8, 20.6.

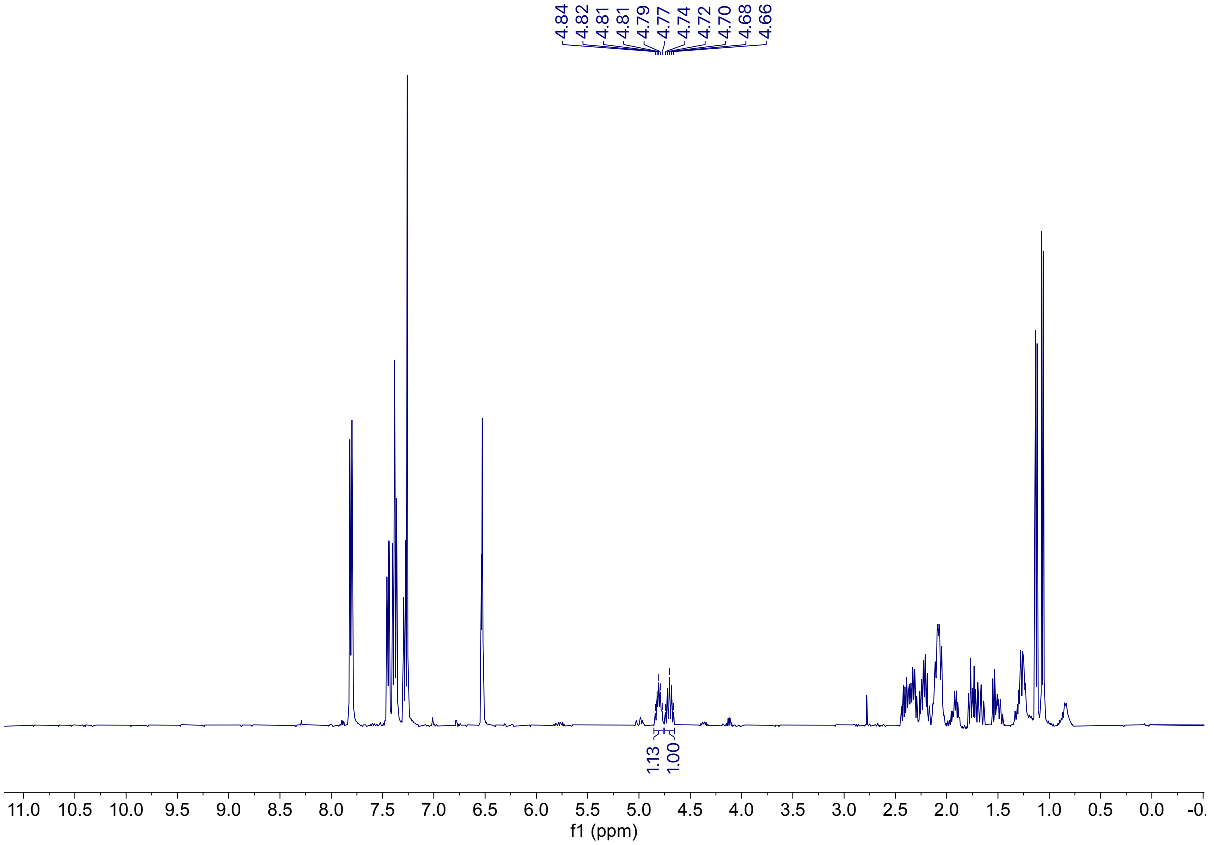

Fig. S18. ^{1}H NMR spectra of **48** as a mixture of diastereomers.

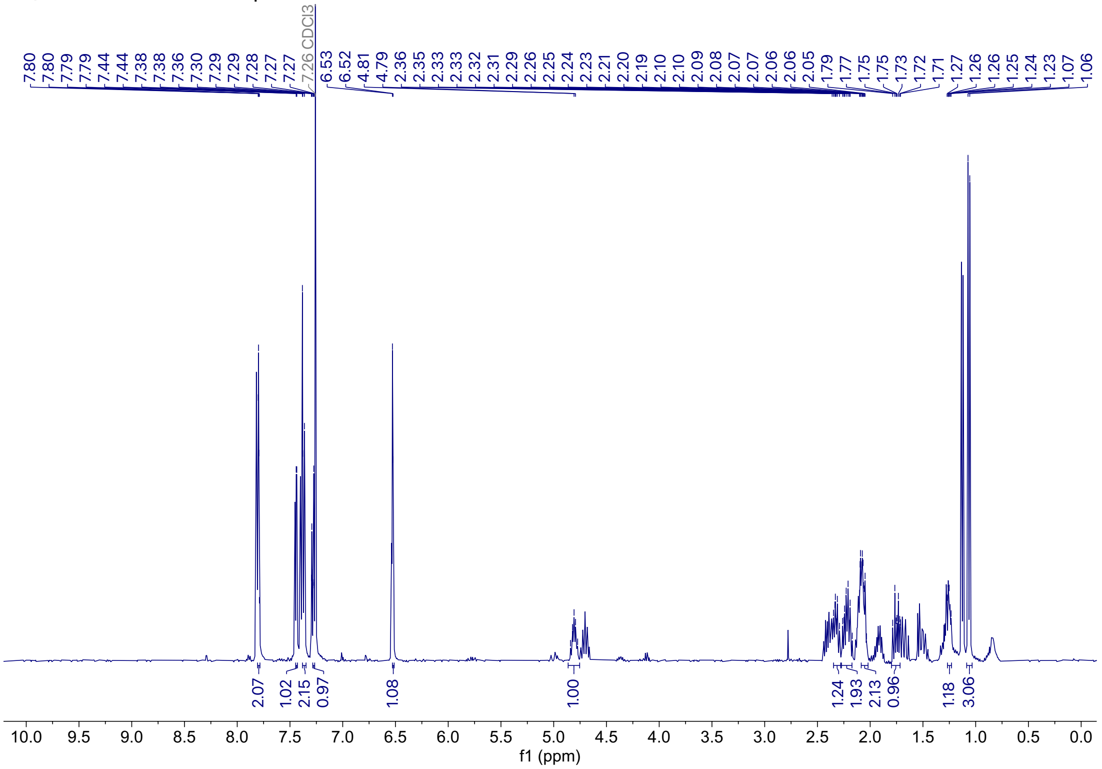

Fig. S19. ^{1}H NMR spectra of **48** as a mixture of diastereomers, peak shifts and integrations for *trans* (major) diastereomer.

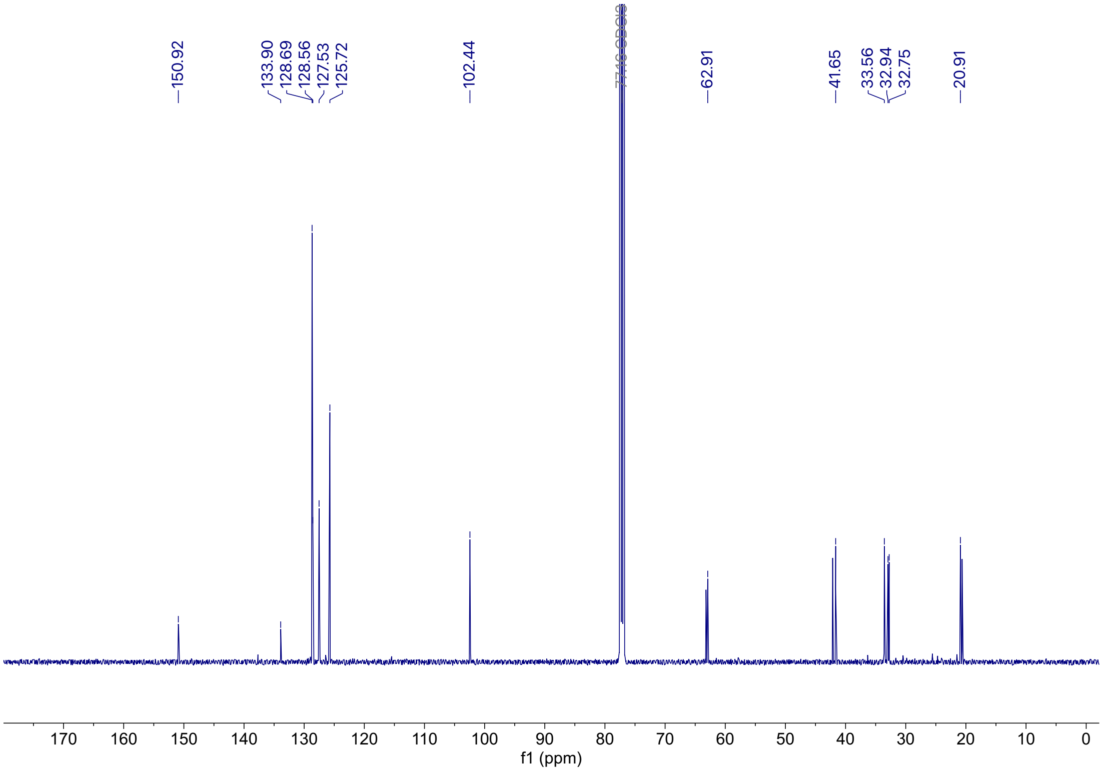

Fig. S20. $^{13}C\{^{1}H\}$ NMR spectra of **48** as a mixture of diastereomers, peaks shifts for *trans* (major) diastereomer.

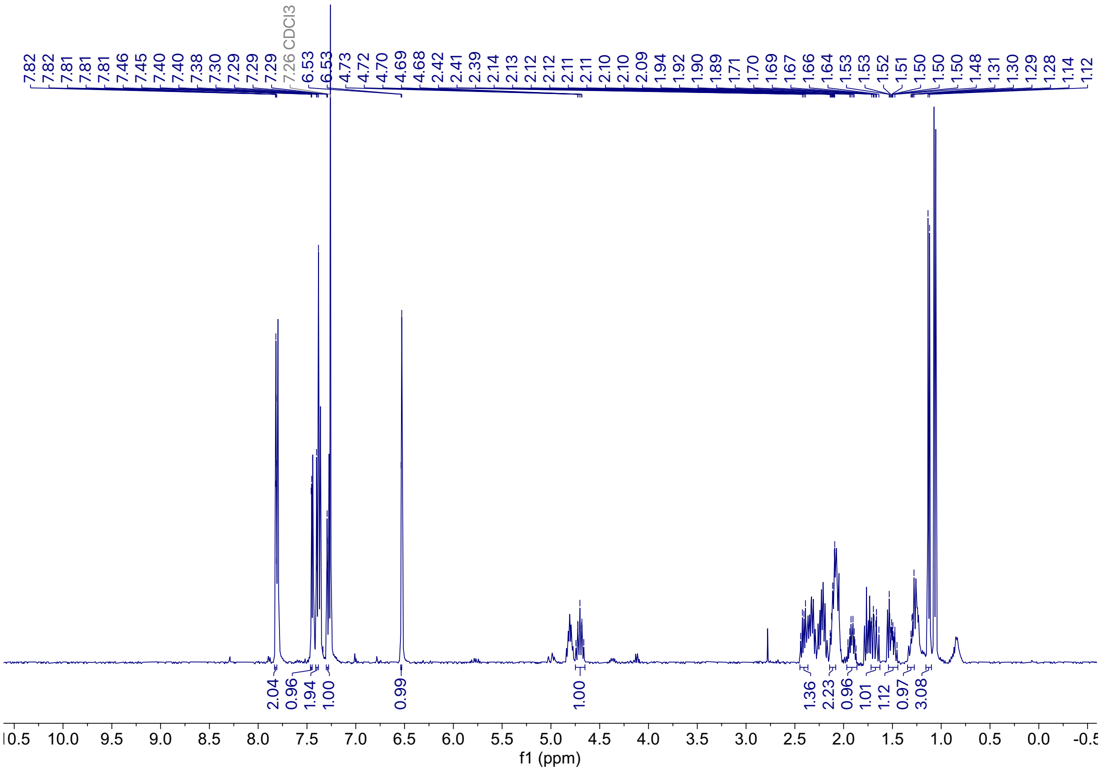

Fig. S21. ^{1}H NMR spectra of **48** as a mixture of diastereomers, peak shifts and integrations for *cis* (minor) diastereomer.

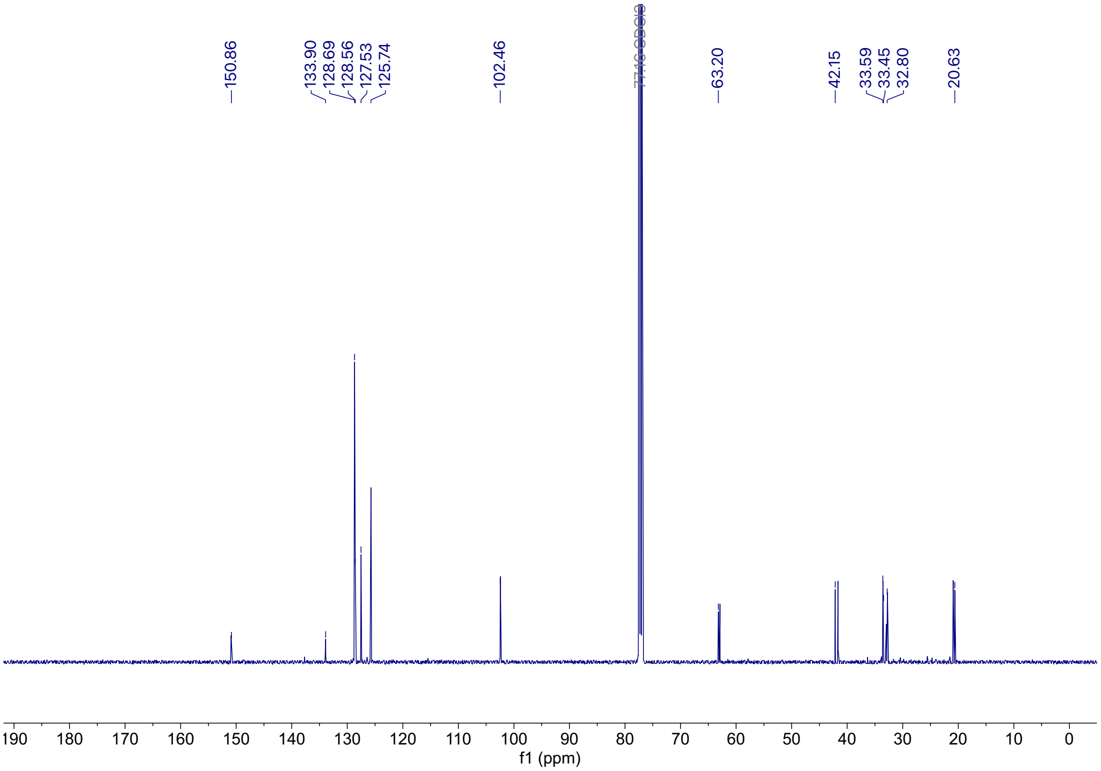

Fig. S22. $^{13}C\{^1H\}$ NMR spectra of **48** as a mixture of diastereomers, peaks shifts for *cis* (minor) diastereomer.

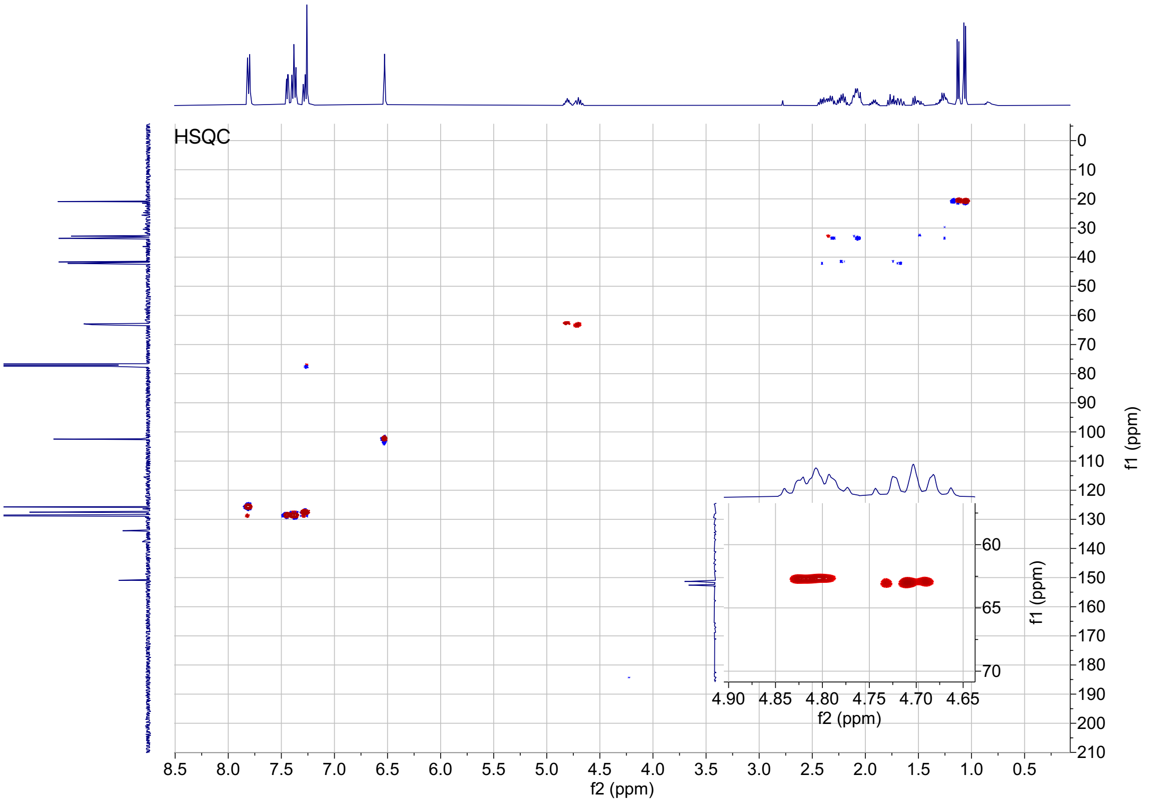

Fig. S23. HSQC spectra of **48** as a mixture of diastereomers.

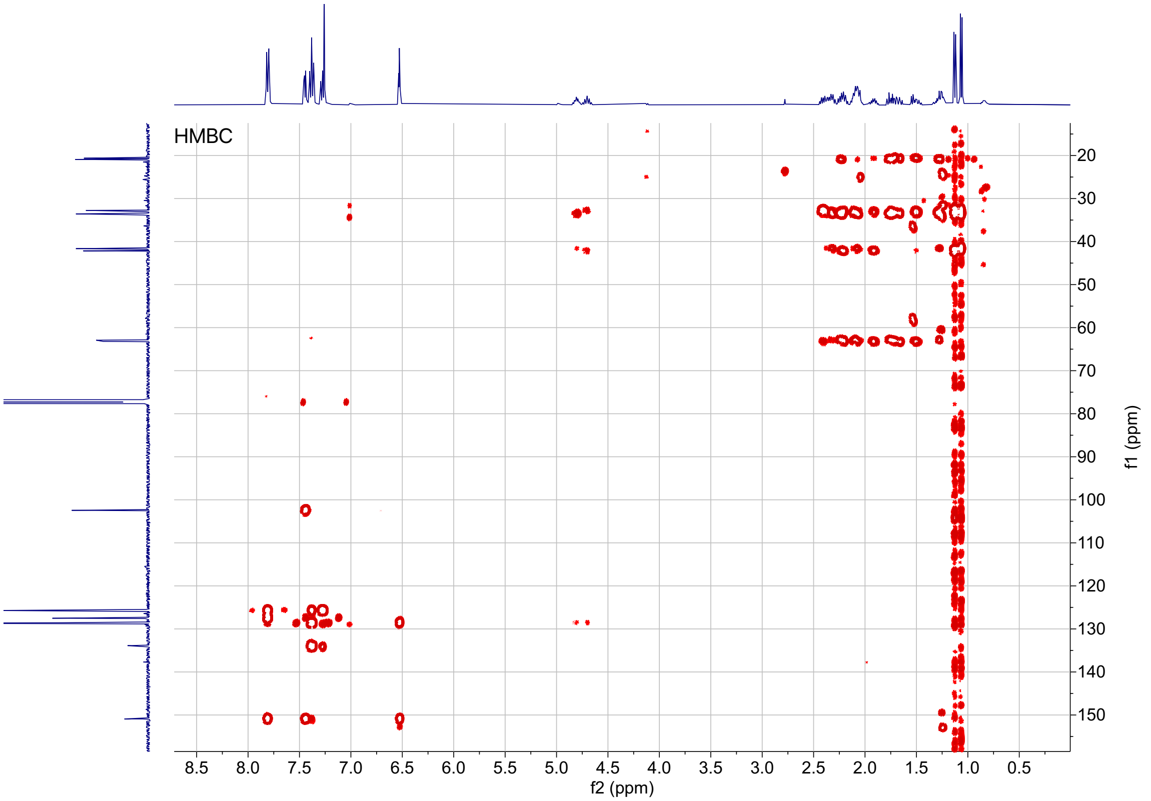

Fig. S24. HMBC spectra of **48** as a mixture of diastereomers.

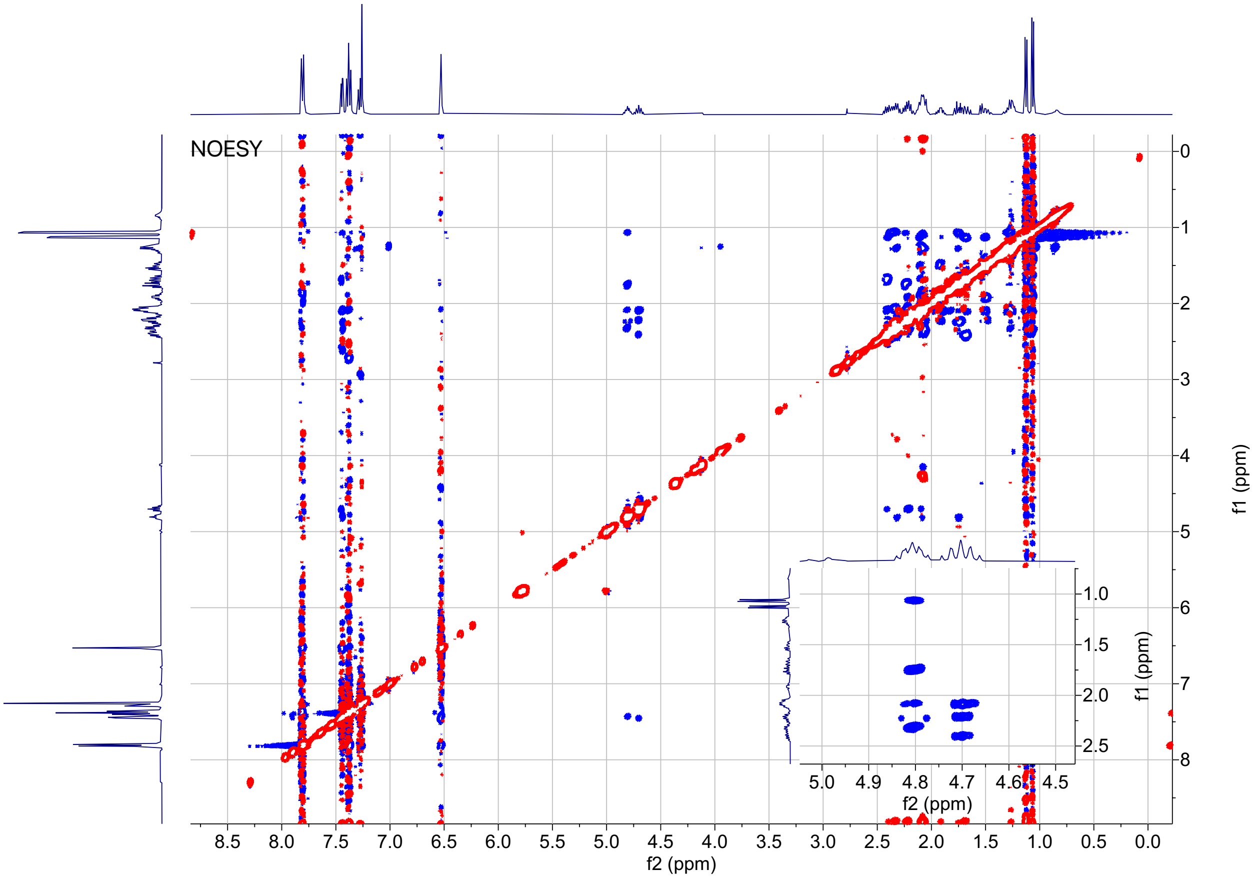

Fig. S25. NOESY spectra of **48** as a mixture of diastereomers.

The terminal methyl protons at 1.6 ppm and the proton at 4.8 ppm correspond to the major isomer. NOESY correlation between these protons indicate the major isomer to be the trans isomer.

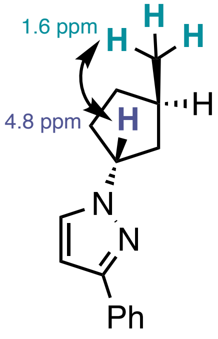

Radical cyclization with diethyl diallylmalonate

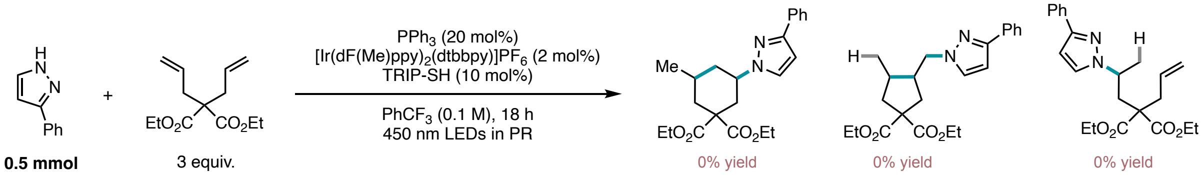

The reaction was prepared according to Procedure A, using 3-phenylpyrazole (14.4 mg, 0.10 mmol, 1.0 equiv.), diethyl diallylmalonate (725 μL, 1.50 mmol, 3.0 equiv.), and triphenylphosphine (26.2 mg, 0.10 mmol, 0.20 equiv.). An aliquot of the crude reaction was taken in $CDCl_3$ and no alkylation products were observed by ^{1}H NMR. All triphenylphosphine was consumed as observed by ^{31}P NMR.

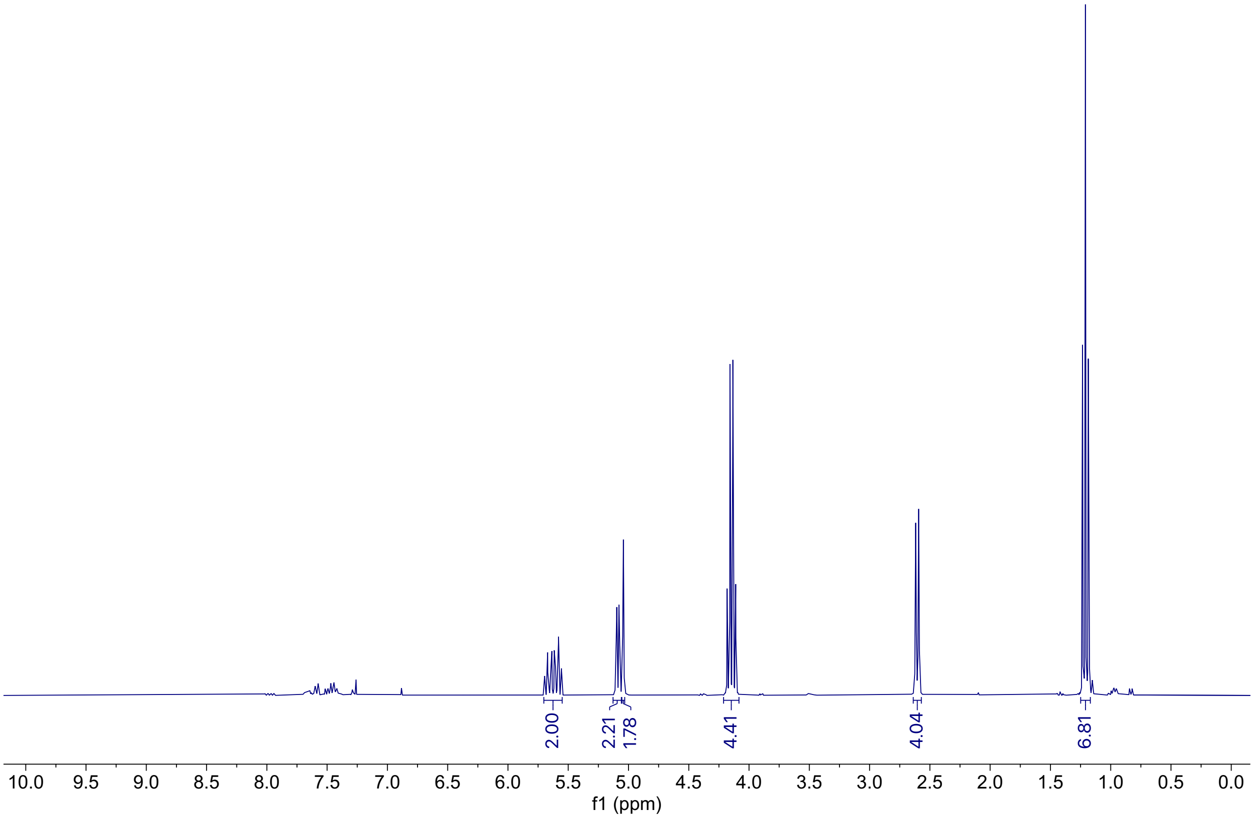

Fig. S26. ^{1}H NMR spectra of crude reaction mixture. Integrations of major peaks correspond to unreacted alkene.

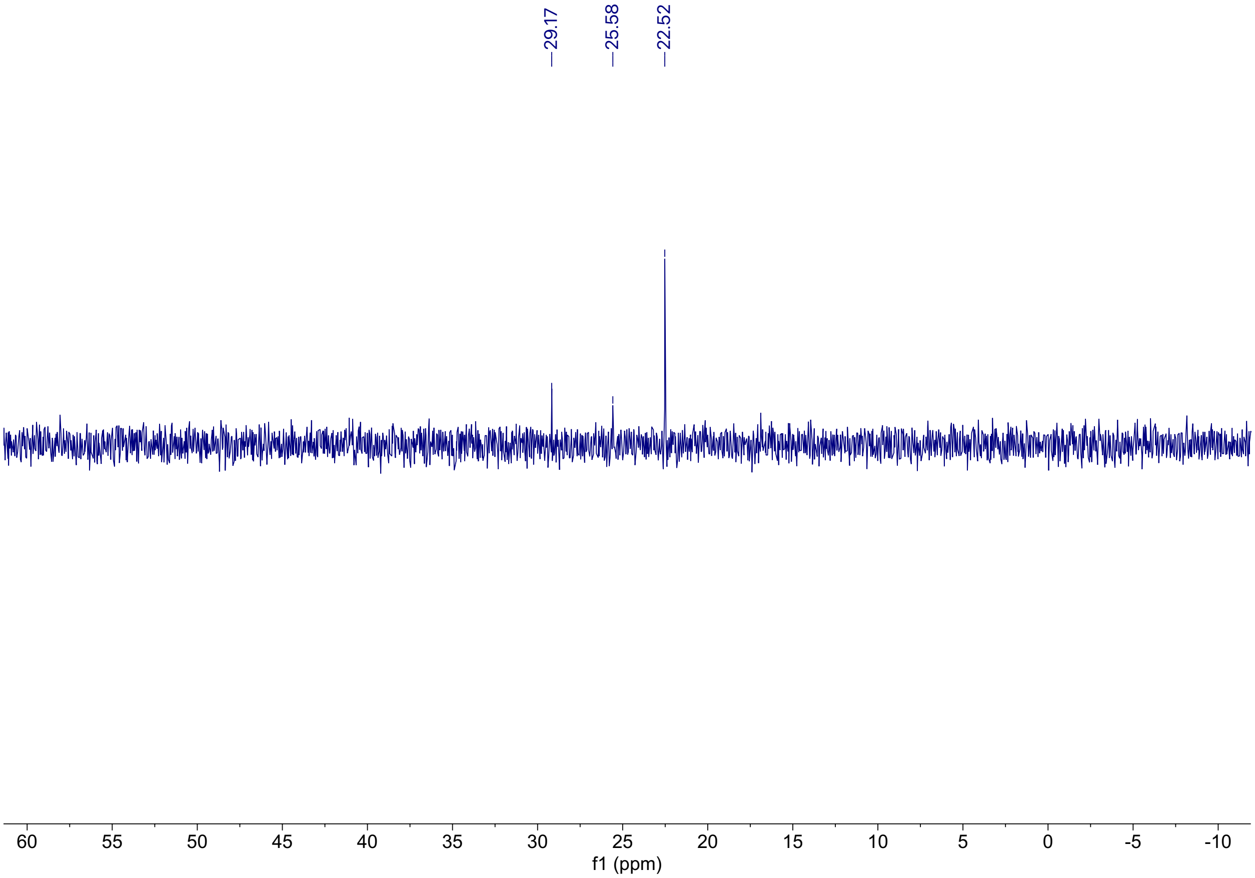

Fig. S27. ^{31}P NMR spectra of crude reaction mixture.

Deuteration Study

Synthesis of deuterated pyrazole

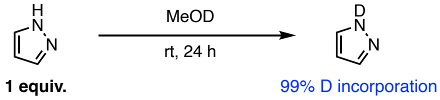

1*H*-pyrazole-1-*d* (A4). In a round bottom flask equipped with a Teflon-coated magnetic stirbar, 1*H*-pyrazole (0.200 g, 1.47 mmol, 1 equiv.) in methanol-*d*4 (0.8 M) was stirred vigorously for 24 hours. The solvent was evaporated and the product collected as a white solid (197 mg, 92% yield). Characterization was in line with previously reported data[10] .

^{1}H NMR (500 MHz, $CDCl_3$): δ 7.63 (d, *J* = 2.1 Hz, 2H), 6.36 (t, *J* = 2.1 Hz, 1H).
^{2}H NMR (77 MHz, $CHCl_3$): δ 9.38 (s, 1H).
$^{13}C\{^{1}H\}$: NMR (126 MHz, $CDCl_3$): δ 133.8, 105.2.

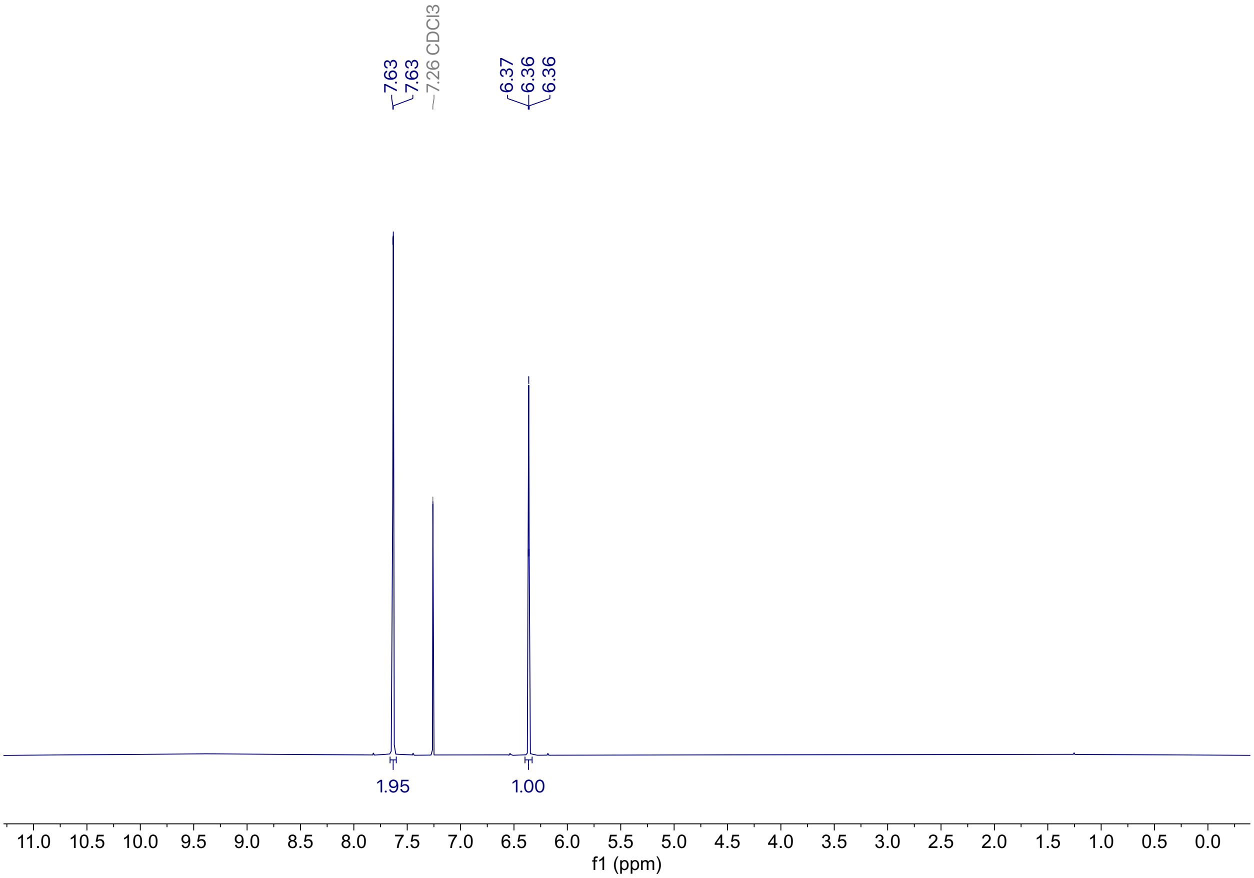

Fig. S28. ^{1}H NMR spectra of **A4**.

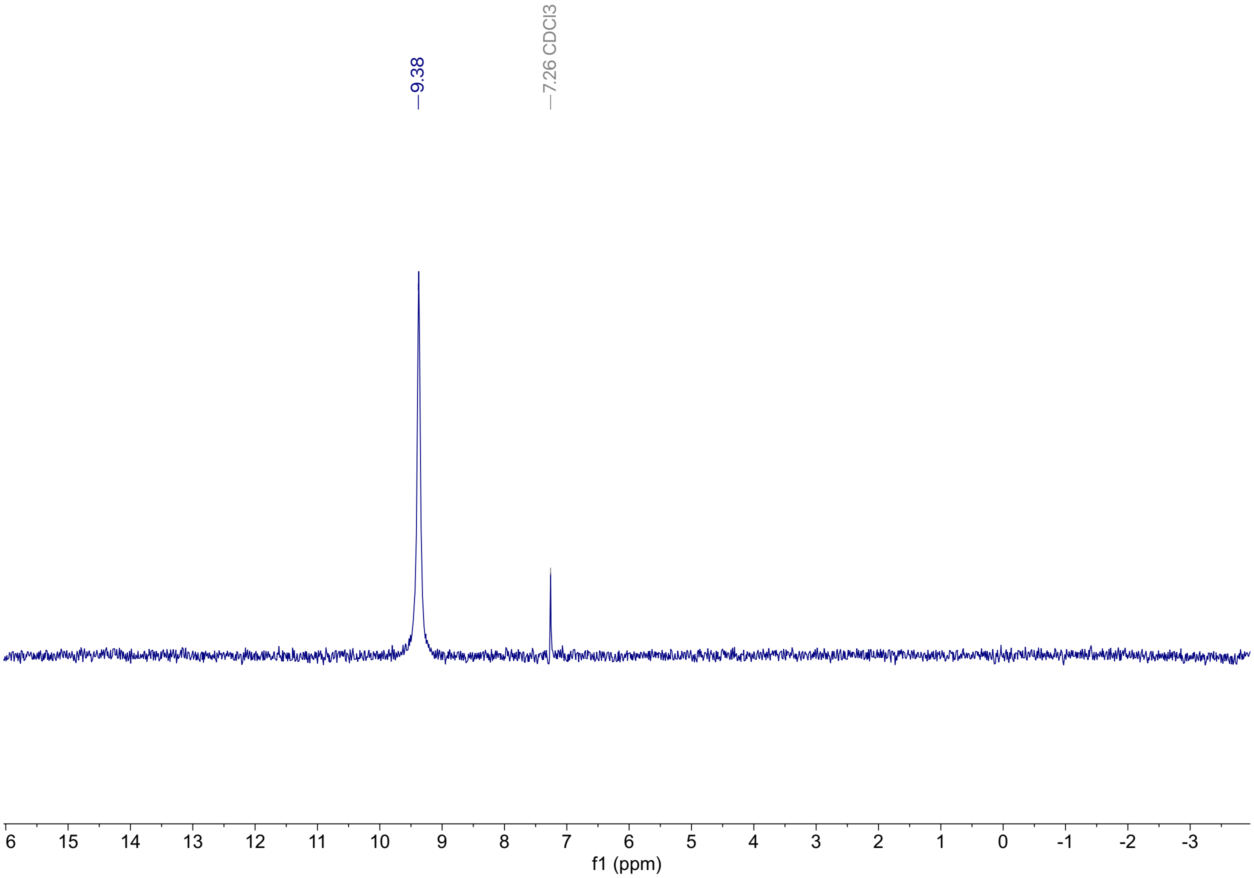

Fig. S29. ²H NMR spectra of **A4**.

Assessing deuterium incorporation

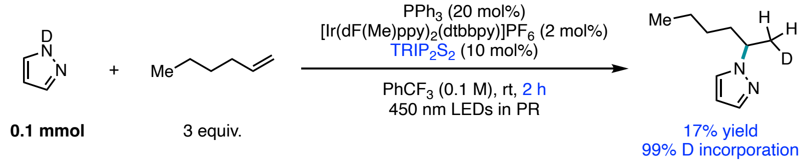

The reaction was prepared according to Procedure A, using deuterated pyrazole and substituting TRIP-SH for $TRIP_2S_2$ to deconvolute possible proton sources. The reaction was removed from irradiation after 2 hours. 1,10-phenanthrene was added (4.6 mg, 0.26 equiv.) and solvent was evaporated. An NMR sample was made of the crude mixture in $CDCl_3$. ^{1}H NMR was used to analyze percent deuteration.

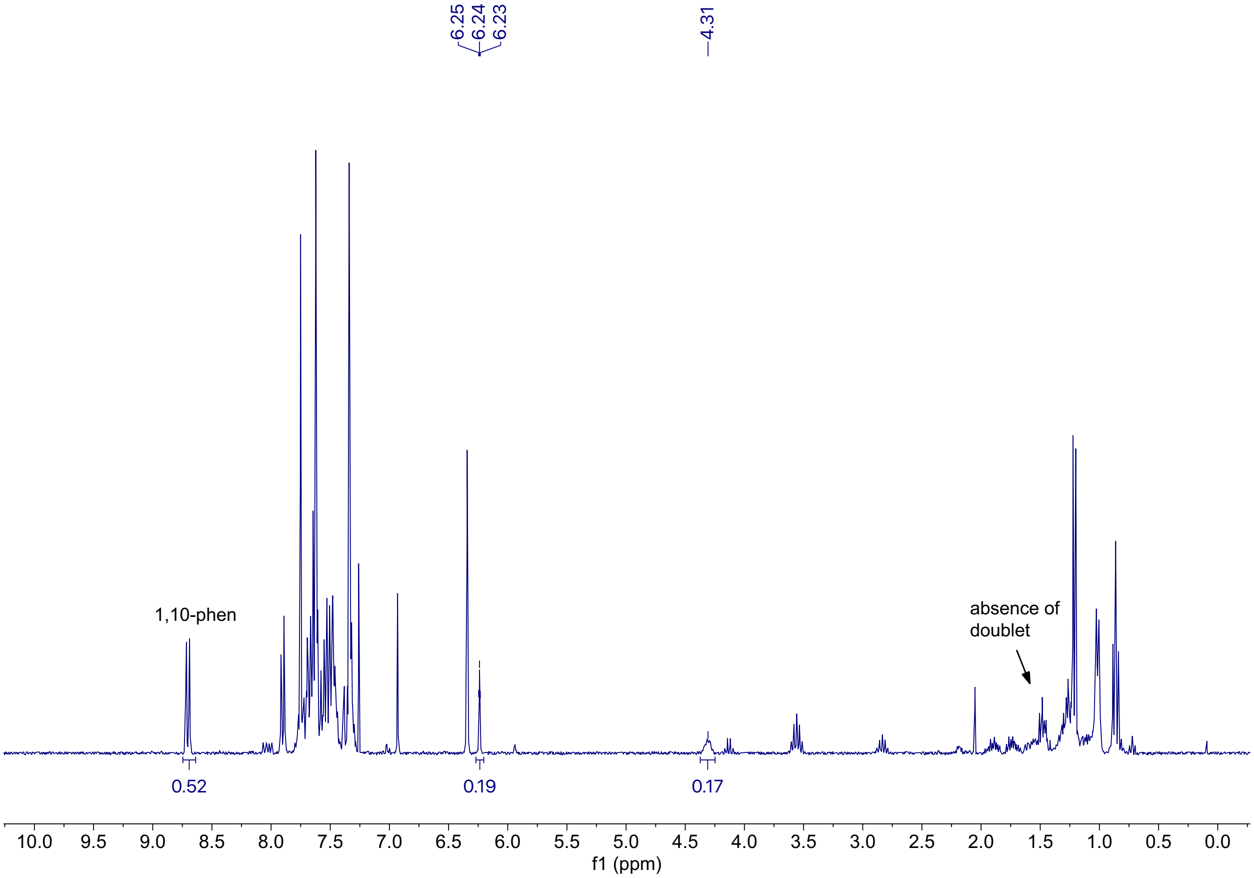

Fig. S30. Crude ^{1}H NMR spectra.

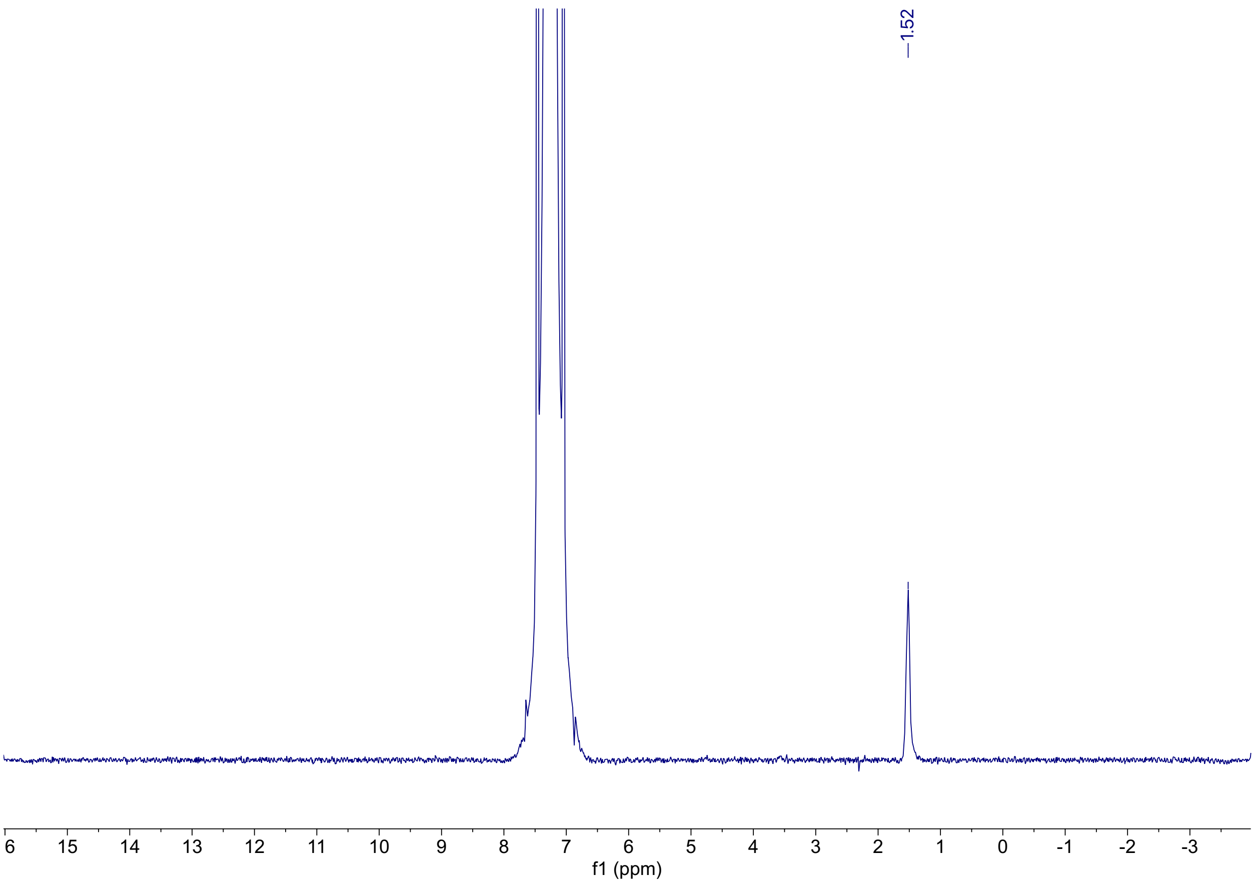

Fig. S31. Crude ^{2}H NMR spectra.

Competition Experiments for Hammett Study

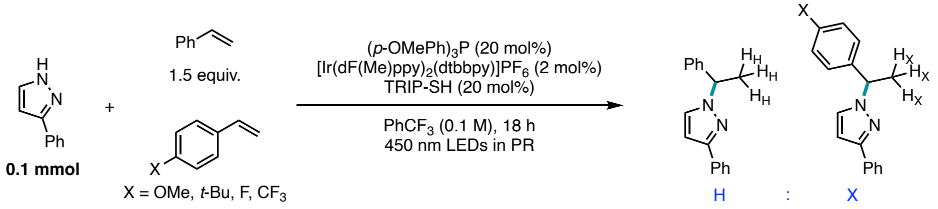

For each competition experiment, the reaction was prepared according to Procedure A, using 3-phenylpyrazole, 1.5 equiv. each of the two styrenes, and 20 mol% TRIP–SH (4.7 μL, 0.2 equiv.) At the end of 18 hours, the vial was removed from the photoreactor. An aliquot was taken in $CDCl_3$ for ^1H NMR and the relative yield determined by taking the ratio of the integrations of the terminal protons H_H and H_X.

Table S49. Ratio of yields.

Substituent	Run 1	Run 2	Run 3	Average
CF_3 to H	0.29	0.22	0.26	0.26
F to H	0.79	0.66	0.75	0.73
H to H	1	1	1	1
t-Bu to H	1.03	0.94	0.98	0.98
OMe to H	1.80	2.10	1.80	1.90

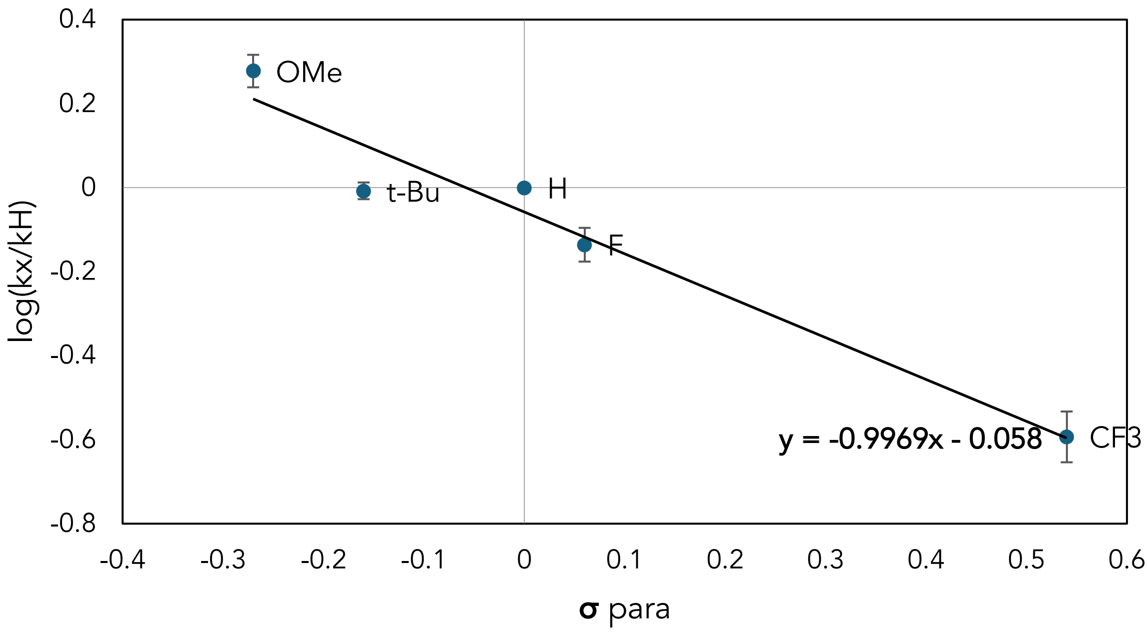

Fig. S32. Hammett plot with σ_p parameters[11].

Correlation with σ radical parameters:

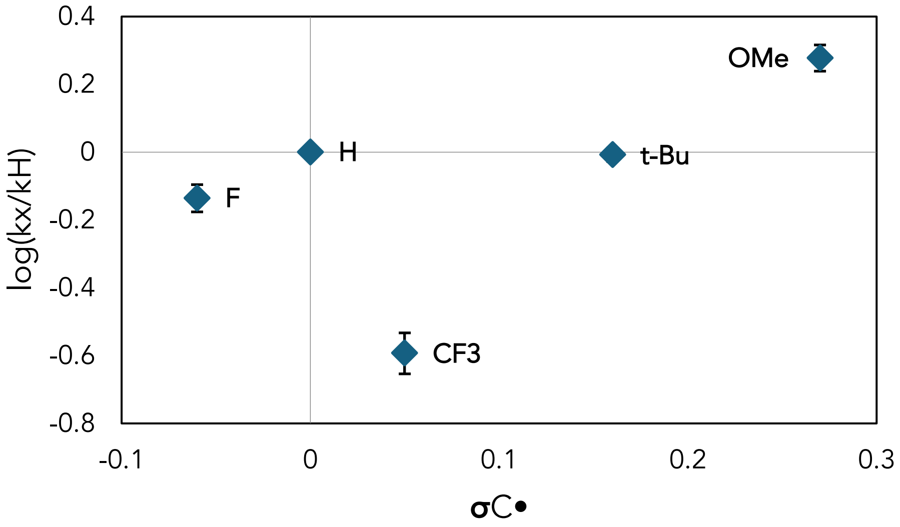

Fig. S33. Hammett plot with σ Creary parameters[12].

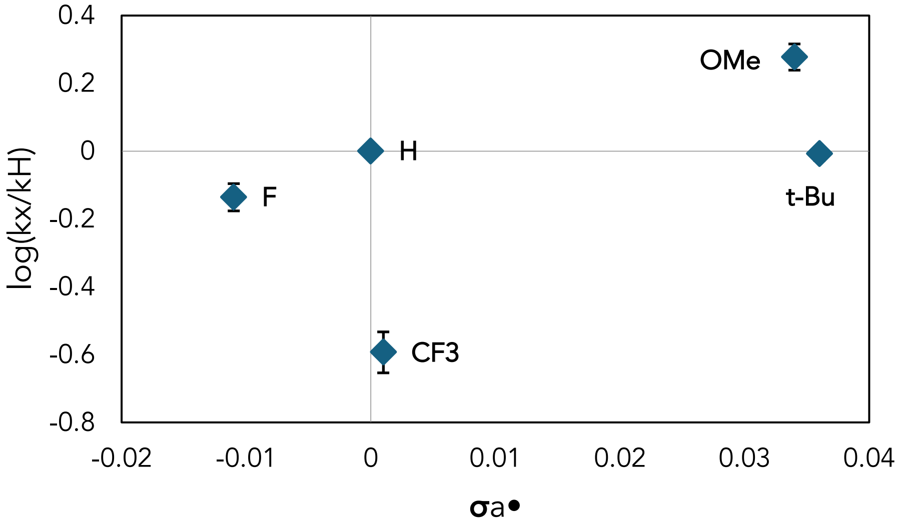

Fig. S34. Hammett plot with σ Arnold parameters[13].

Poor linear correlation is observed with radical parameters, suggesting a polar type of mechanism is operative.

Kinetic Isotope Effect Experiments

Absolute rates experiment

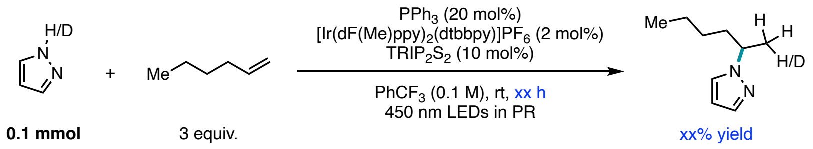

Given that deprotonation is proposed to occur prior to both **TS-1** and **TS-2**, use of a deuterated azole counterpart can provide insight into whether C–N bond formation is the rate-determining step. Initial rates from reactions set up according to Procedure A were approximated by product yield since **Fig. S1** indicates the reaction with pyrazole to progress linearly over time.

Table S50. Initial rates data with 1*H*-pyrazole and 1*D*-pyrazole.

with 1*H*-pyrazole

Time (h)	Run 1	Run 2	Run 3	Average	Std. error
0	0	0	0	0	0
2	5	8	12	8	4
4	18	21	19	19	2
6	36	39	33	36	3
8	39	62	42	48	13

with 1*D*-pyrazole

Time (h)	Run 1	Run 2	Run 3	Average	Std. error
0	0	0	0	0	0
2	8	13	15	12	4
4	18	15	20	18	3
6	37	43	41	40	3
8	47	40	38	42	5

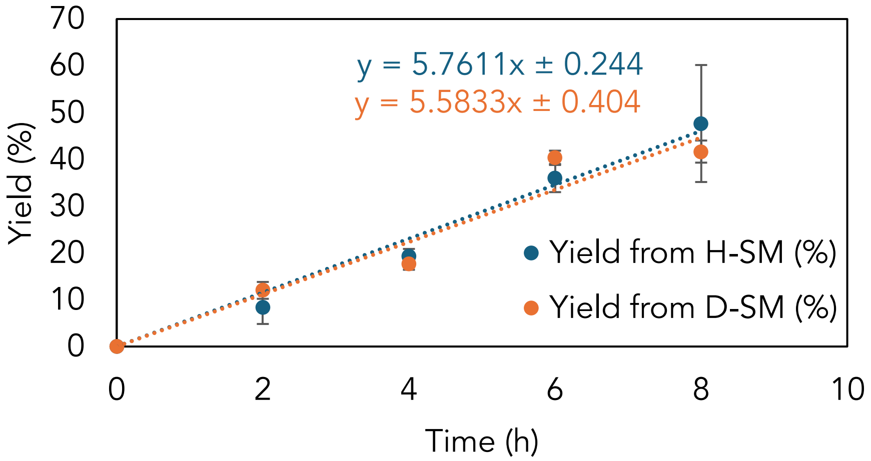

Fig. S35. Time course of 1*H*-pyrazole and 1*D*-pyrazole with 1-hexene.

From Fig. S35, k_H/k_D = 5.7611/5.5833 = 1.032 ± 0.647. Given the error in measurements, we interpret this value as the absence of a KIE, which is in line with our hypothesis that N–H bond-breaking is not involved in the RDS.

Intermolecular competition experiment

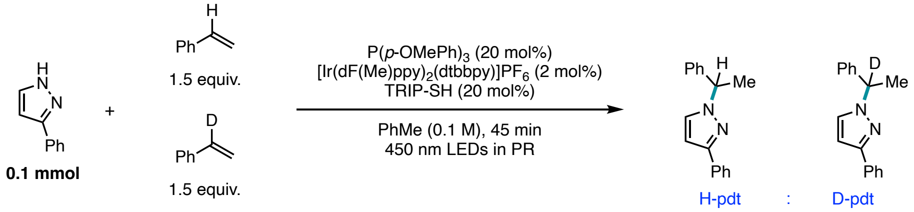

The reaction was set up in triplicate according to Procedure A, with 1,10-phenanthrene used as internal standard instead of 1,3,5-trimethoxybenzene.

$$KIE = \frac{k_H}{k_D} = \frac{\ln(1-F)}{\ln\left[(1-F)\frac{R}{R_0}\right]}$$

F = fractional conversion of protiated SM
R = starting material ratio (SM_D/SM_H) at the end of the reaction
R_0 = starting material ratio (SM_D/SM_H) at the beginning of the reaction

Example calculation for run 1:
Integrals were obtained from ^{1}H NMR spectra of aliquots in $CDCl_3$.

$$F = \frac{1.35}{1.45} = 0.93$$

$$R = \frac{SM_{D(end)}}{SM_{H(end)}} = \frac{1.31}{1.35} = 0.97$$

$$R_0 = \frac{SM_{D(start)}}{SM_{H(start)}} = \frac{1.12}{1} = 1.12$$

$$\frac{k_H}{k_D} = \frac{\ln(1-0.93)}{\ln[(1-0.93)\times\frac{0.97}{1.12}]} = 0.95$$

where

1.45 = actual equivalence of H-styrene added at the beginning of the reaction. Referenced to amount of TRIP-SH added since other components are heterogenous.

1.35 = integral of H-styrene α-proton at end of reaction, with phenanthrene as internal standard

1.31 = average integral of D-styrene β-protons at end of reaction, with phenanthrene as internal standard

1.12 = average integral of D-styrene β-protons at beginning of reaction, when H-styrene α-proton is set to 1

Table S51. KIE values and product yields from individual runs.

	Run 1	Run 2	Run 3	Average KIE
k_H/k_D	0.95	0.95	0.94	0.95
Yield (%) of H-pdt	9	10	12	–
Yield (%) of D-pdt	11	11	13	–

KIE predictions of the C–N bond formation in **TS-1** (for 3-phenylpyrazole with 1-hexene and P(*p*-OMePh)$_3$ as phosphine) and in **TS-2** (for 4-azabenzimidazole (*N1*-alkylation) with 1-hexene and P(*p*-OMePh)$_3$ as phosphine) were performed using KINISOT.py[14], with temperature set to 298.15 K.

Table S52. KIE predictions.

	**KIE**	**corr_KIE**
k_H/k_D in **TS-1** for 3-phenylpyrazole/styrene / P(*p*-OMePh)$_3$	0.931	0.934
k_H/k_D in **TS-1** for 3-phenylpyrazole/1-hexene /P(*p*-OMePh)$_3$	0.777	0.777
k_H/k_D in **TS-2** for 4-azabenzimidazole/ 1-hexene/P(*p*-OMePh)$_3$	0.759	0.760

An inverse secondary deuterium isotope effect is observed in both cases, indicative of a sp^2 to sp^3 change in hybridization that would occur during the proposed C–N bond formation.

Competitive Intramolecular Reactivity

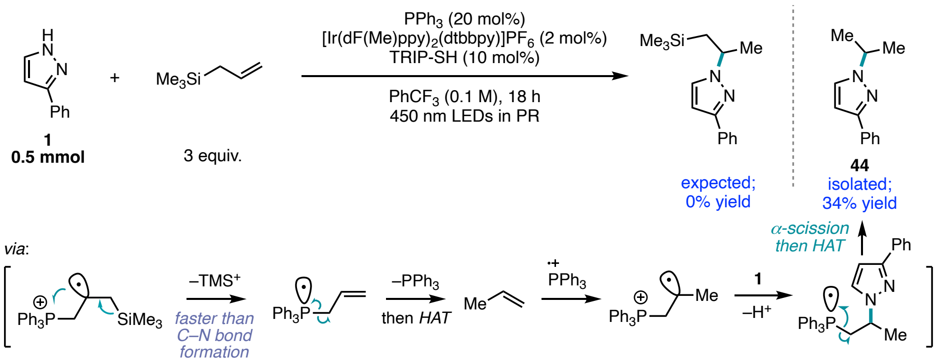

Fig. S36. Scheme showing reaction and proposed mechanism for observed isolation of **44**.

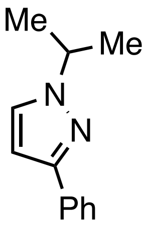

44

1-isopropyl-3-phenyl-1*H*-pyrazole (44): The reaction was set up following general procedure B1 using 3-phenylpyrazole (72.1 mg, 0.500 mmol, 1.00 equiv.), allyltrimethylsilane (238 µL, 1.50 mmol, 3.00 equiv.), and triphenylphosphine (26.2 mg, 0.100 mmol, 0.200 equiv.). The crude product was purified using silica gel flash column chromatography eluting with Et_2O/Hex (0% → 100%, eluted 100%) to give the title compound as a yellow oil (44 mg, 47% yield).

IR (FT-ATR, cm^{-1}, neat): 3064, 3034, 2976, 2932, 2871, 1606, 1497, 1457, 1415, 1385, 1367, 1359, 1343, 1325, 1303, 1282, 1259, 1219, 1176, 1157, 1129, 1105, 1086, 1073, 1046, 1028, 991, 949, 915, 882, 747, 694.
^{1}H NMR (500 MHz, $CDCl_3$): δ 7.84 – 7.78 (m, 2H), 7.45 (d, *J* = 2.3 Hz, 1H), 7.42 – 7.35 (m, 2H), 7.32 – 7.26 (m, 1H), 6.54 (d, *J* = 2.3 Hz, 1H), 4.57 (hept, *J* = 6.7 Hz, 1H), 1.55 (d, *J* = 6.7 Hz, 6H).
^{13}C{^{1}H} NMR (126 MHz, $CDCl_3$): δ 150.8, 134.0, 128.7, 127.5, 127.4, 125.8, 102.4, 54.0, 23.2.
HRMS (APCI): Exact Mass calculated for $[C_{12}H_{14}N_2 + H]^+$ requires *m/z* = 187.1230. Found 187.1230.

To detect the formation of propene, as would be generated in the proposed mechanism above, the reaction was prepared according to Procedure A, omitting the stir bar and using toluene-*d*8 as the solvent, with no azole nucleophile. The reaction was then transferred to a J-Young tube and sealed. The J-Young tube was placed 2 cm away from a 427 nm PR160L Kessil Lamp, and was irradiated at 100% intensity for 18 h. The tube was inverted to mix the reaction every 30 mins for the first and last two hours. The J-Young tube was directly subject to NMR analysis. Representative peaks indicate formation of propene.

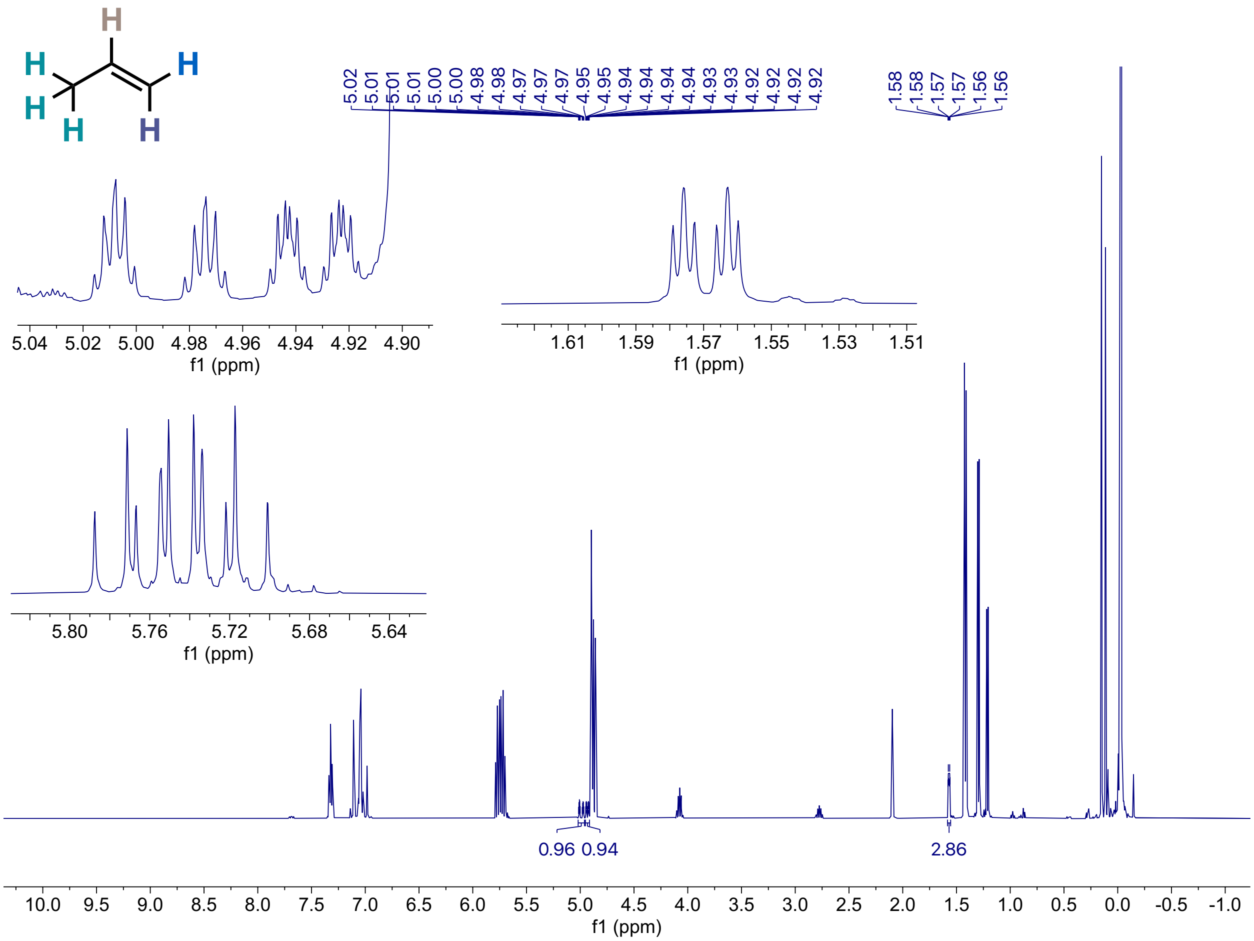

Fig. S37. ^{1}H NMR spectra of reaction mixture in J-Young tube.

Cyclic Voltammetry

Cyclic voltammetry (CV) experiments were obtained with a Gamry Interface 1010 Potentiostat/Galvanostat/ZRA instrument and processed using Gamry Echem FrameworkTM and AnalystTM software (working electrode: glassy carbon; reference electrode: Ag/Ag+; counter electrode: Pt wire; scan rate: 0.1 V/s; sample concentration: 1 mM). All measurements were taken in degassed MeCN with NBu_4PF_6 (0.1 M) as electrolyte at 298 K. Ground state reduction potentials (E_{red}) were identified as half of the absolute maximum current value during the reduction event. Ferrocene (Fc) was used as an internal standard or an external standard. When Fc was used as an internal standard, ferrocene was added into the sample solution and one CV was performed. When Fc was used as an external standard, cyclic voltammetry (CV) was performed with ferrocene only under the same experimental conditions, and its $E_{1/2}$ (vs. Ag/AgCl) was recorded. $E_{1/2}$ = 0.42 V (vs. SCE) for Fc/Fc+ is used for conversion.

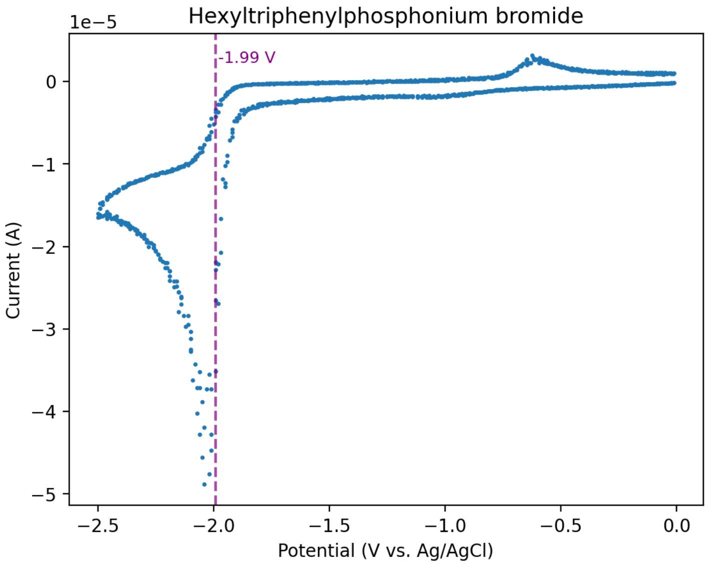

Fig. S38. Cyclic voltammogram of **B2** in MeCN.

$$E_{1/2}(P^+/P^\bullet) = -2.41 \text{ V vs. SCE}$$

Given $E_{1/2}(P^+/P^\bullet)$ is more negative than $E(Ir^{II})_{1/2\ Red} = -1.43$ V and $E(Ir^{III*})_{1/2\ Red} = -0.92$ V for $[Ir(dF(Me)ppy)_2dtbbpy]PF_6$[15], the phosphonium is unlikely to be reduced to the corresponding phosphoranyl radical under the standard conditions.

UV-Vis Study

This study was performed to evaluate potential electron-donor acceptor (EDA) complex formation between a distonic radical cation with 1-hexene and N–H azole as an alternative mechanism for electron transfer. Since the distonic radical cation is not an isolable species, we employed the corresponding phosphonium bromide salt **B2** as a substitute.

The following stock solutions were prepared:

A. 20 µM solution of 3-phenylpyrazole in MeCN
B. 5 µM solution of hexyltriphenylphosphonium bromide in MeCN

Ratios of these solutions were added separately to oven-dried 1 cm quartz cuvettes to make the following samples, with a total volume of 3 mL:

- 50% A + 50% MeCN
- 50% B + 50% MeCN
- 50% A + 50% B

The cuvettes were sealed and subjected to UV-vis spectroscopy.

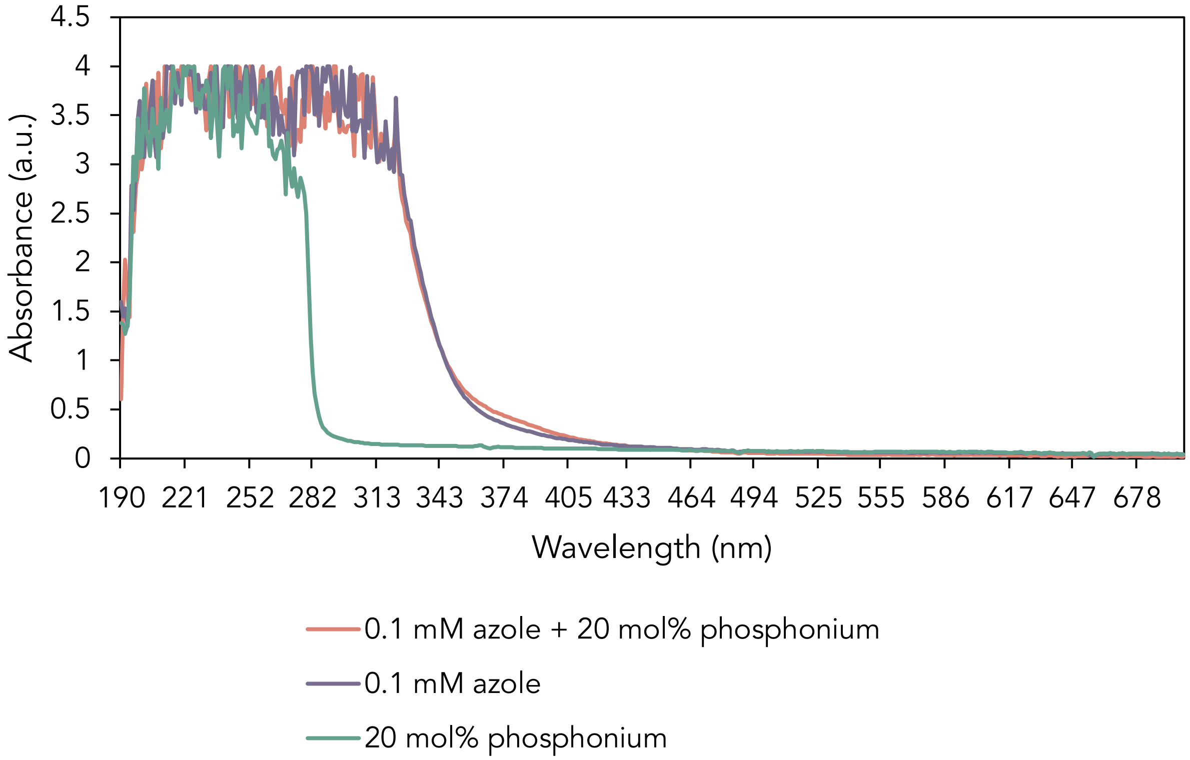

Fig. S39. UV-vis spectrum of various concentrations of hexyltriphenylphosphonium bromide **B2** and 3-phenylpyrazole.

Given the overlap in absorbance for 1:0.2 Azole:Phosphonium compared to the two reagents separately, the formation of an EDA complex between the azole and the phosphonium in situ is unlikely.

Internal Alkenes as Substrates

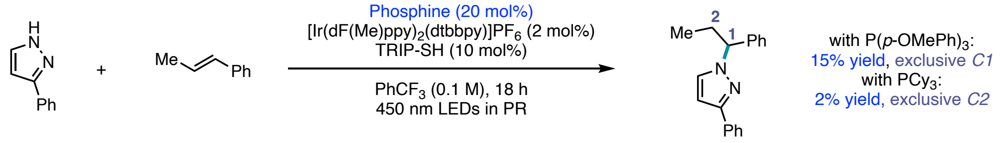

A 1,2-disubstituted substrate such as β-methylstyrene is largely unreactive under both anti-Markovnikov conditions (PCy_3 as phosphine catalyst) and Markovnikov conditions (P(*p*-OMePh)$_3$ as phosphine catalyst), likely because styrenes and terminal alkenes are unreactive under the anti-Markovnikov conditions and internal alkenes are sterically congested for this Markovnikov chemistry. However, we observe *N*-alkylation α- to the styrene when P(*p*-OMePh)$_3$ is used as catalyst, and *N*-alkylation β- to the styrene when PCy_3 is used as catalyst. These results are consistent with the more "anti-Markovnikov" product arising from NCR into the β-carbon to give

the more stabilized benzylic radical for HAT, and the "Markovnikov" product being α-substitution to the styrene.

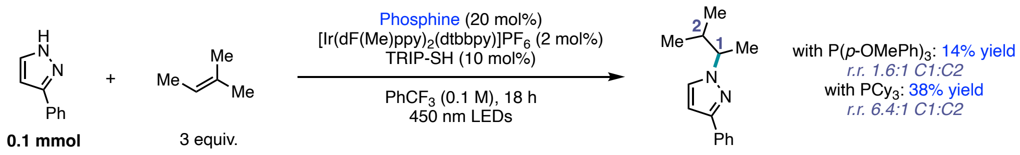

Consistent with our proposal for nucleophilic amination vs. NCR addition into the alkene for either Markovnikov or anti-Markovnikov regioselectivity, respectively, for these substrates, given the steric bias against nucleophilic amination, the reactivity is diverted towards the anti-Markovnikov pathway. If NCR addition is kinetically tractable with that alkene, it should favor forming anti-Markovnikov product. If NCR addition is too kinetically uphill, no reactivity should be observed, consistent with internal alkenes being less competent substrates overall. Consistent with this explanation, when PCy_3 is employed as phosphine catalyst, the reaction above gives a higher product and regioisomeric ratio of anti-Markovnikov product.[1]

Unsuccessful Substrates

These substrates were screened following Procedure A and (unless otherwise stated) gave no ^{1}H NMR yield with comparison to an internal standard of 1,3,5-trimethoxybenzene. Incompatibilities likely result from substitution that present steric hindrance and/or functional group intolerance. Electronically activated alkenes are also less compatible than unactivated alkenes under the general conditions.

N-Nucleophiles

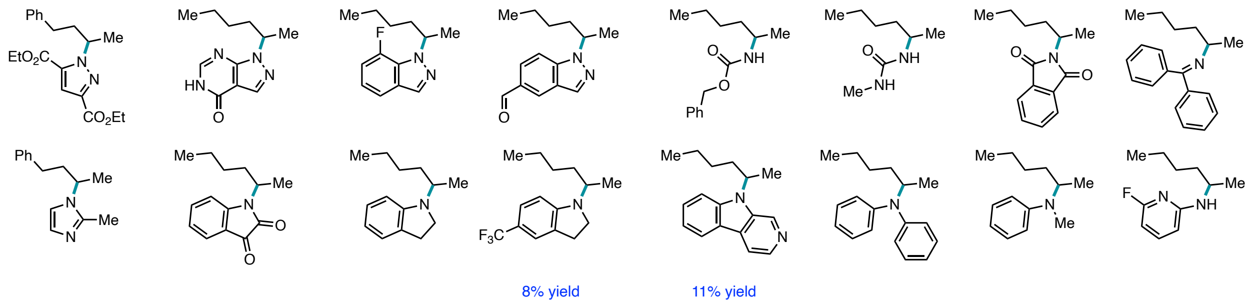

Alkenes

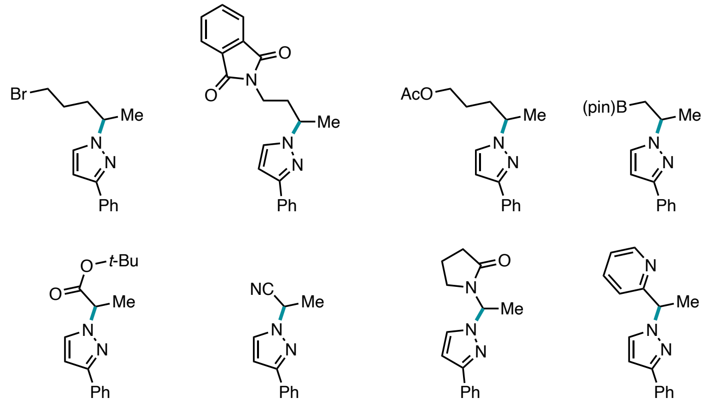

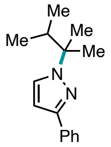

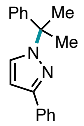

10% yield

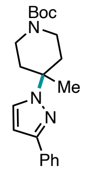

12% yield

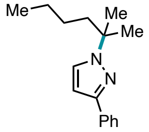

21% yield

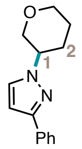

15% yield,
r.r. 2:1 C1:C2

10. DFT Studies

General Considerations

All Density Functional Theory (DFT) calculations were performed using the Gaussian16 rev. A.03 software package[16]. Unless specified otherwise, geometry optimization calculations were performed using the Minnesota hybrid meta-GGA M06-2X[17] density functional paired with Ahlrichs's split valence double-ζ, def2-SVP[18,19], basis set on all atoms. The geometries of all open-shell species were optimized with spin-unrestricted orbitals with the (U)M06-2X functional and def2-SVP basis set on all atoms. Intermediates **INT-4D(')** and **INT-6A(')** were subject to meta-dynamics conformational sampling using CREST[20] version 3.0 at the GFN2-xTB level of theory. An energetic cutoff of 5.0 kcal/mol was employed to filter the conformers, followed by DFT-level optimization using the (U)M06-2X[17] functional paired with the def2-SVP[18,19] basis set on all atoms. Harmonic vibrational frequencies were computed at the geometry optimization level of theory to verify that stationary points were either minima or first-order saddle points on the potential energy surface. Single-point energies of the optimized structures were calculated using the (U)M06-2X functional, all with the SMD solvation model for toluene[21,22] and the def2-TZVP basis set. Single-point energies of the optimized structures were calculated using the (U)M06-2X functional, while employing the def2-TZVP basis set[23].To correct for solvation, the SMD implicit solvation model was employed with toluene to simulate the solvent reaction field in the solution state[21,22]. Final Gibbs free energies were obtained by applying thermodynamic corrections obtained at the optimization level of theory to the refined electronic energies. These corrections were performed using Paton's *GoodVibes* package at 298.15 K and 1 atm, with a vibrational normal mode frequency cutoff of 100 cm^{-1} [24–26]. A standard state of 1 M is assumed for all species. Natural Bond Orbital (NBO) calculations were performed within the *Gaussian16* implementation of the NBO version 3.1 package at the (U)M06-2X level of theory[27]. Electronic energies for Distortion-Interaction Analysis[28] were calculated using the (U)M06-2X functional and the def2-TZVP basis set. Molecular structure graphics were rendered in CYLview[29], PyMOL[30] , and Chimera[31] .

DFT coordinates and CYLview structures are attached in the folder 'xyz_coords'.
Electronic and free energies for computed structures and single point energy calculations are reported in Hartrees and can be found in the Excel sheet 'energies_SI.xlsx'.

<u>NCR Addition to 1-Hexene</u>

The results for nitrogen-centered radical (NCR) addition to 1-hexene with either Markovnikov or anti-Markovnikov regioselectivity are summarized in Figs. S28 to S30. Since the Markovnikov-selective addition is calculated to be disfavored over anti-Markovnikov-selective addition by $\Delta\Delta G^{\ddagger}$ = 1.7 kcal/mol, this result suggests that NCR addition is not a productive pathway towards the observed Markovnikov regioselectivity.

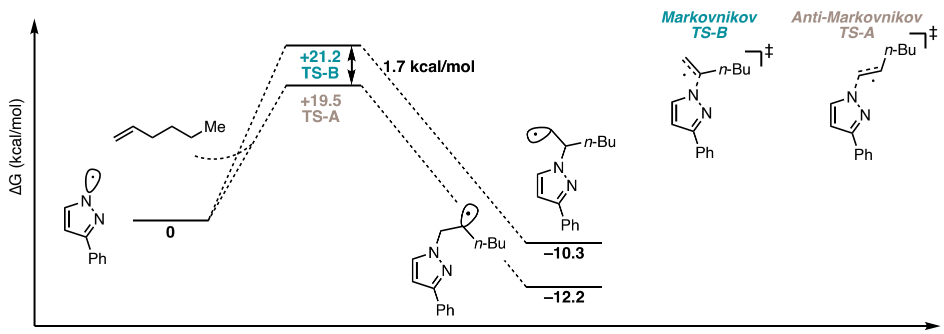

Fig. S40. Regioselective outcomes for addition of 3-phenylpyrazole NCR to 1-hexene.

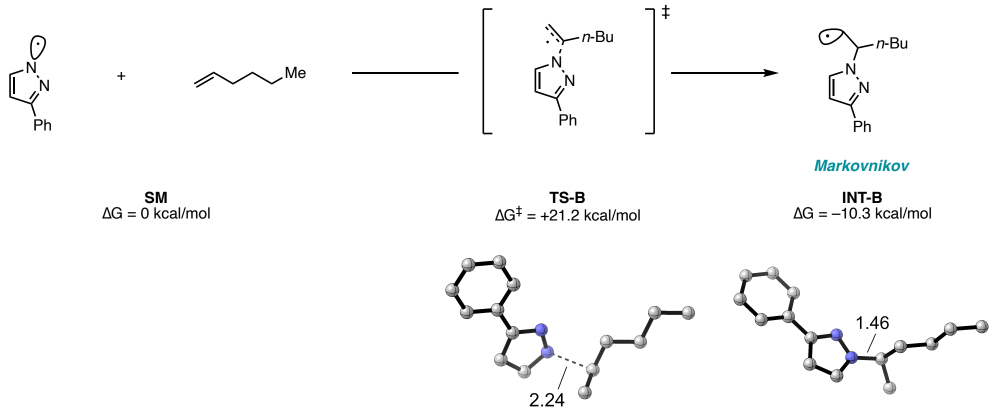

Fig. S41. Markovnikov addition of 3-phenylpyrazole NCR to 1-hexene. CYLview: Hydrogens omitted for clarity.

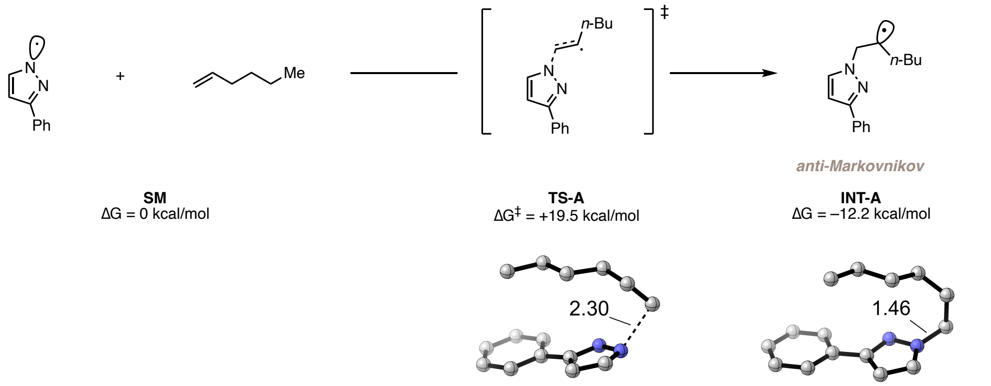

Fig. S42. Anti-Markovnikov addition of 3-phenylpyrazole NCR to 1-hexene. CYLview: Hydrogens omitted for clarity.

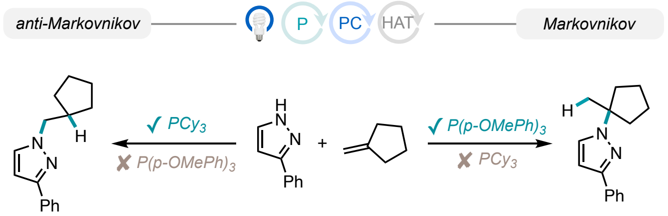

Fig. S43. Observation of opposite regioselectivity from exclusively phosphine catalyst switch using 3-phenylpyrazole as N–H azole and methylene cyclopentane (MCP) as alkene partner.

MCP is an alkene that is reactive when either PCy_3 or P(*p*-OMePh)$_3$ is employed as the phosphine catalyst, resulting in the anti-Markovnikov product under PCy_3-catalyzed conditions and Markovnikov product under P(*p*-OMePh)$_3$-catalyzed conditions (Fig. S43). Terminal alkenes are unreactive with PCy_3 as phosphine catalyst. Therefore, we calculated the proposed pathways for Markovnikov selectivity using methylene cyclopentane (MCP) as the alkene (Fig. S44). Both pathways (i) and (ii) to **INT-6A** are calculated to be kinetically tractable and thermodynamically downhill, followed by subsequent α-scission to **INT-7A**. NBO charge and spin density comparison of the P center and C radical from **INT-9** to **TS-1** and **INT-4D** to **TS-2**, respectively, reveal similar change in values for both pathways (Fig. S45). Fig. S46 shows the energy surface for the preferred anti-Markovnikov NCR addition to MCP if the regioselective step is agnostic to phosphine identity. This process is higher in energy than either nucleophilic amination pathway, suggesting the identity of the phosphine to be crucial for Markovnikov selectivity.

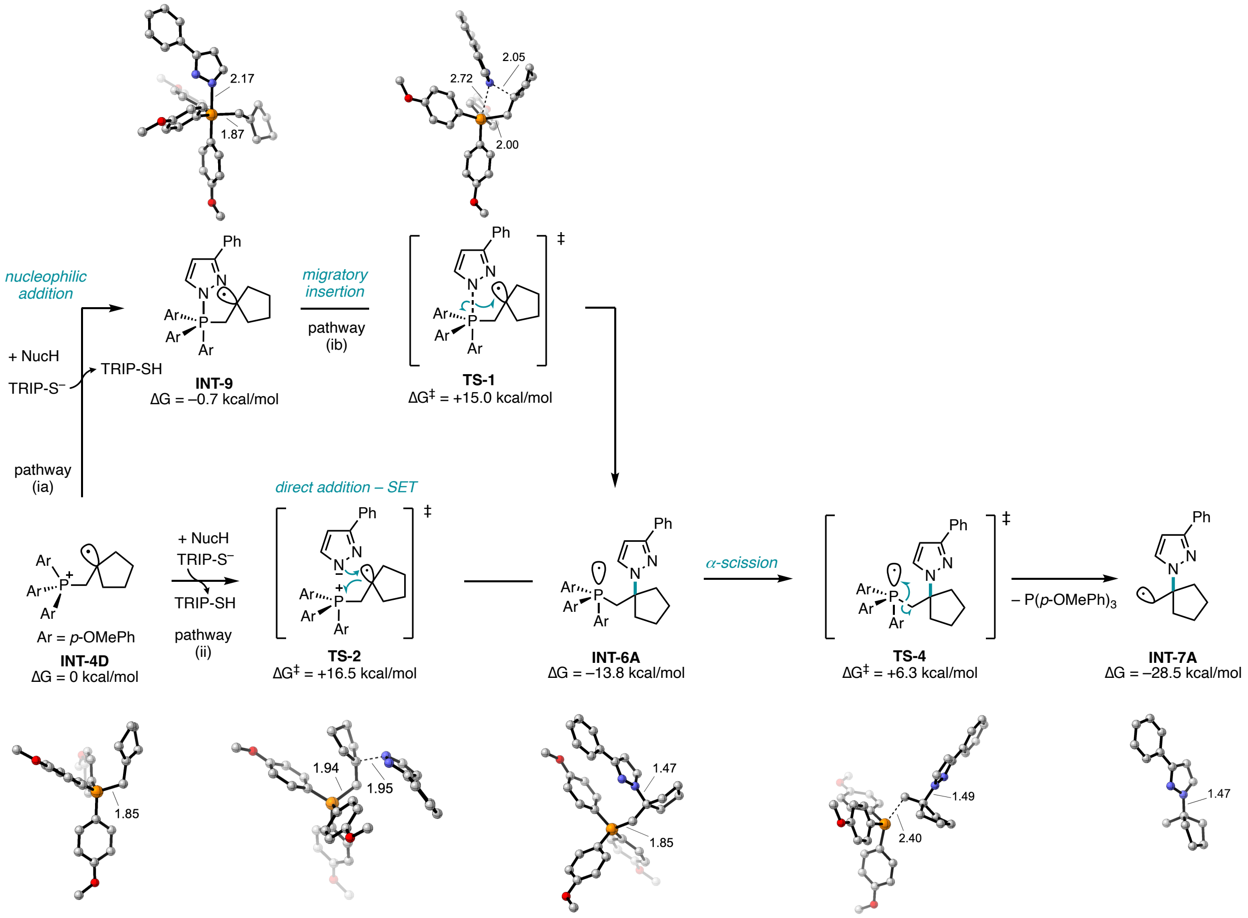

Fig. S44. Top: proposed mechanism for Markovnikov-selective hydroamination via **TS-1**, bottom: proposed mechanism for Markovnikov-selective hydroamination via **TS-2**. CYLview: Hydrogens omitted for clarity.

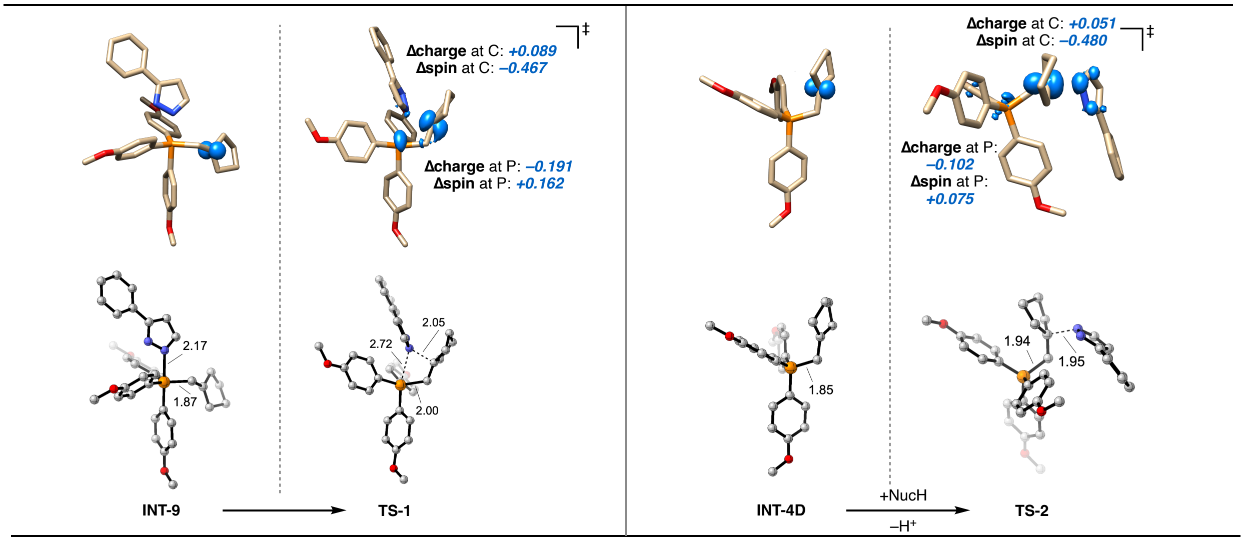

Fig. S45. Left, top: spin density difference plots for pentavalent phosphorane **INT-9** (left) and C–N bond formation transition state **TS-1** (right); left, bottom: corresponding structures with bond lengths in Angstroms. Right, top: spin density difference plots for distonic radical cation **INT-4D** (left) and C–N bond formation transition state **TS-2** (right); right, bottom: corresponding structures with bond lengths in Angstroms. NBO charge and spin are labelled for relevant atoms. CYLview and Chimera: Hydrogens omitted for clarity.

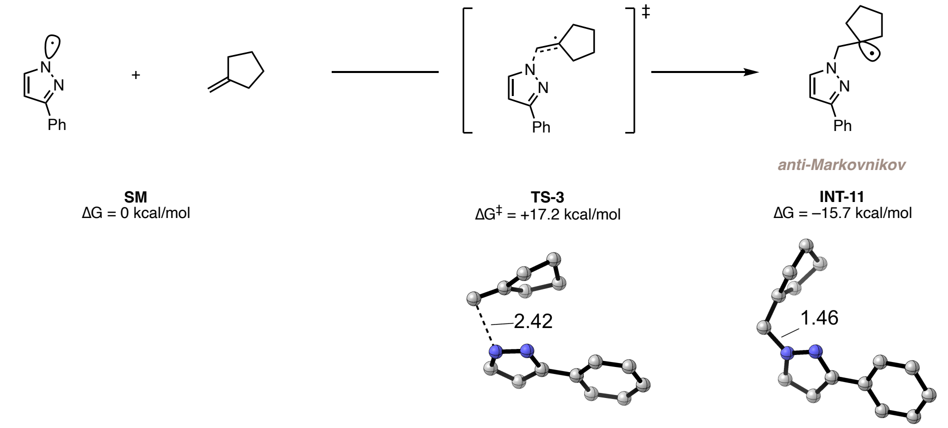

Fig. S46. Anti-Markovnikov addition of 3-phenylpyrazole NCR to methylene cyclopentane. CYLview: Hydrogens omitted for clarity.

N-site Selectivity

The energy surfaces under the mechanistic hypothesis of pathway (ii) for the *N*-site selectivity observed with substrate **14** are summarized in Fig. S33. The $\Delta\Delta G^{\ddagger}$ = 4.9 kcal/mol between *N1* vs. *N3* N–C bond formation is consistent with obtaining exclusive *N1*-site selectivity. Favorable non-covalent interactions between the substrate and the aryl rings of the phosphine may stabilize

the transition state to a greater extent when the *N1* nitrogen forms the C–N bond, as supported by the more negative $\Delta E^{\ddagger}_{int}$ from Distortion-Interaction Analysis (Table S52). However, less catalyst (**INT-4D**) distortion in **TS-2-N1** has a more significant effect. A conformational difference between **TS-2-N3** and **TS-2-N1** is the shorter distance between the P center and C atom undergoing bond formation in the latter, suggesting this TS complex may facilitate faster intramolecular SET to **INT-6B** with C–N bond formation.

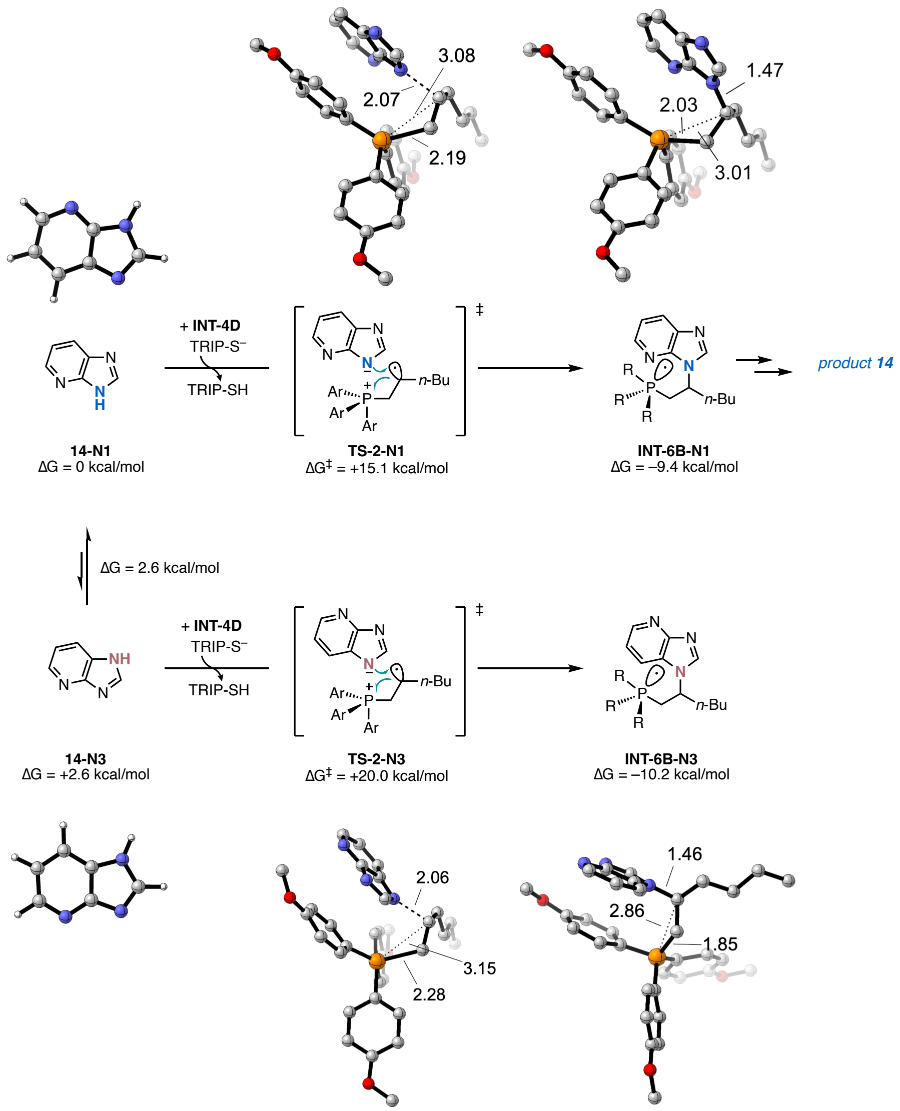

Fig. S47. *N*-site regioselectivity outcomes for 4-azabenzimidazole under the mechanistic proposal for Markovnikov-selective C–N bond formation. CYLview: Hydrogens omitted for clarity.

Structure	$\Delta E^{‡}_{distort}$	$\Delta E^{‡}_{distort\text{-}cat}$	$\Delta E^{‡}_{distort\text{-}sub}$	$\Delta E^{‡}_{int}$
TS-2-N1	24.8	24.1	0.7	-82.2
TS-2-N3	29.5	28.2	1.3	-81.0

Cat = distonic radical cation
Sub = azole
Table S53. Distortion-Interaction Analysis of TS-2-N1 and TS-2-N3. (Energies in kcal/mol).

TS-2	E‡(TS)	E‡(cat_TS)	E‡(sub_TS)	E(cat_ground)	E(sub_ground)
N1	-2010.859878	-1615.383106	-395.345825	-1615.421577	-395.346918
N3	-2010.850604	-1615.37669	-395.344767		

Table S54. Electronic energies for Distortion-Interaction Analysis. (Energies in Hartrees)

Equations:

Equation 1: $\Delta E^{\ddagger}(distort\_cat) = [E^{\ddagger}(cat\_TS) - E(cat\_ground)] * 627.509$

Equation 2: $\Delta E^{\ddagger}(distort\_sub) = [E^{\ddagger}(sub\_TS) - E(sub\_ground)] * 627.509$

Equation 3: $\Delta E^{\ddagger}(distort) = \Delta E^{\ddagger}(distort\_cat) + \Delta E^{\ddagger}(distort\_sub)$

Equation 4: $\Delta E^{\ddagger}(int) = [E^{\ddagger}(cat\_TS) + E^{\ddagger}(sub\_TS) - E^{\ddagger}(TS)] * 627.509$

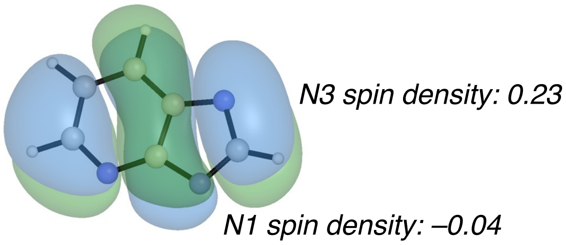

Fig. S48. SOMO for 4-azabenzimidazole nitrogen-centered radical (NCR). Greater NBO spin density on *N3* nitrogen supports the observed major *N3*-site selectivity under reaction conditions in which NCR addition to the alkene is proposed to be rate-determining[1].

<u>Comparison of C–N Bond Formation between Phosphines</u>

The energy surface of C–N bond formation under the mechanistic hypothesis of pathway (ib) when PCy_3 is used as the phosphine catalyst and methylene cyclopentane as the alkene is summarized in Fig. S49. The energy barrier of $\Delta G^{\ddagger} = 21.1$ kcal/mol for this step is significantly higher than both when P(*p*-OMePh)$_3$ is used as the phosphine catalyst (Markovnikov selectivity observed, $\Delta G^{\ddagger} = 15.0$ kcal/mol, Fig. S44) and for NCR addition to MCP (anti-Markovnikov selectivity observed with PCy_3, $\Delta G^{\ddagger} = 17.2$ kcal/mol, Fig. S46). Thus, when PCy_3 is used as the

phosphine catalyst, NCR addition to the alkene will be the rate and selectivity-determining step, consistent with the experimental observation of anti-Markovnikov selectivity under these conditions. NBO charge and spin evaluations from **INT-4D′** to **TS-2′** (Fig. S50) are consistent with the proposed mechanism for nucleophilic amination if PCy_3 did facilitate reactivity. Distortion-Interaction Analysis with regards to **TS-1** and **TS-2′**, with the caveat that this is not a head-to-head comparison given the different phosphines, suggests slightly greater substrate (3-phenyl pyrazole anion) distortion and reduced interaction ($\Delta E^{\ddagger}_{int}$) in **TS-2′**.

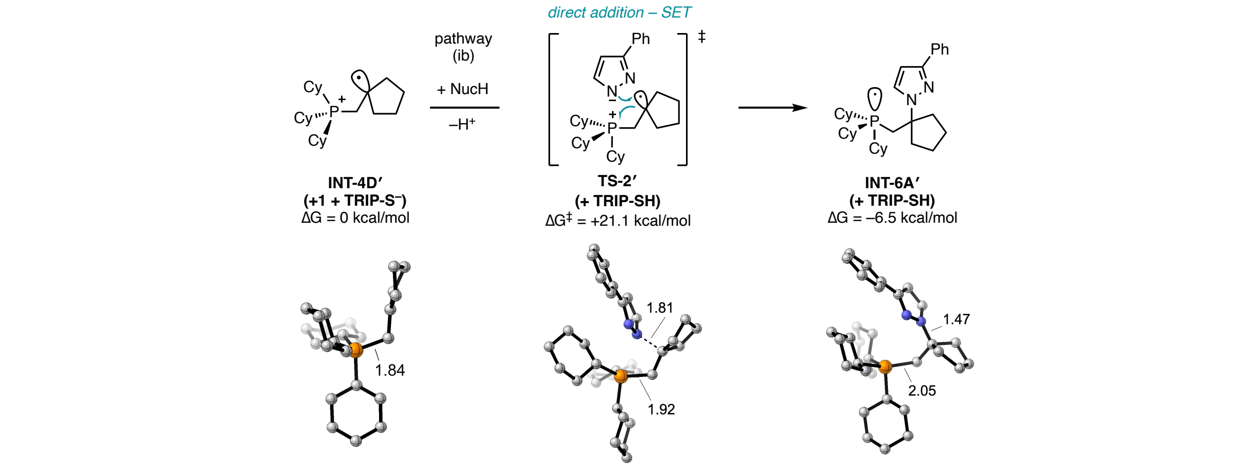

Fig. S49. Markovnikov C–N bond formation with PCy_3 (not favored). CYLview: Hydrogens omitted for clarity.

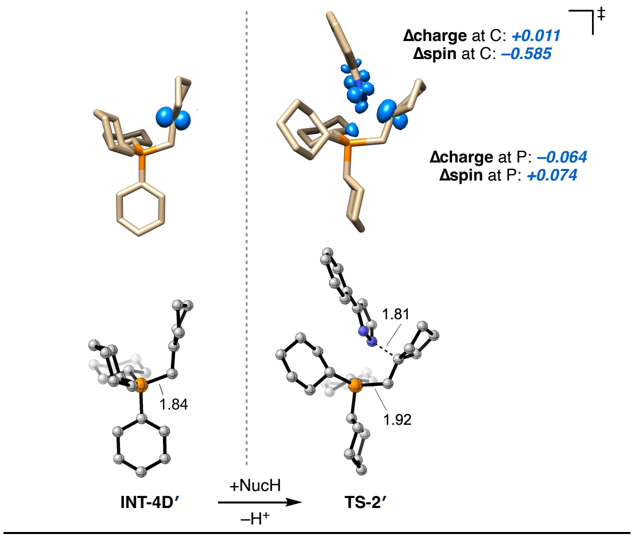

Fig. S50. Top: spin density difference plots for distonic radical cation **INT-4D′** (left) and C–N bond formation transition state **TS-2′** (right); bottom: corresponding structures with bond lengths in Angstroms. NBO charge and spin are labelled for relevant atoms. CYLview and Chimera: Hydrogens omitted for clarity.

Structure	$\Delta E^{\ddagger}_{distort}$	$\Delta E^{\ddagger}_{distort\text{-}cat}$	$\Delta E^{\ddagger}_{distort\text{-}sub}$	$\Delta E^{\ddagger}_{int}$
TS-1 [P(*p*-OMePh)$_3$]	24.7	24.3	0.4	-86.9
TS-2′ [PCy$_3$]	25.3	24.1	1.2	-85.2

Cat = distonic radical cation
Sub = azole

Table S55. Distortion-Interaction Analysis of TS-1 [P(*p*-OMePh)$_3$] and TS-2′ [PCy$_3$]. (Energies in kcal/mol)

TS	E‡(TS)	E‡(cat_TS)	E‡(sub_TS)	E(cat_ground)	E(sub_ground)
TS-1 [P(*p*-OMePh)$_3$]	-2070.999493	-1614.18813	-456.672865	-1614.226878	-456.673502
TS-2′ [PCy$_3$]	-1738.250928	-1281.443552	-456.671621	-1281.481997	-456.673502

Table S56. Electronic energies for Distortion-Interaction Analysis. (Energies in Hartrees)

Comparison of Azole vs. Alkene Nucleophilic Addition

Given that the first step for either regioselectivity proposal differs (Figure 4A), we wanted to computationally investigate whether alkene vs. azole addition into the phosphine radical cation could be the rate- and regio-determining step.

The addition of methylene cyclopentene addition into the P(*p*-OMePh)$_3$ radical cation is exergonic (ΔG = –8.6 kcal/mol), though not as substantial compared to nucleophilic amination with 3-phenylpyrazole (Figure 6C, ΔG = –13.1 kcal/mol, **INT-9 → INT-6A**). However, quantitative characterization of the free energy activation barrier to this addition has been difficult to calculate. We could not observe any local maxima on the electronic potential energy surface when employing a relaxed potential energy surface scan along the trajectory of the C–P bond formation coordinate, suggesting a ‘barrierless’ and reversible alkene addition.

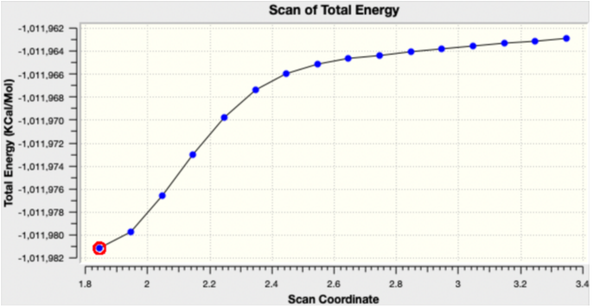

Fig. S51. Relaxed PES scan for methylene cyclopentane addition into P(*p*-OMePh)$_3$ radical cation.

Liu et al. and Ando et al. have also noted a 'barrierless' addition for this type of reactivity[32,33]. If alkene addition into the PRC was Markovnikov regioselectivity determining, downstream intermediates should not be observed under conditions that give exclusive anti-Markovnikov product. However, under anti-Markovnikov conditions[4], the PCy_3 analog of phosphonium **B1** was identified, suggesting that alkene addition to the PRC occurs and is likely not the rate- or regioselectivity-determining step.

With 1-hexene, we found addition to P(*p*-OMePh)$_3$ radical cation to proceed with an activation barrier of $\Delta G^‡$ of +9.9 kcal/mol and ΔG = –5.9 kcal/mol (Fig. S52). This barrier is smaller than that for nucleophilic amination ($\Delta G^‡$ = +14.9 kcal/mol) with 3-phenylpyrazole, further supporting that a kinetically fast and thermodynamically reversible alkene addition to the PRC is not likely to be the rate- or regio-determining step.

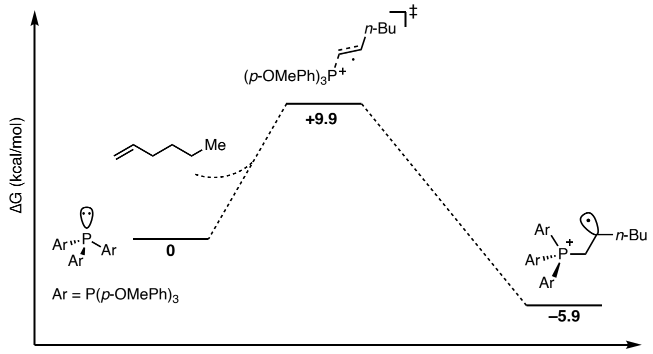

Fig. S52. Addition of 1-hexene to P(*p*-OMePh)$_3$ radical cation.

Azole addition to the phosphine radical cation is also exergonic (ΔG = –26.3 kcal/mol), as calculated by the following equation:

ΔG = (P–N phosphoranyl radical with 3-phenylpyrazole + TRIP–SH) – (TRIP-S$^-$ + NH 3-phenylpyrazole + P(*p*-OMePh)$_3$ radical cation) = –26.3 kcal/mol

The formation of P-N phosphoranyl radical, as proposed to occur under either anti-Markovnikov[1,4] or Markovnikov conditions, requires deprotonation of the azole; however, there are several considerations on how this deprotonation could occur that present a challenge to computing the kinetic barrier(s). If starting with a deprotonated azole, the azole addition step to PRC would also be rapid, given the ionic nature of the reaction. Based on pKa[34], it is unlikely that either phosphine or thiolate can deprotonate the free azole; however, this deprotonation may be possible if the azole is participating in a complex or non-covalent interactions. In considering direct addition of NH azole into the PRC, no stable NH-azole-PRC structure could be identified and efforts to find a transition state were consequently unsuccessful.

Several experimental observations suggest this addition step to be distinct from the regioselectivity-determining step. First, under the same experimental conditions as conducted

with terminal alkenes, we observe both anti-Markovnikov and Markovnikov product with our standard azole and P(*p*-OMePh)$_3$ when an internal alkene is employed:

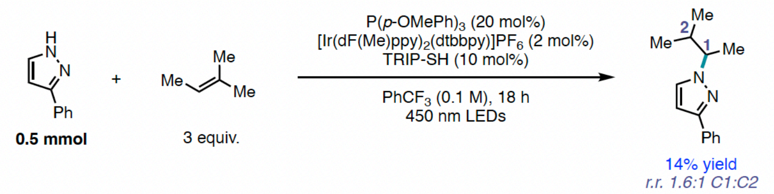

This indicates that under the reaction conditions, competitive azole addition into the PRC will occur, followed by subsequent α-scission to form the NCR, at which point NCR addition into the alkene is rate-determining. Second, although the standard substrate pairing in the anti-Markovnikov conditions[1], benzimidazole and methylene cyclopentene, was not competent with P(*p*-OMePh)$_3$ under the catalytic conditions, we did observe 6% of anti-Markovnikov product, suggesting azole addition into the PRC to be feasible with this triaryl phosphine.

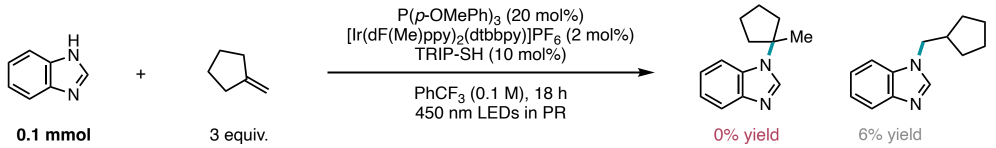

This observation leads us to believe the proposed nucleophilic amination of the distonic phosphine radical cation with methylene cyclopentene with benzimidazole to be kinetically more uphill than that of benzimidazole NCR addition into methylene cyclopentene.

Therefore, although the barriers for azole vs. alkene addition into the PRC may vary, we hypothesize that for a substrate to be reactive, both barriers must be lower than either NCR addition into the alkene (anti-Markovnikov selectivity) and nucleophilic amination into the distonic radical cation (Markovnikov selectivity).

Alternative Product Formation Pathways

Reactivity from P–N Phosphoranyl Radical

We considered P–N phosphoranyl radical **INT-12** addition into the alkene via **TS-D** as an alternative pathway to the productive **INT-9** (right); however, this process is prohibitively high kinetically under the reaction conditions and is thermodynamically unfavorable. Instead, α-scission from **INT-12** is kinetically preferred (left).

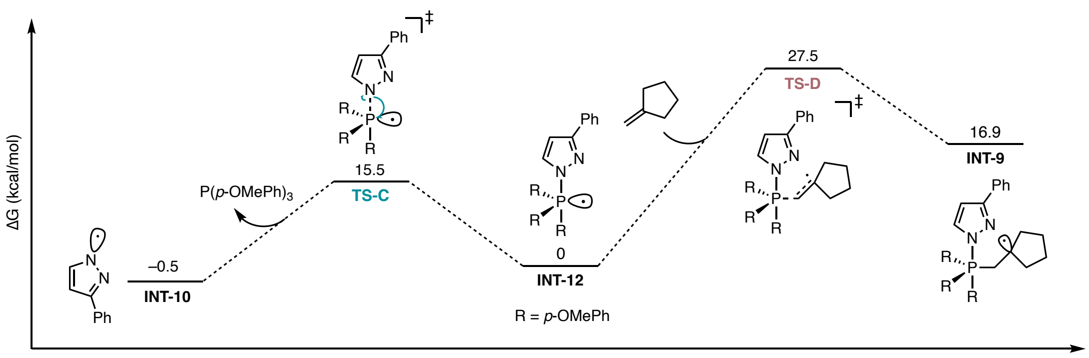

Fig. S53. Potential reactivity pathways from P–N phosphoranyl radical **INT-12**.

Deprotonation via Ternary Complex

We considered a ternary complex involving 3-phenylpyrazole, thiolate, and distonic radical cation **INT-4D** to probe where deprotonation of the azole occurs in the nucleophilic amination step following pathway (ii). We were unable to find a formal transition state, but through constrained optimization calculations found a ternary complex with imaginary frequencies of -599.14 cm^{-1} and -36.76 cm^{-1}. The former frequency corresponds to a vibrational mode along the C–N bond, while the latter frequency corresponds to a vibrational mode along the S–H–N bond. These results suggest a concerted deprotonation with all three components together is unlikely; rather, deprotonation is likely to occur stepwise. Given the smaller magnitude of the S–H–N vibrational mode, deprotonation of the azole is fast. Hence, we considered a deprotonated azole in our calculations for **TS-1** and **TS-2**, with the energies of the thiolate / thiol accounted for in the thermodynamic calculations.

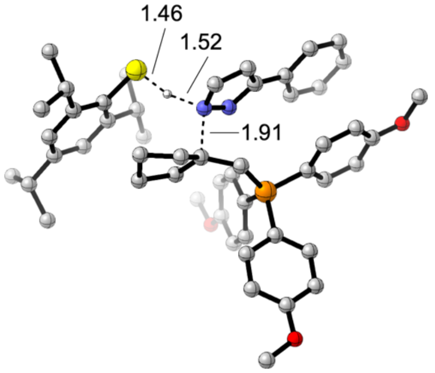

Fig. S54. Ternary complex for concerted deprotonation / nucleophilic addition into **INT-4D**.

Selectivity in Indole Functionalization

For indole *N*-alkylated product **16**, the remaining mass balance is isolated unreacted indole starting material and byproduct resulting from **INT-7**-type addition into excess hexene. Letting the reaction run for longer (36 h instead of 18 h) did not lead to improved yield.

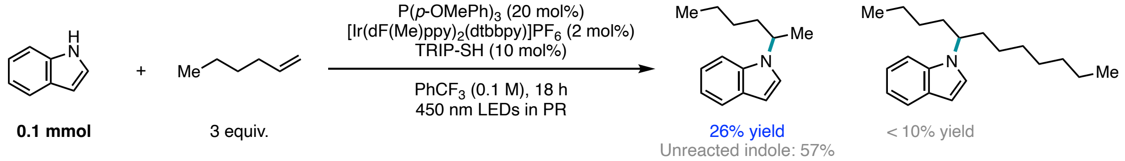

We did not observe any *C3*-alkylated indole derivatives, which we hypothesize is due to the conformational unfavourability of *C3* addition into the distonic radical cation, as computational attempts to locate a transition state complex were unsuccessful. Alternatively, we considered indole addition to proceed through a P(V) intermediate of the **INT-9** type. We found a stable ground-state complex **INT-9-I** and "migratory insertion" transition state **TS-1-I** with the indole nitrogen (see below) in line with the proposed nucleophilic amination pathway.

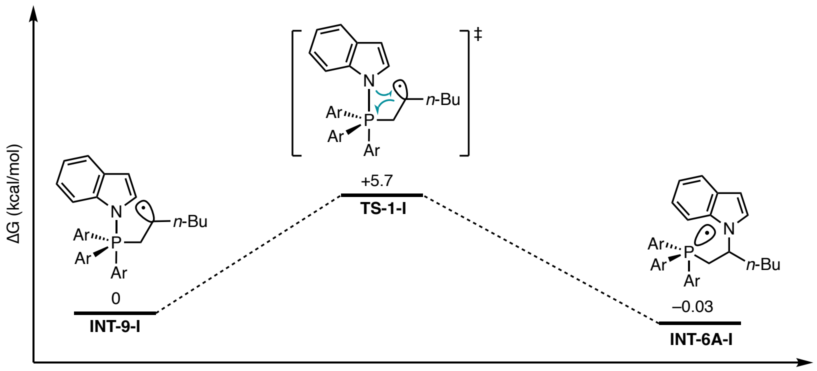

Fig. S55. C–N bond formation with indole and 1-hexene.

11. References

1. Sedillo, K., Fan, F., Knowles, R. R. & Doyle, A. G. Cooperative Phosphine-Photoredox Catalysis Enables N–H Activation of Azoles for Intermolecular Olefin Hydroamination. *J. Am. Chem. Soc.* **146**, 20349–20356 (2024).

2. Collins, K. D., Rühling, A. & Glorius, F. Application of a robustness screen for the evaluation of synthetic organic methodology. *Nat. Protoc.* **9**, 1348–1353 (2014).

3. Ma, S., Xi, Y., Fan, H., Roediger, S. & Hartwig, J. F. Enantioselective hydroamination of unactivated terminal alkenes. *Chem* **8**, 532–542 (2022).

4. Chinn, A. J., Sedillo, K. & Doyle, A. G. Phosphine/Photoredox Catalyzed Anti-Markovnikov Hydroamination of Olefins with Primary Sulfonamides via α-Scission from Phosphoranyl Radicals. *J. Am. Chem. Soc.* **143**, 18331–18338 (2021).

5. Cismesia, M. A. & Yoon, T. P. Characterizing chain processes in visible light photoredox catalysis. *Chem. Sci.* **6**, 5426–5434 (2015).

6. Ruos, M. E., Kinney, R. G., Ring, O. T. & Doyle, A. G. A General Photocatalytic Strategy for Nucleophilic Amination of Primary and Secondary Benzylic C–H Bonds. *J. Am. Chem. Soc.* **145**, 18487–18496 (2023).

7. Hatchard, C. G. & Parker, C. A. A new sensitive chemical actinometer - II. Potassium ferrioxalate as a standard chemical actinometer. *Proc. R. Soc. Lond. Ser. A Math. Phys. Sci.* **235**, 518–536 (1956).

8. Masuda, Y., Tsuda, H. & Murakami, M. Photoinduced Dearomatizing Three-Component Coupling of Arylphosphines, Alkenes, and Water. *Angew. Chem. Int. Ed.* **60**, 3551–3555 (2021).

9. Masuda, Y., Uno, M. & Murakami, M. Photoinduced Reaction of Triarylphosphines with Alkenes Forming Fused Tricyclic Phosphonium Salts. *Org. Lett.* **23**, 8445–8449 (2021).

10. Jiu, A. Y., Slocumb, H. S., Yeung, C. S., Yang, X. & Dong, V. M. Enantioselective Addition of Pyrazoles to Dienes**. *Angew. Chem. Int. Ed.* **60**, 19660–19664 (2021).

11. Hansch, C., Leo, A. & Taft, R. W. A survey of Hammett substituent constants and resonance and field parameters. *Chem. Rev.* **91**, 165–195 (1991).

12. Creary, X. Super Radical Stabilizers. *Acc. Chem. Res.* **39**, 761–771 (2006).

13. Dust, J. M. & Arnold, D. R. Substituent effects on benzyl radical ESR hyperfine coupling constants. The σα• scale based upon spin delocalization. *J. Am. Chem. Soc.* **105**, 1221–1227 (1983).

14. Paton, R. S. *Patonlab/Kinisot: Kinisot Version 2.0.2 (v2.0.2)*. (Zenodo, 2023).

15. Teegardin, K., Day, J. I., Chan, J. & Weaver, J. Advances in Photocatalysis: A Microreview of Visible Light Mediated Ruthenium and Iridium Catalyzed Organic Transformations. *Org. Process Res. Dev.* **20**, 1156–1163 (2016).

16. Frisch, M. J. *et al. Gaussian 16 Rev. A.03*. (Wallingford, CT, 2016).

17. Zhao, Y. & Truhlar, D. G. The M06 suite of density functionals for main group thermochemistry, thermochemical kinetics, noncovalent interactions, excited states, and transition elements: two new functionals and systematic testing of four M06-class functionals and 12 other functionals. *Theor. Chem. Acc.* **120**, 215–241 (2008).

18. Weigend, F. & Ahlrichs, R. Balanced basis sets of split valence, triple zeta valence and quadruple zeta valence quality for H to Rn: Design and assessment of accuracy. *Phys. Chem. Chem. Phys.* **7**, 3297–3305 (2005).

19. Weigend, F. Accurate Coulomb-fitting basis sets for H to Rn. *Phys. Chem. Chem. Phys.* **8**, 1057–1065 (2006).

20. Pracht, P., Bohle, F. & Grimme, S. Automated exploration of the low-energy chemical space with fast quantum chemical methods. *Phys. Chem. Chem. Phys.* **22**, 7169–7192 (2020).

21. Marenich, A. V., Cramer, C. J. & Truhlar, D. G. Universal Solvation Model Based on Solute Electron Density and on a Continuum Model of the Solvent Defined by the Bulk Dielectric Constant and Atomic Surface Tensions. *J. Phys. Chem. B* **113**, 6378–6396 (2009).

22. Ribeiro, R. F., Marenich, A. V., Cramer, C. J. & Truhlar, D. G. Use of Solution-Phase Vibrational Frequencies in Continuum Models for the Free Energy of Solvation. *J. Phys. Chem. B* **115**, 14556–14562 (2011).

23. Schäfer, A., Huber, C. & Ahlrichs, R. Fully optimized contracted Gaussian basis sets of triple zeta valence quality for atoms Li to Kr. *J. Chem. Phys.* **100**, 5829–5835 (1994).

24. Luchini, G., Alegre-Requena, J. V., Funes-Ardoiz, I. & Paton, R. S. GoodVibes: automated thermochemistry for heterogeneous computational chemistry data. *F1000Research* **9**, 291 (2020).

25. Grimme, S. Supramolecular Binding Thermodynamics by Dispersion-Corrected Density Functional Theory. *Chem. A Eur. J.* **18**, 9955–9964 (2012).

26. Li, Y.-P., Gomes, J., Sharada, S. M., Bell, A. T. & Head-Gordon, M. Improved Force-Field Parameters for QM/MM Simulations of the Energies of Adsorption for Molecules in Zeolites and a Free Rotor Correction to the Rigid Rotor Harmonic Oscillator Model for Adsorption Enthalpies. *J. Phys. Chem. C* **119**, 1840–1850 (2015).

27. Glendening, E. D., Reed, A. E., Carpenter, J. E. & Weinhold, F. *NBO Version 3.1*.

28. Bickelhaupt, F. M. & Houk, K. N. Analyzing Reaction Rates with the Distortion/Interaction-Activation Strain Model. *Angew. Chem. Int. Ed.* **56**, 10070–10086 (2017).

29. Legault, C. Y. *CYLview, 1.0b*. (Université de Sherbrooke, 2009).

30. Schrodinger, L. *The PyMOL Molecular Graphics System, Version 2.1.* (2015).

31. Pettersen, E. F. *et al.* UCSF Chimera--a visualization system for exploratory research and analysis. *J. Comput. Chem.* **25**, (2004).

32. Liu, K. *et al.* Photon-driven radical hydro-phosphoniumylation of unactivated olefins. *Chem Catal.* **5**, (2024).

33. Ando, T., Yokogawa, D., Ohmatsu, K. & Ooi, T. Deoxygenative [3 + 2] Annulation of α,β-Unsaturated Carbonyl Compounds and Electron-Rich Olefins via Photocatalytic Umpolung of Triarylphosphine. *J. Am. Chem. Soc.* **147**, 24220-24224 (2025).

34. Williams, R. pKa Data. Preprint at https://organicchemistrydata.org/hansreich/resources/pka/pka_data/pka-compilation-williams.pdf (2022).

12. NMR Characterization

HETEROCYCLES

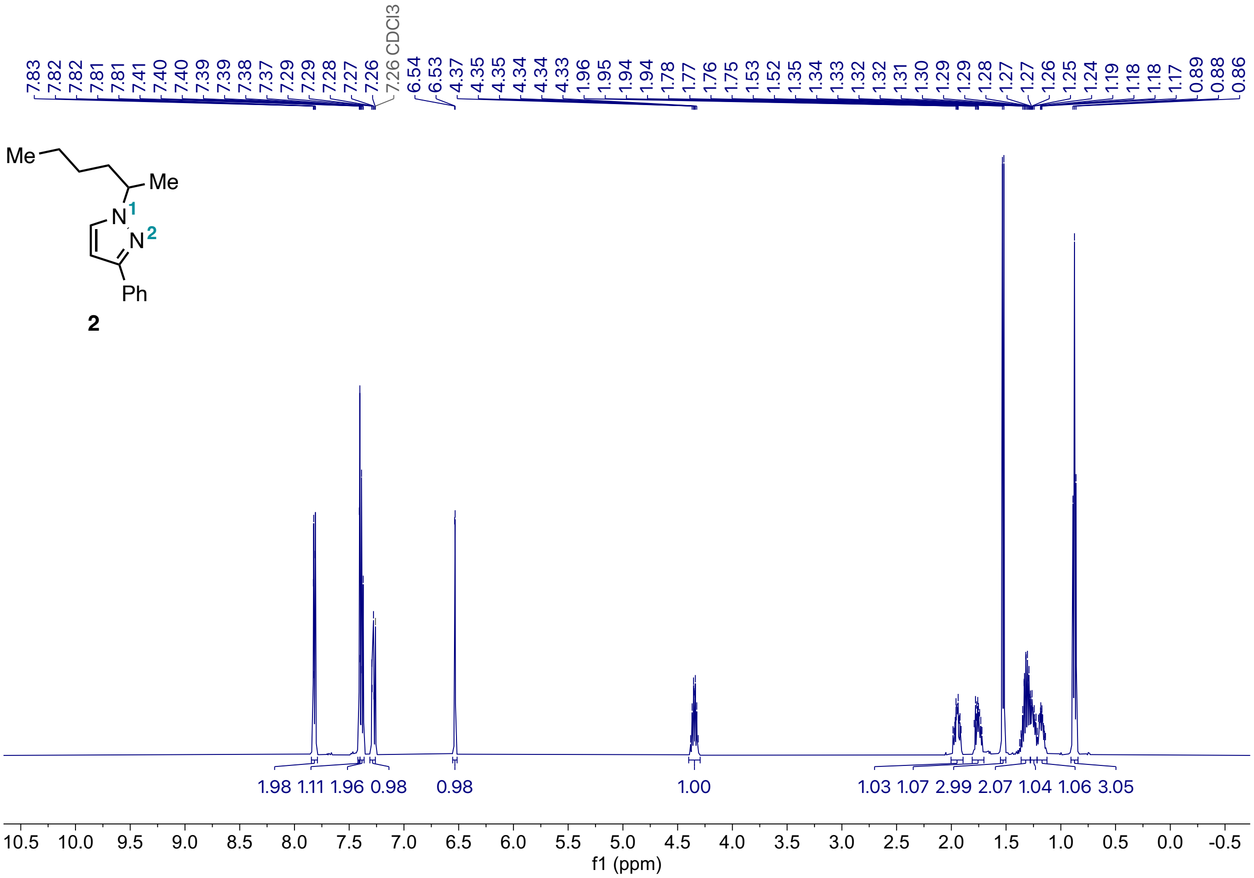

^{1}H NMR spectrum of **2** taken in $CDCl_3$.

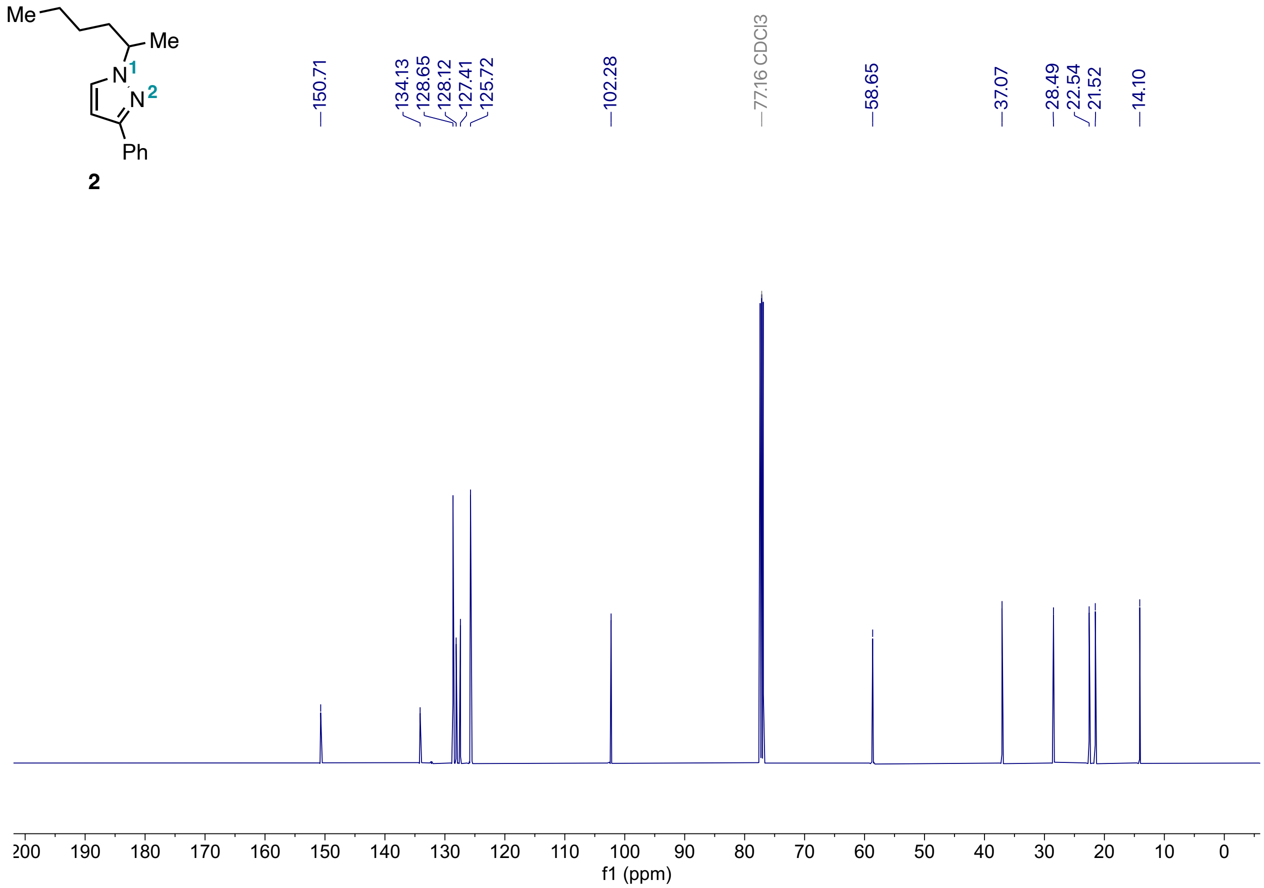

^{13}C NMR spectrum of **2** taken in $CDCl_3$.

HMBC correlation between ^{13}C peak at 128.1 ppm and proton at 4.3 ppm supports N1 isomer.

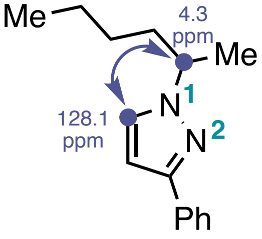

2

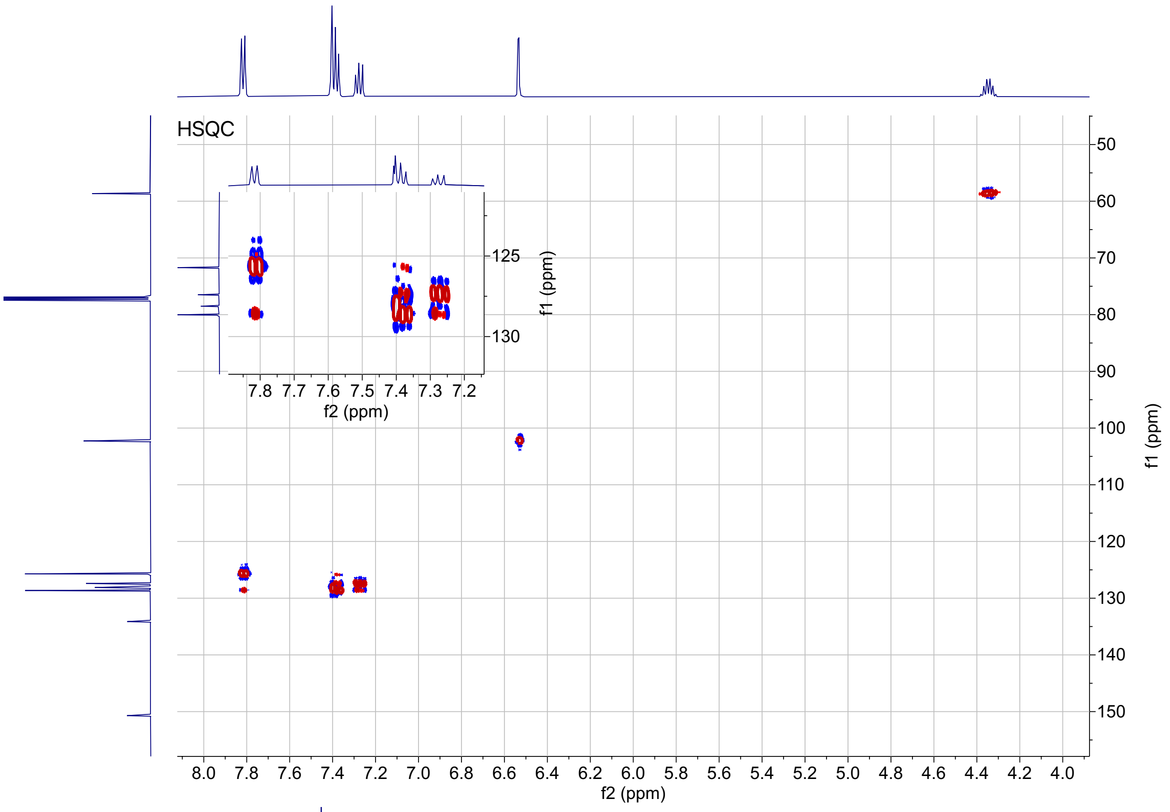
HSQC
f1 (ppm)
f2 (ppm)

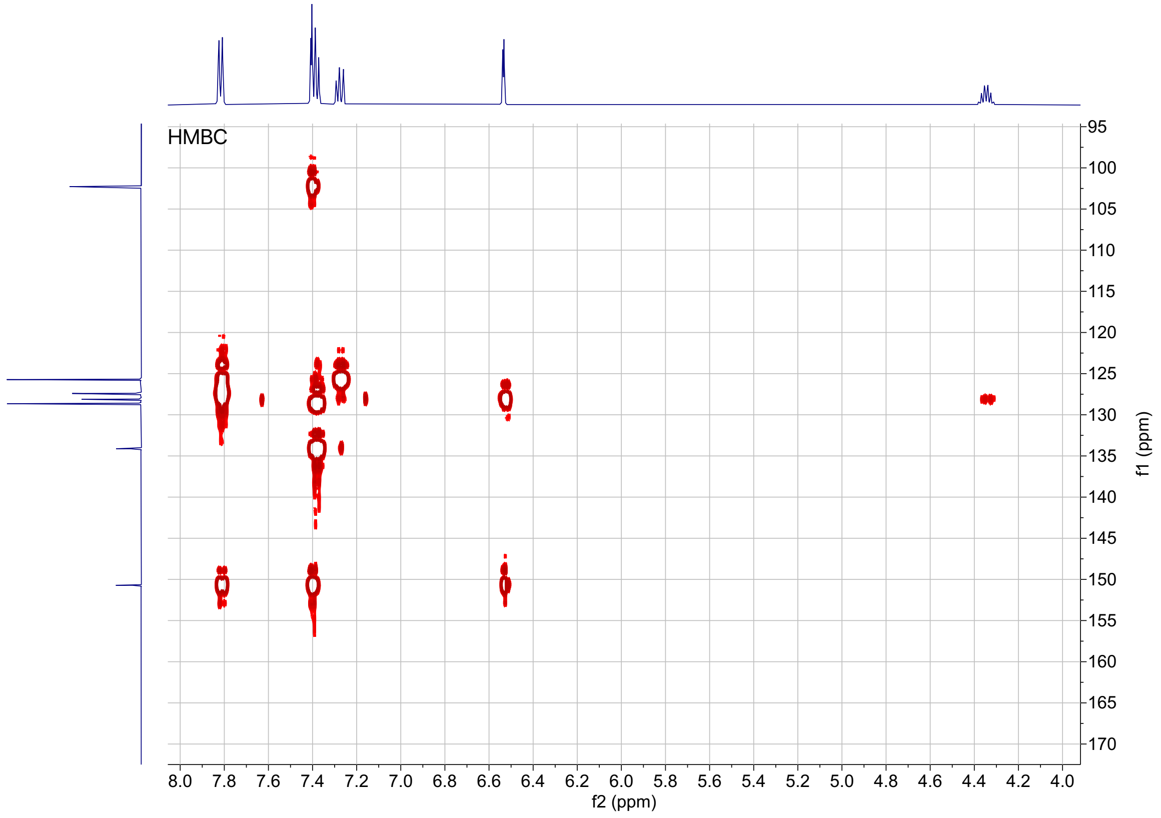
HMBC
f1 (ppm)
f2 (ppm)

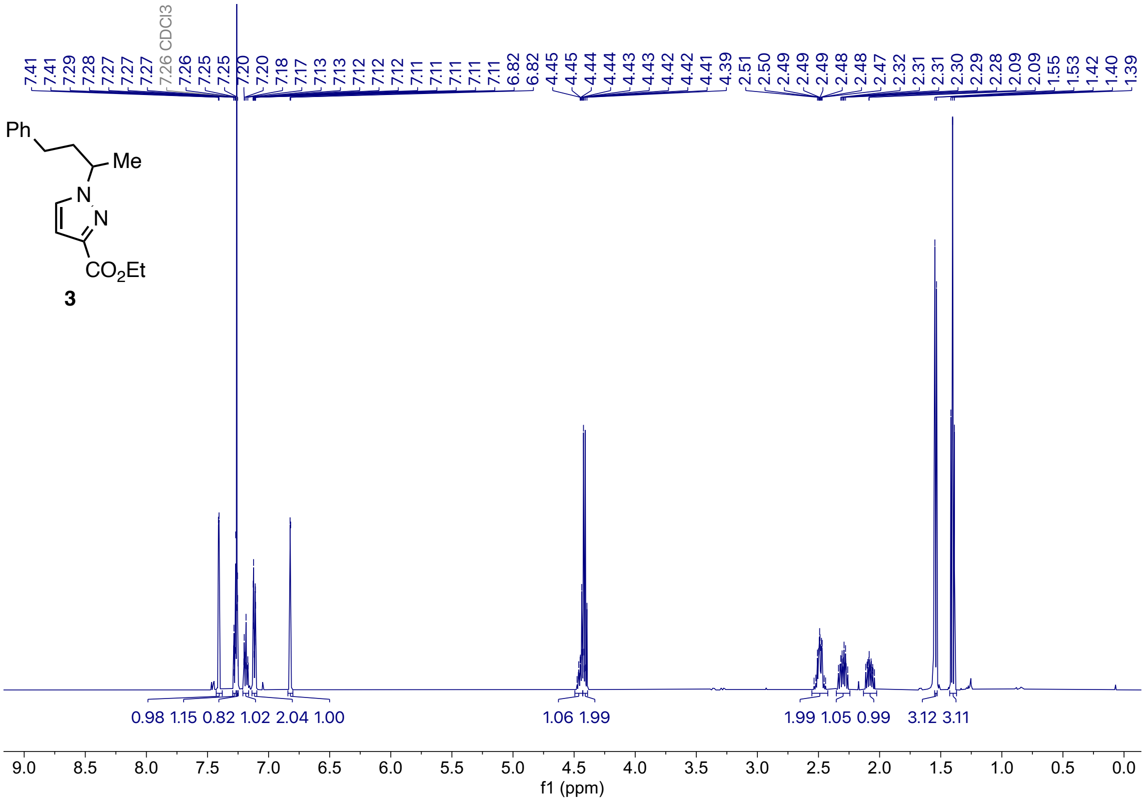

^{1}H NMR spectrum of **3** taken in $CDCl_3$.

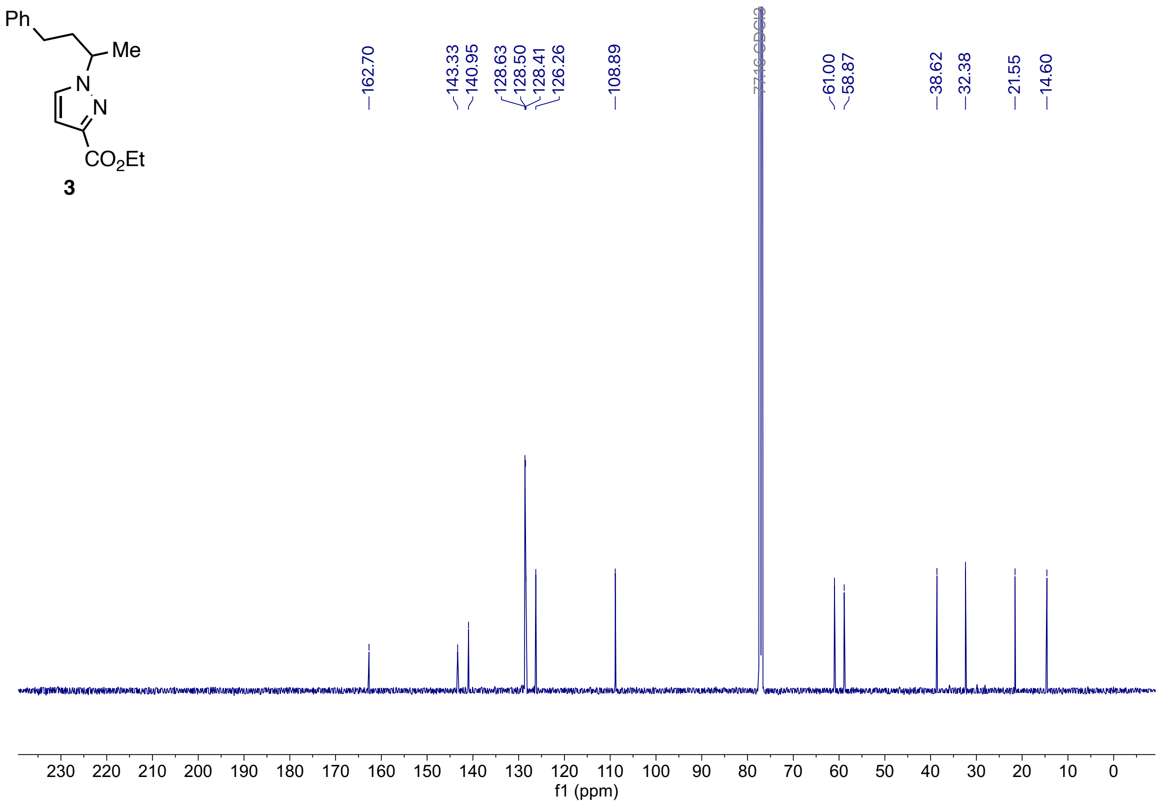

^{13}C NMR spectrum of **3** taken in $CDCl_3$.

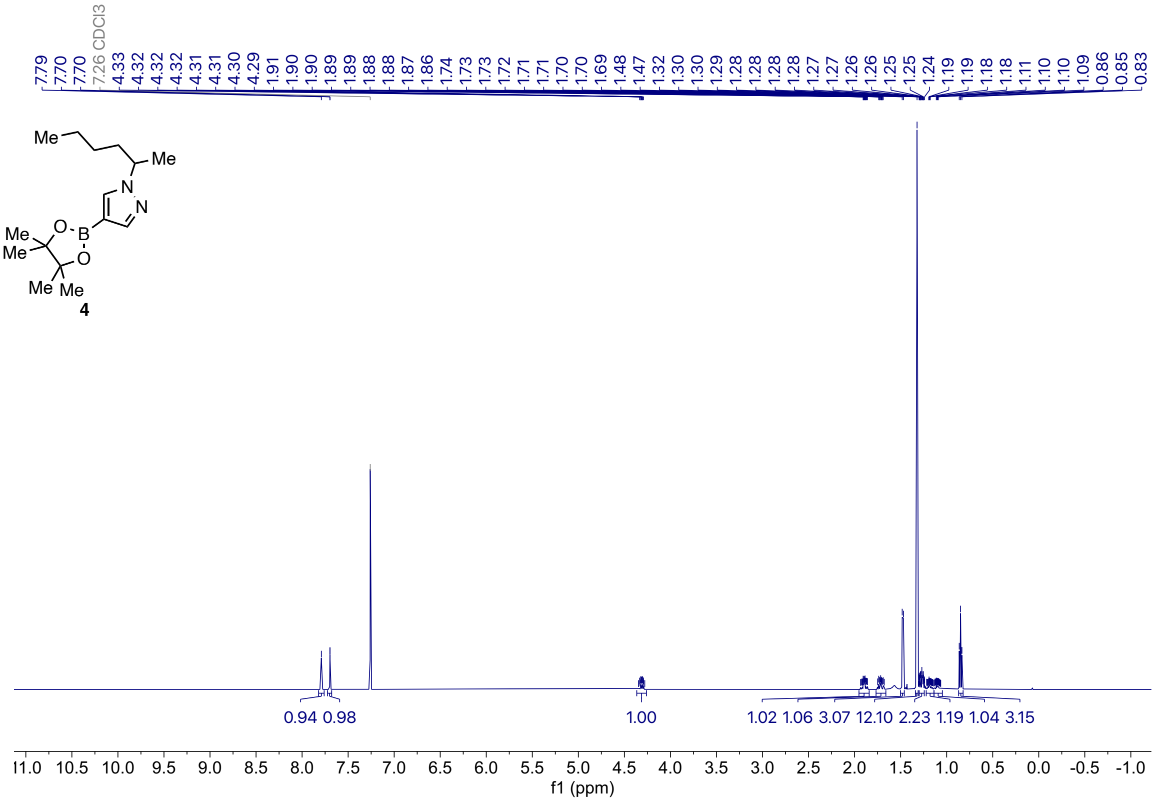

^{1}H NMR spectrum of **4** taken in $CDCl_3$.

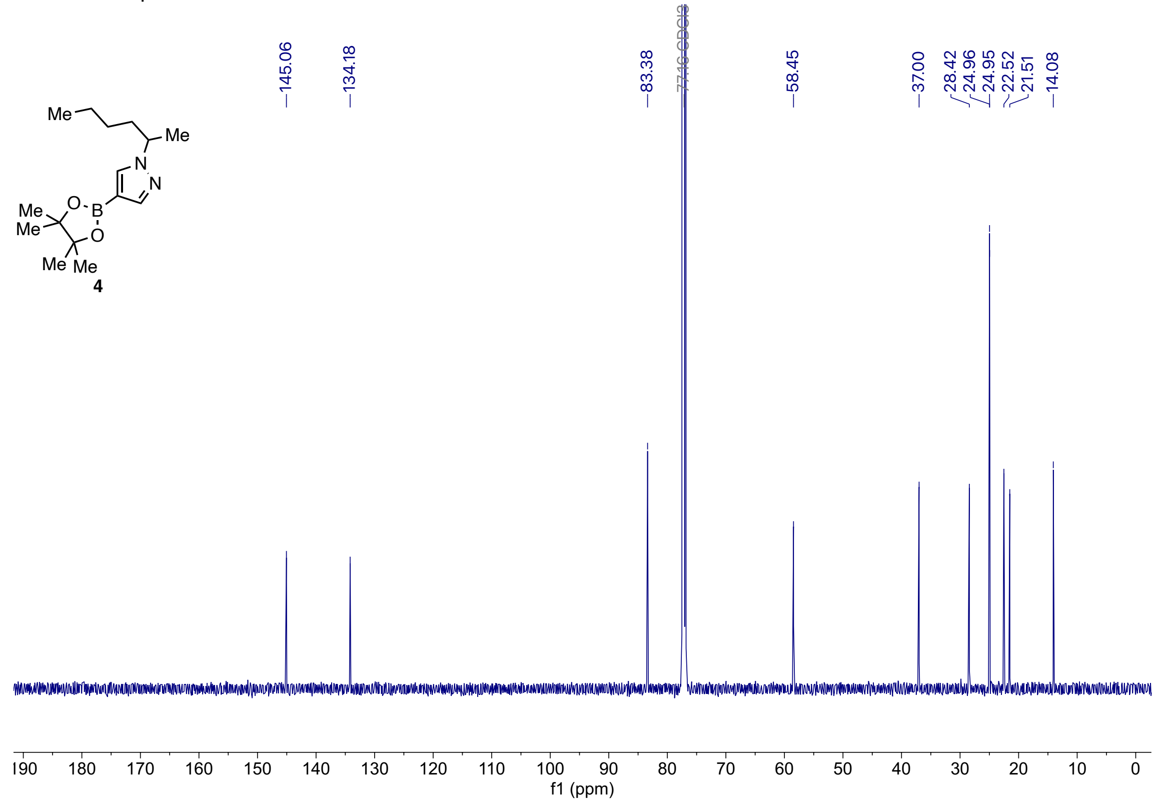

^{13}C NMR spectrum of **4** taken in $CDCl_3$.

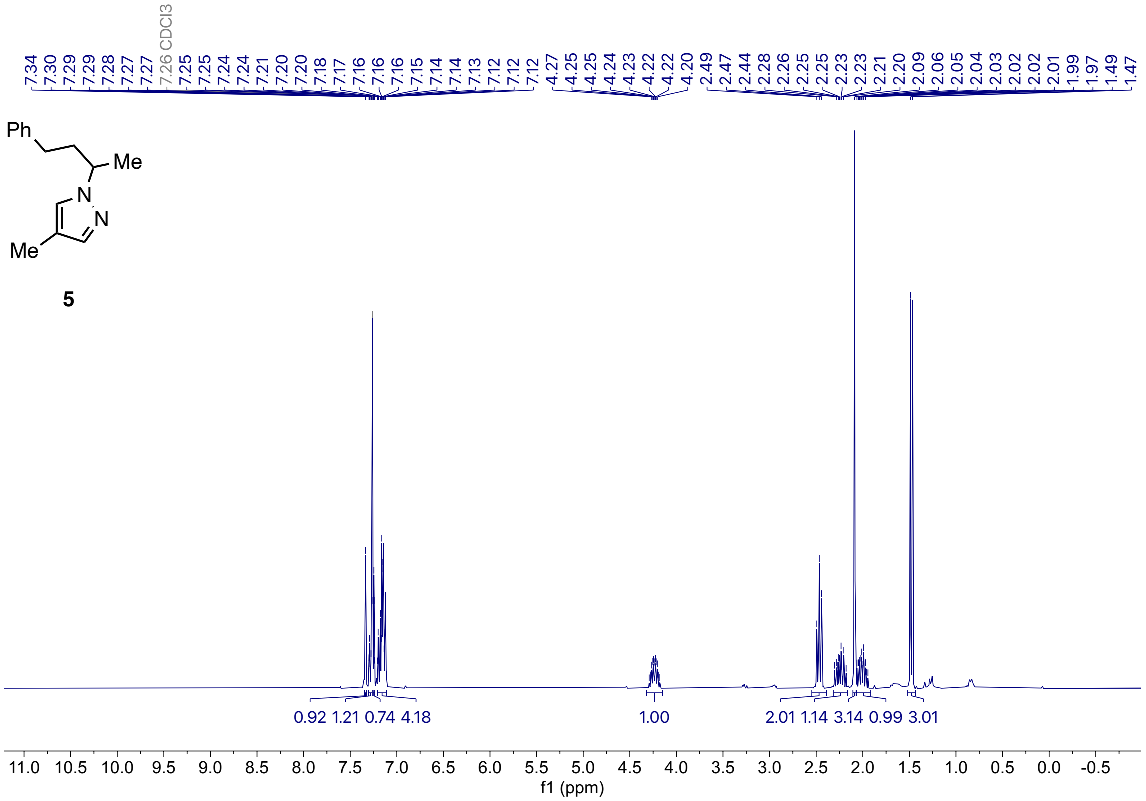

^{1}H NMR spectrum of **5** taken in $CDCl_3$.

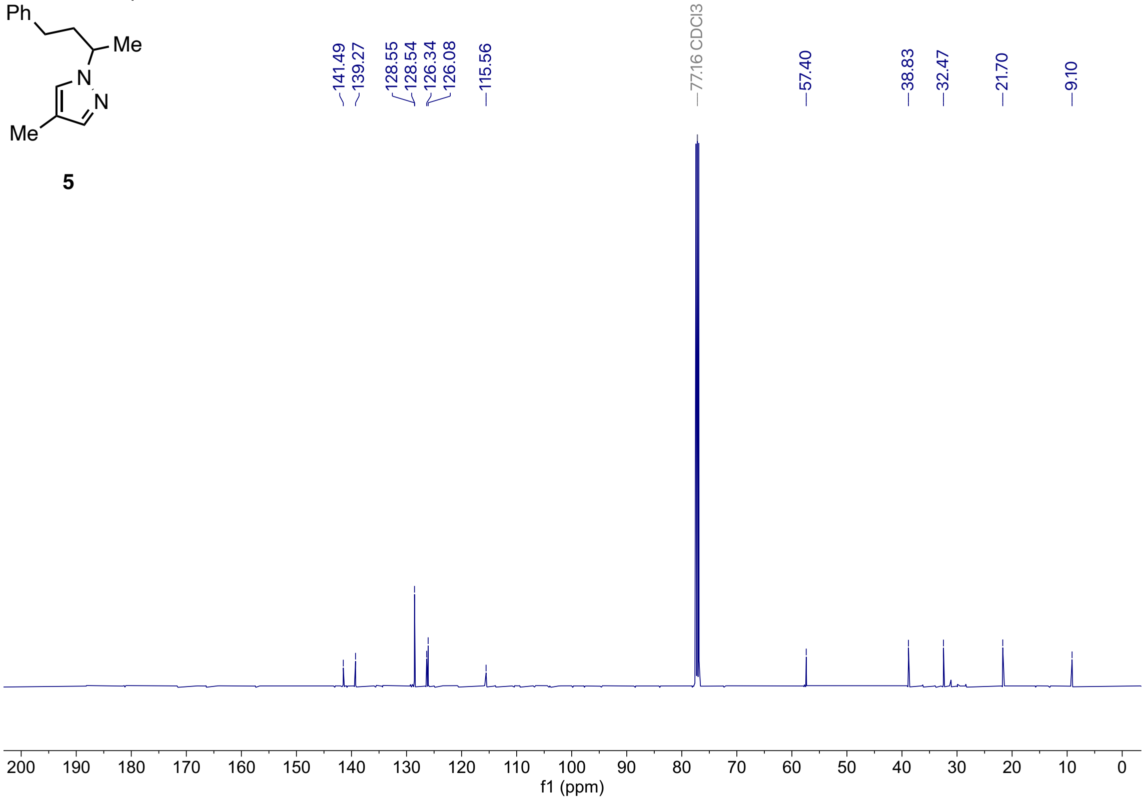

^{13}C NMR spectrum of **5** taken in $CDCl_3$.

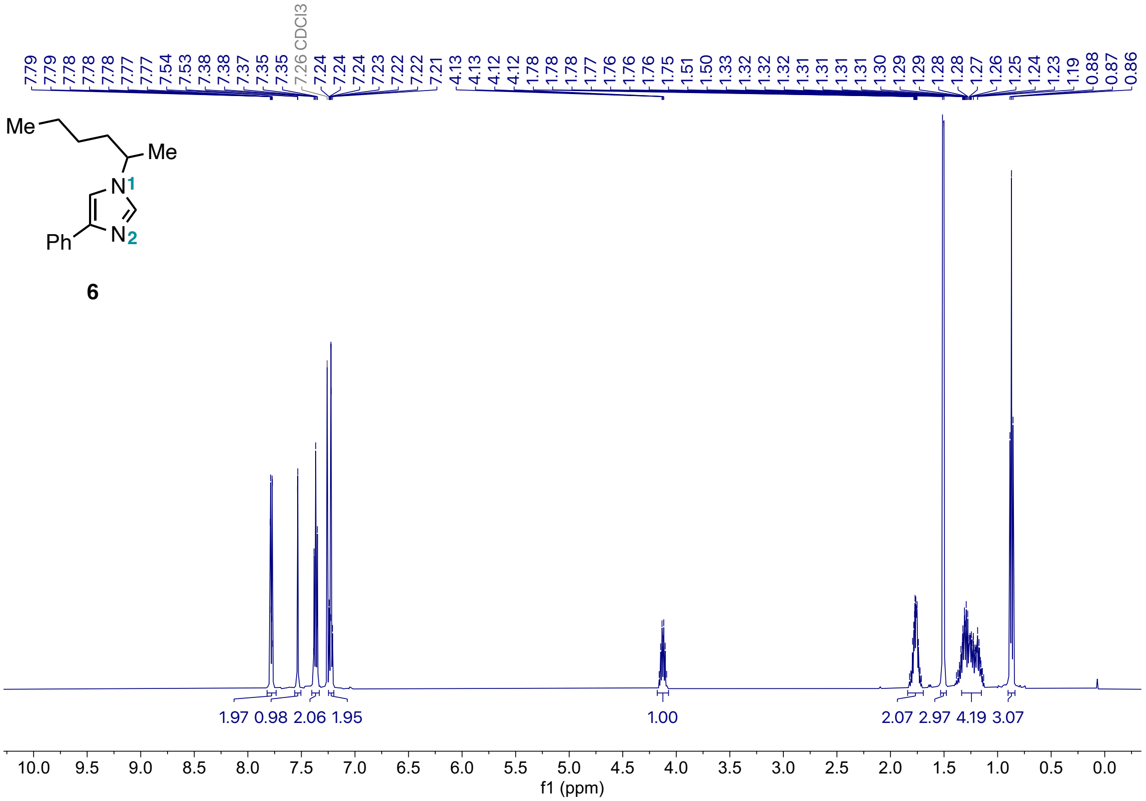

¹H NMR spectrum of **6** taken in $CDCl_3$.

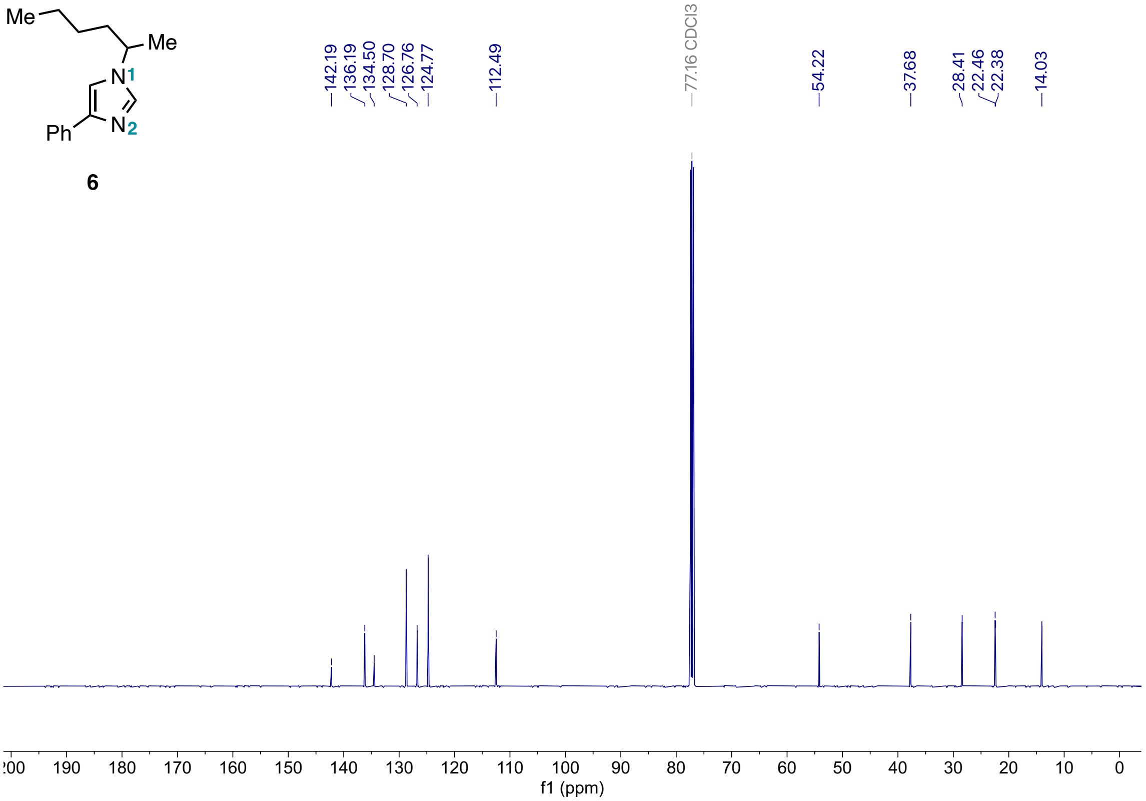

HMBC correlations between ^{13}C peaks at 136.3 ppm and 112.4 ppm, and proton at 4.1 ppm supports N1 isomer.

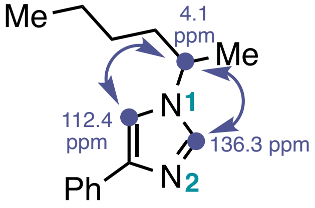

6

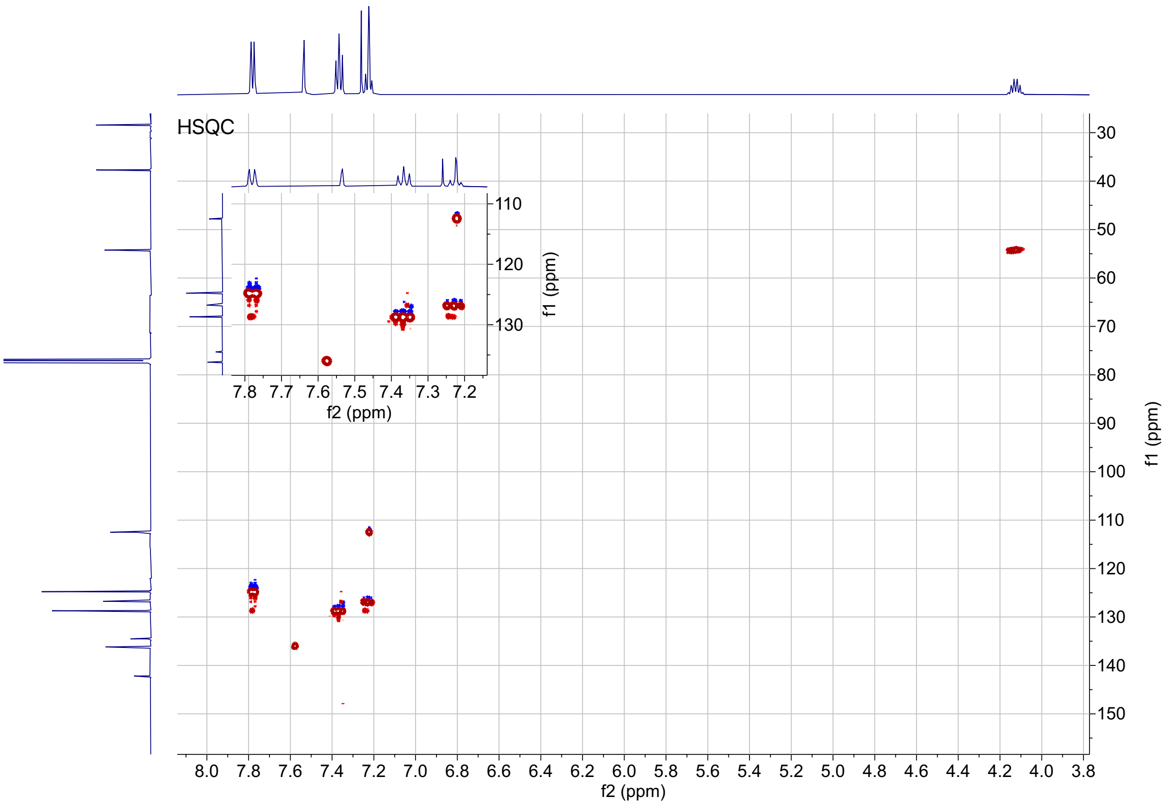

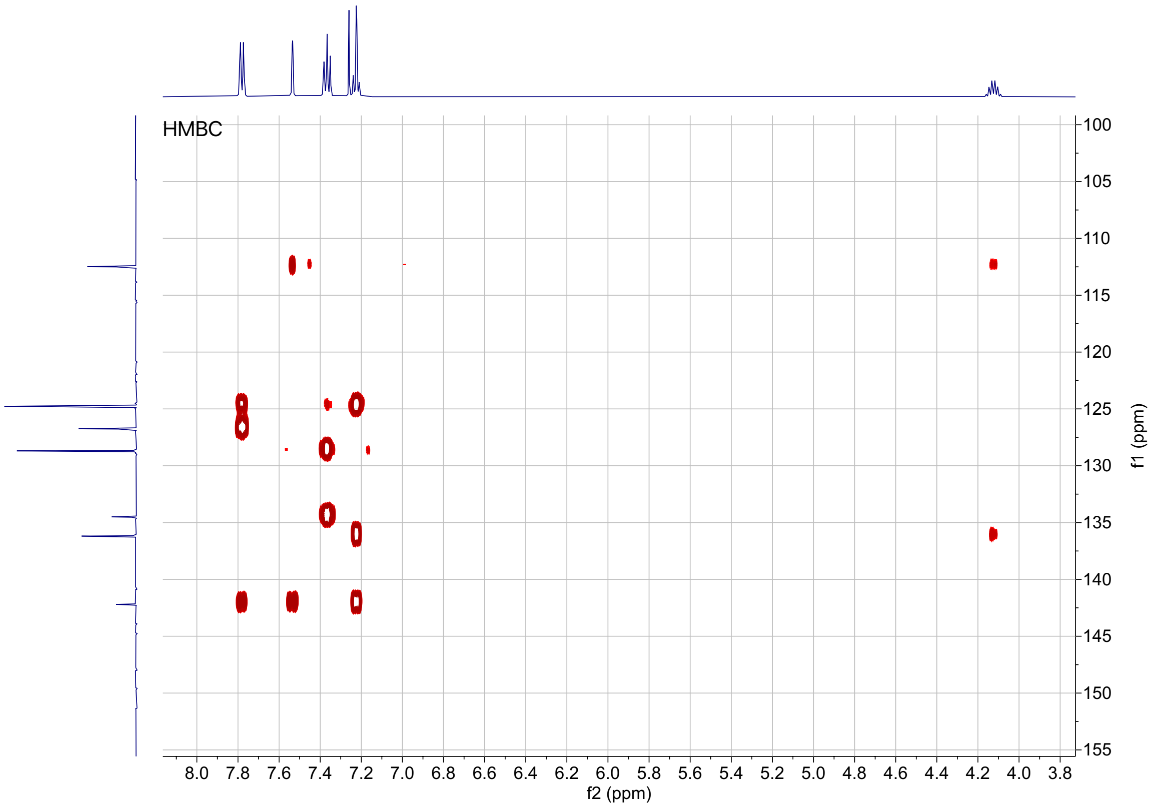
HMBC
8.0
7.8
7.6
7.4
7.2
7.0
6.8
6.6
6.4
6.2
6.0
5.8
5.6
5.4
5.2
5.0
4.8
4.6
4.4
4.2
4.0
3.8
f2 (ppm)
100
105
110
115
120
125
130
135
140
145
150
155
f1 (ppm)

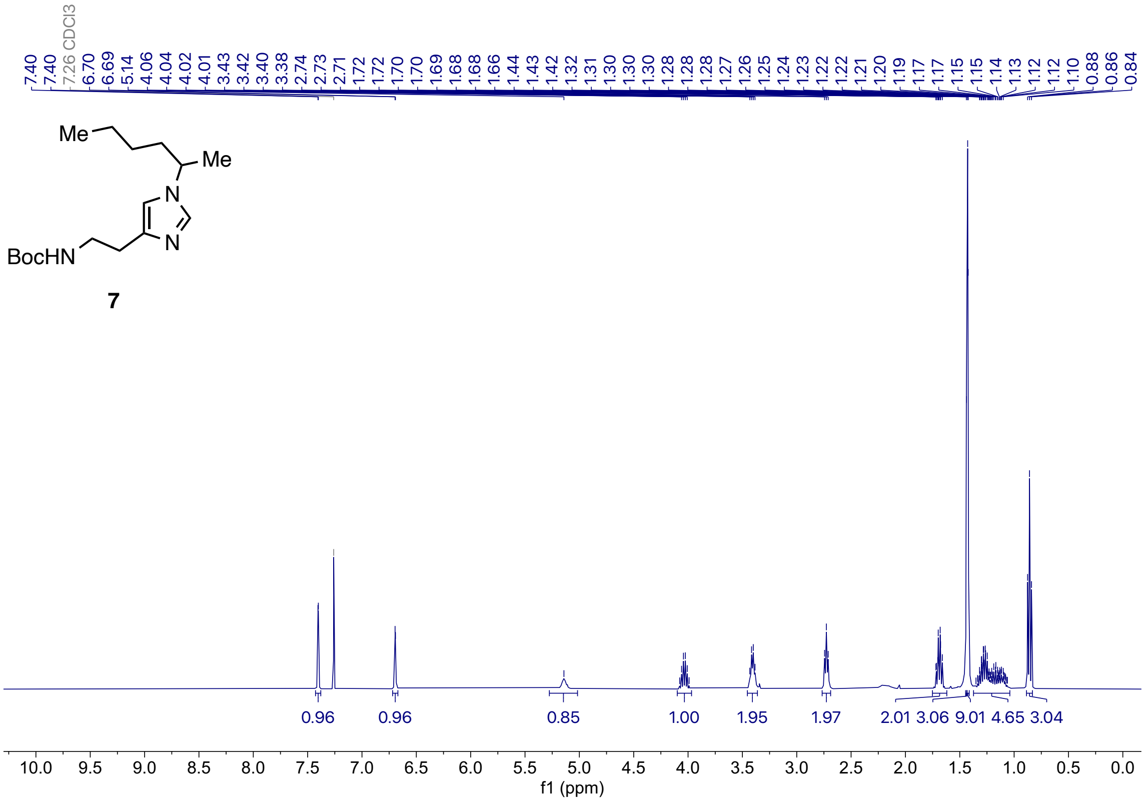

^{1}H NMR spectrum of **7** taken in $CDCl_3$.

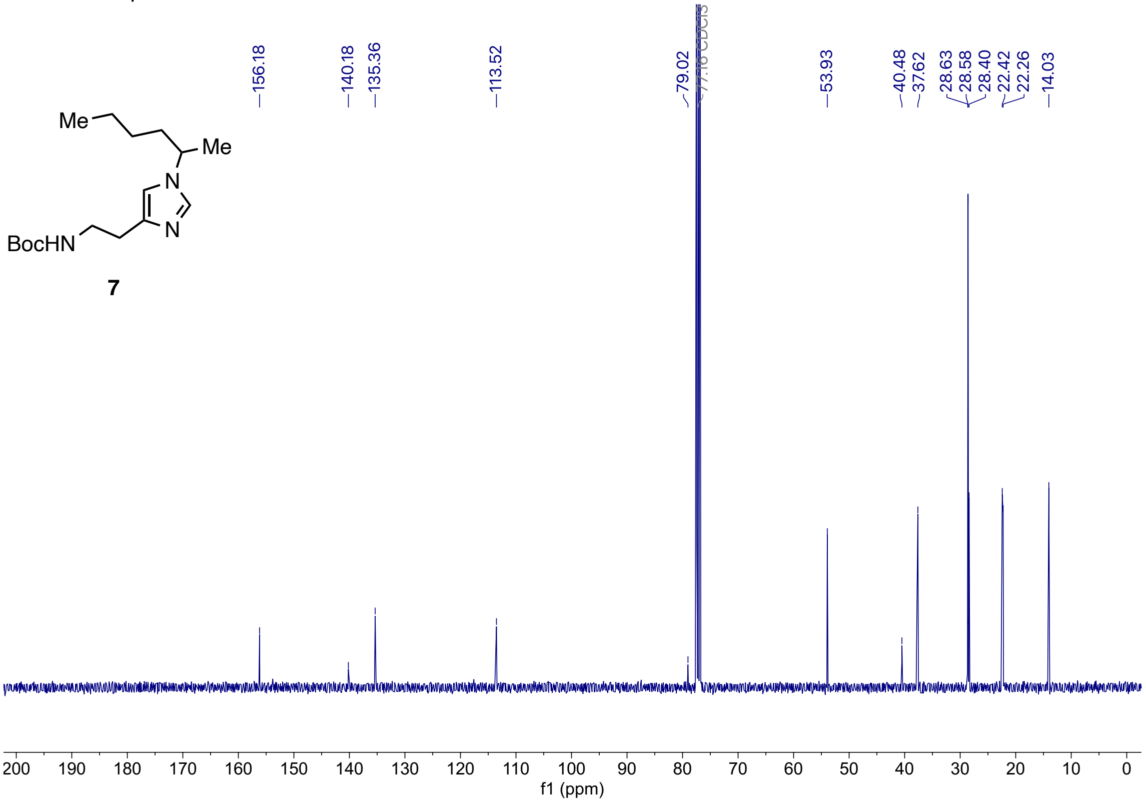

^{13}C NMR spectrum of **7** taken in $CDCl_3$.

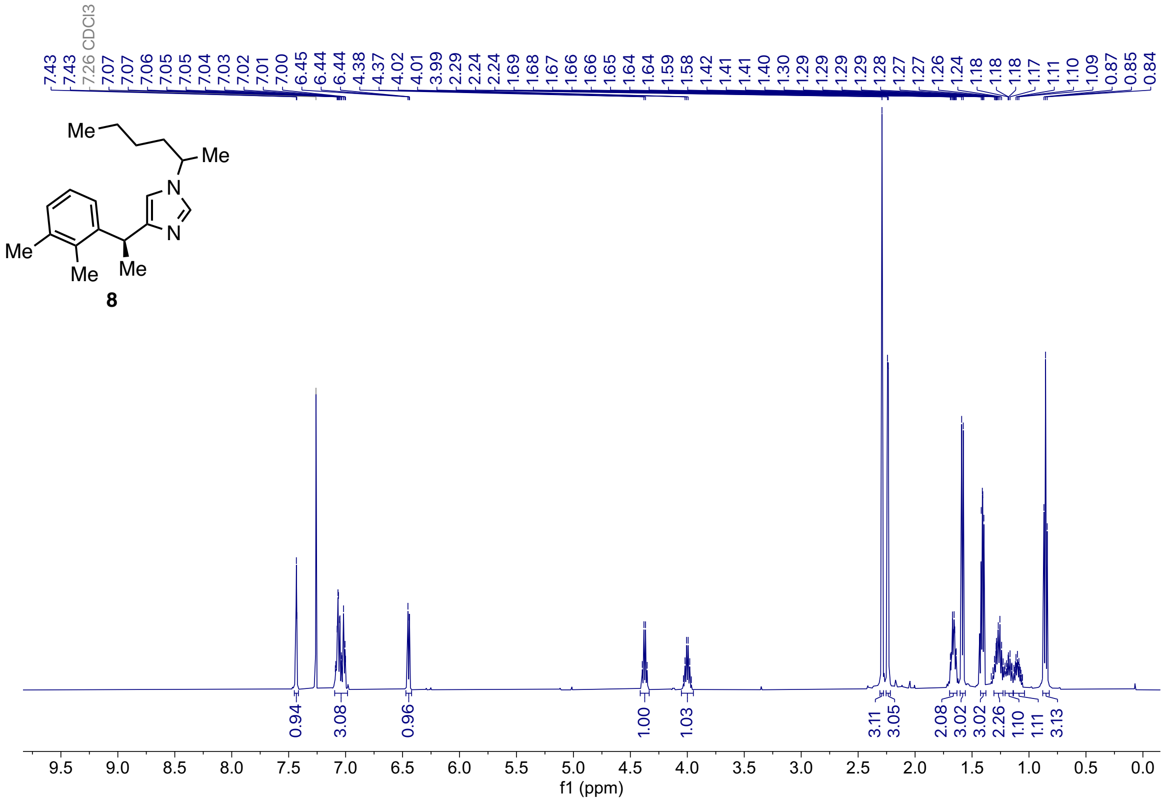

^{1}H NMR spectrum of **8** taken in $CDCl_3$.

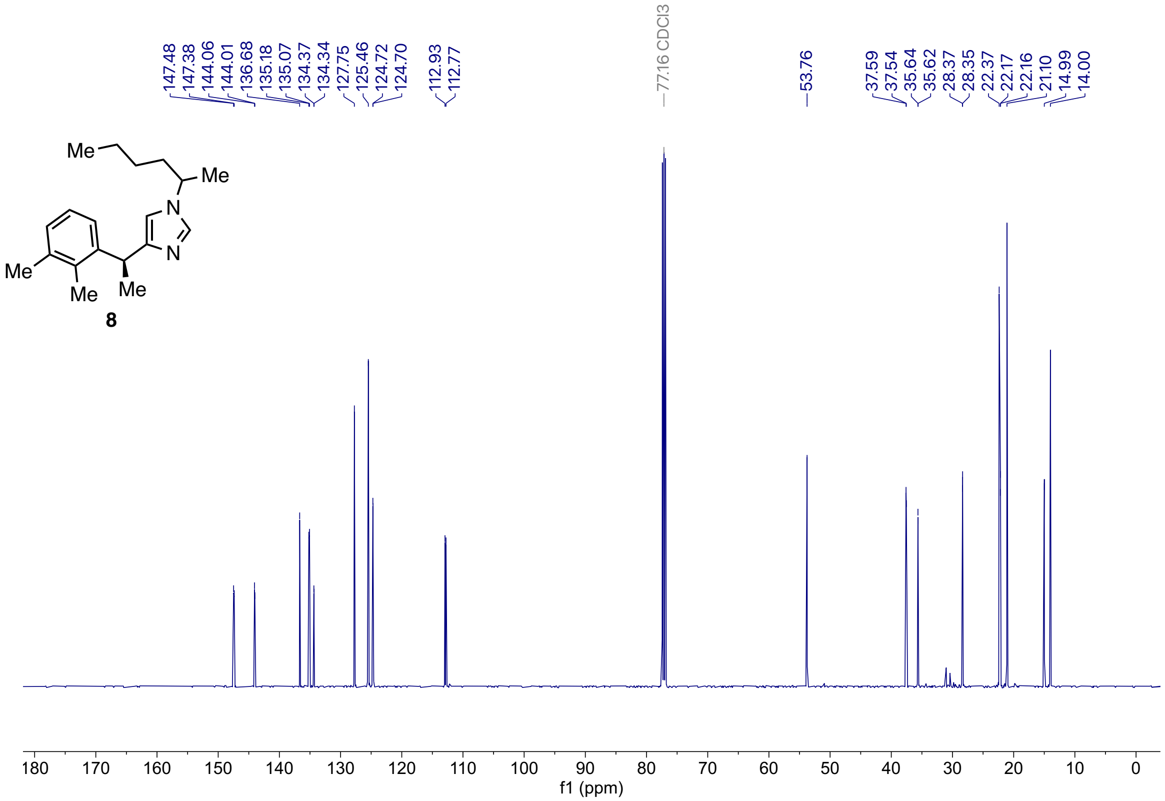

^{13}C NMR spectrum of **8** taken in $CDCl_3$.

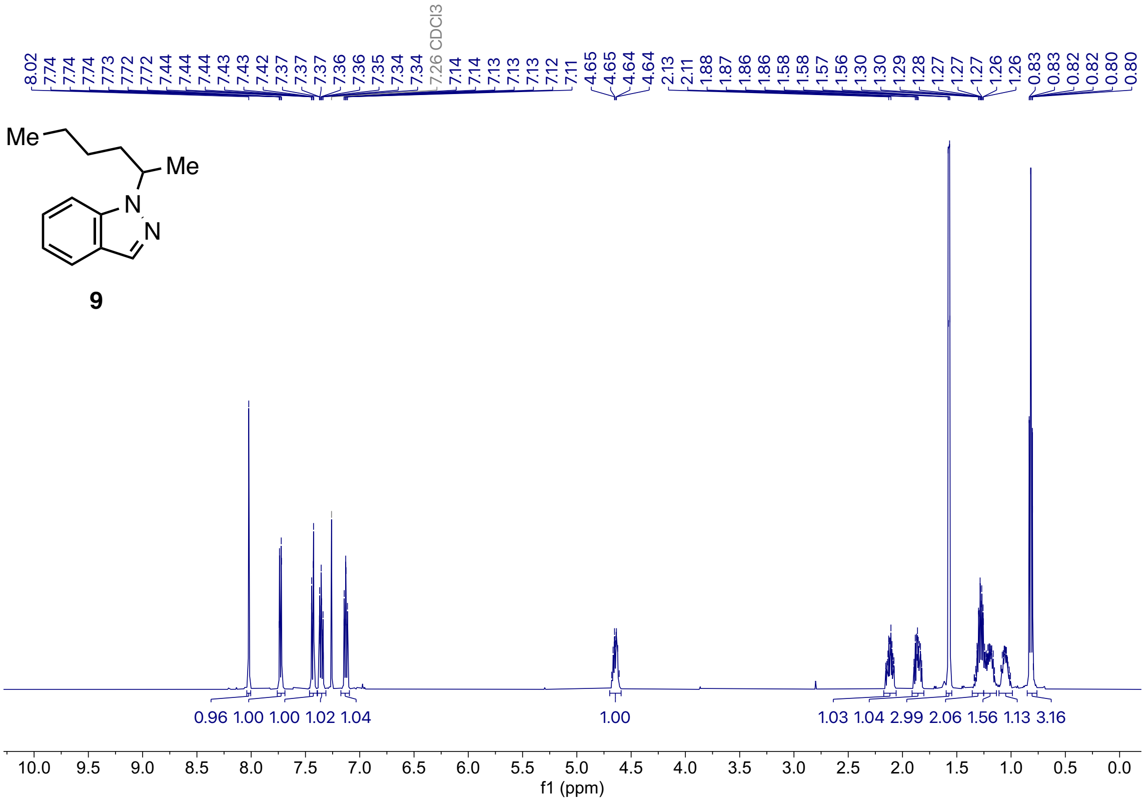

9

^{1}H NMR spectrum of **9** taken in $CDCl_3$.

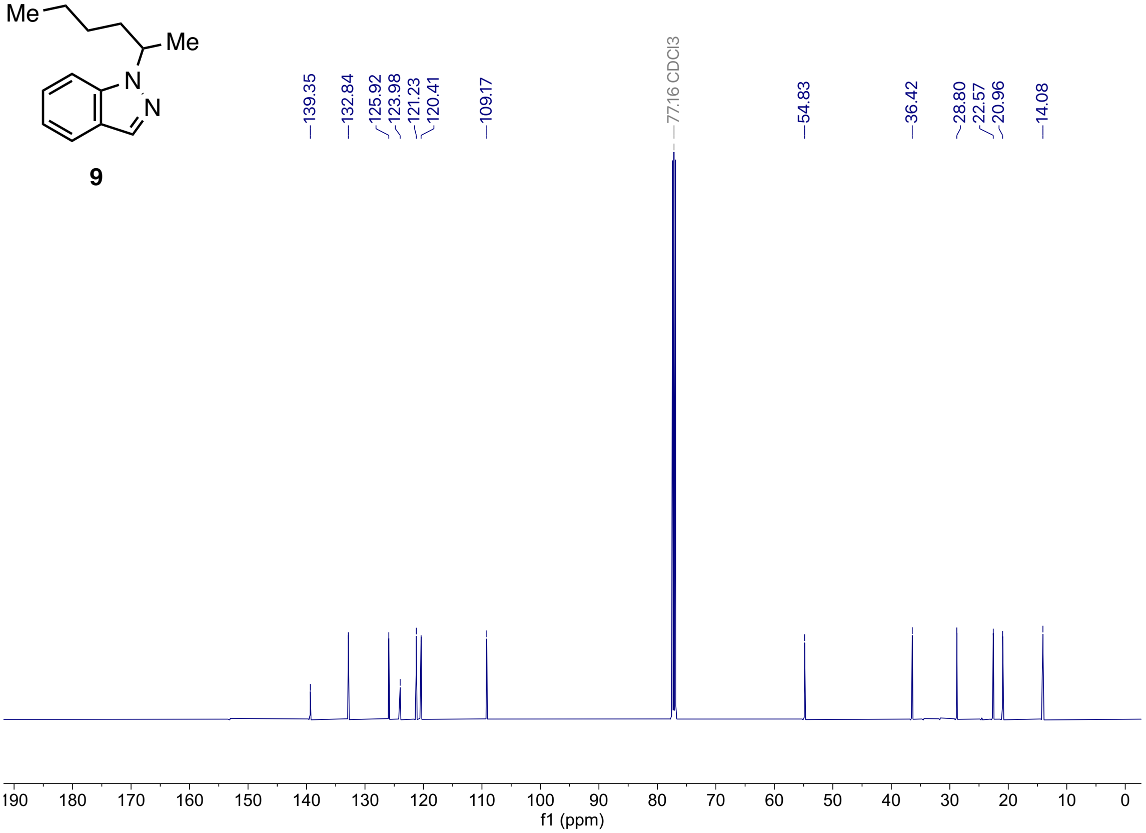

9

^{13}C NMR spectrum of **9** taken in $CDCl_3$.

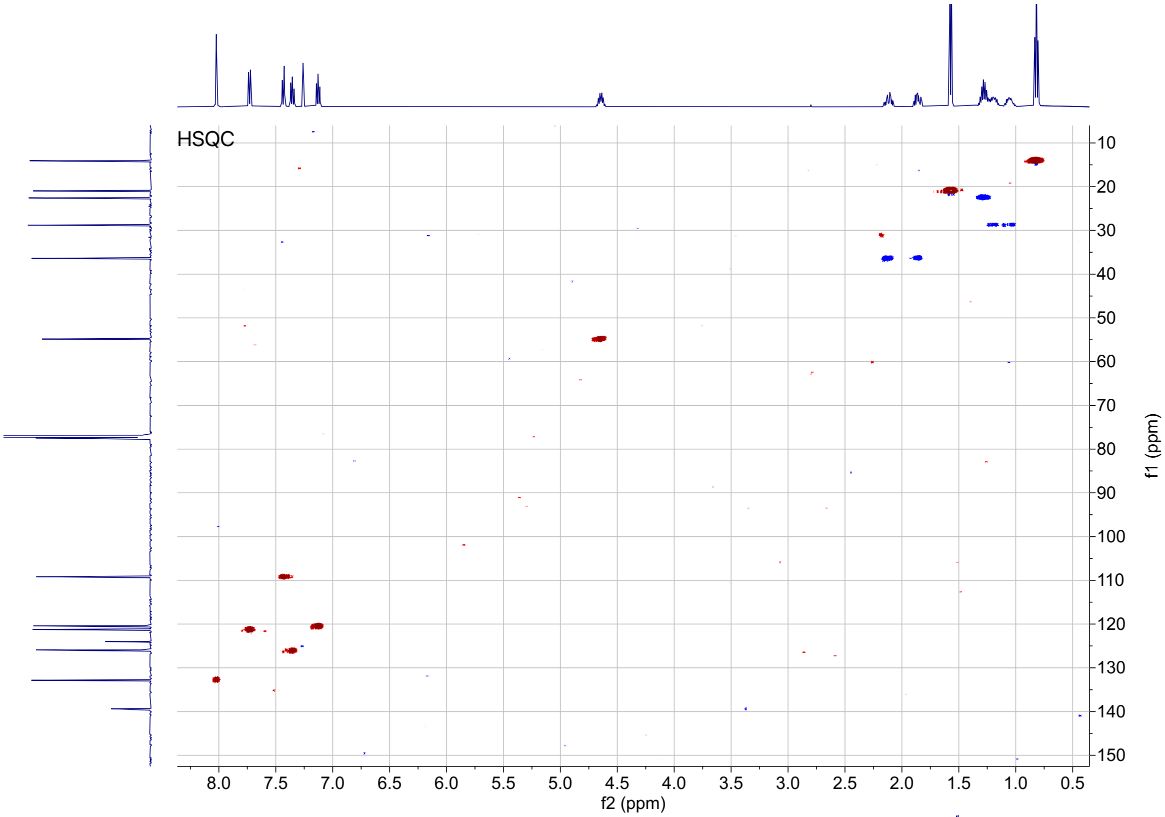

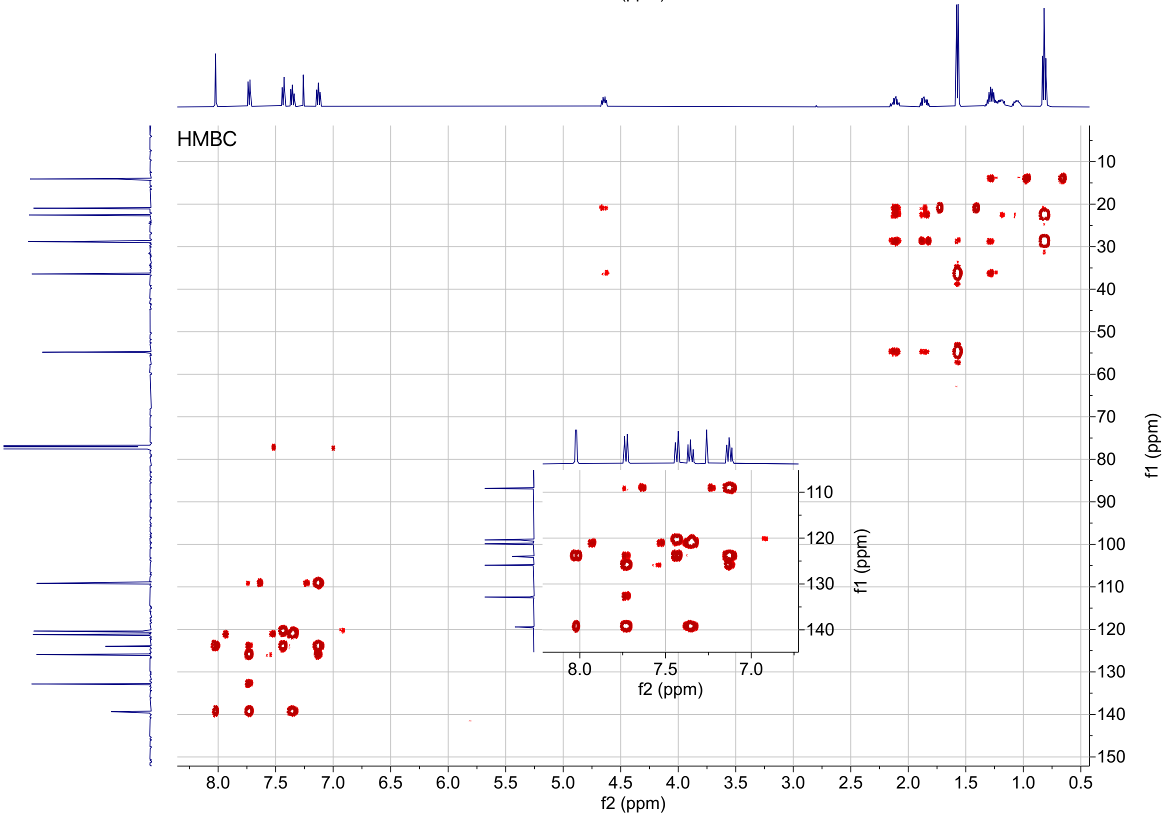

NOESY correlation between proton at 7.4 ppm and proton at 4.6 ppm supports N1 isomer.

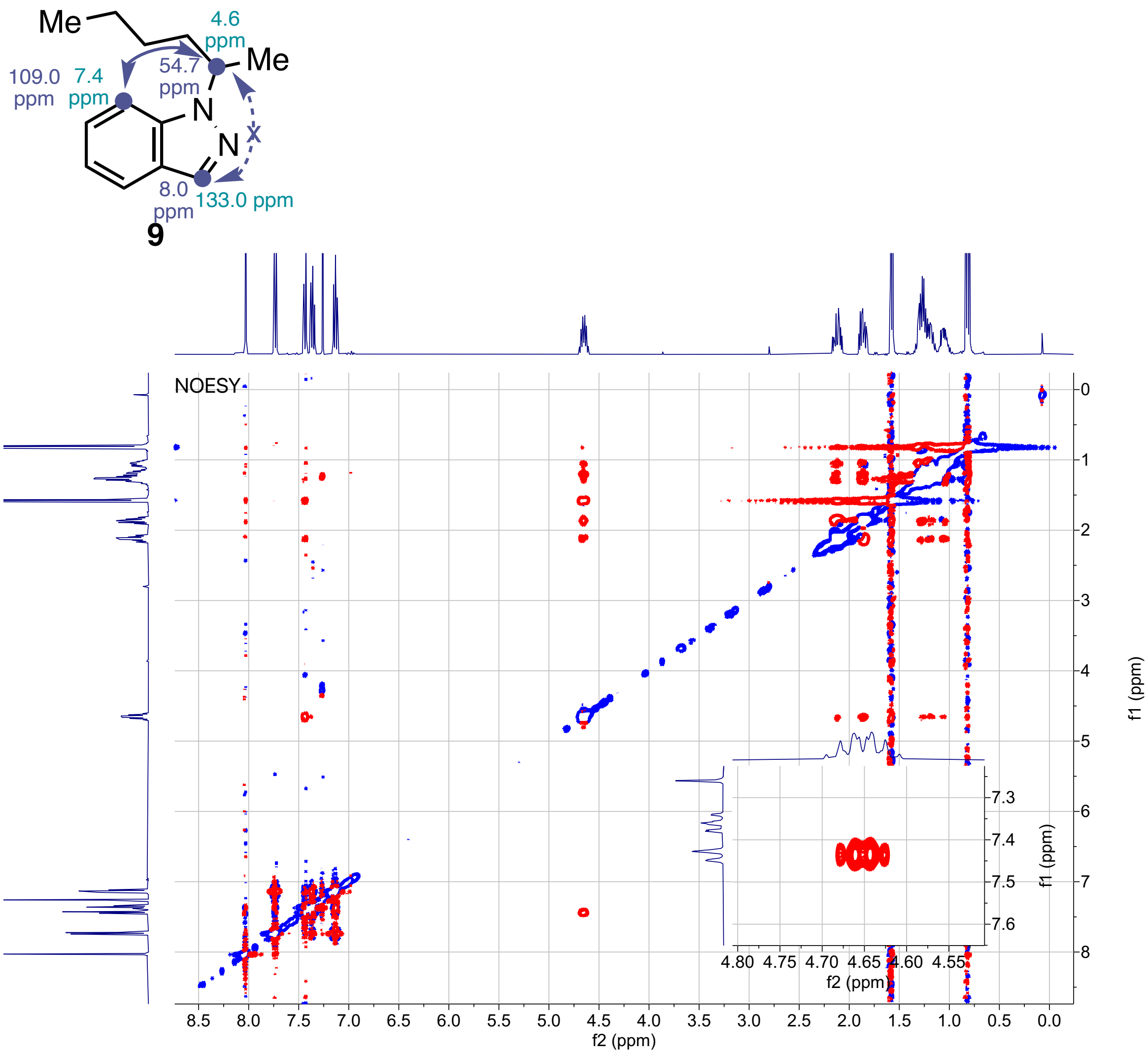
Me
Me
4.6 ppm
54.7 ppm
109.0 ppm
7.4 ppm
N
N
8.0 ppm
133.0 ppm
9
NOESY
f2 (ppm)
f1 (ppm)

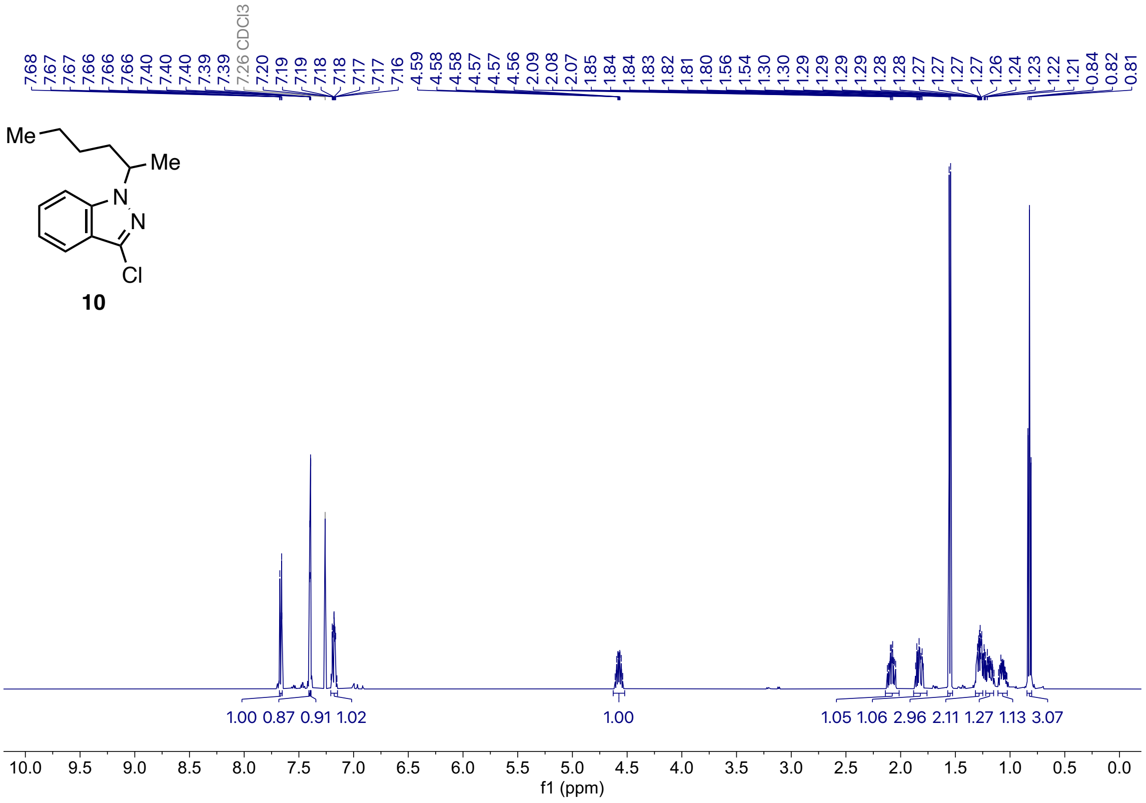

^{1}H NMR spectrum of **10** taken in $CDCl_3$.

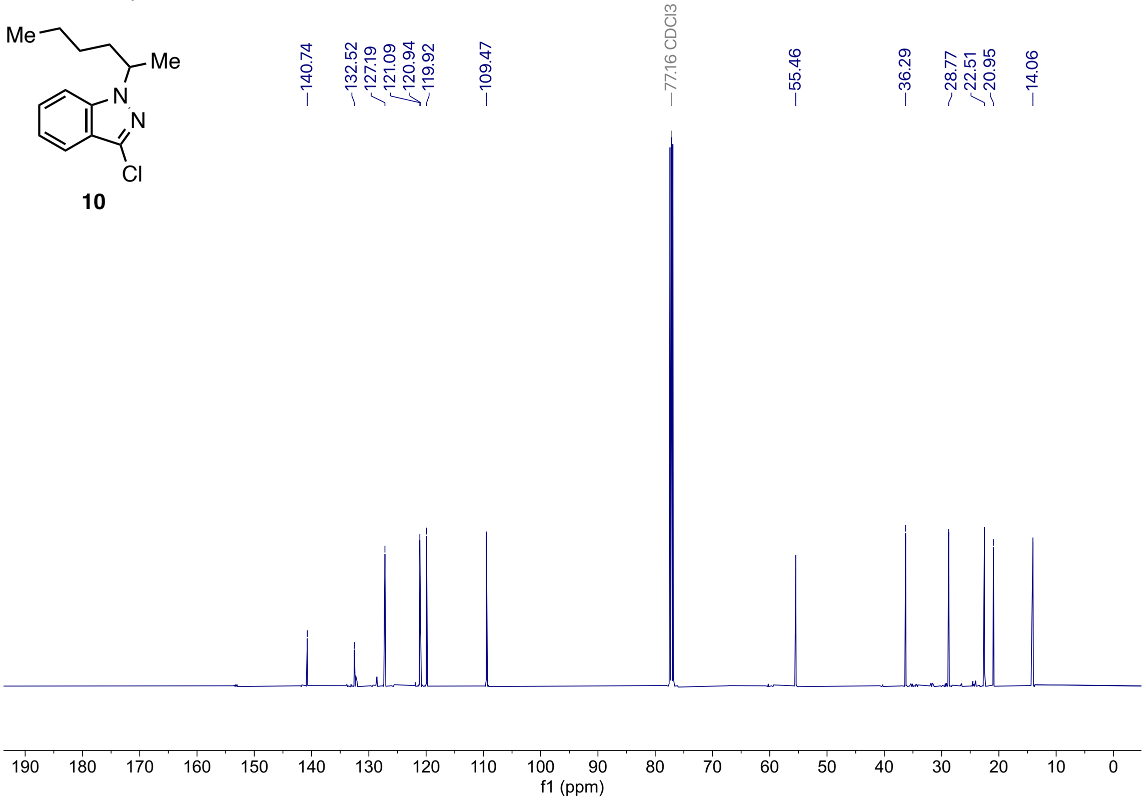

^{13}C NMR spectrum of **10** taken in $CDCl_3$.

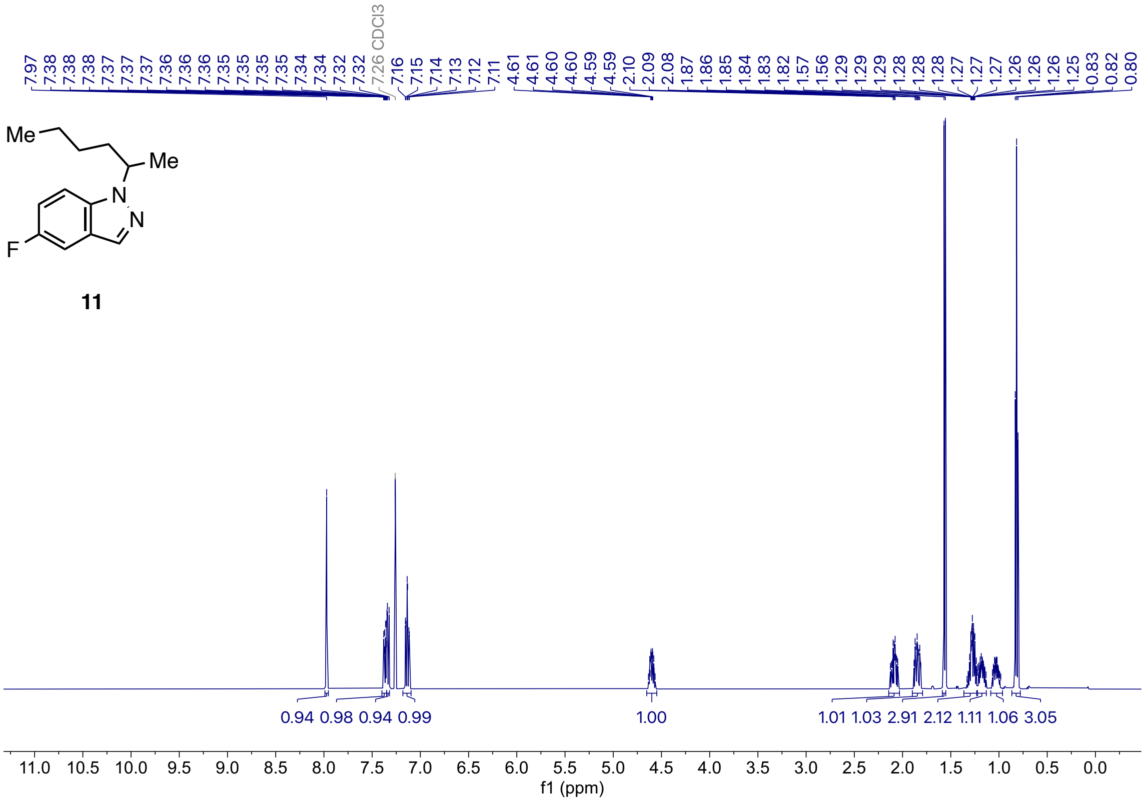

^{1}H NMR spectrum of **11** taken in $CDCl_3$.

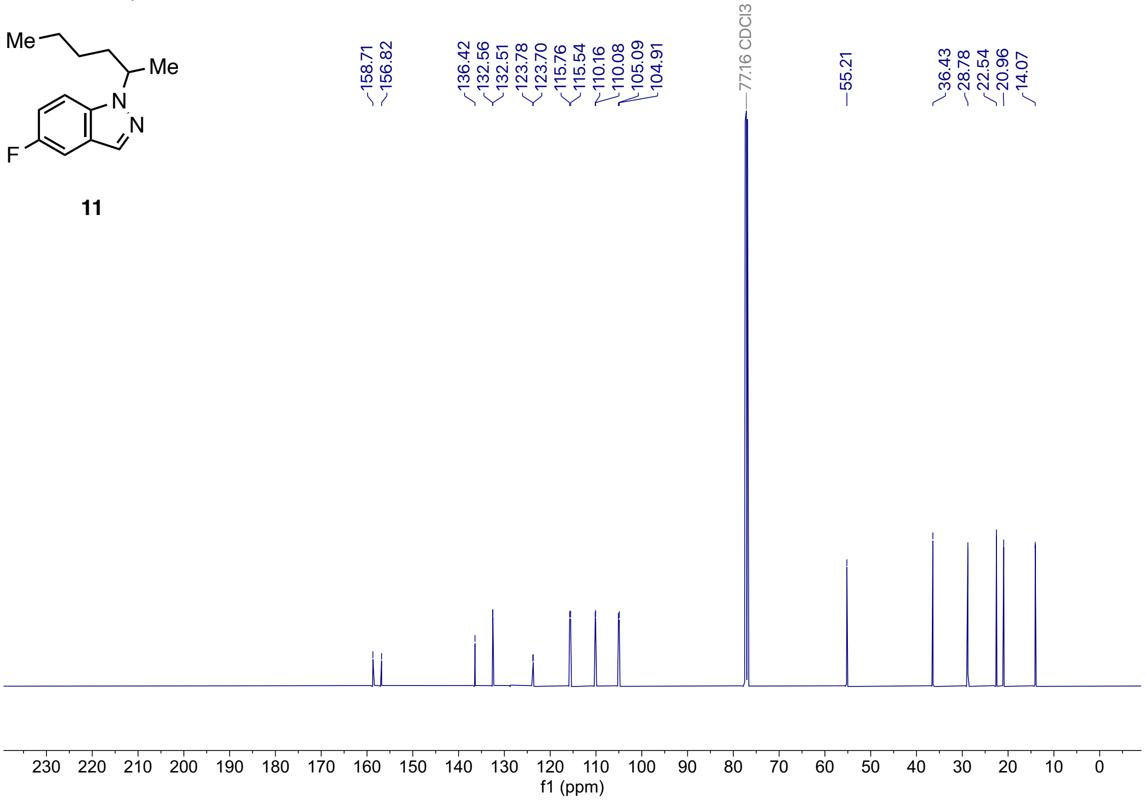

^{13}C NMR spectrum of **11** taken in $CDCl_3$.

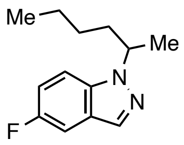

11

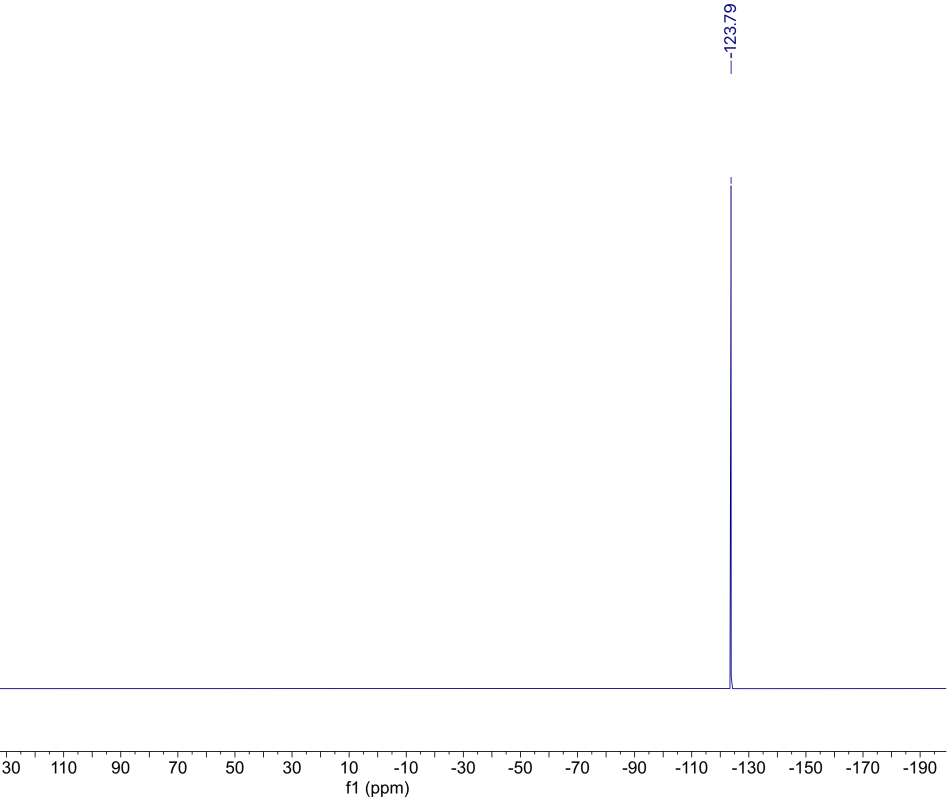

$^{19}F\{^{1}H\}$ NMR spectrum of **11** taken in $CDCl_3$.

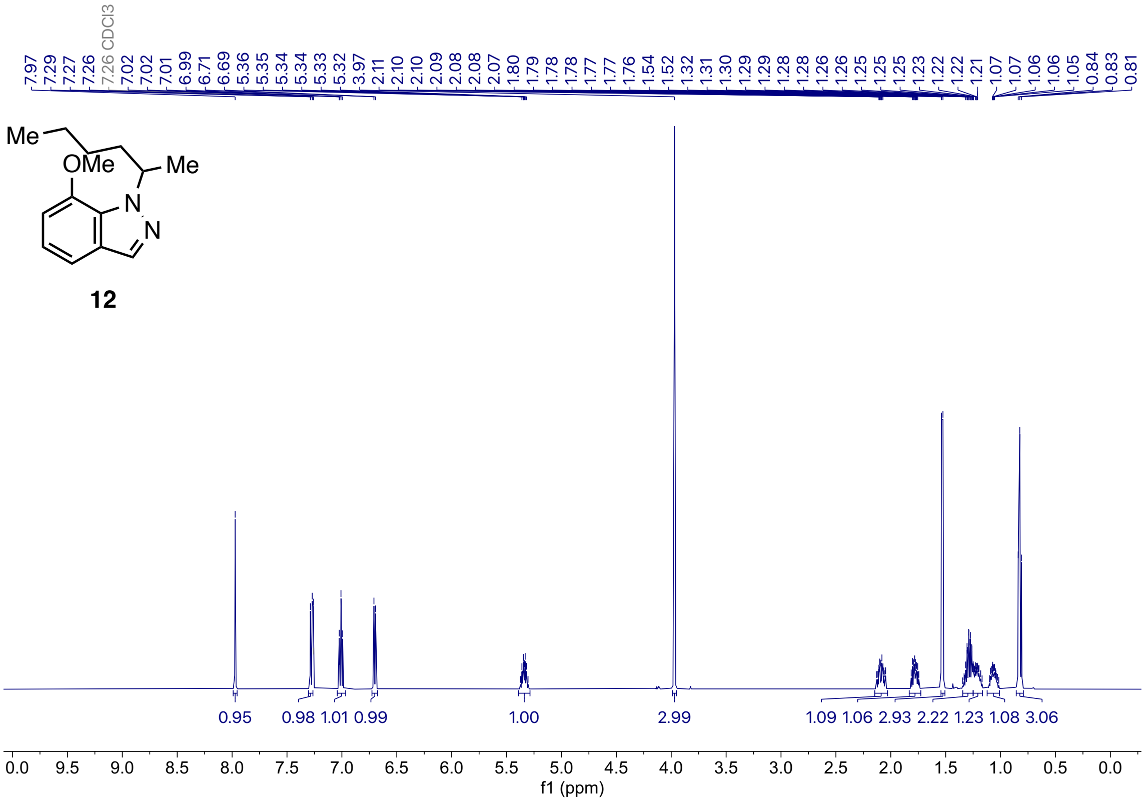

^{1}H NMR spectrum of **12** taken in $CDCl_3$.

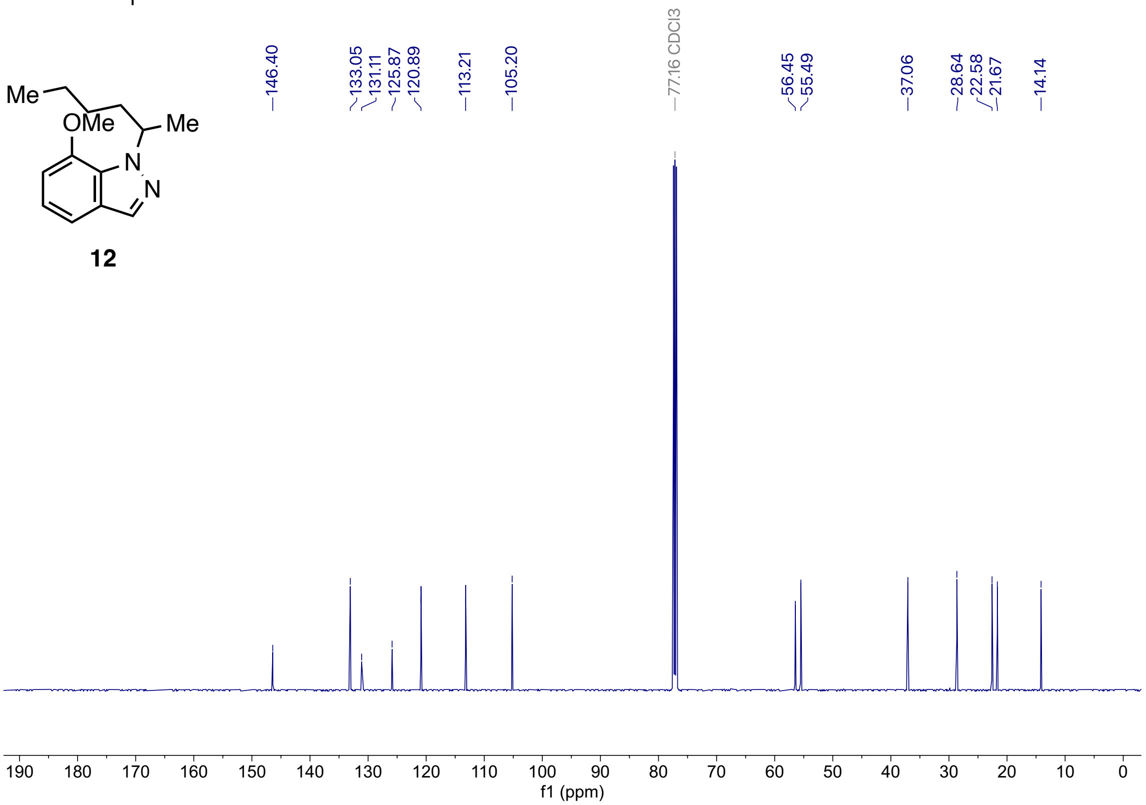

^{13}C NMR spectrum of **12** taken in $CDCl_3$.

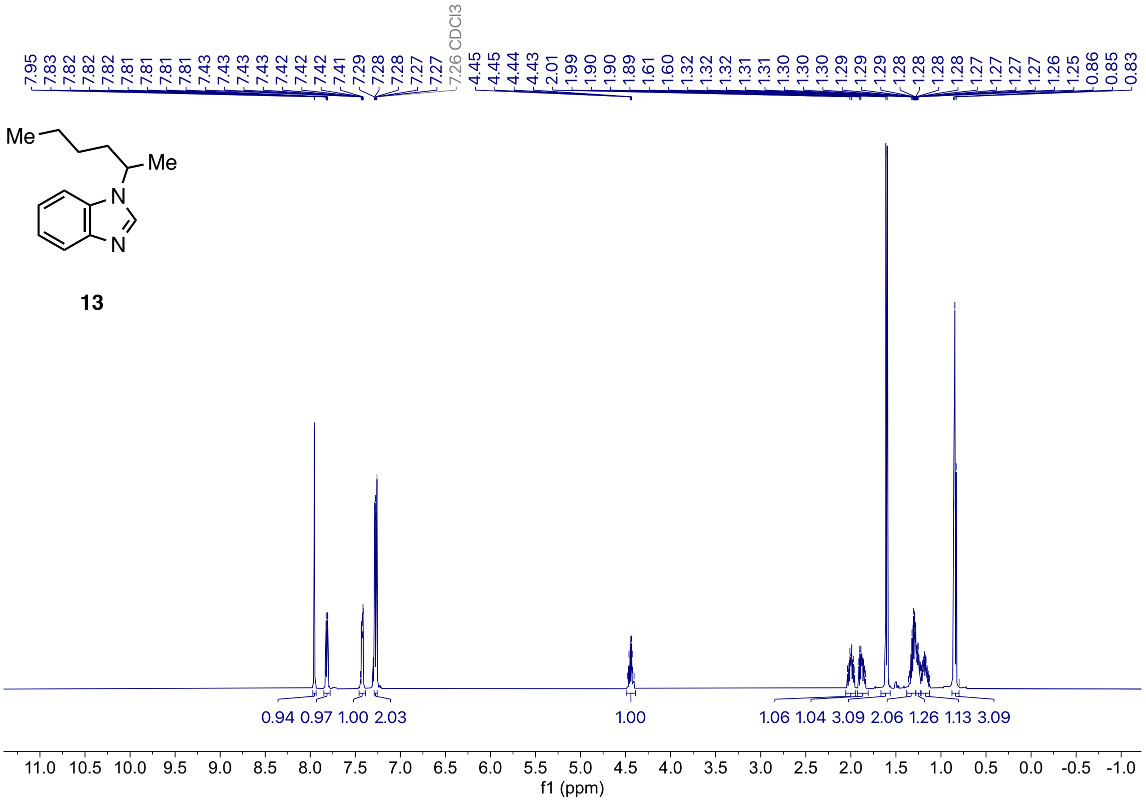

^{1}H NMR spectrum of **13** taken in $CDCl_3$.

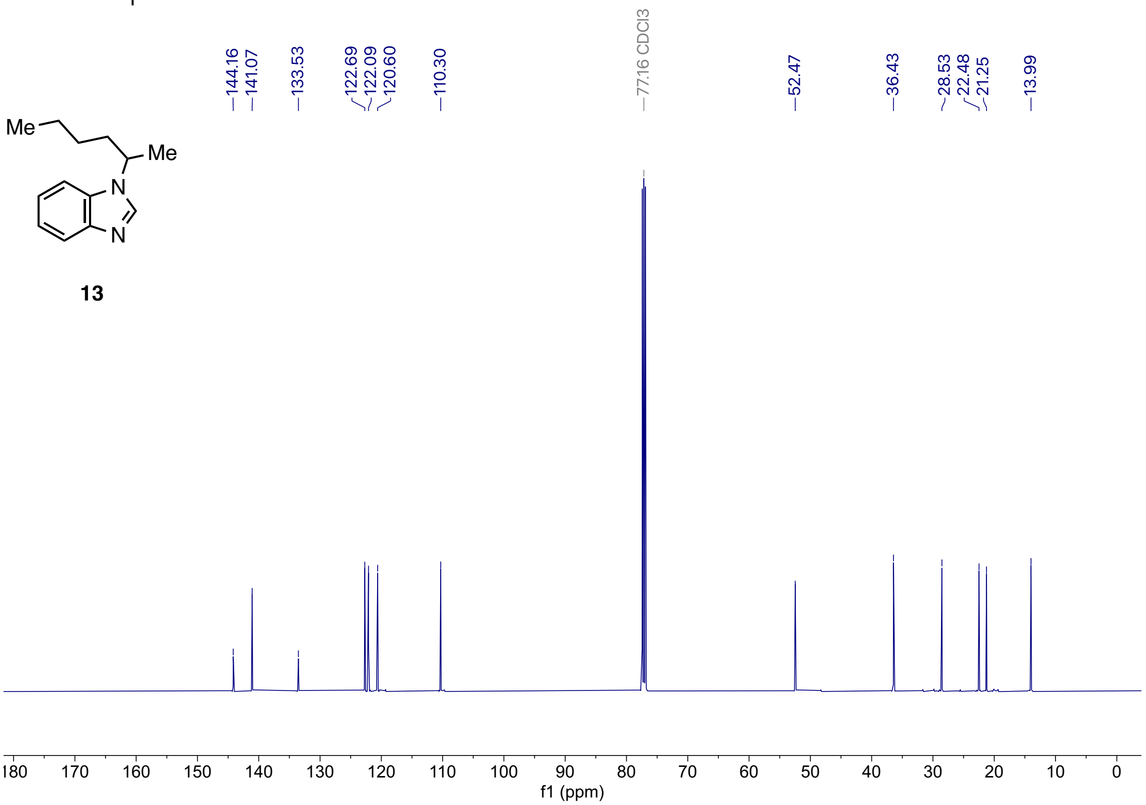

^{13}C NMR spectrum of **13** taken in $CDCl_3$.

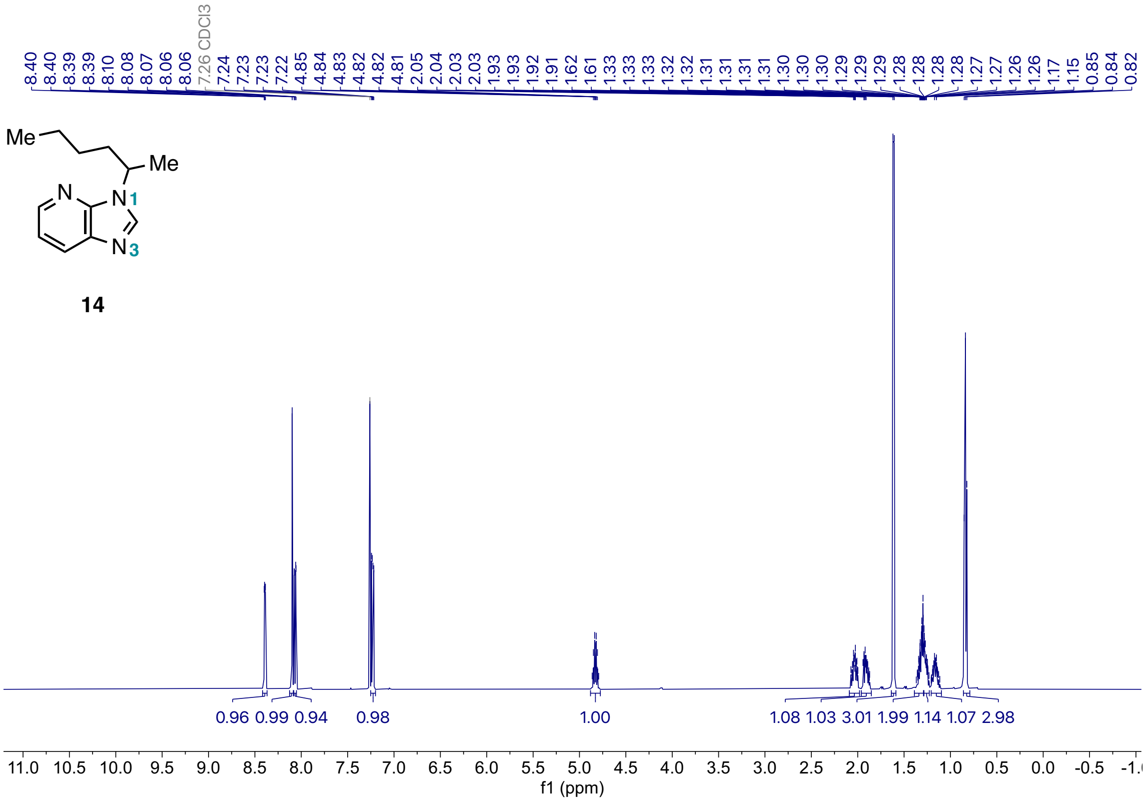

^{1}H NMR spectrum of **14** taken in $CDCl_3$.

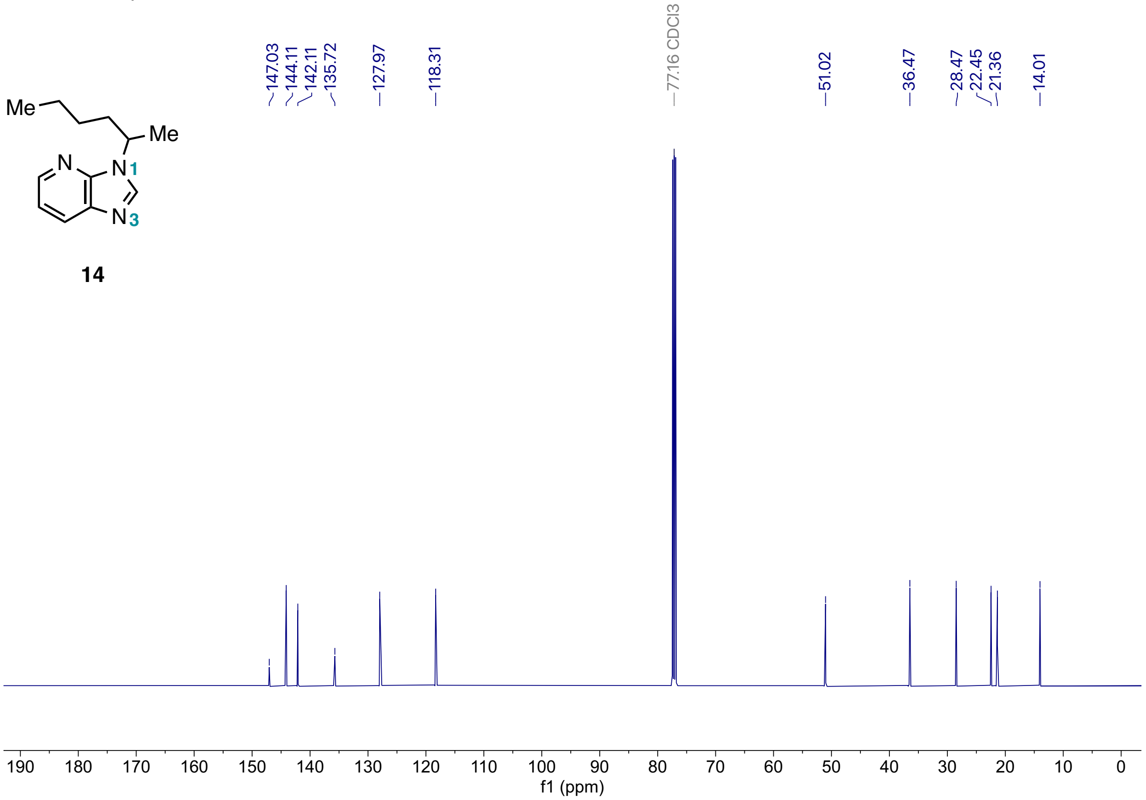

^{13}C NMR spectrum of **14** taken in $CDCl_3$.

HMBC correlation between ^{13}C peak at 142.1 ppm and proton at 4.8 ppm, and absence of correlation between other aromatic ^{13}C peaks and proton at 4.8 ppm supports N1 isomer.

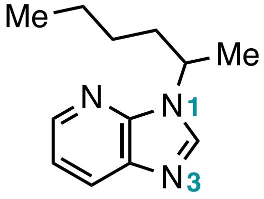

14

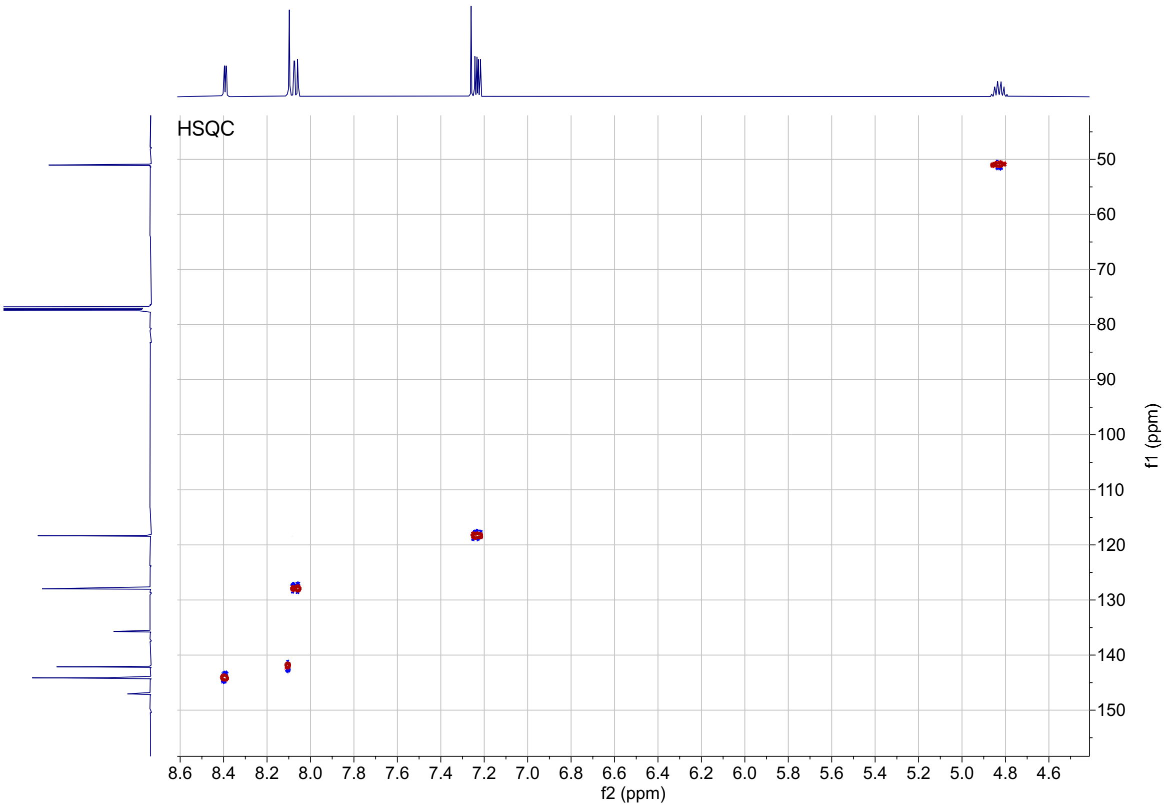

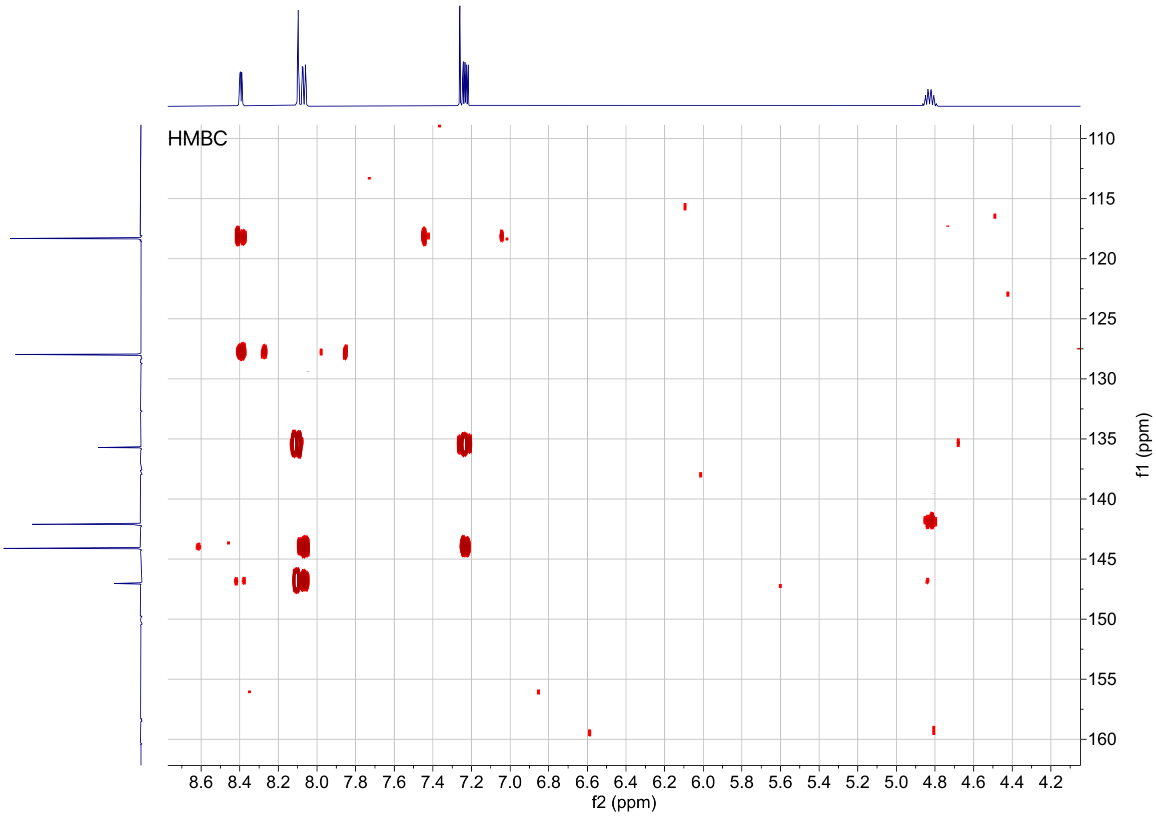
HMBC
f1 (ppm)
f2 (ppm)

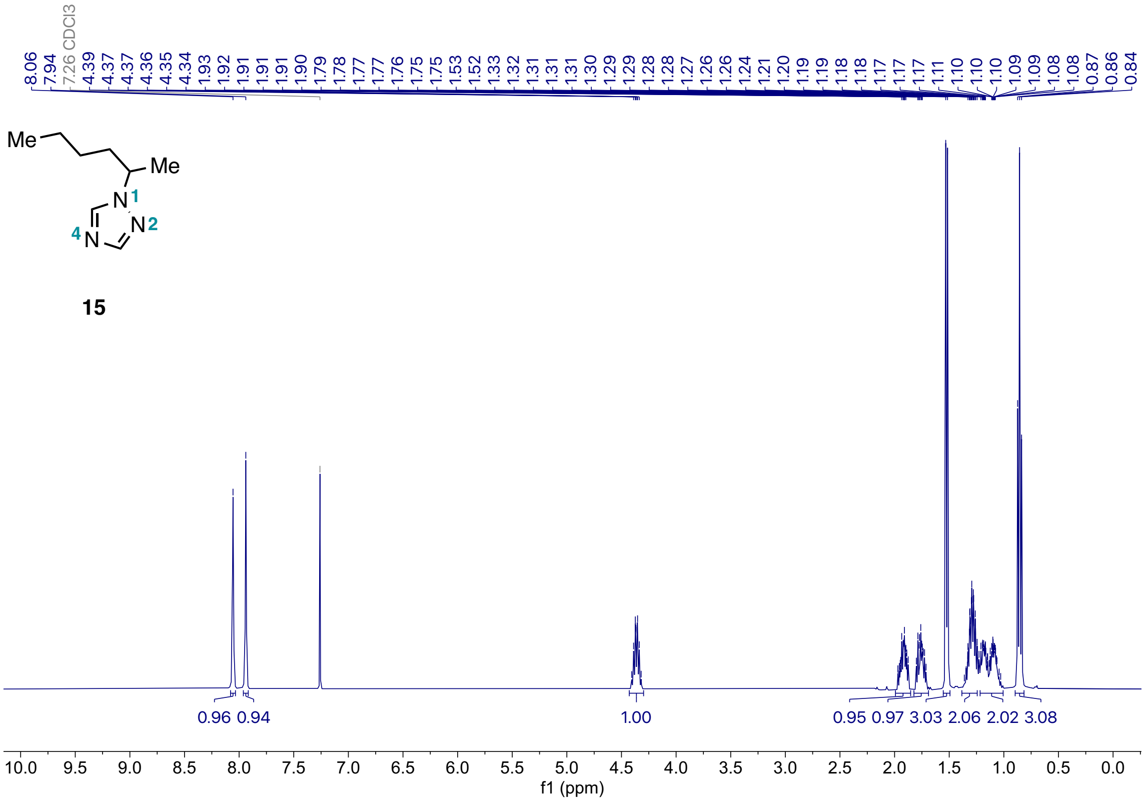

^{1}H NMR spectrum of **15** taken in $CDCl_3$.

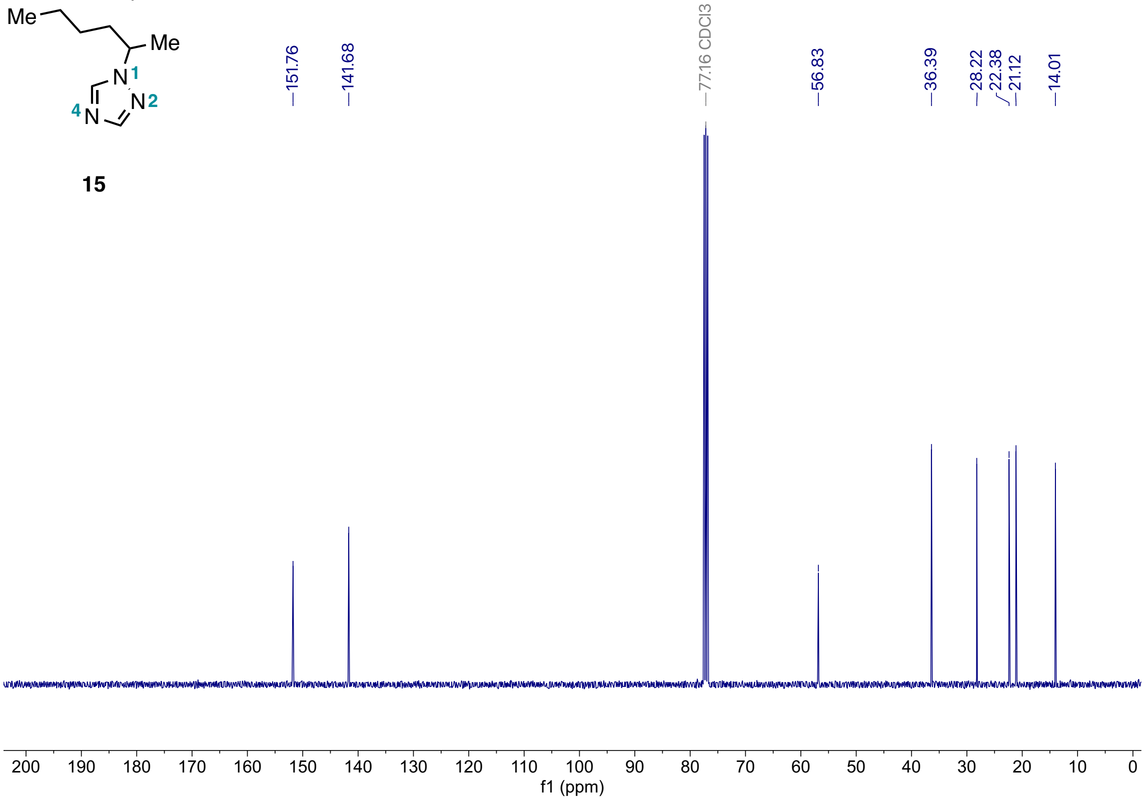

^{13}C NMR spectrum of **15** taken in $CDCl_3$.

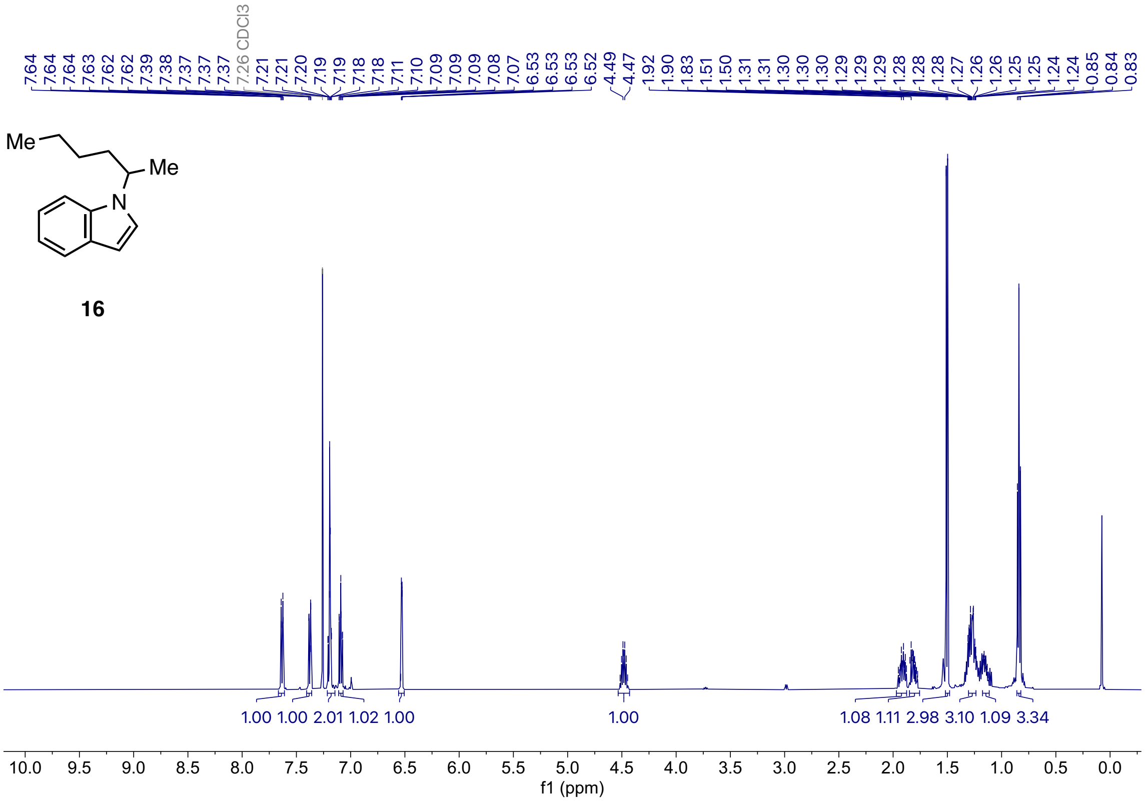

^1H NMR spectrum of **16** taken in $CDCl_3$.

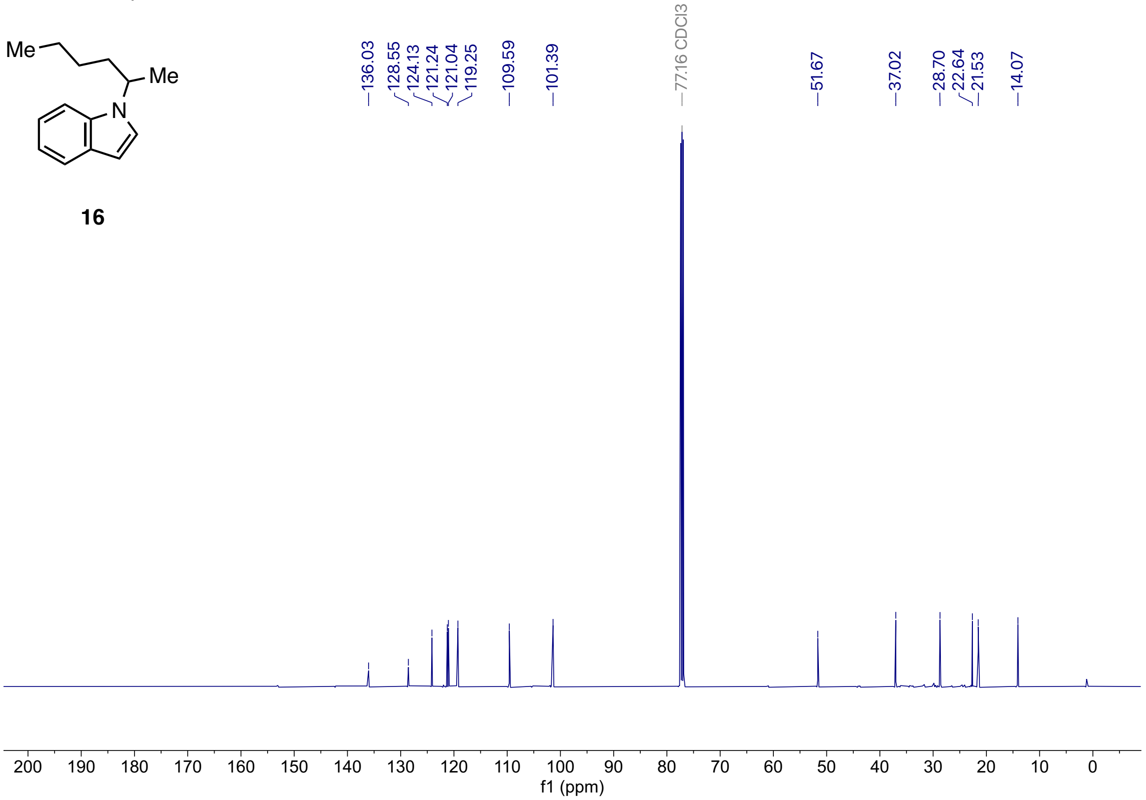

^{13}C NMR spectrum of **16** taken in $CDCl_3$.

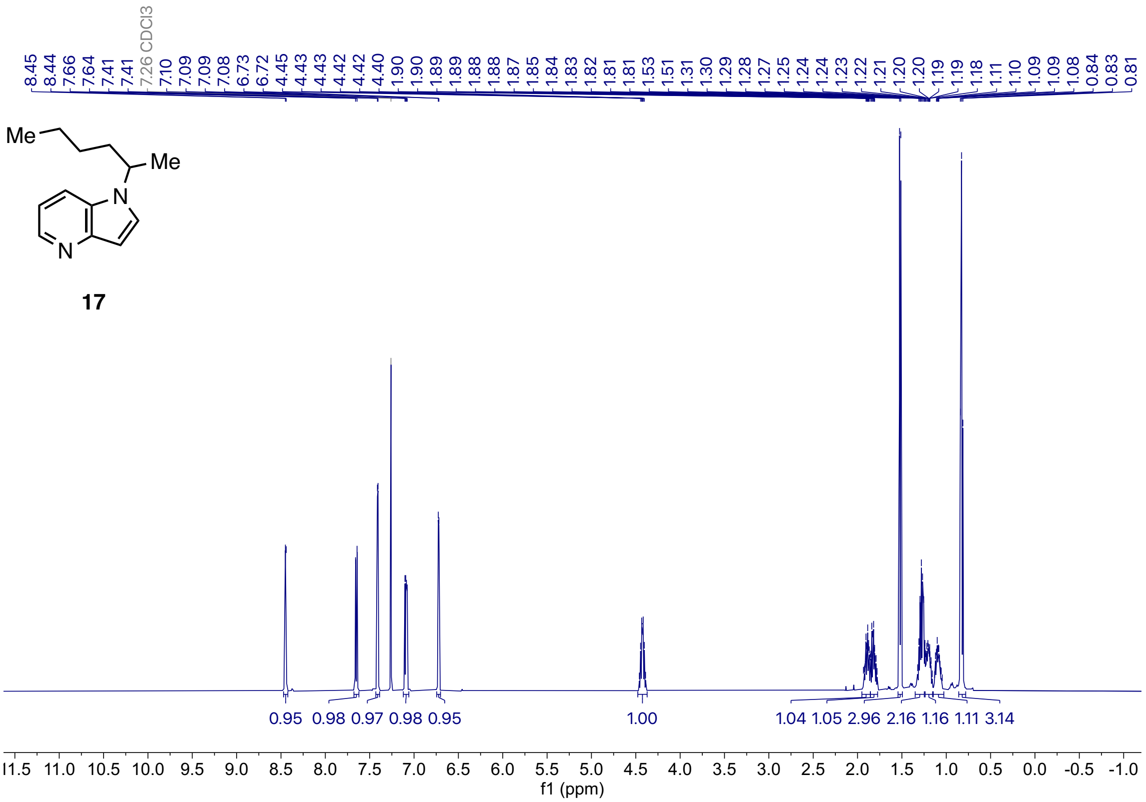

^{1}H NMR spectrum of **17** taken in $CDCl_3$.

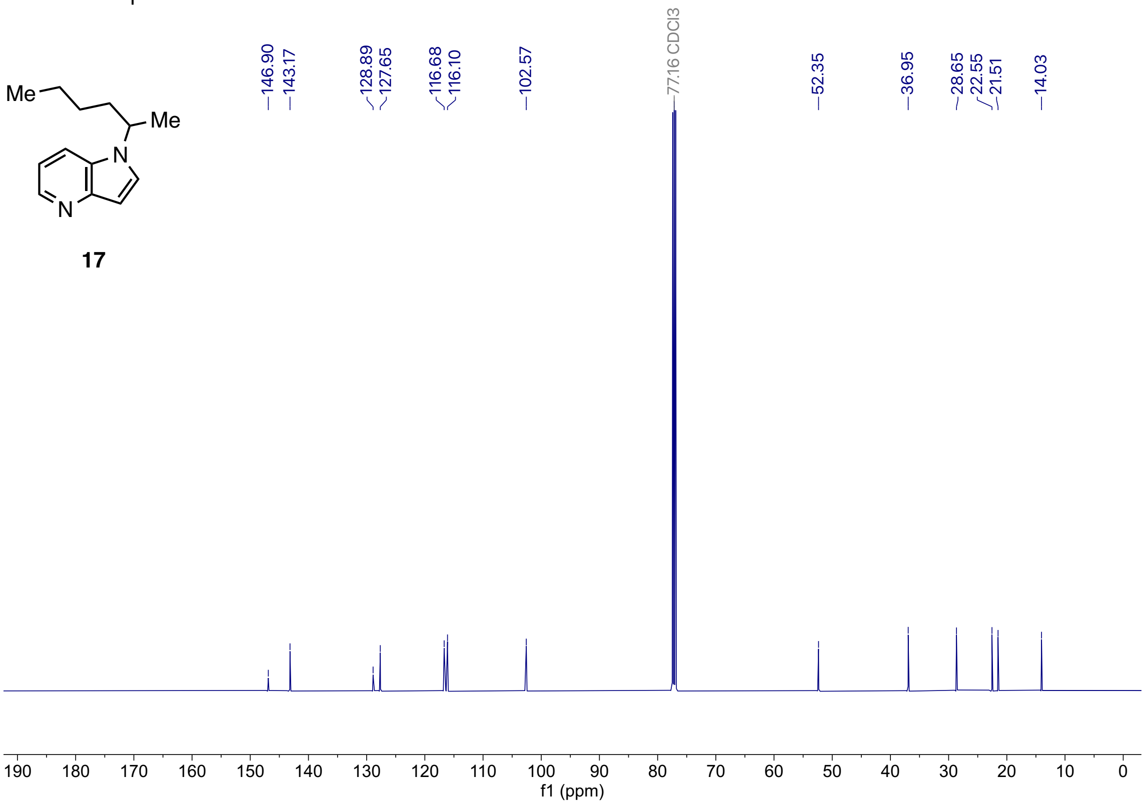

^{13}C NMR spectrum of **17** taken in $CDCl_3$.

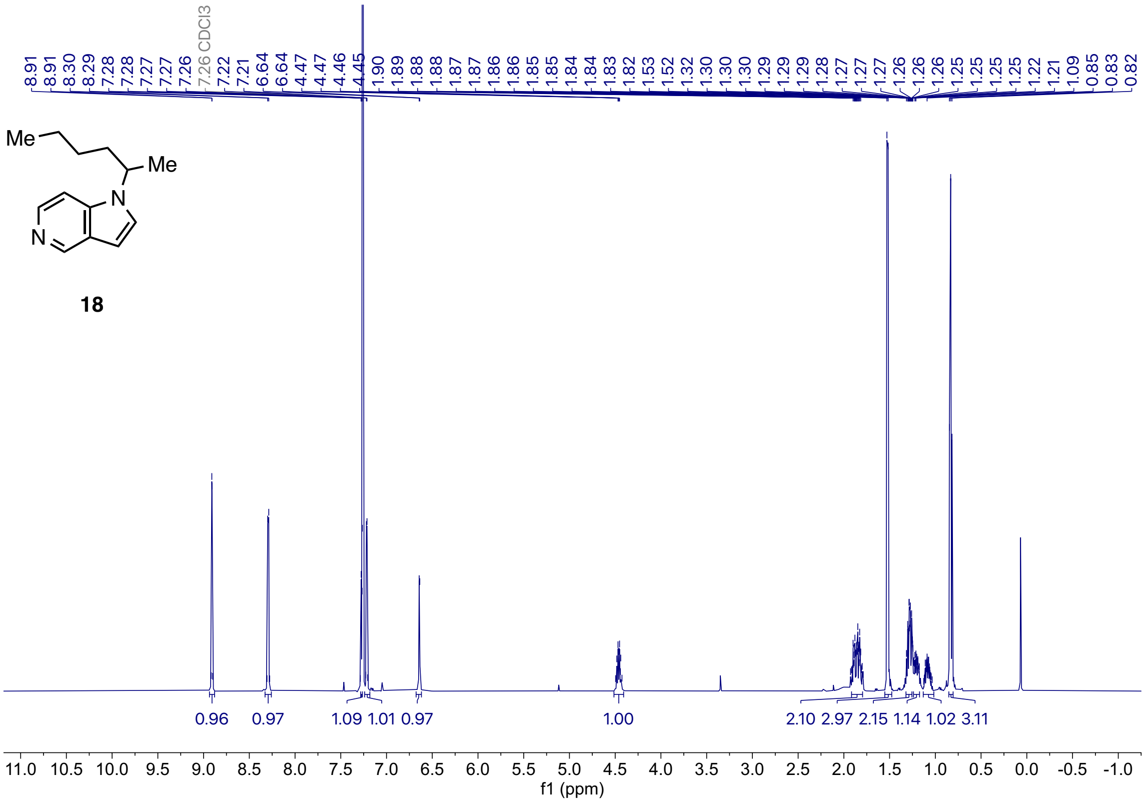

^{1}H NMR spectrum of **18** taken in $CDCl_3$.

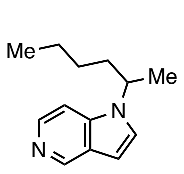

18

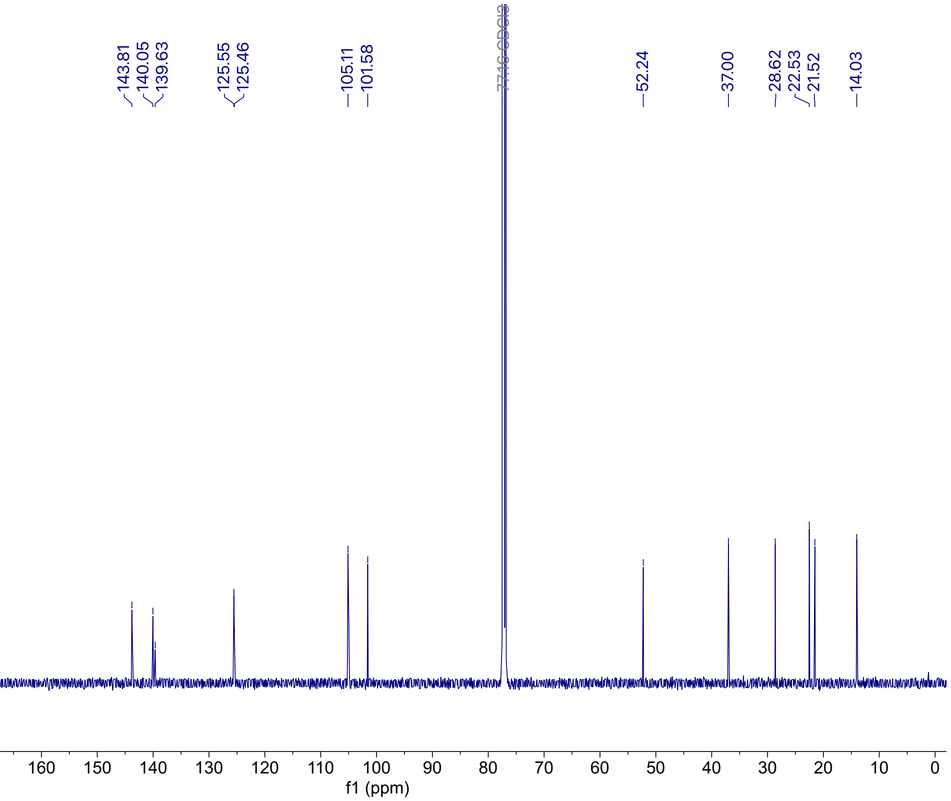

^{13}C NMR spectrum of **18** taken in $CDCl_3$.

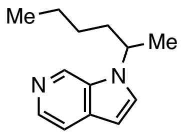

19

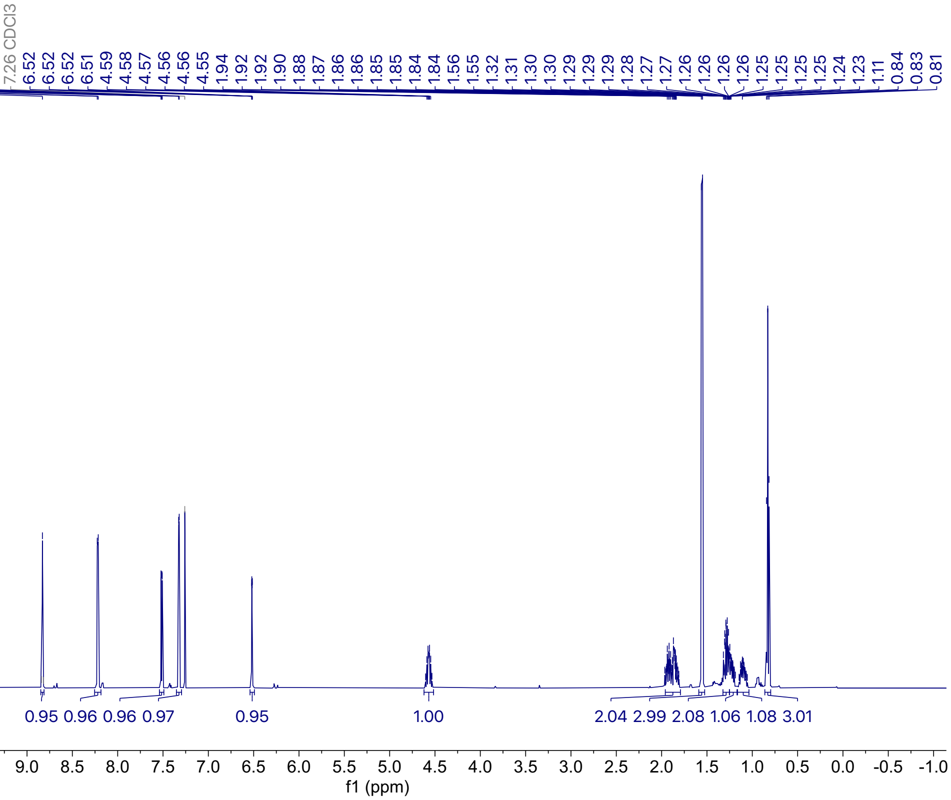

^{1}H NMR spectrum of **19** taken in $CDCl_3$.

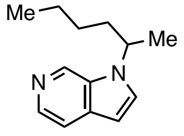

19

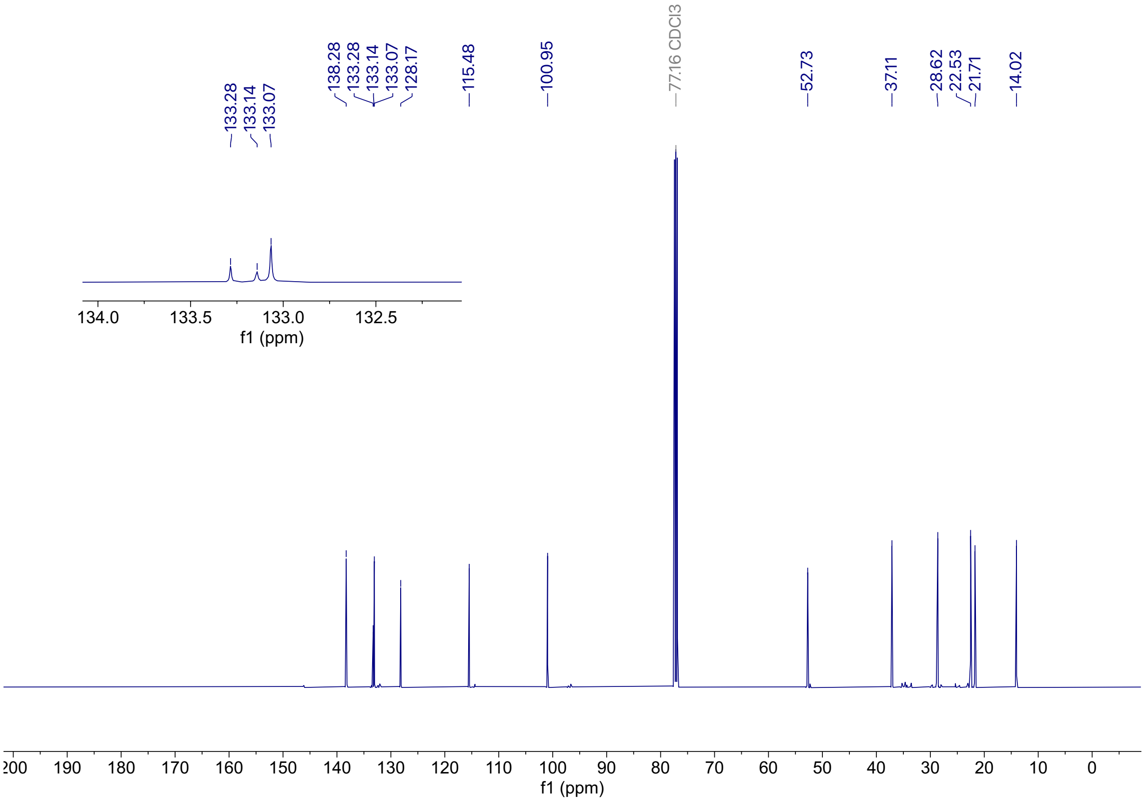

^{13}C NMR spectrum of **19** taken in $CDCl_3$.

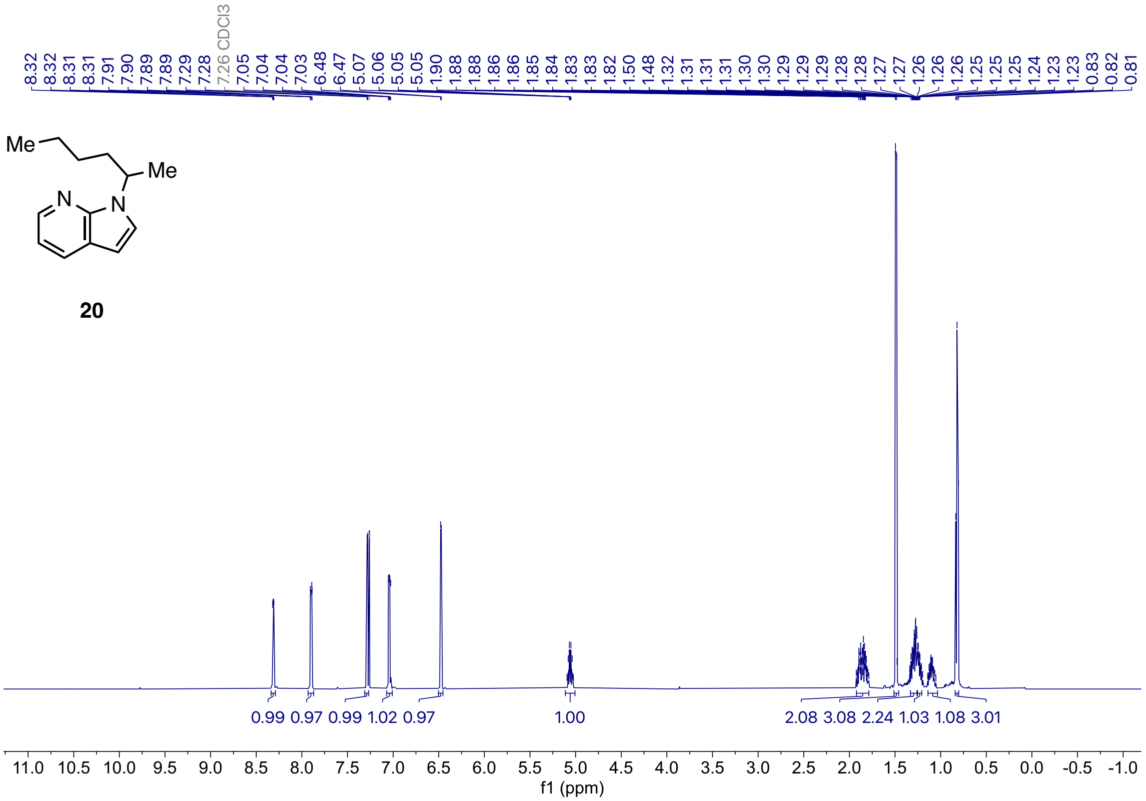

^{1}H NMR spectrum of **20** taken in $CDCl_3$.

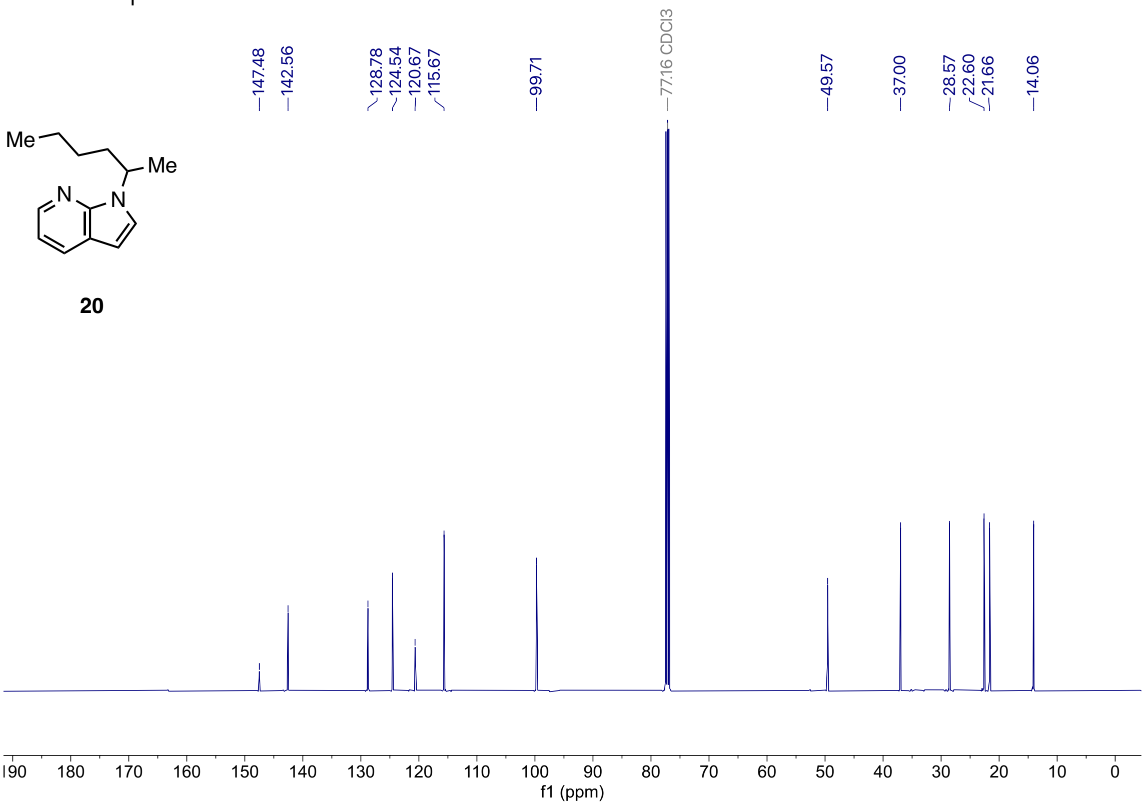

^{13}C NMR spectrum of **20** taken in $CDCl_3$.

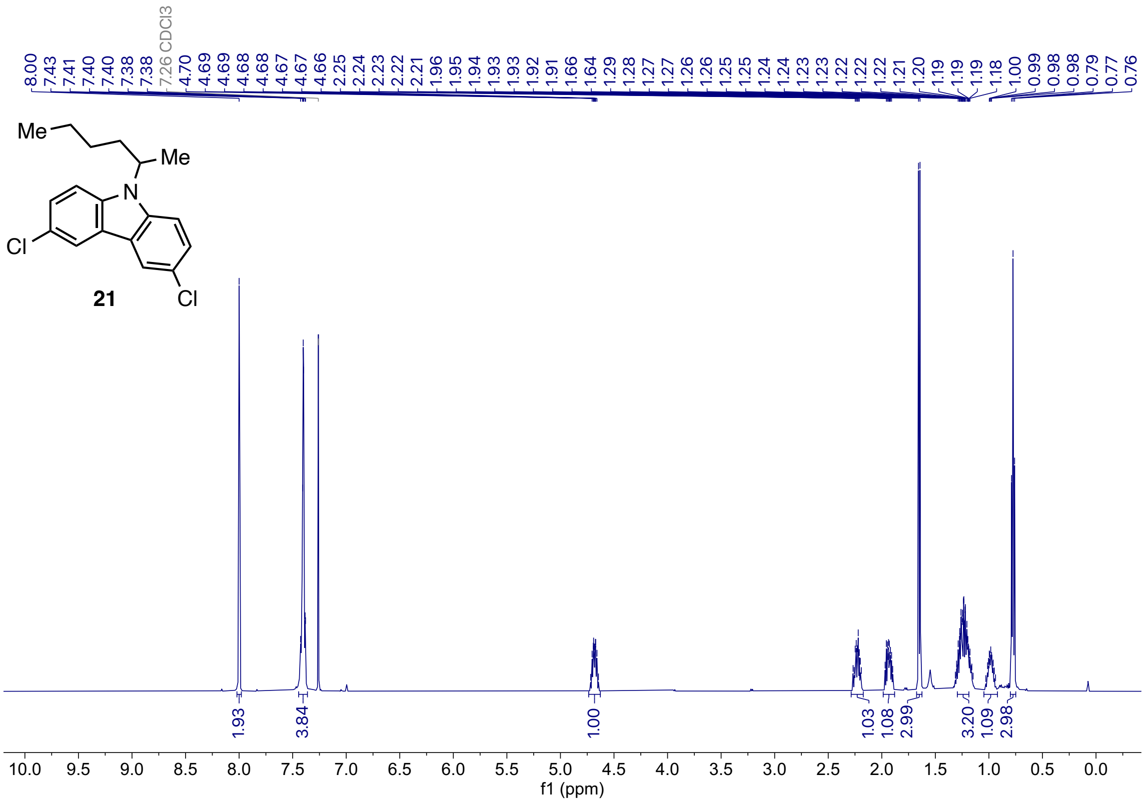

^{1}H NMR spectrum of **21** taken in $CDCl_3$.

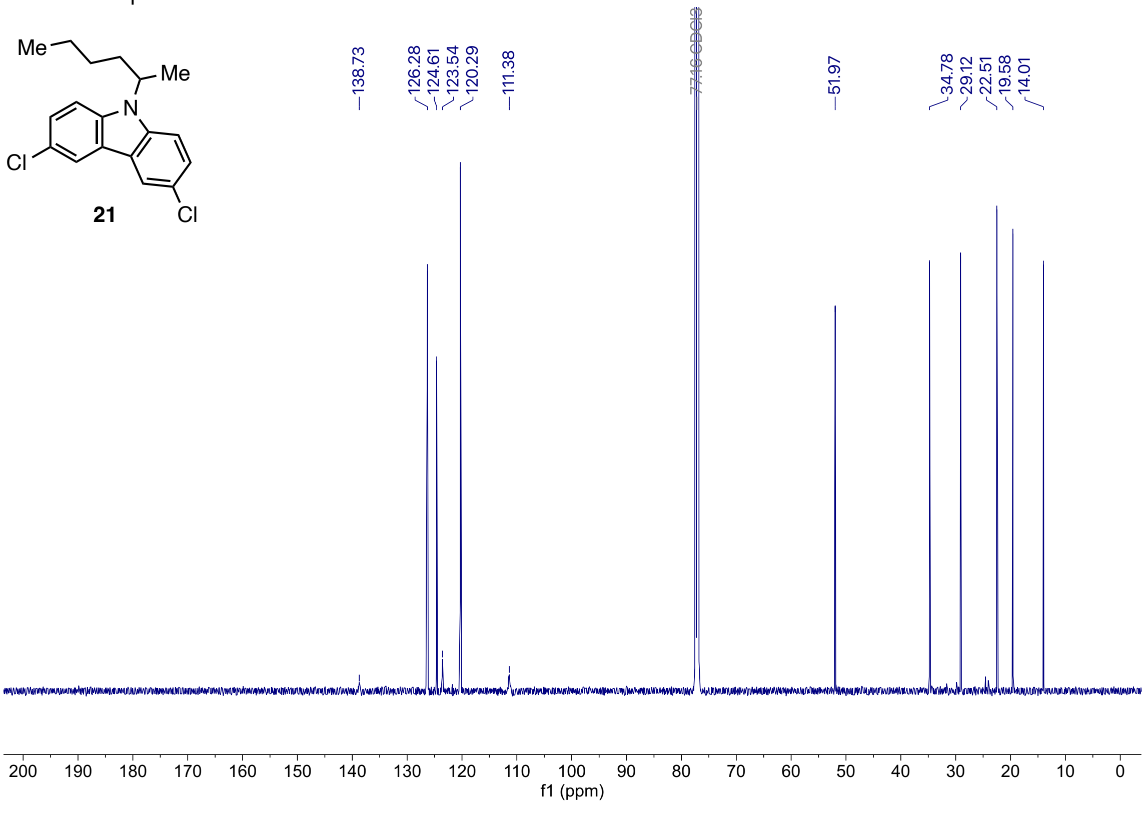

^{13}C NMR spectrum of **21** taken in $CDCl_3$.

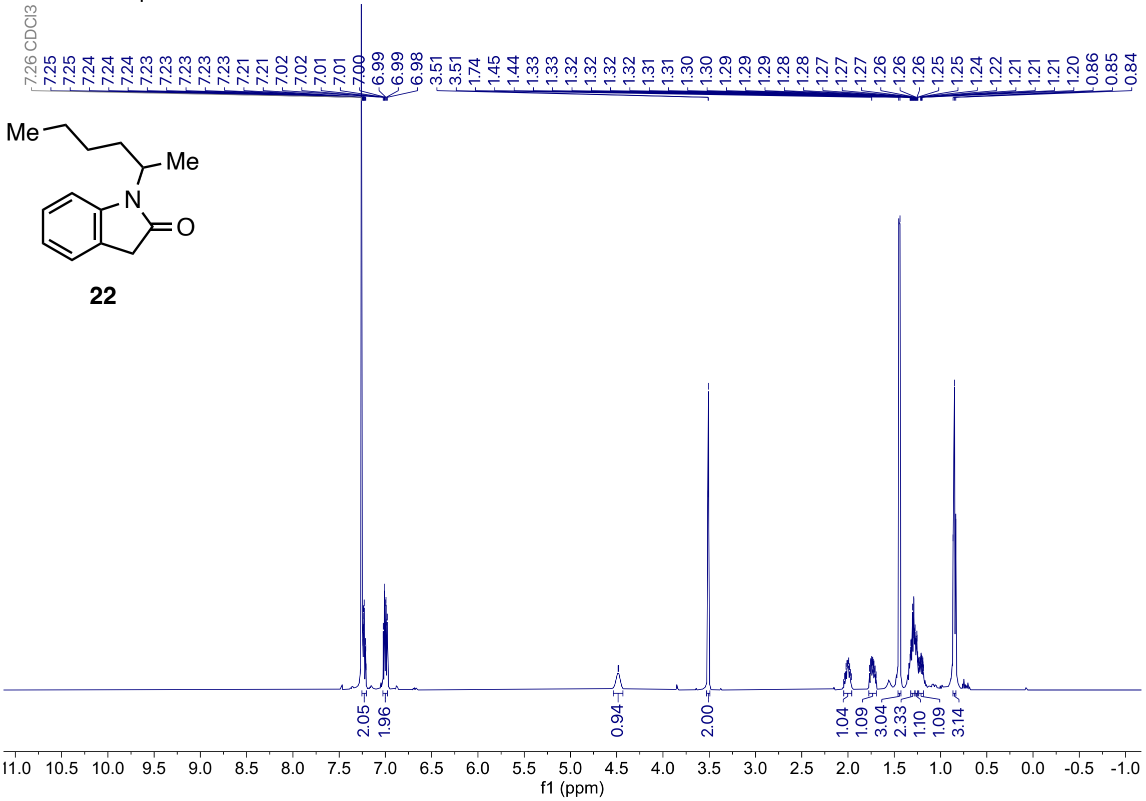

^{1}H NMR spectrum of **22** taken in $CDCl_3$.

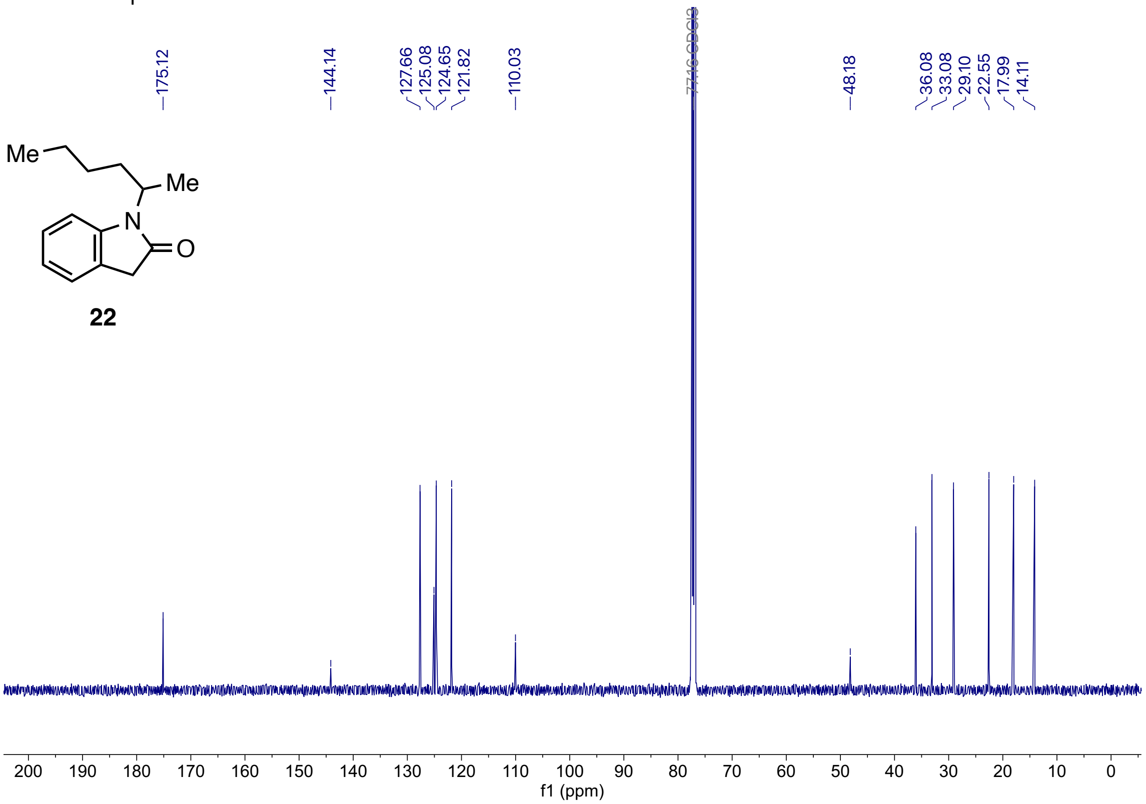

^{13}C NMR spectrum of **22** taken in $CDCl_3$.

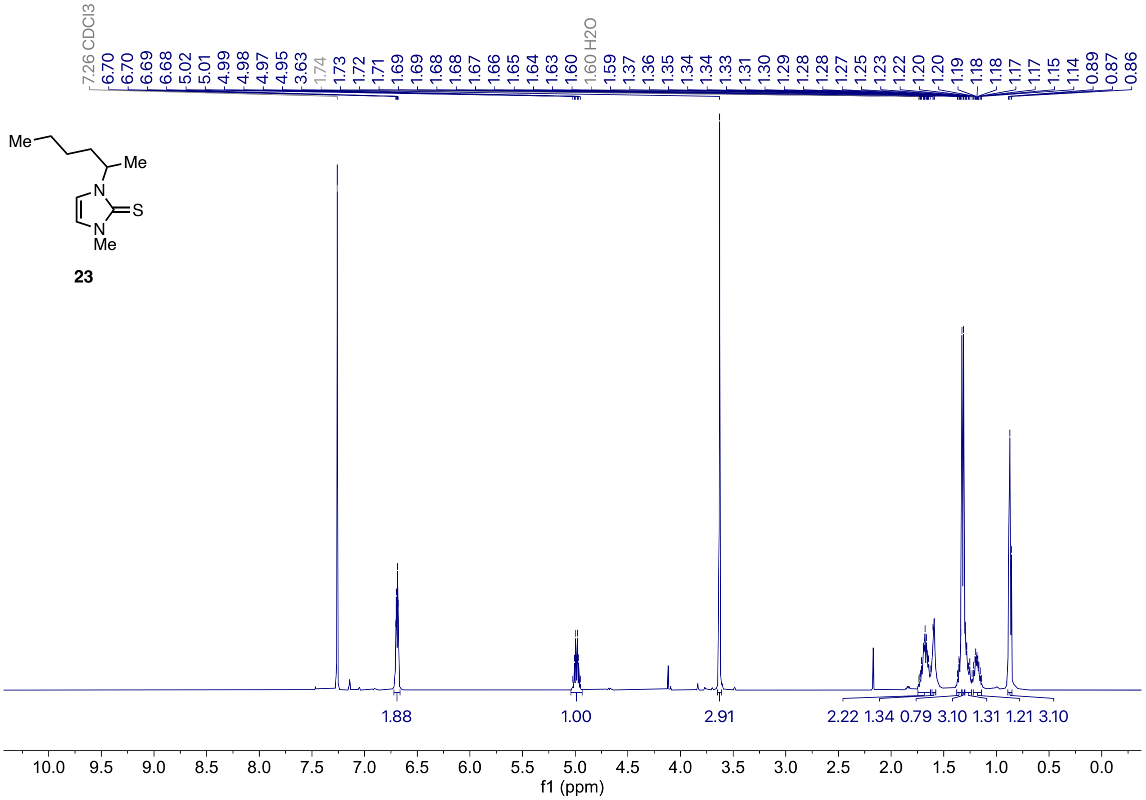

^{1}H NMR spectrum of **23** taken in $CDCl_3$.

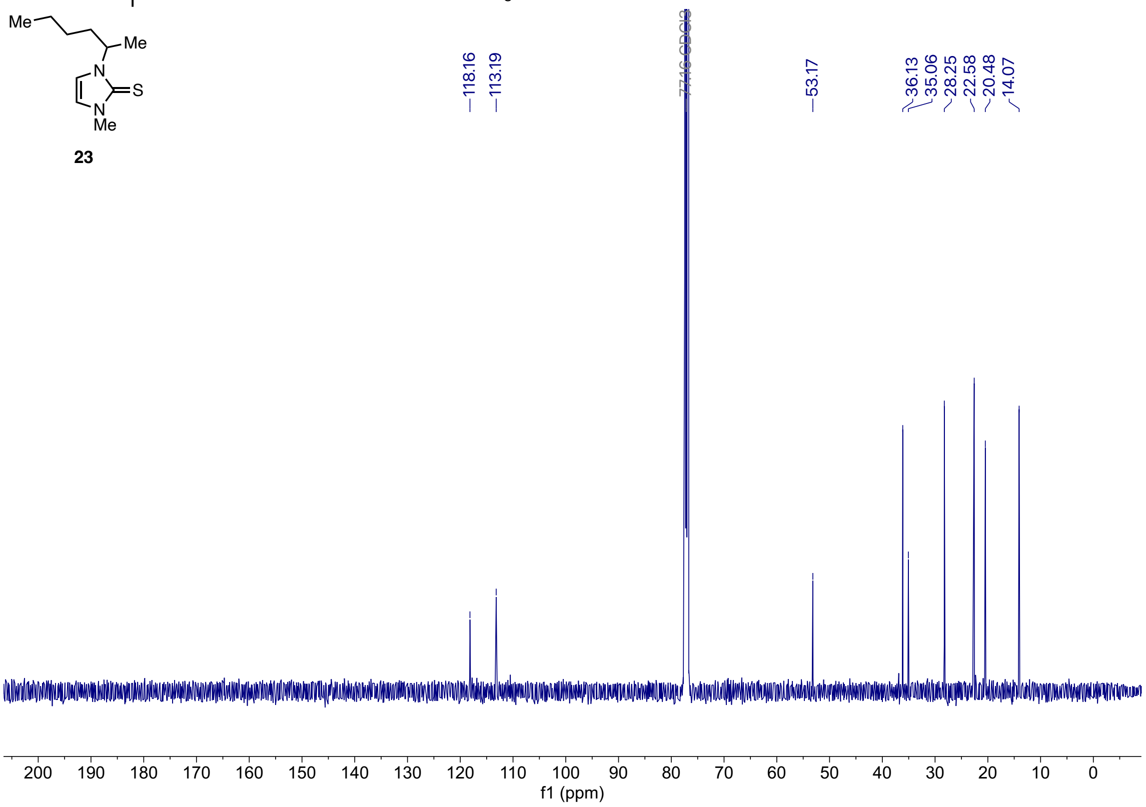

^{13}C NMR spectrum of **23** taken in $CDCl_3$.

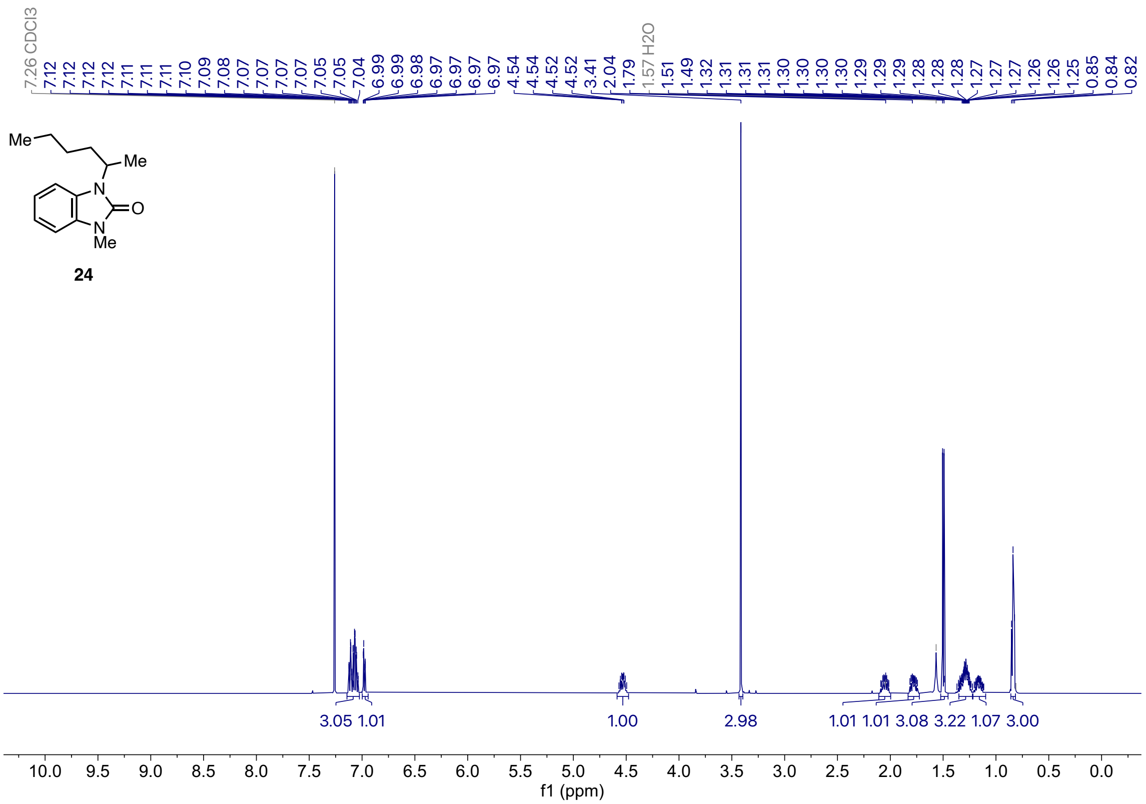

^{1}H NMR spectrum of **24** taken in $CDCl_3$.

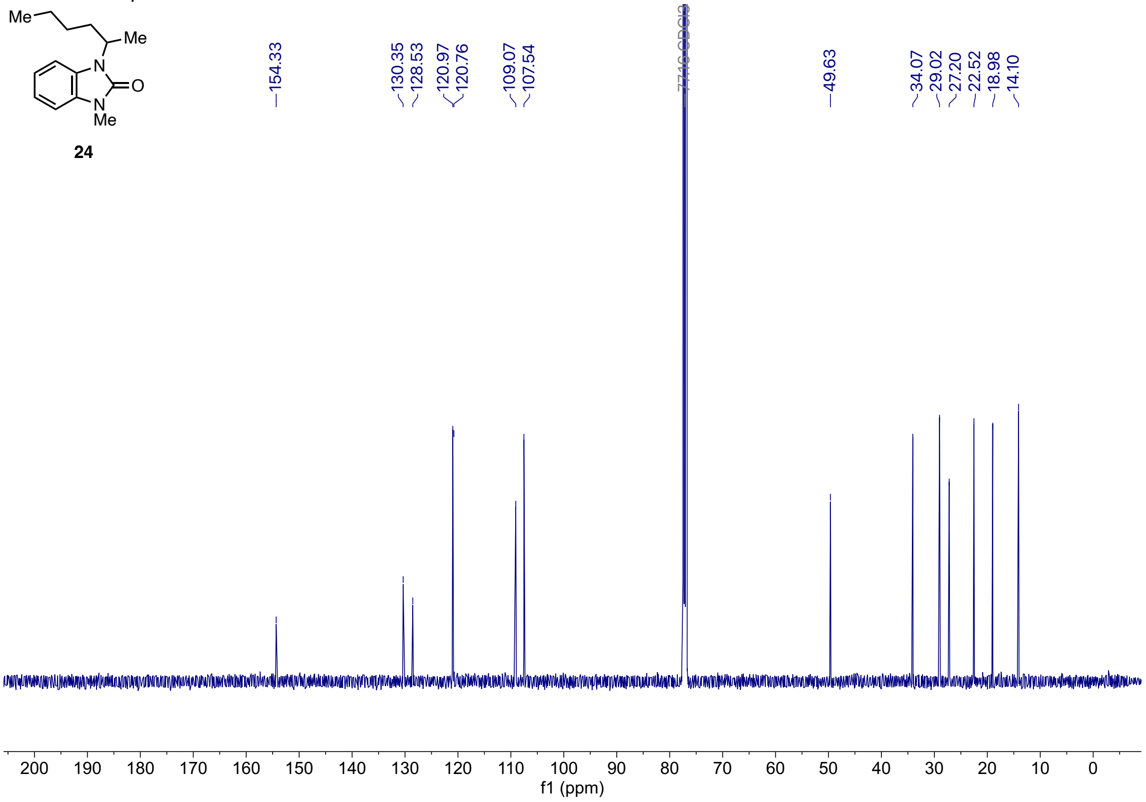

^{13}C NMR spectrum of **24** taken in $CDCl_3$.

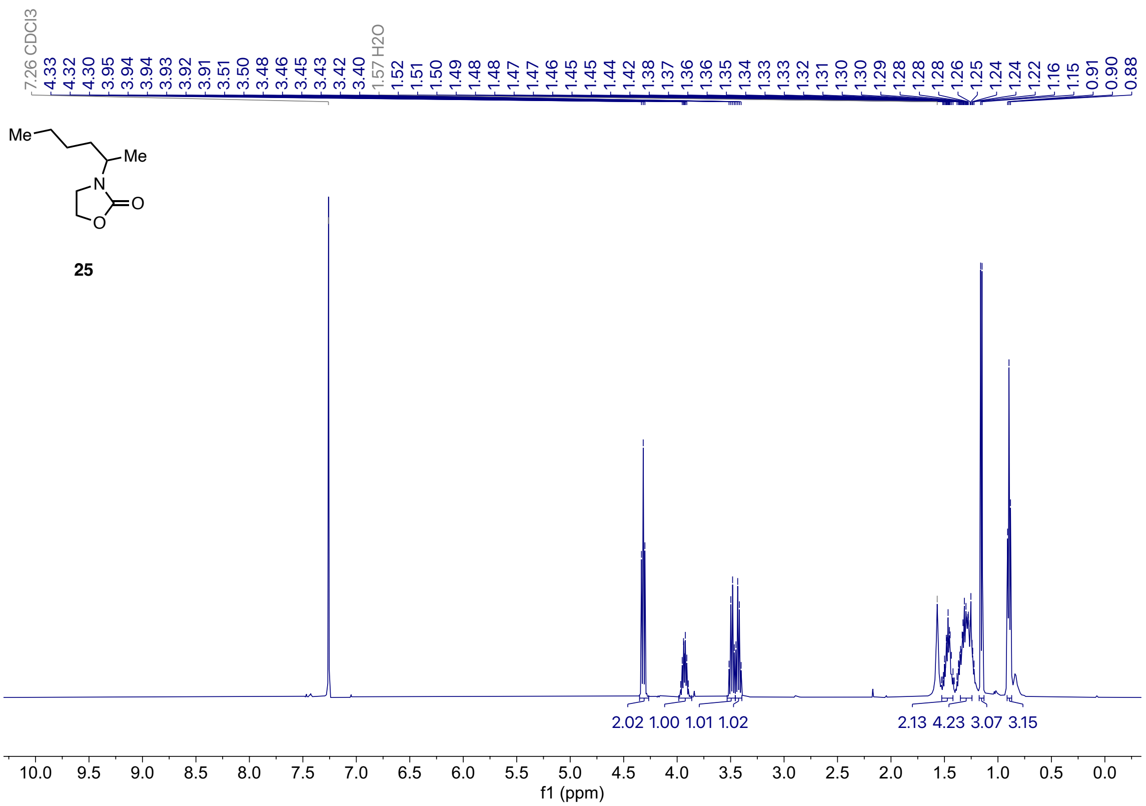

^{1}H NMR spectrum of **25** taken in $CDCl_3$.

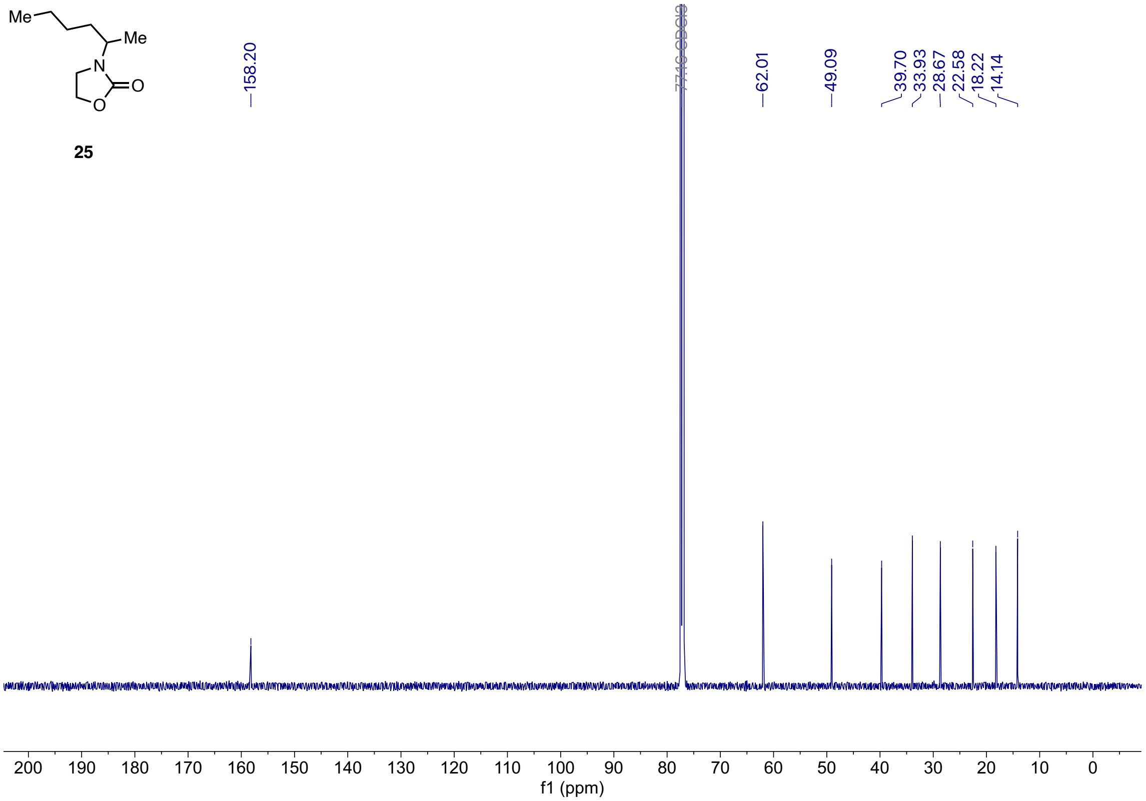

^{13}C NMR spectrum of **25** taken in $CDCl_3$.

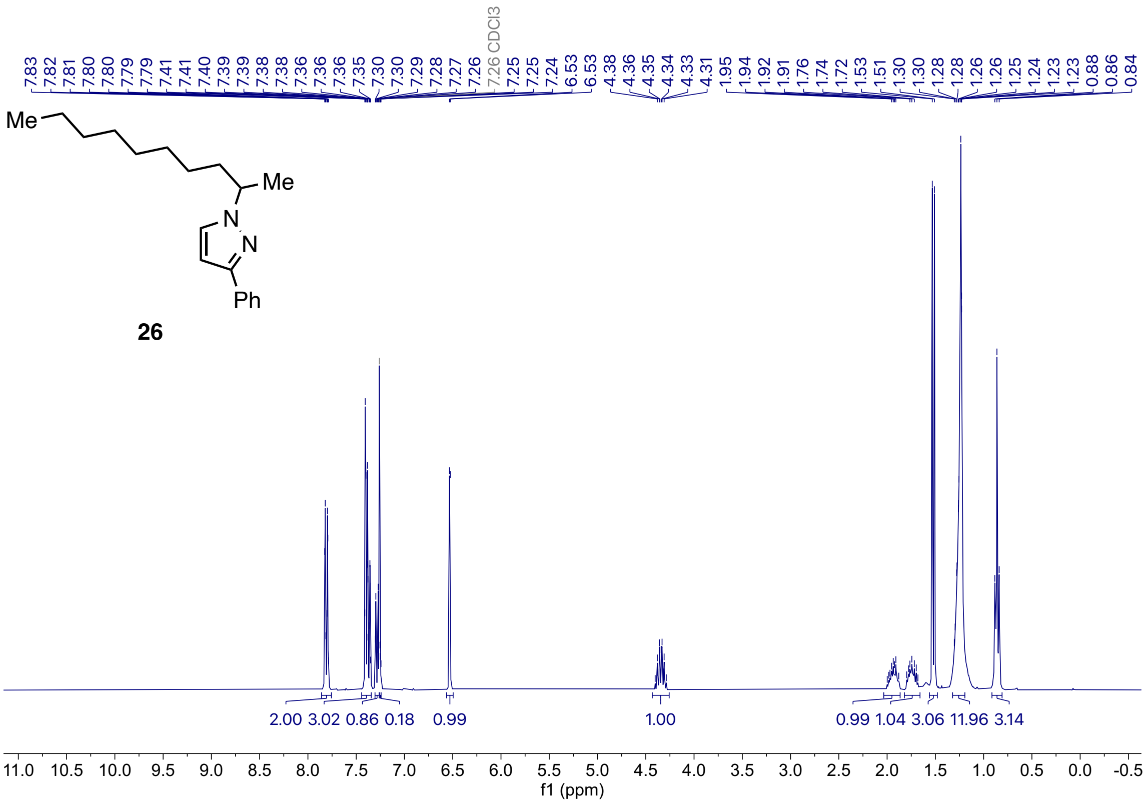

^{1}H NMR spectrum of **26** taken in $CDCl_3$.

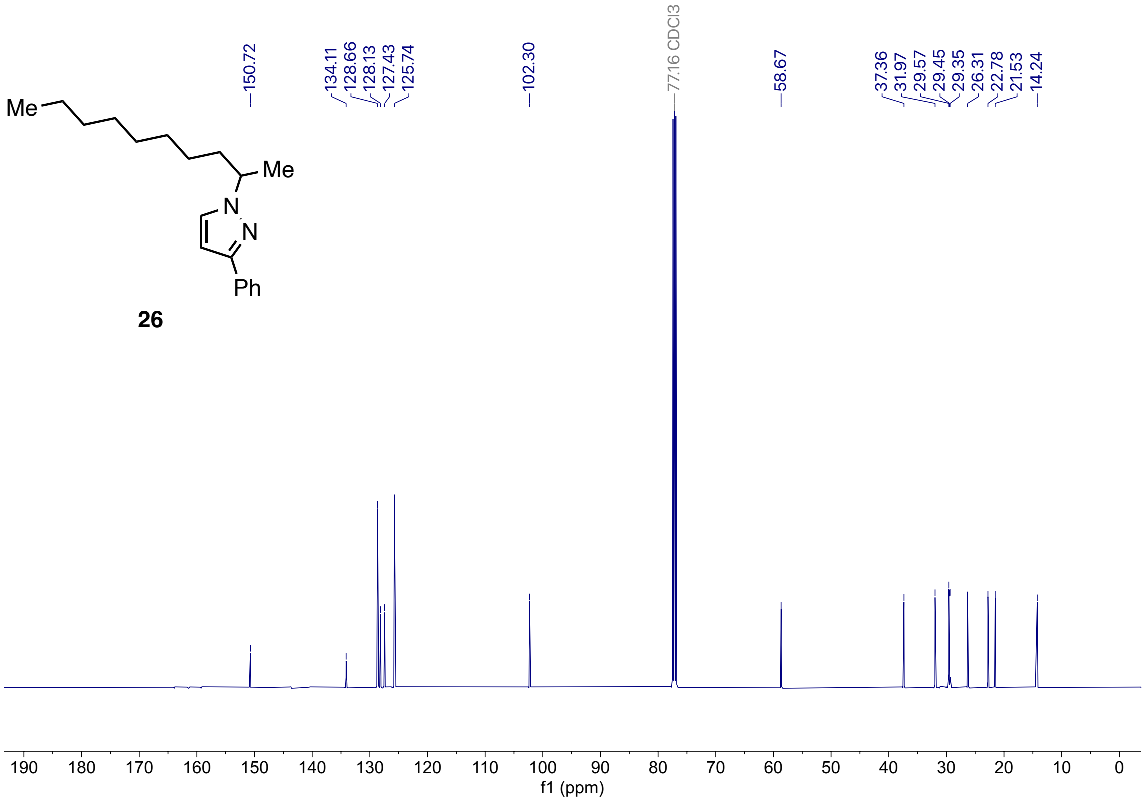

^{13}C NMR spectrum of **26** taken in $CDCl_3$.

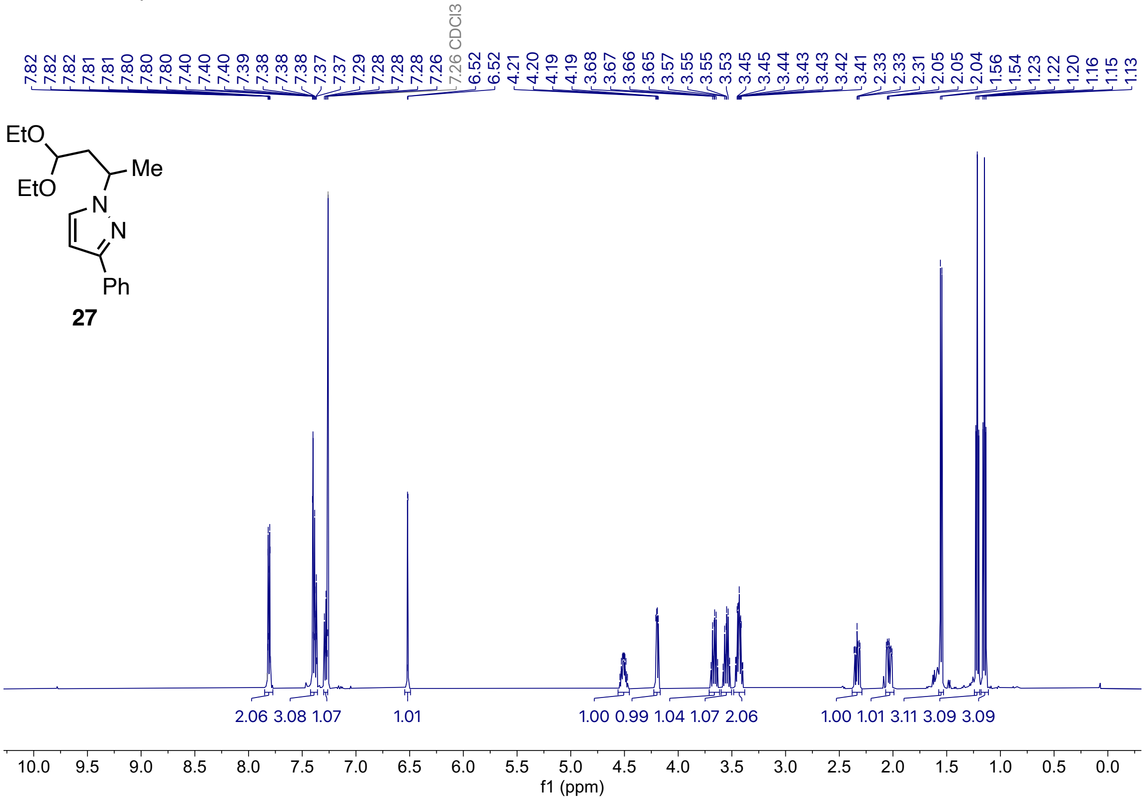

^{1}H NMR spectrum of **27** taken in $CDCl_3$.

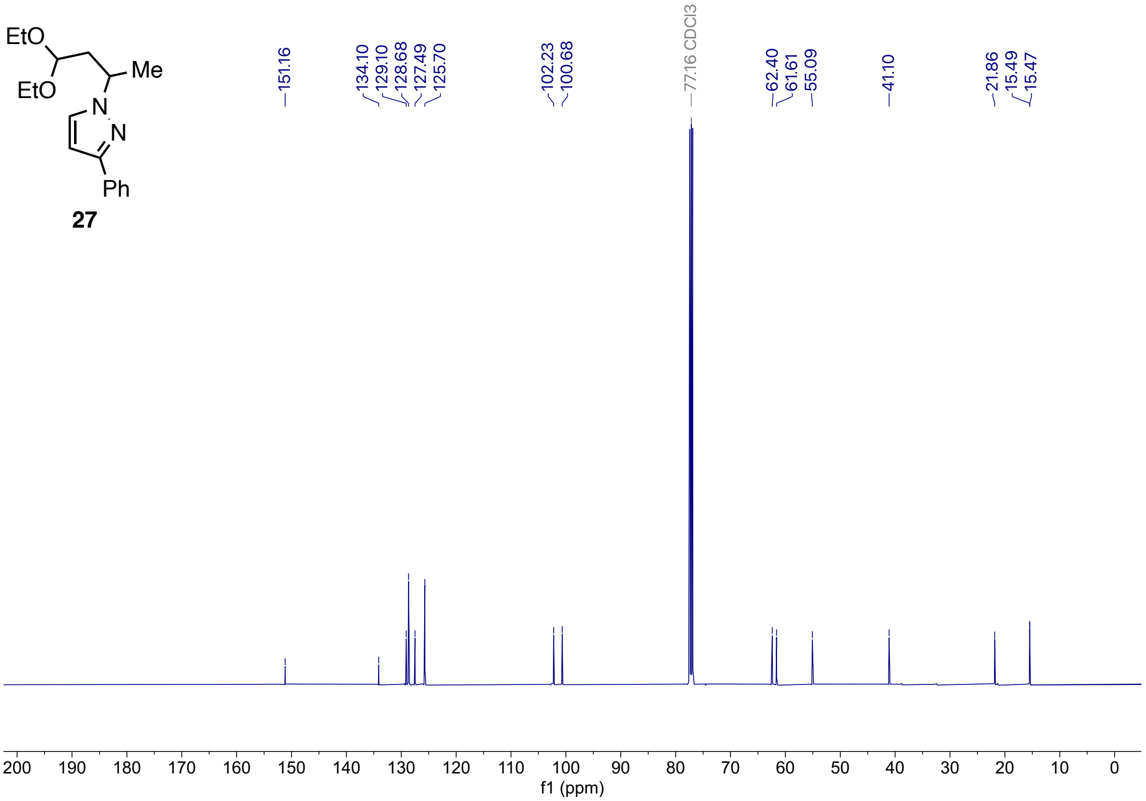

^{13}C NMR spectrum of **27** taken in $CDCl_3$.

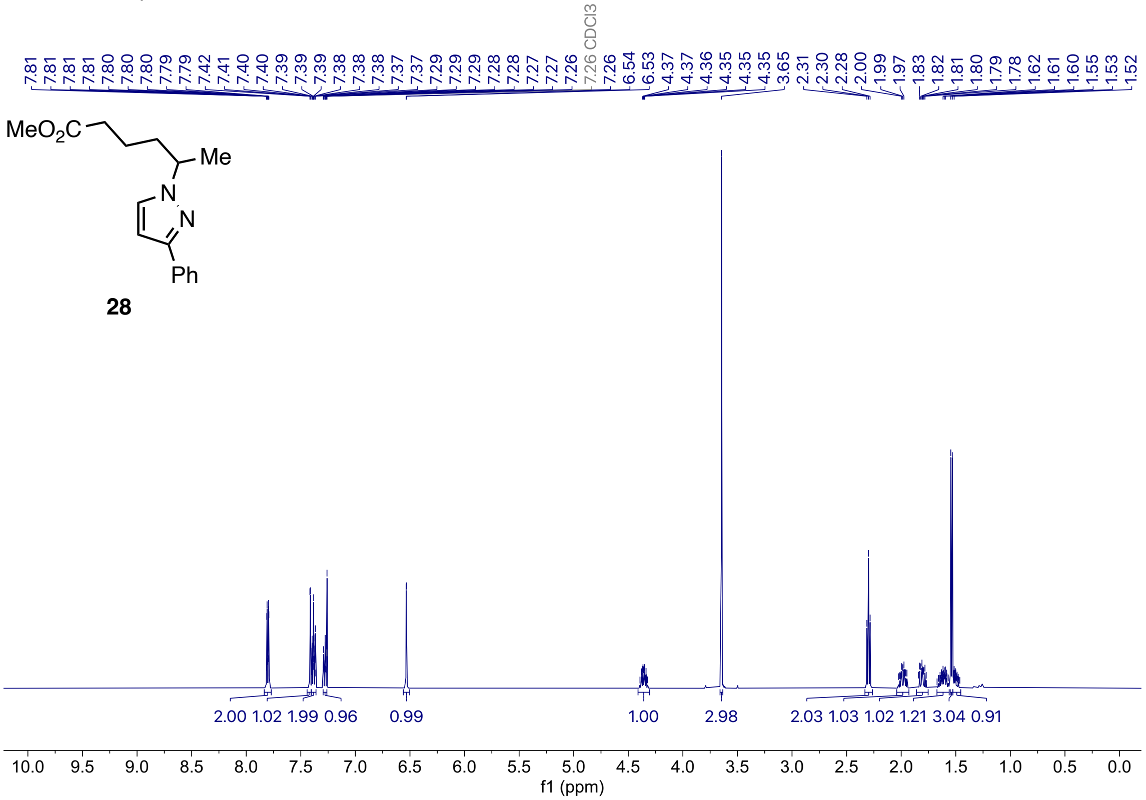

^{1}H NMR spectrum of **28** taken in $CDCl_3$.

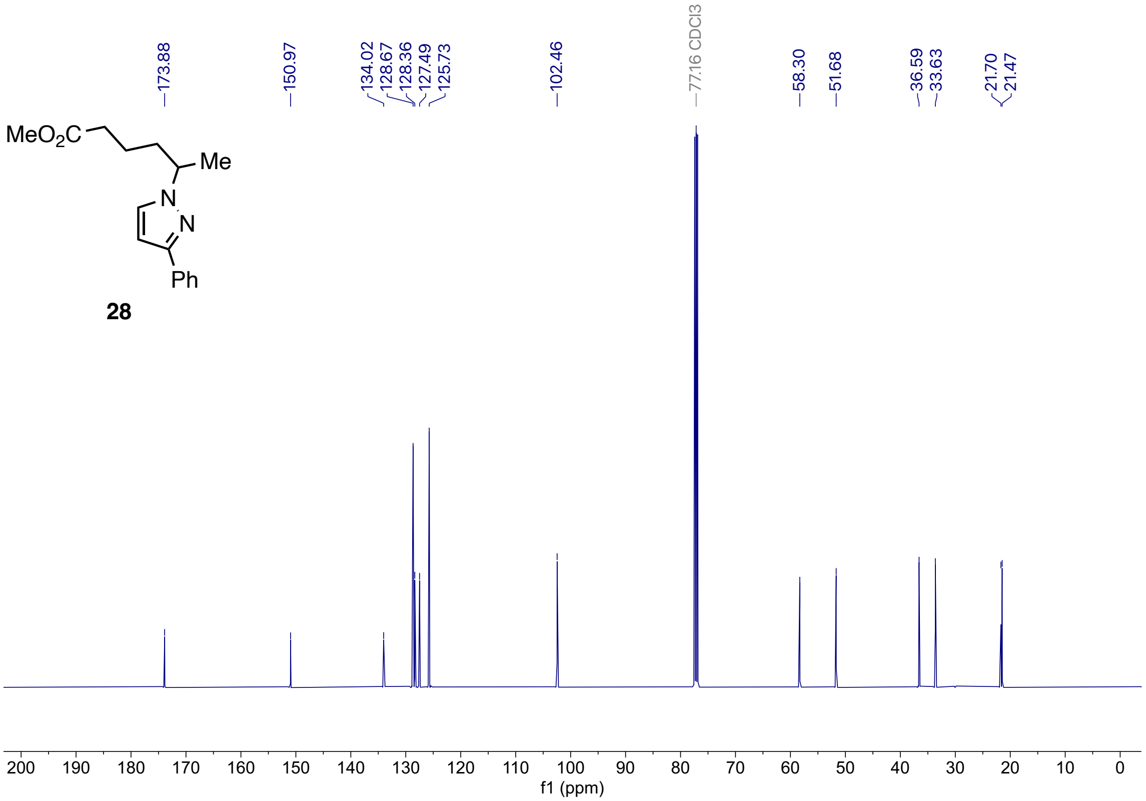

28

^{13}C NMR spectrum of **28** taken in $CDCl_3$.

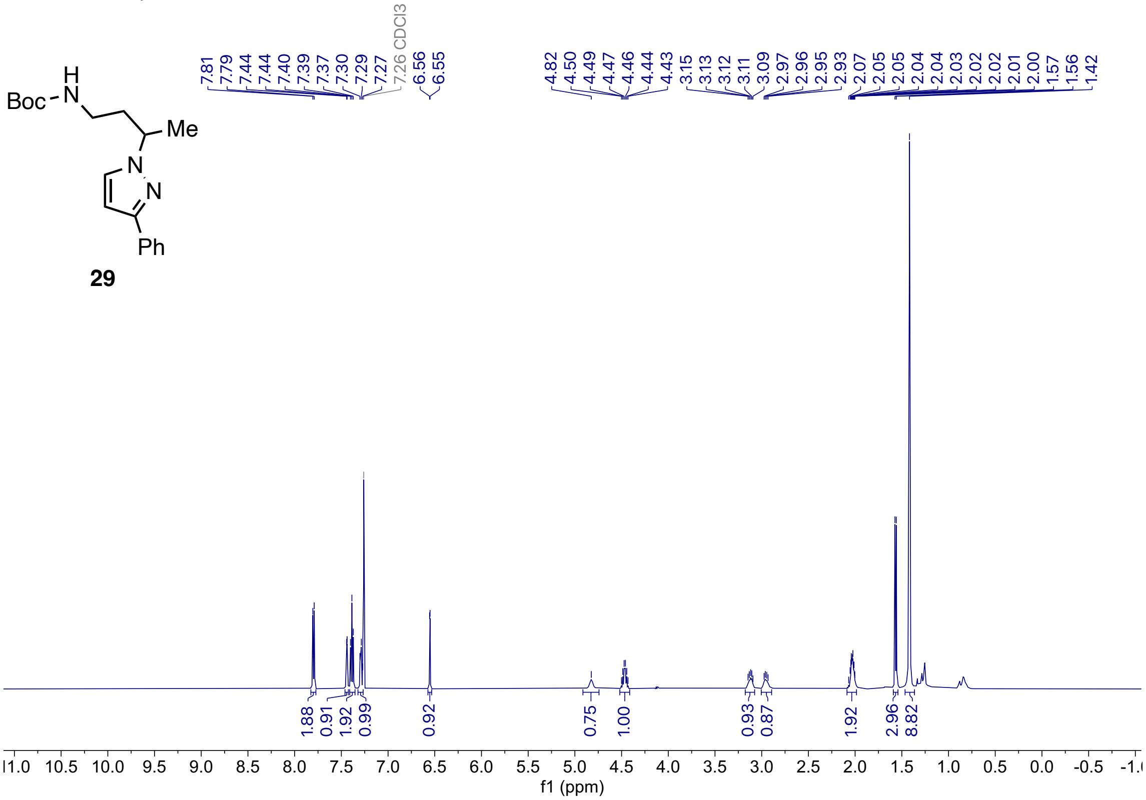

29

^{1}H NMR spectrum of **29** taken in $CDCl_3$.

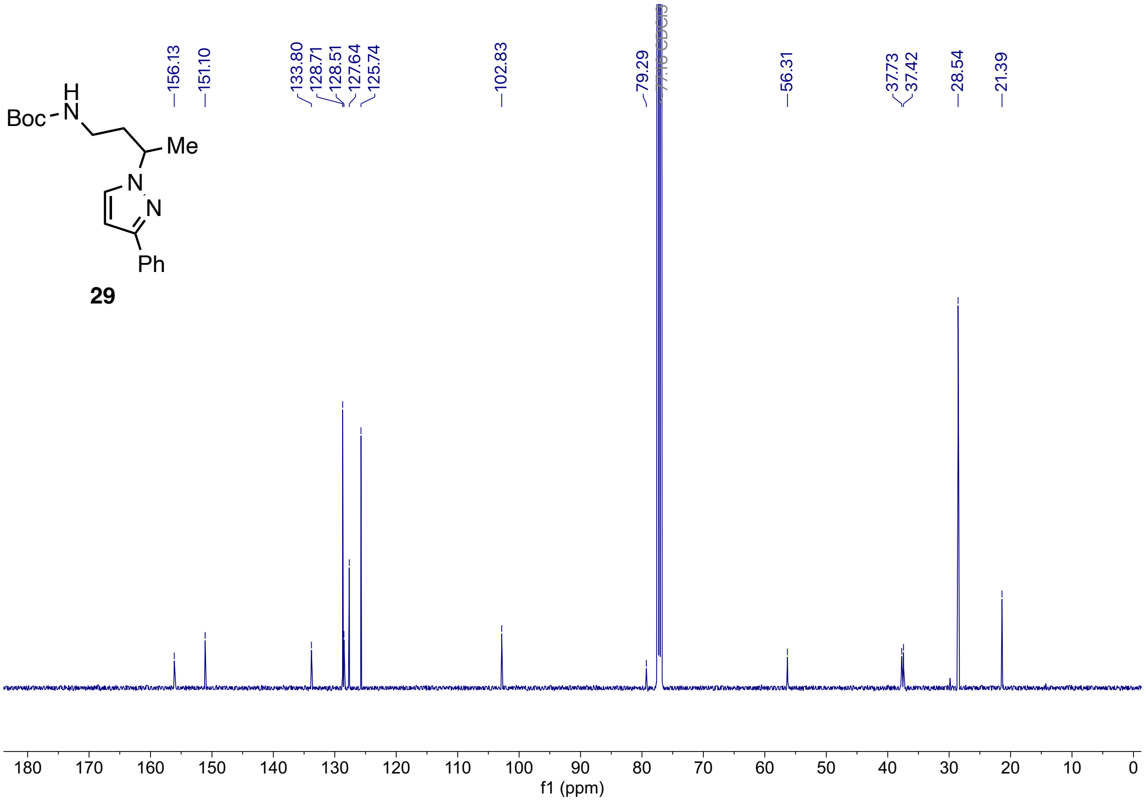

^{13}C NMR spectrum of **29** taken in $CDCl_3$.

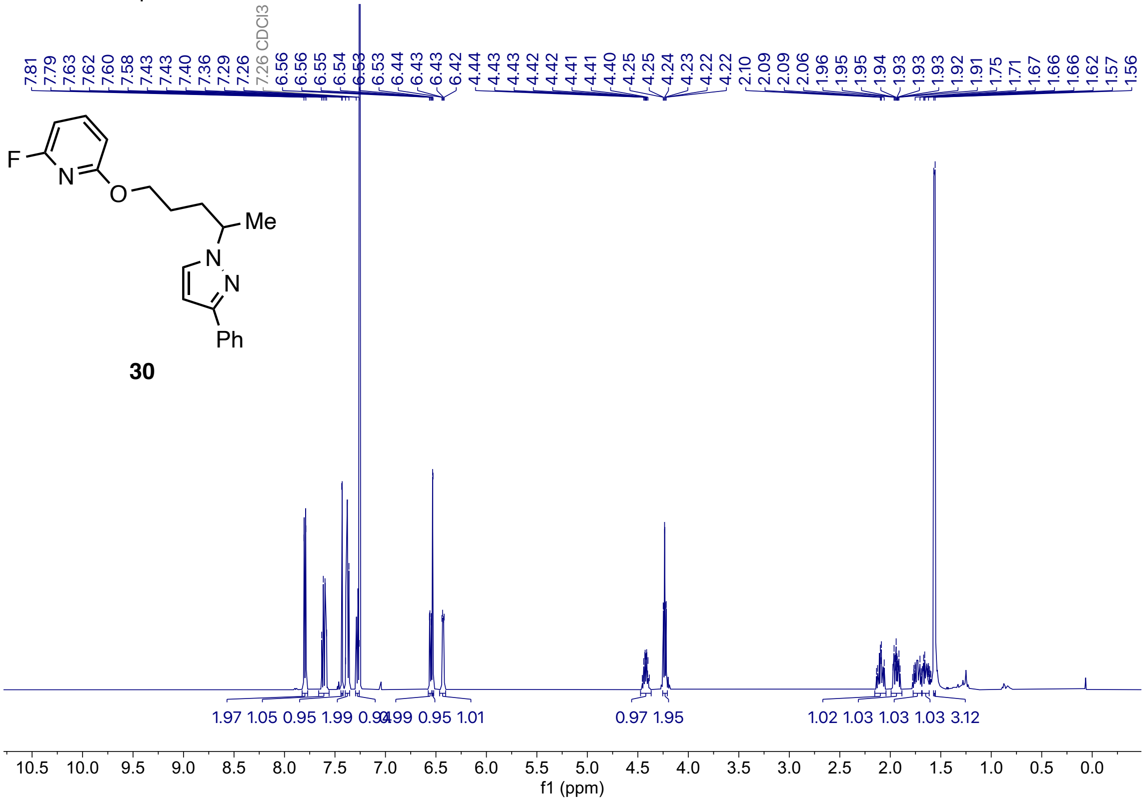

^{1}H NMR spectrum of **30** taken in $CDCl_3$.

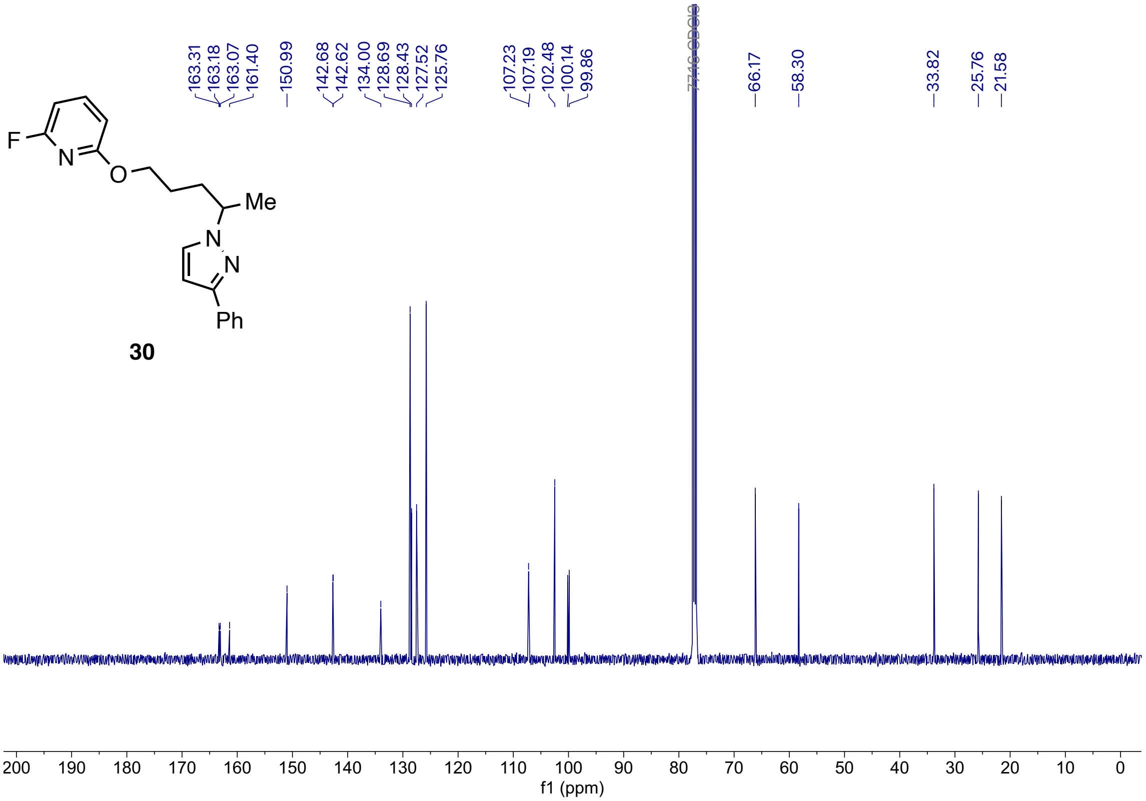

^{13}C NMR spectrum of **30** taken in $CDCl_3$.

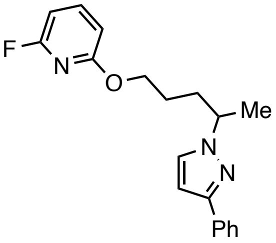

30

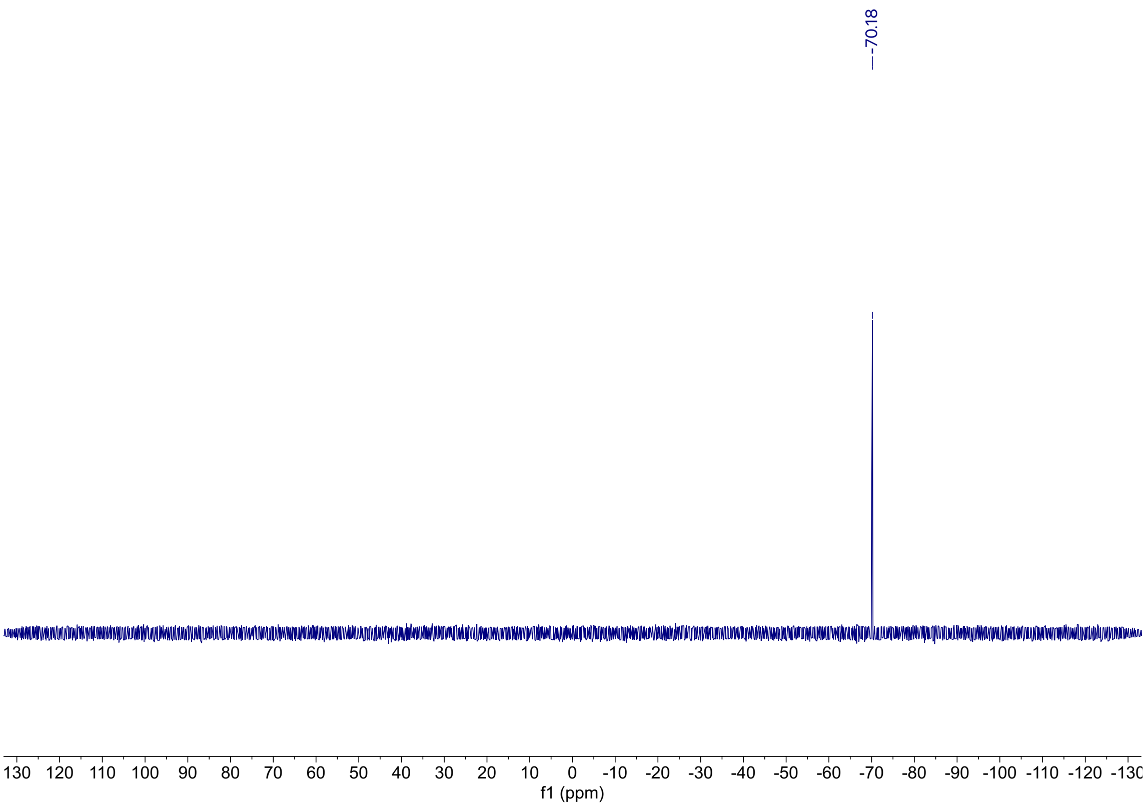

$^{19}F\{^{1}H\}$ NMR spectrum of **30** taken in $CDCl_3$.

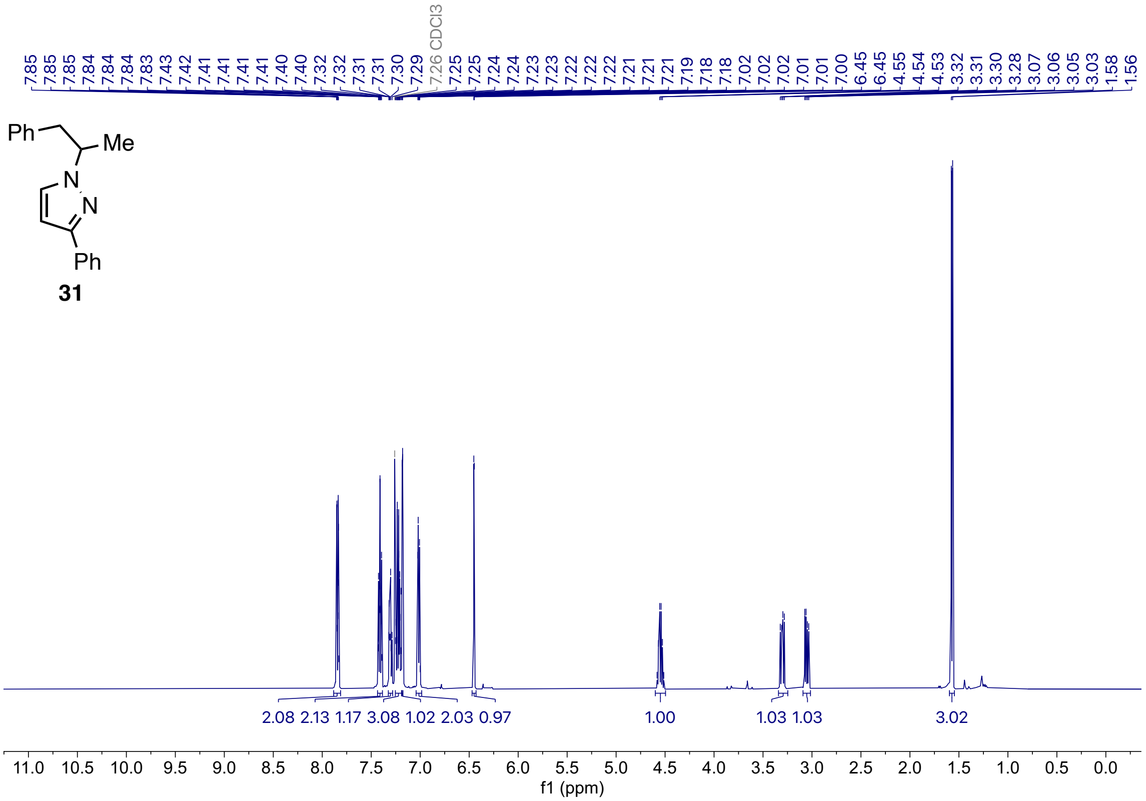

^{1}H NMR spectrum of **31** taken in $CDCl_3$.

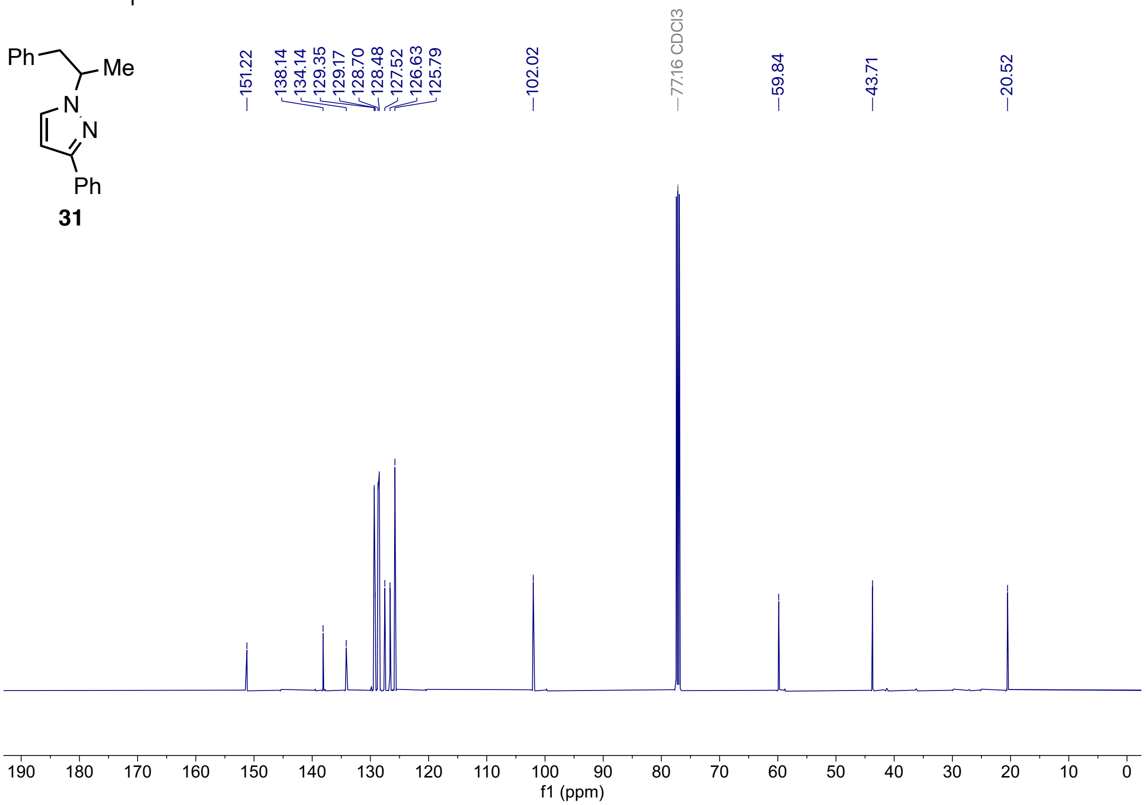

^{13}C NMR spectrum of **31** taken in $CDCl_3$.

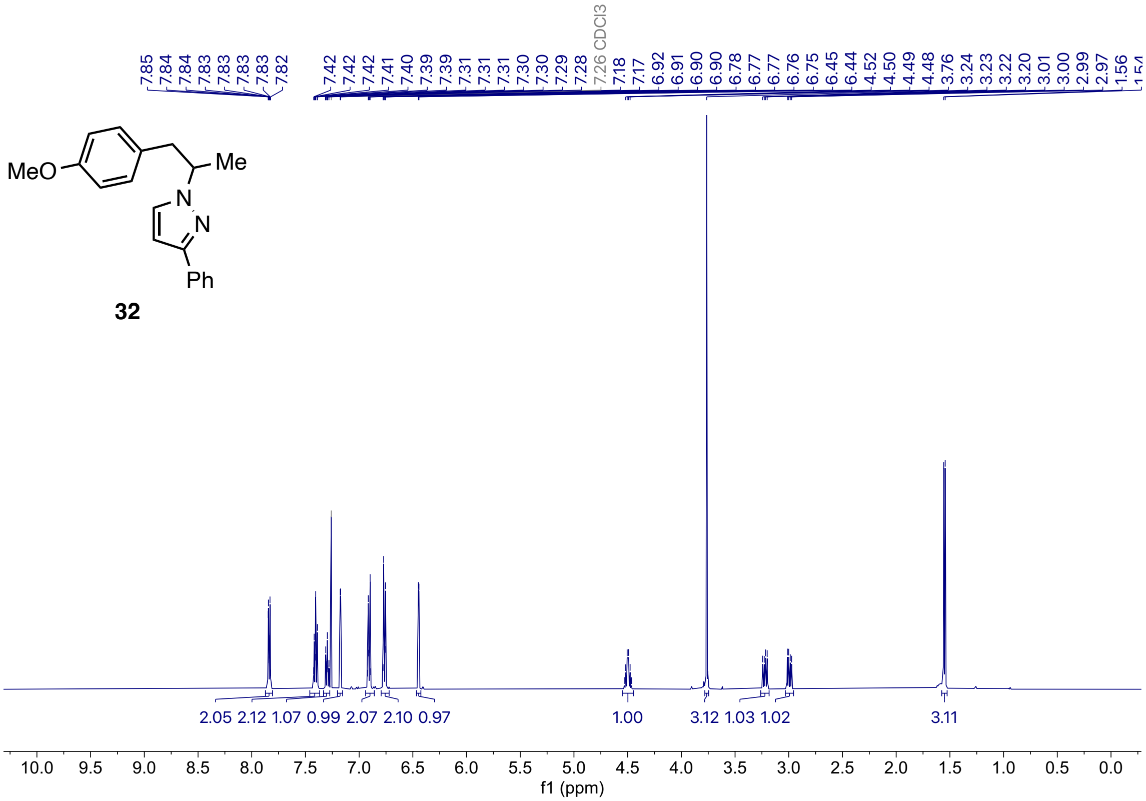

^{1}H NMR spectrum of **32** taken in $CDCl_3$.

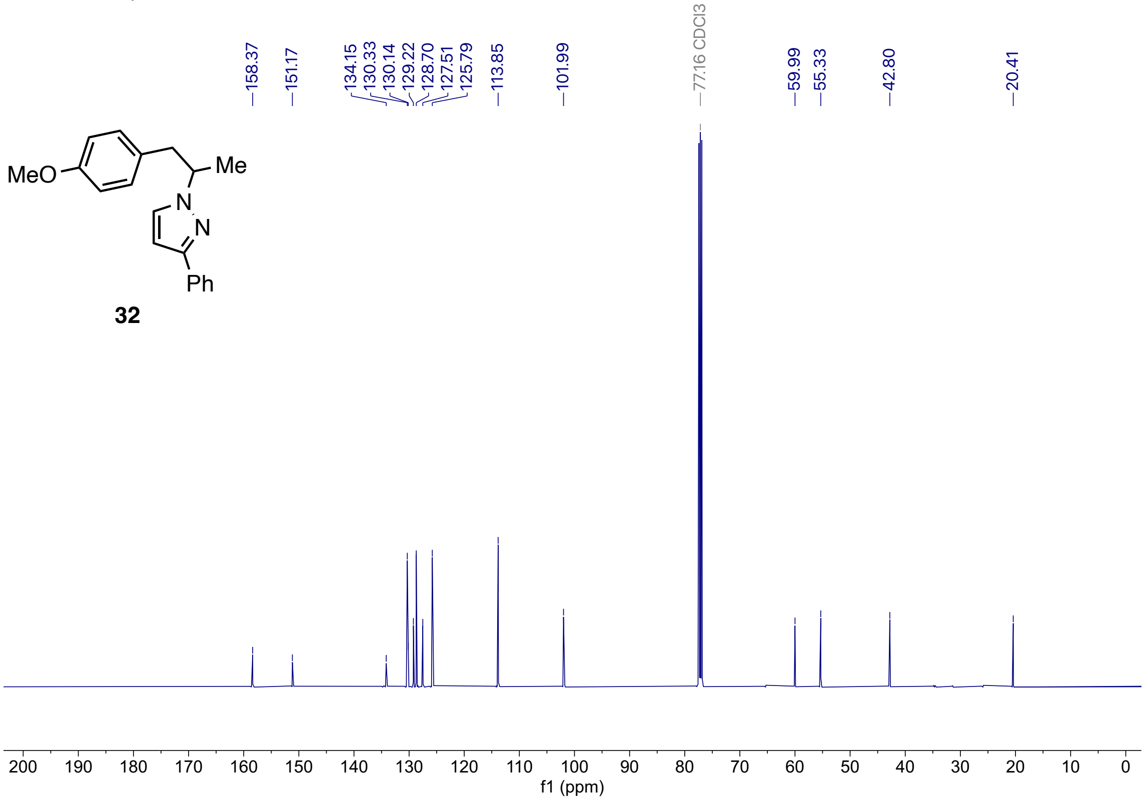

^{13}C NMR spectrum of **32** taken in $CDCl_3$.

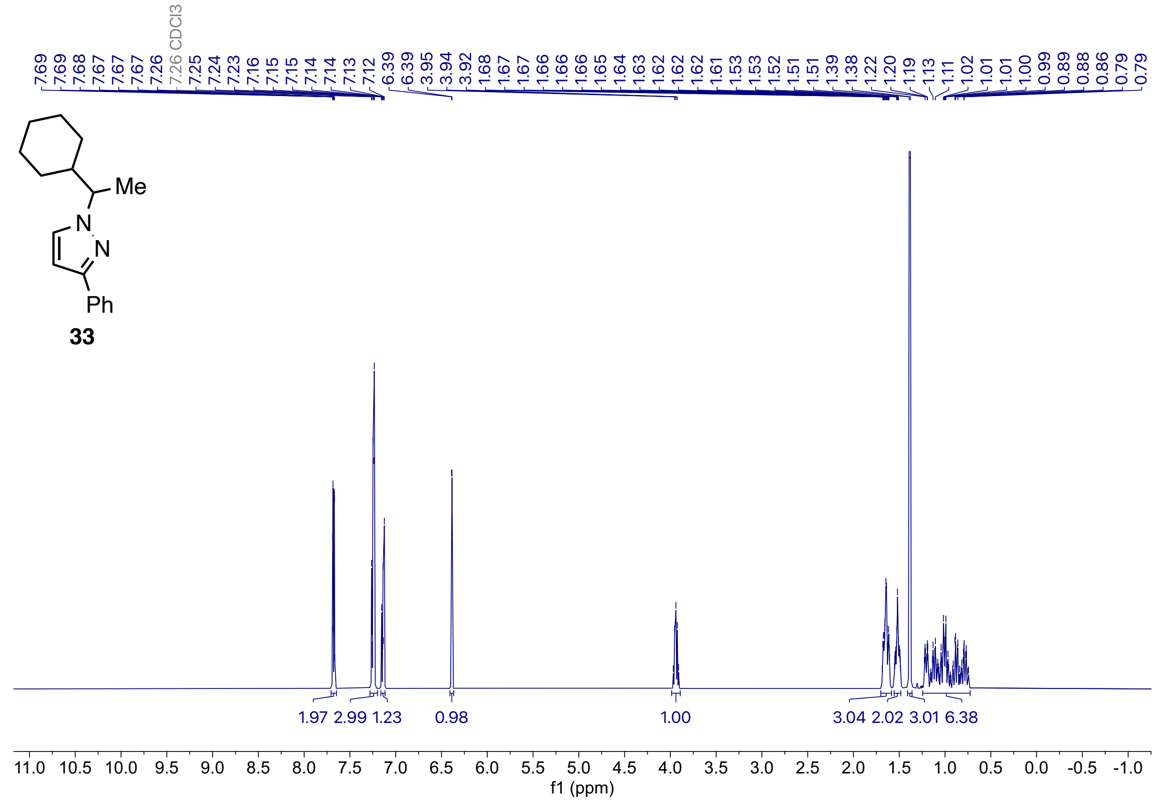

^{1}H NMR spectrum of **33** taken in $CDCl_3$.

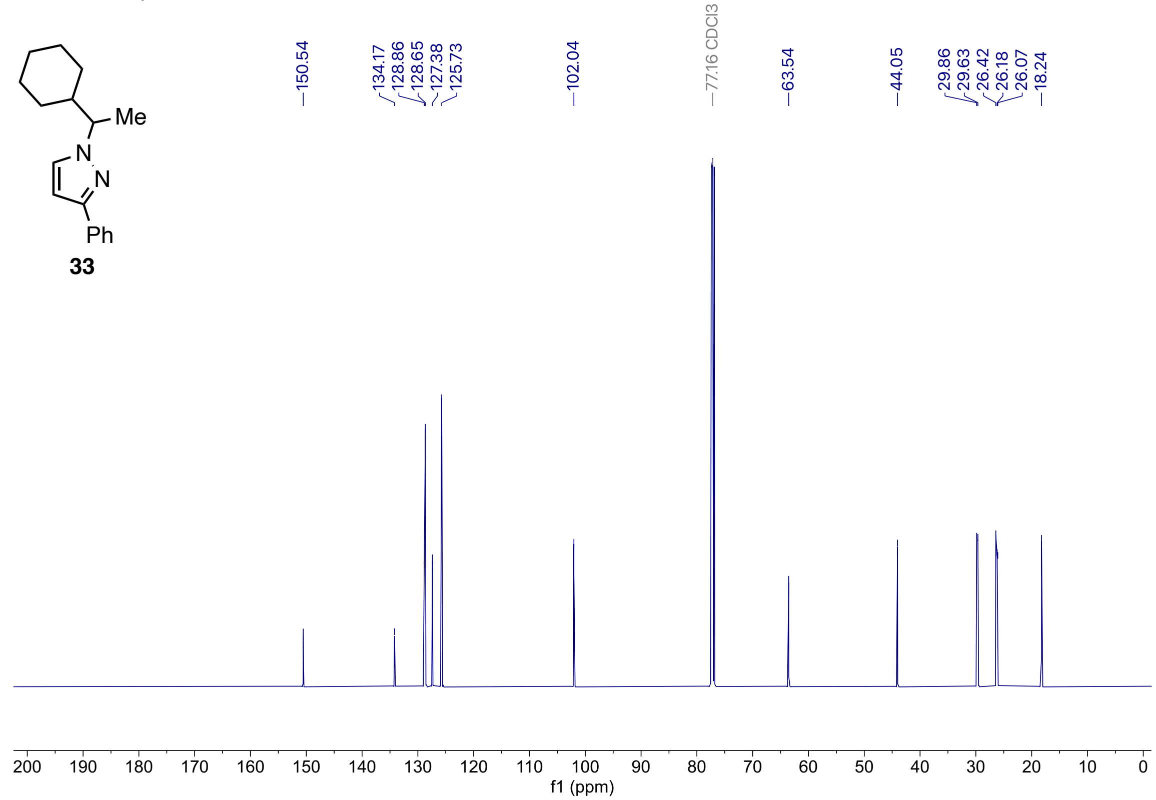

^{13}C NMR spectrum of **33** taken in $CDCl_3$.

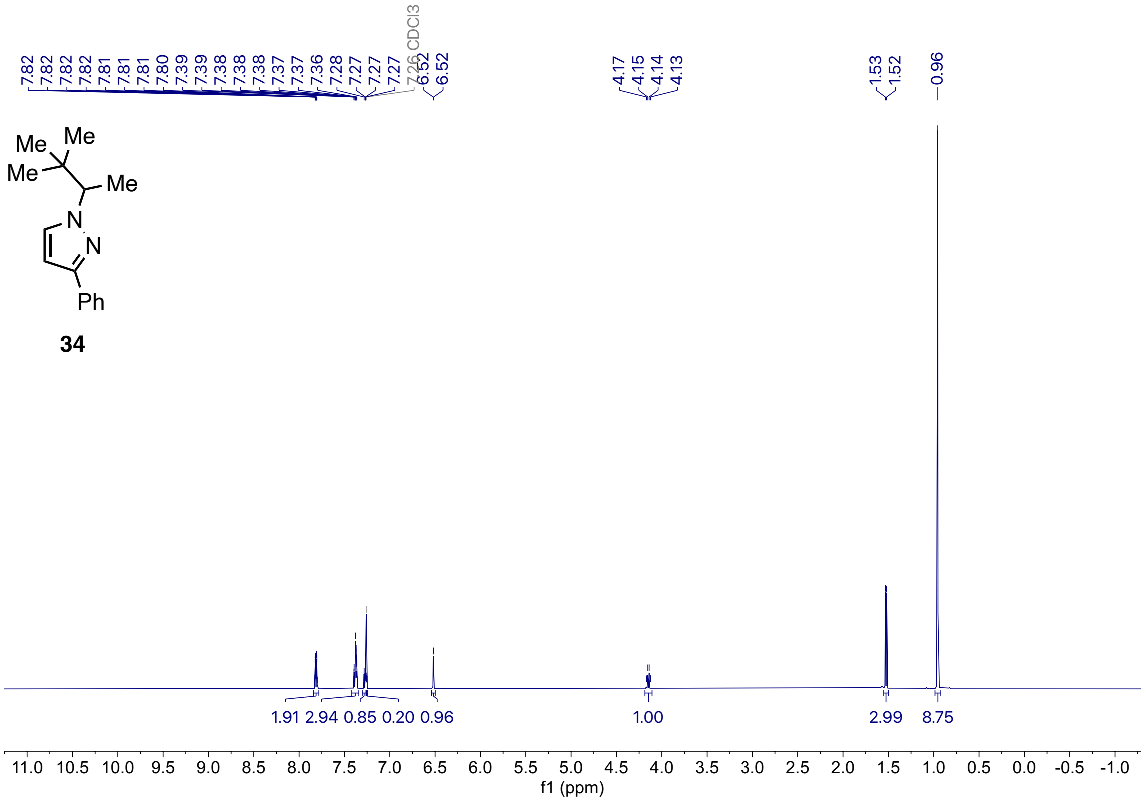

^{1}H NMR spectrum of **34** taken in $CDCl_3$.

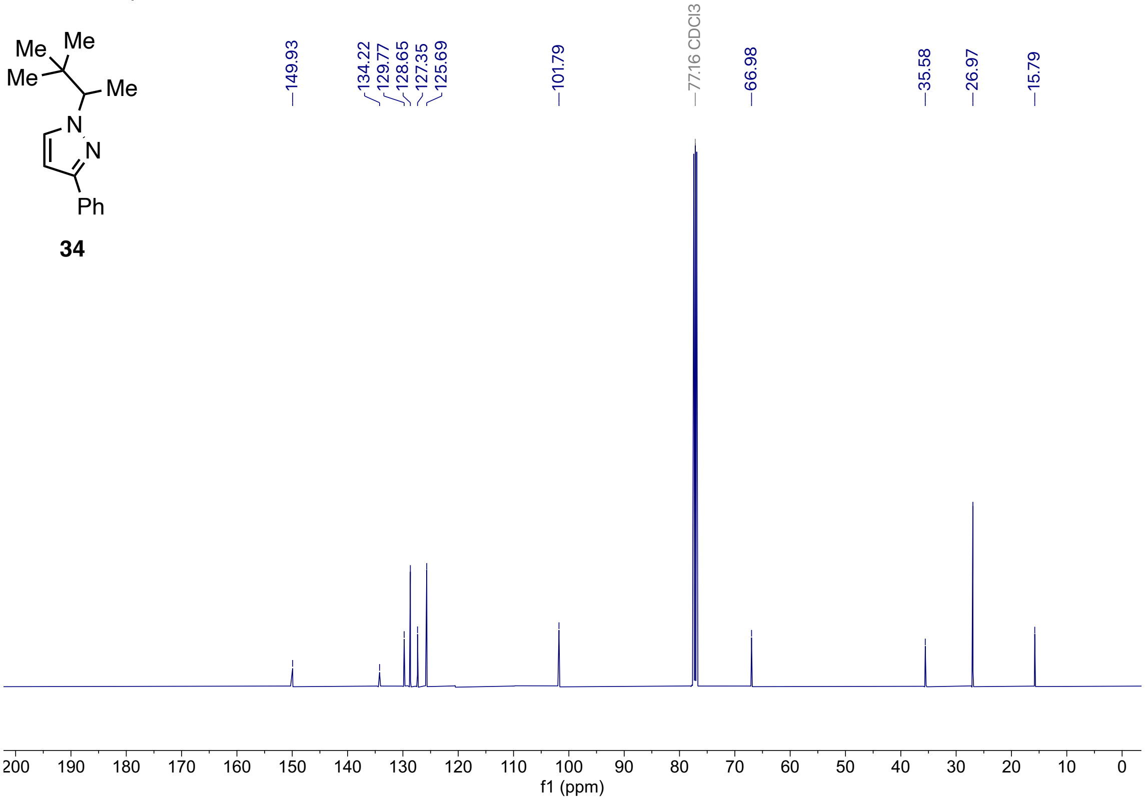

^{13}C NMR spectrum of **34** taken in $CDCl_3$.

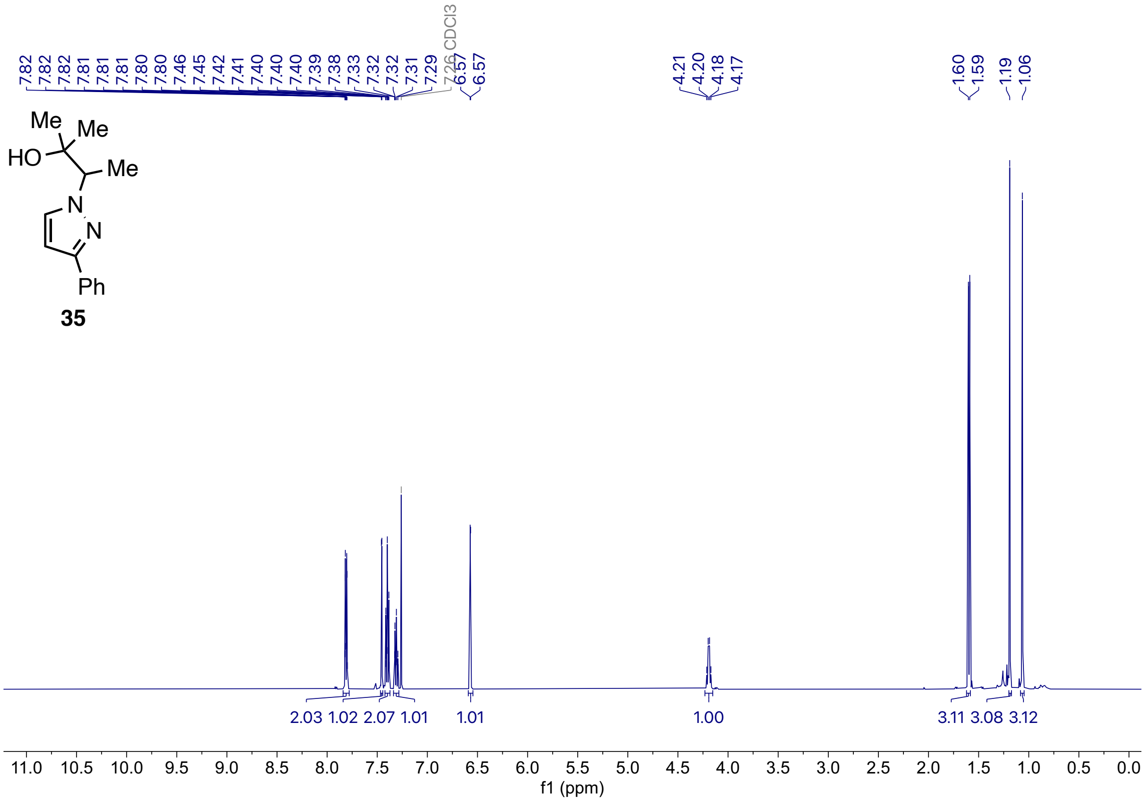

^1H NMR spectrum of **35** taken in $CDCl_3$.

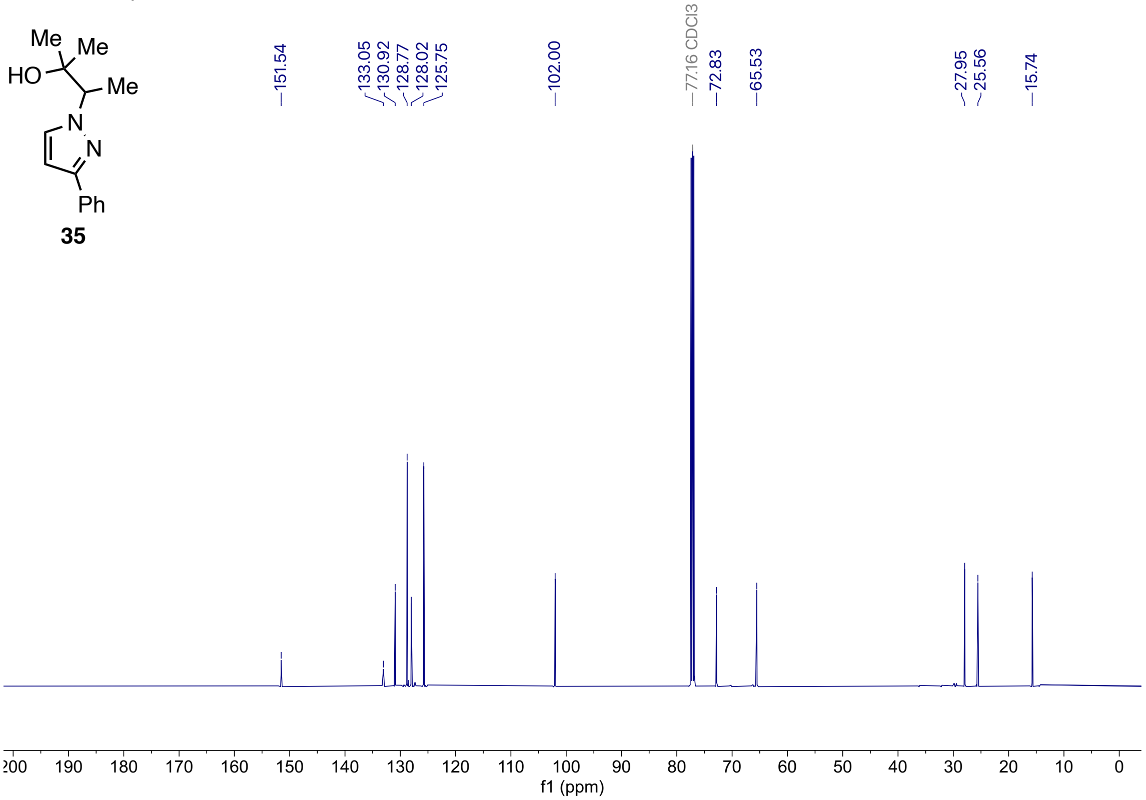

^{13}C NMR spectrum of **35** taken in $CDCl_3$.

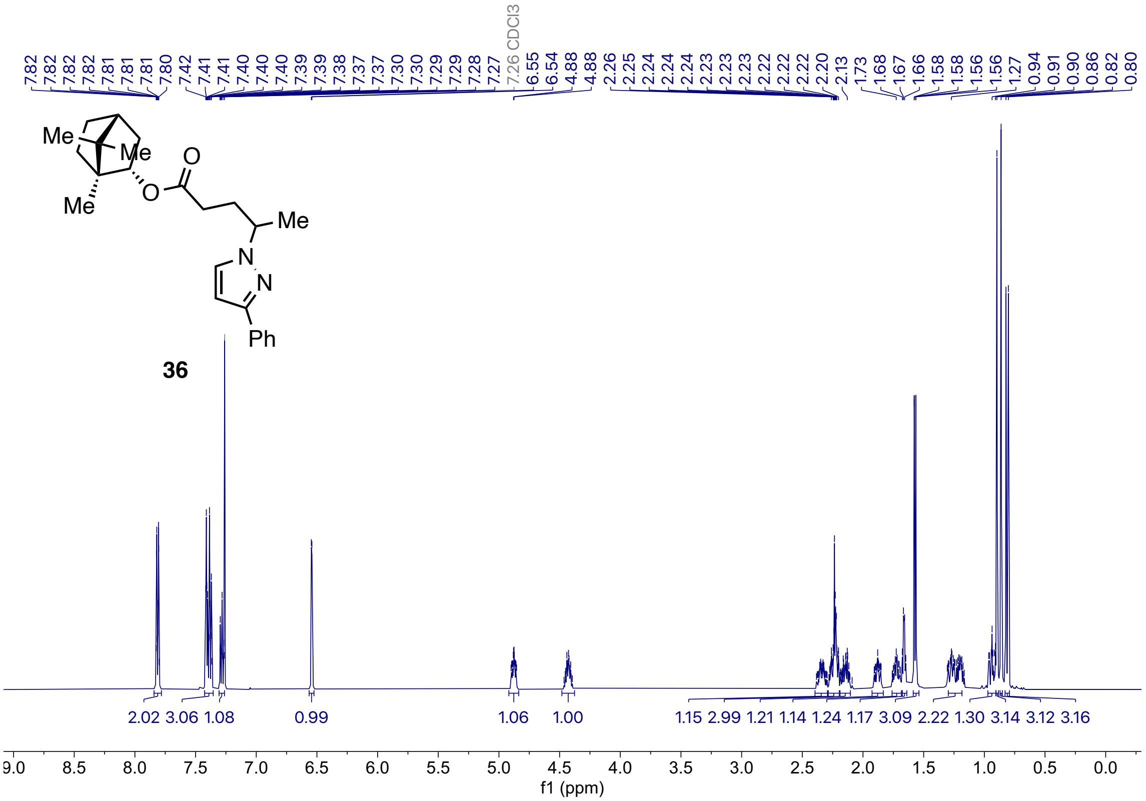

^{1}H NMR spectrum of **36** taken in $CDCl_3$.

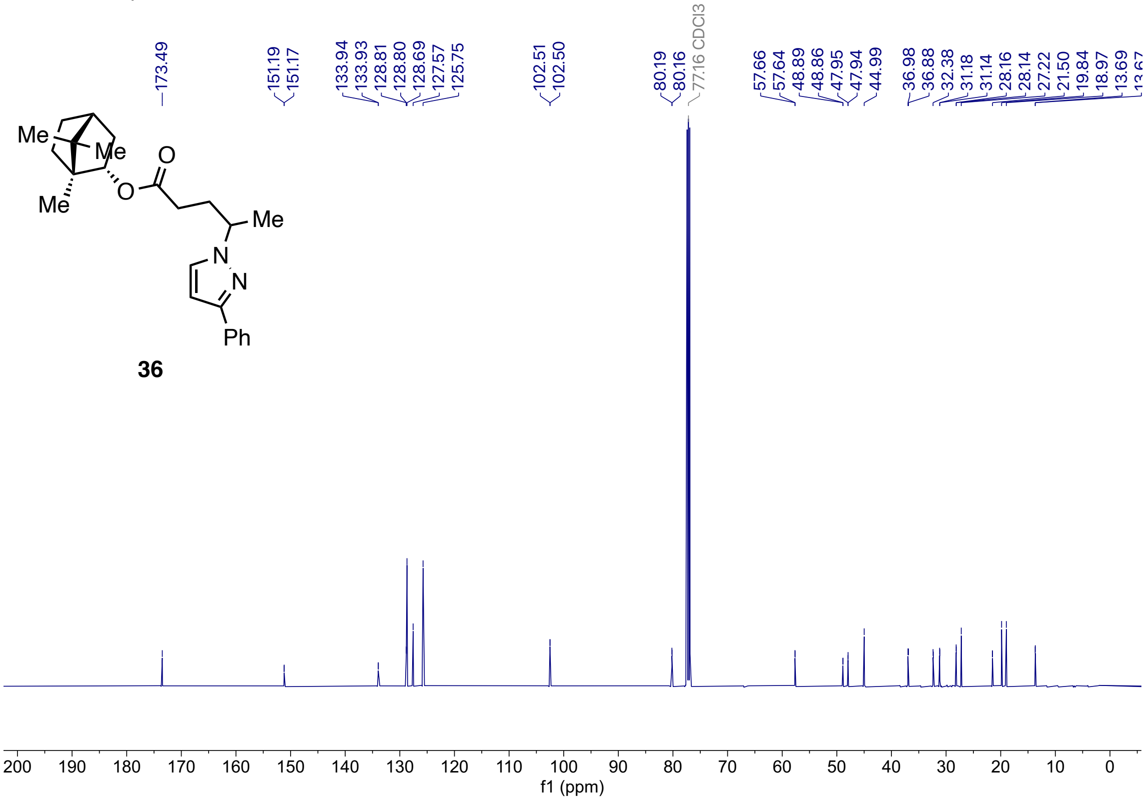

^{13}C NMR spectrum of **36** taken in $CDCl_3$.

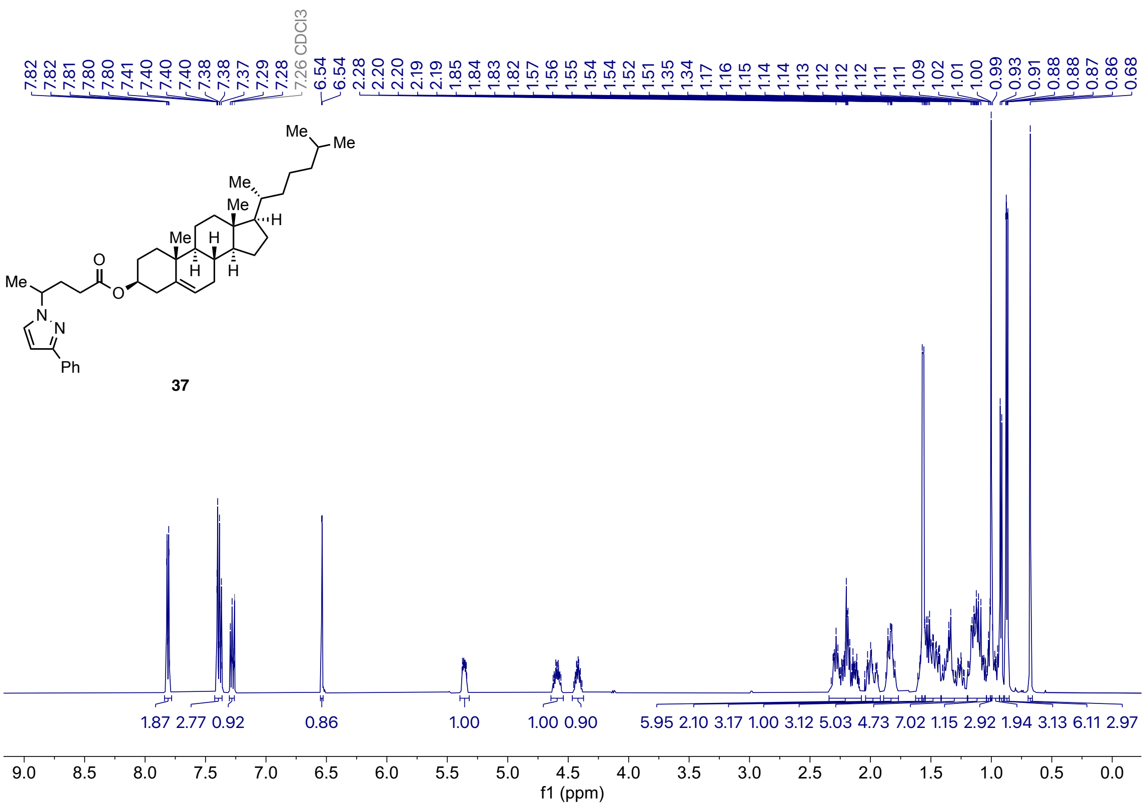

^{1}H NMR spectrum of **37** taken in $CDCl_3$.

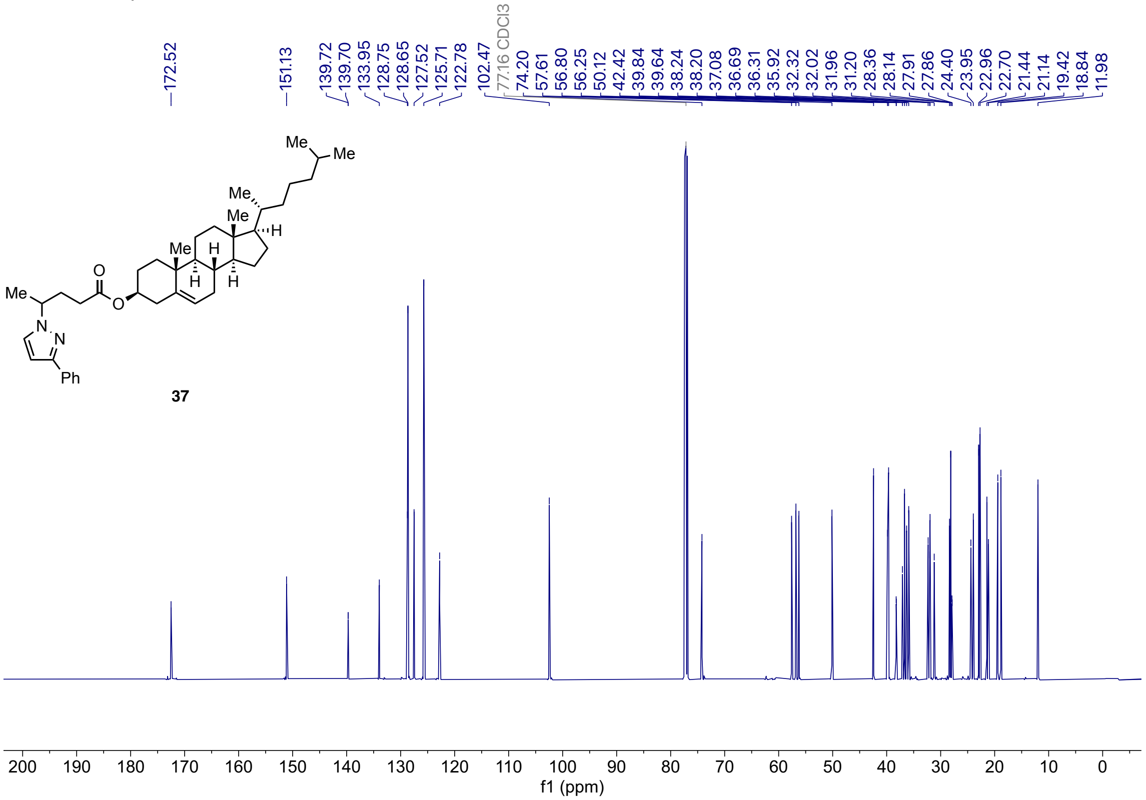

^{13}C NMR spectrum of **37** taken in $CDCl_3$.

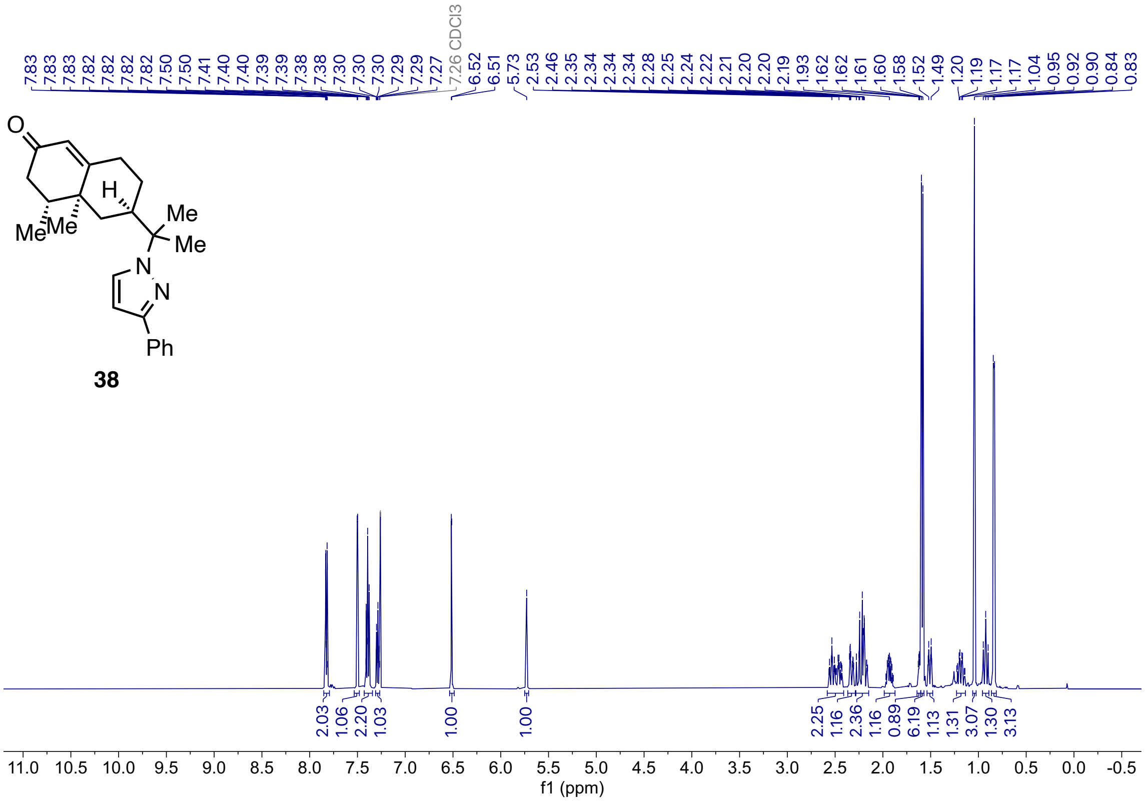

^{1}H NMR spectrum of **38** taken in $CDCl_3$.

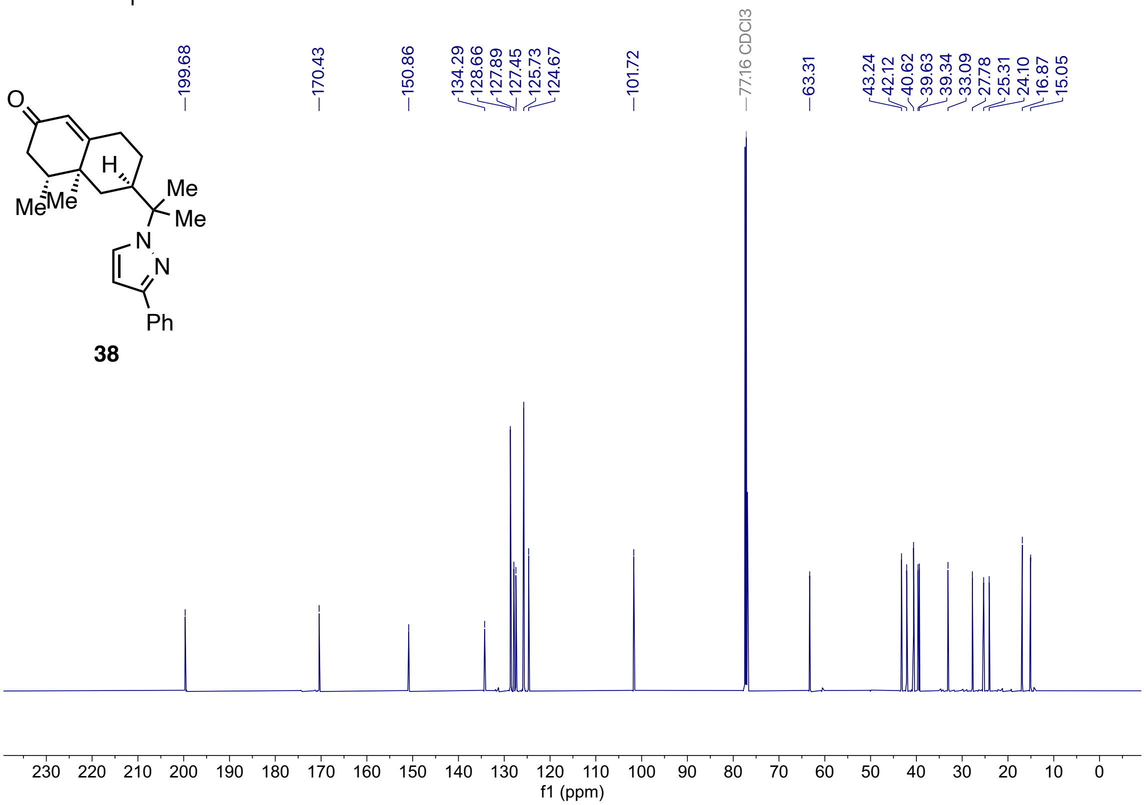

^{13}C NMR spectrum of **38** taken in $CDCl_3$.

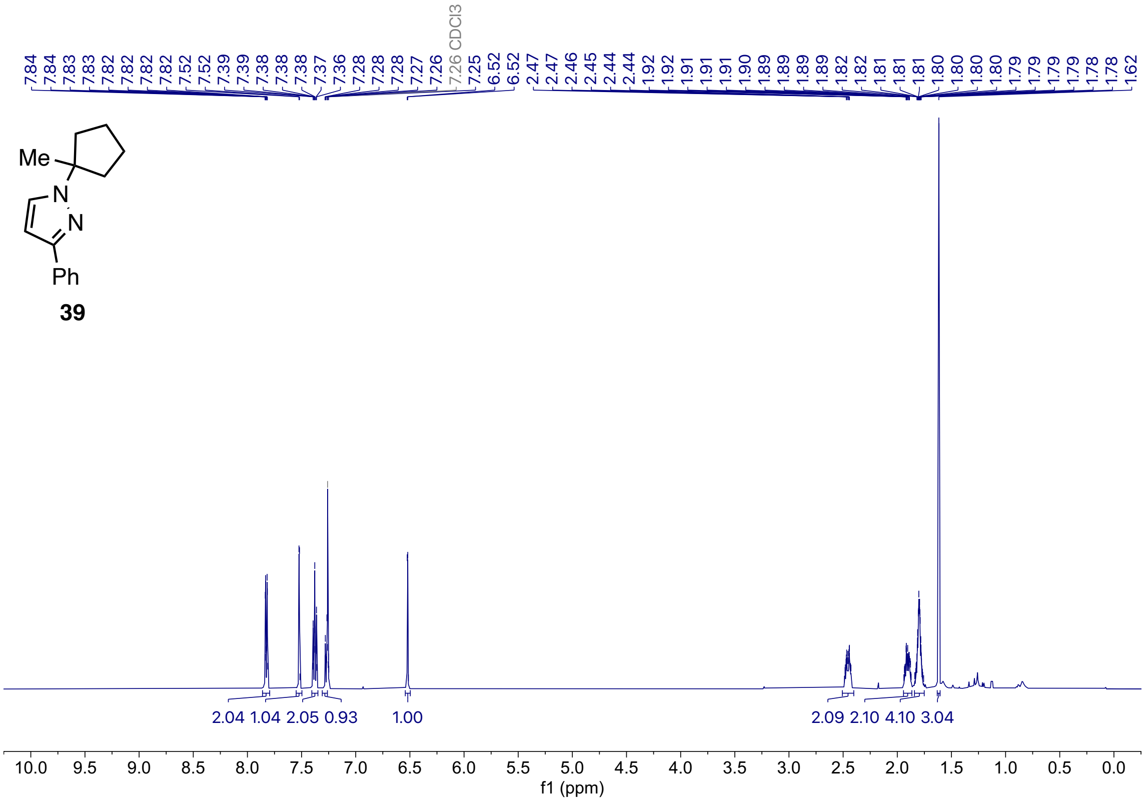

^{1}H NMR spectrum of **39** taken in $CDCl_3$.

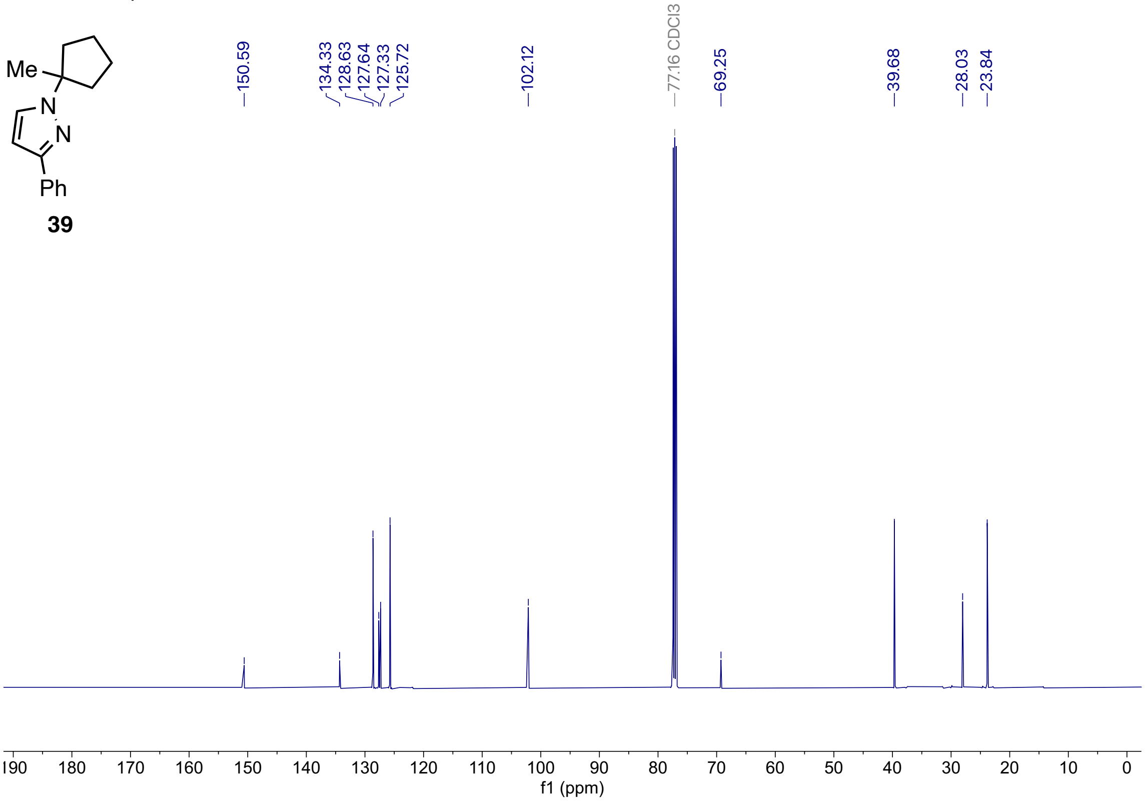

^{13}C NMR spectrum of **39** taken in $CDCl_3$.

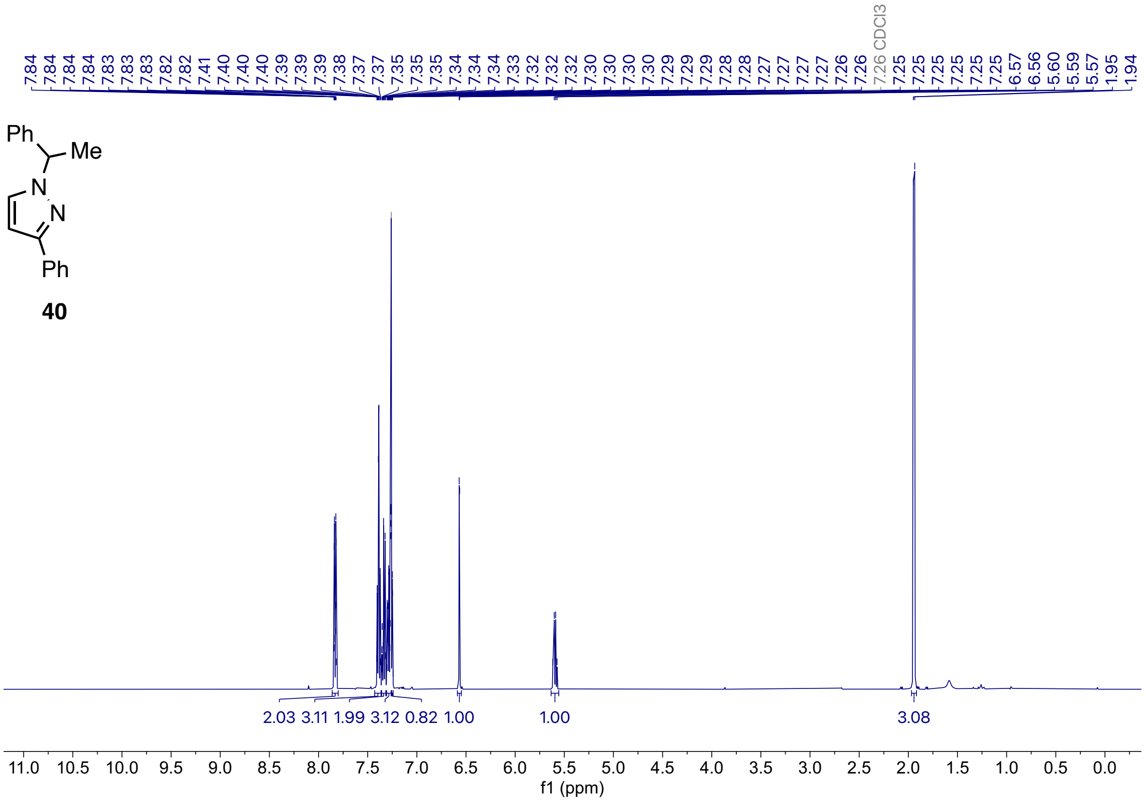

^{1}H NMR spectrum of **40** taken in $CDCl_3$.

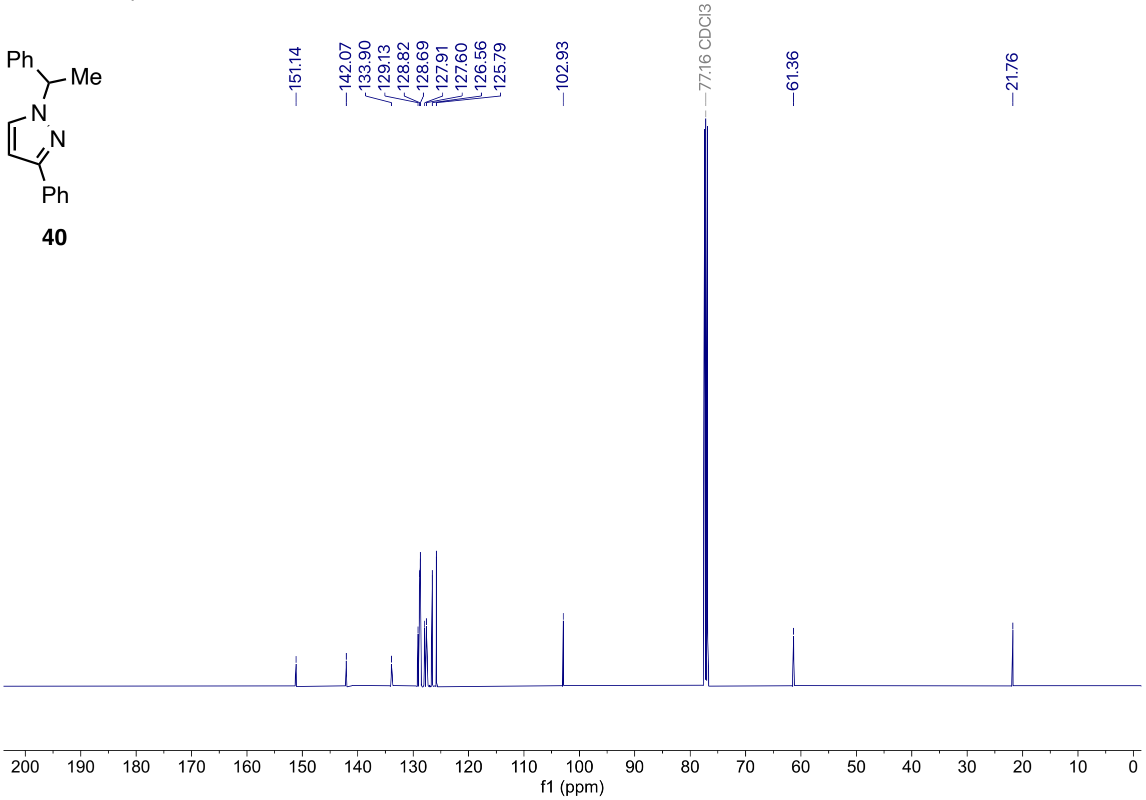

^{13}C NMR spectrum of **40** taken in $CDCl_3$.

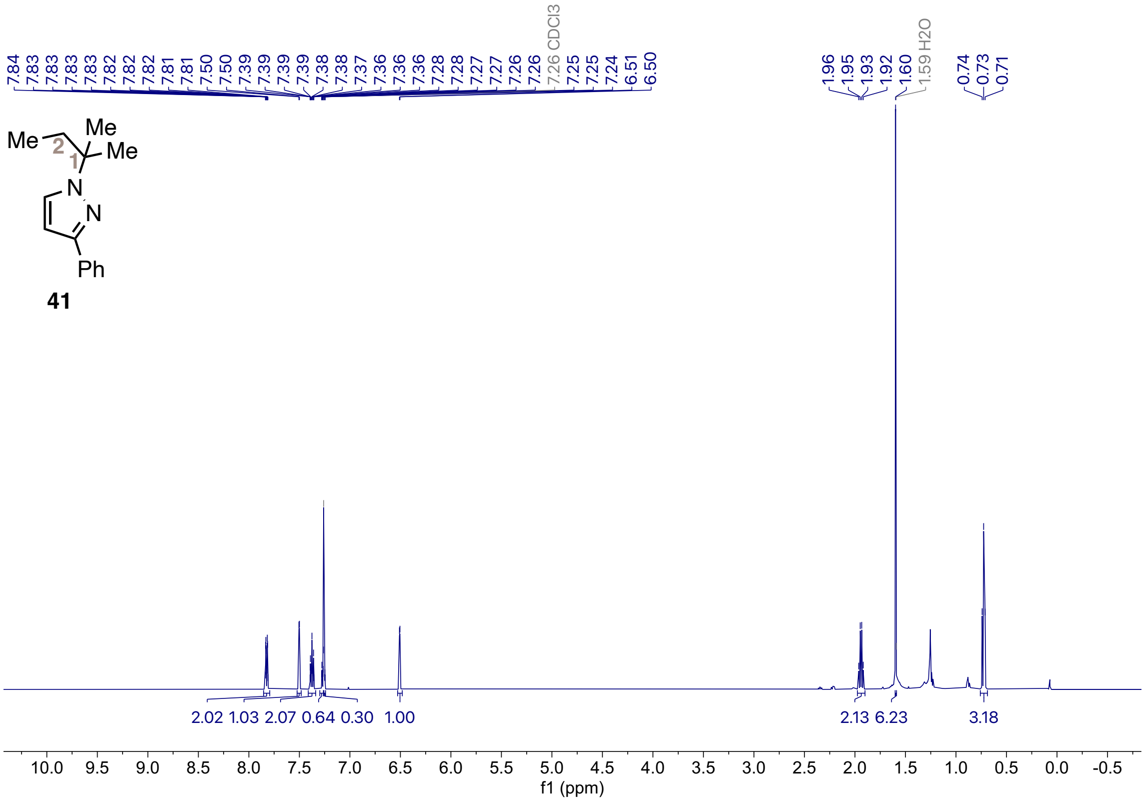

^{1}H NMR spectrum of **41** taken in $CDCl_3$.

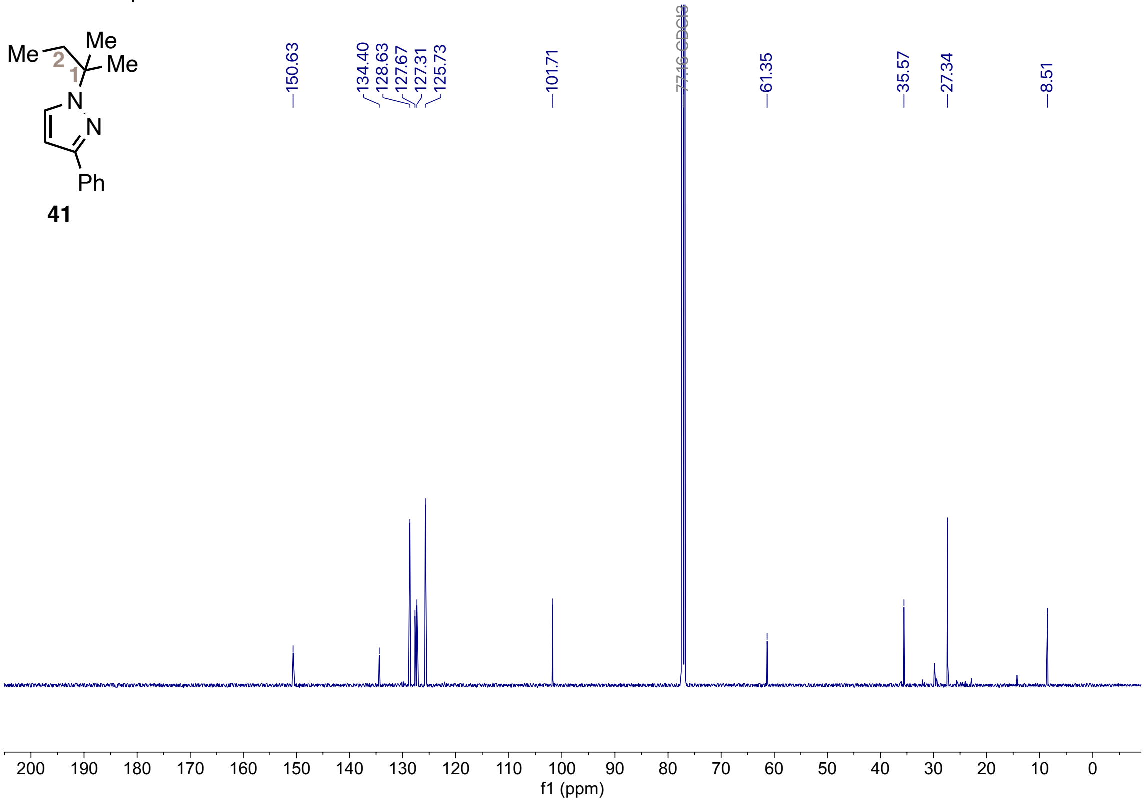

^{13}C NMR spectrum of **41** taken in $CDCl_3$.

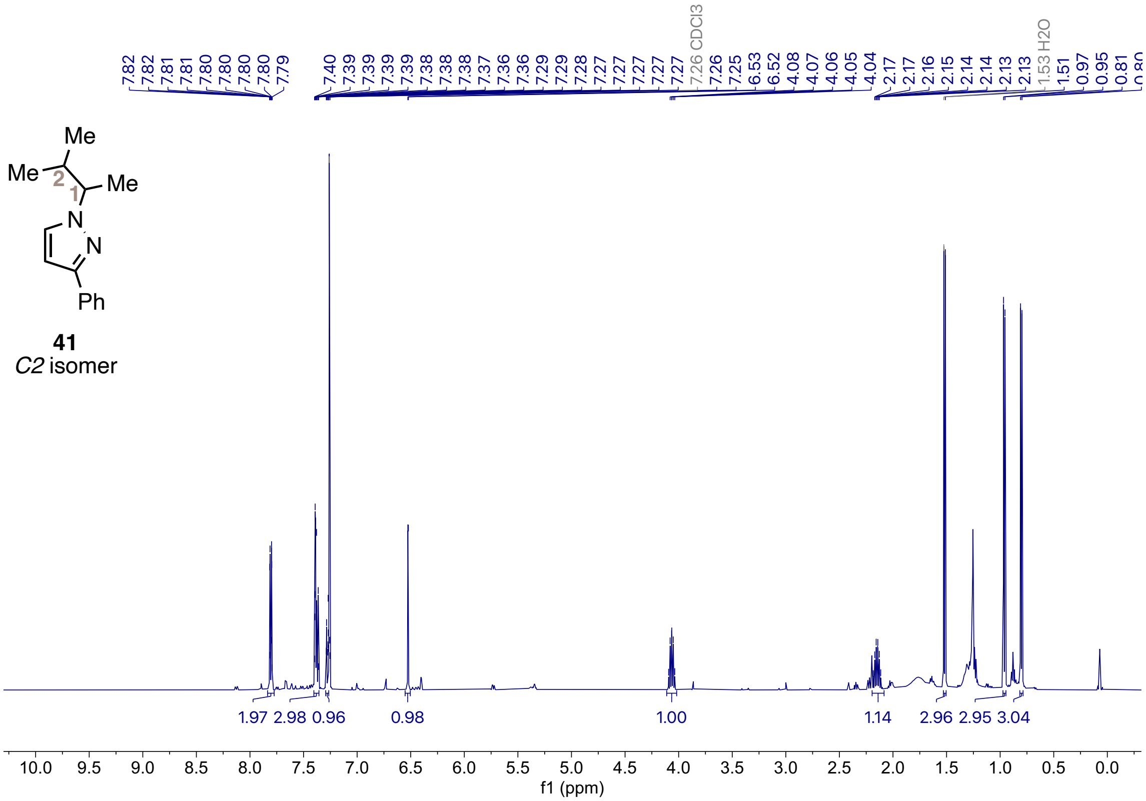

^{1}H NMR spectrum of **41** (*C2* isomer) taken in $CDCl_3$.

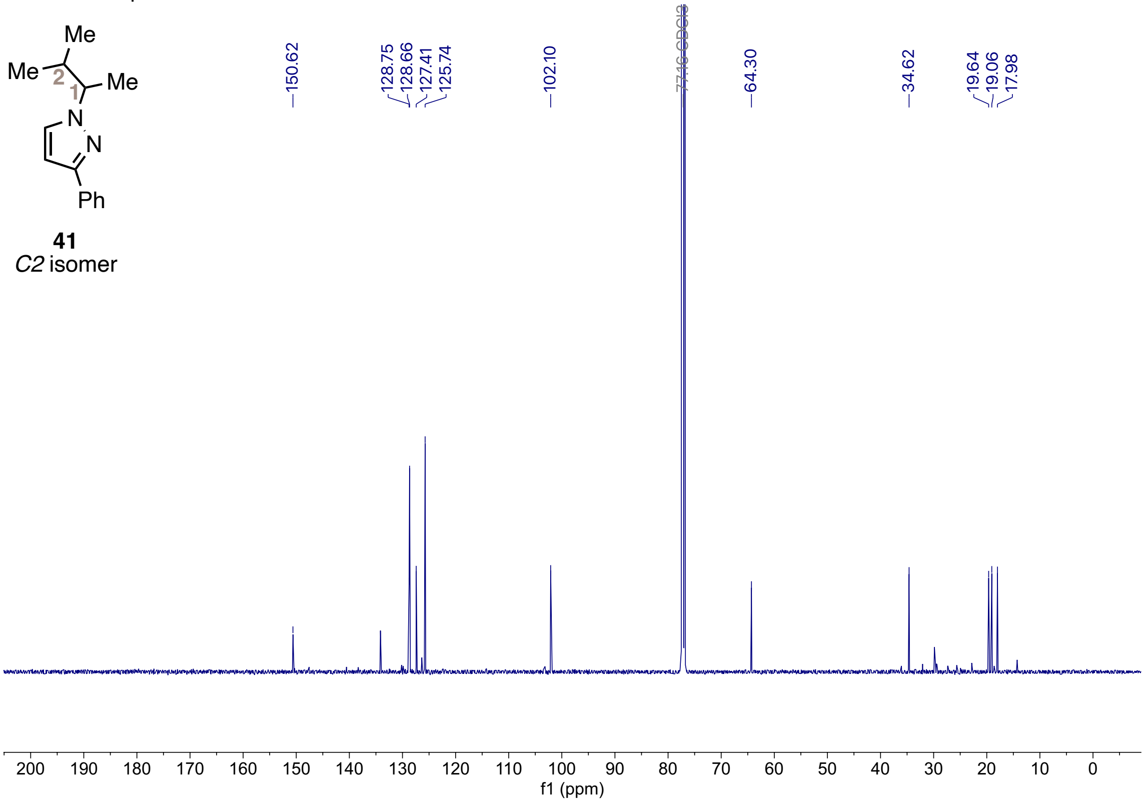

^{13}C NMR spectrum of **41** (*C2* isomer) taken in $CDCl_3$.

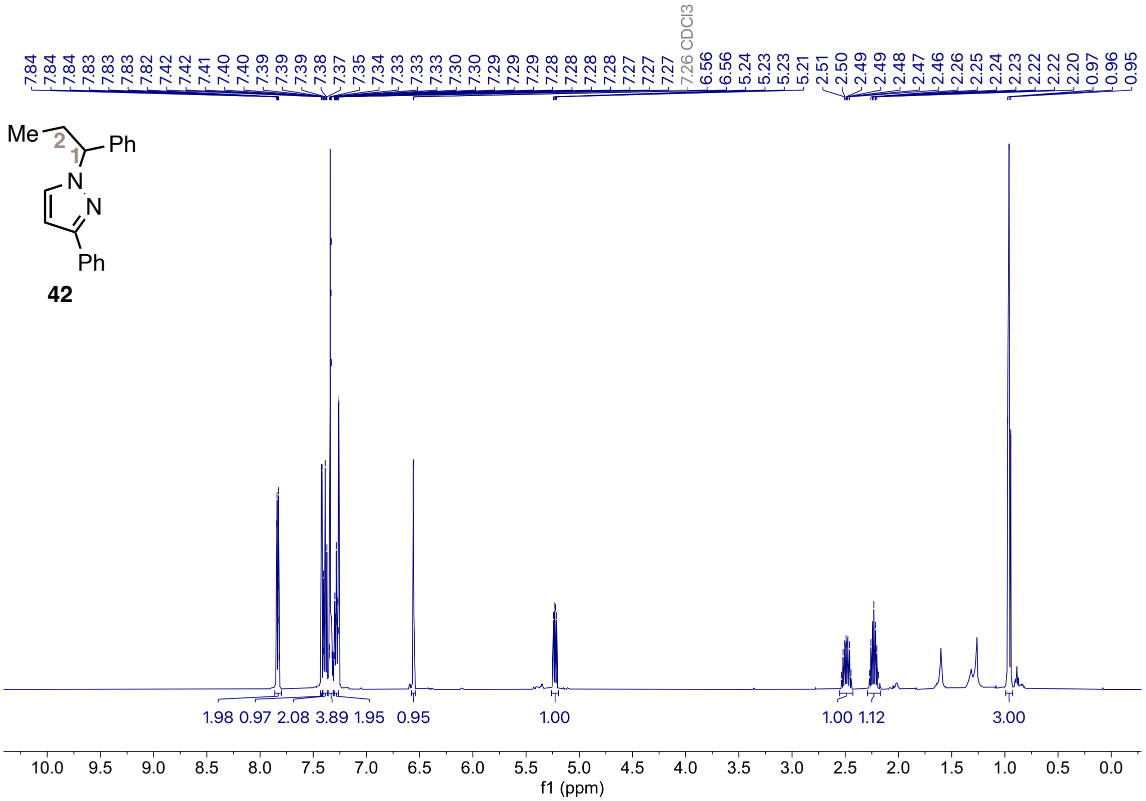

^{1}H NMR spectrum of **42** taken in $CDCl_3$.

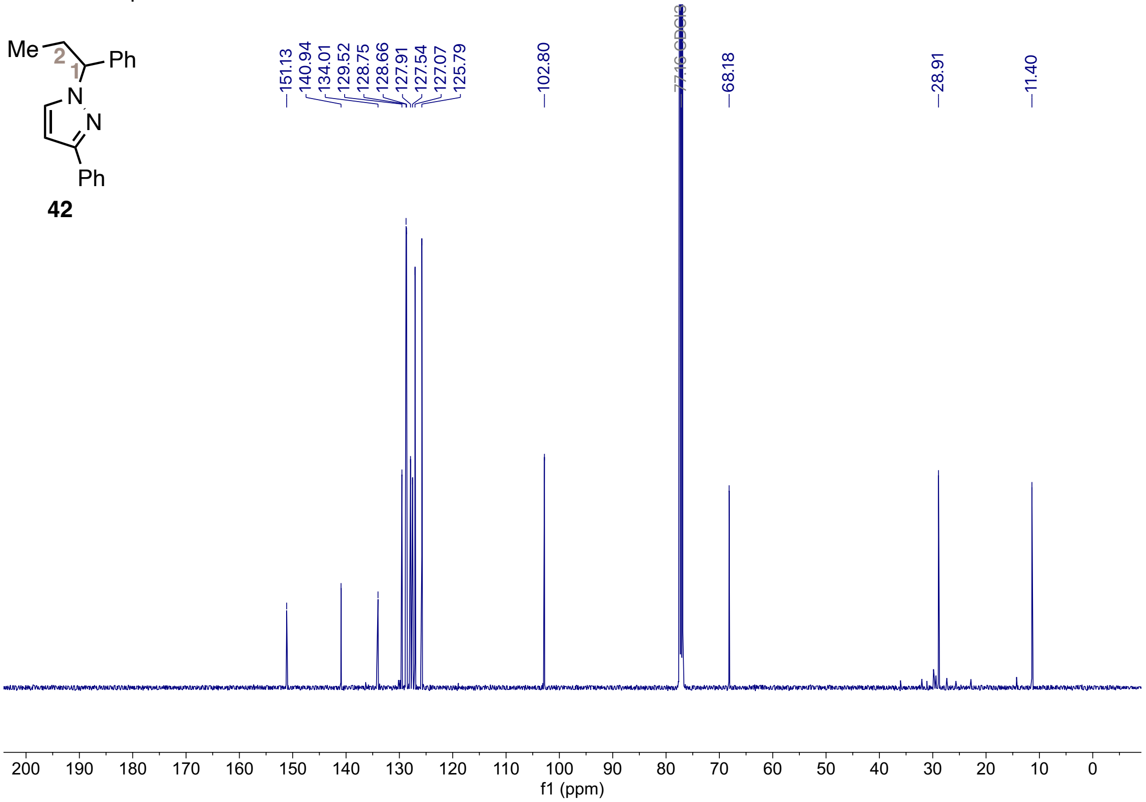

^{13}C NMR spectrum of **42** taken in $CDCl_3$.

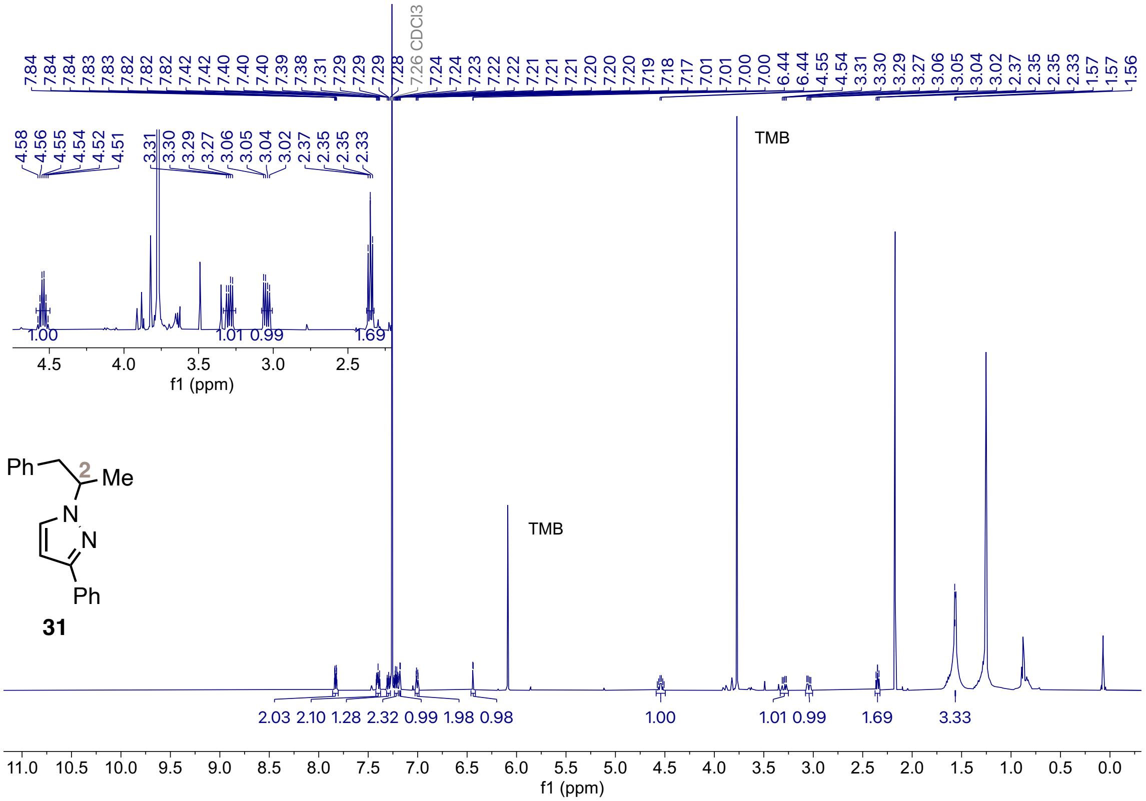

^{1}H NMR spectrum of **31** taken in $CDCl_3$. Co-eluted with 1,3,5-TMB.

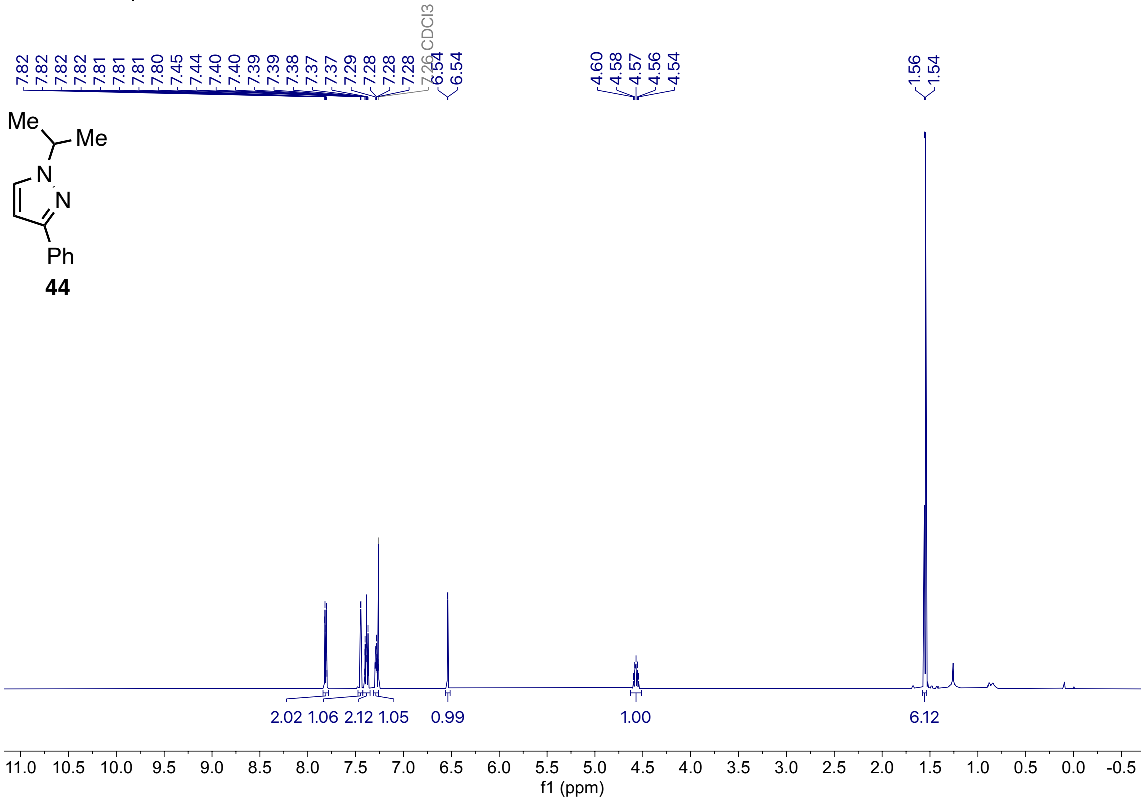

^{1}H NMR spectrum of **44** taken in $CDCl_3$.

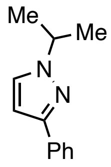

44

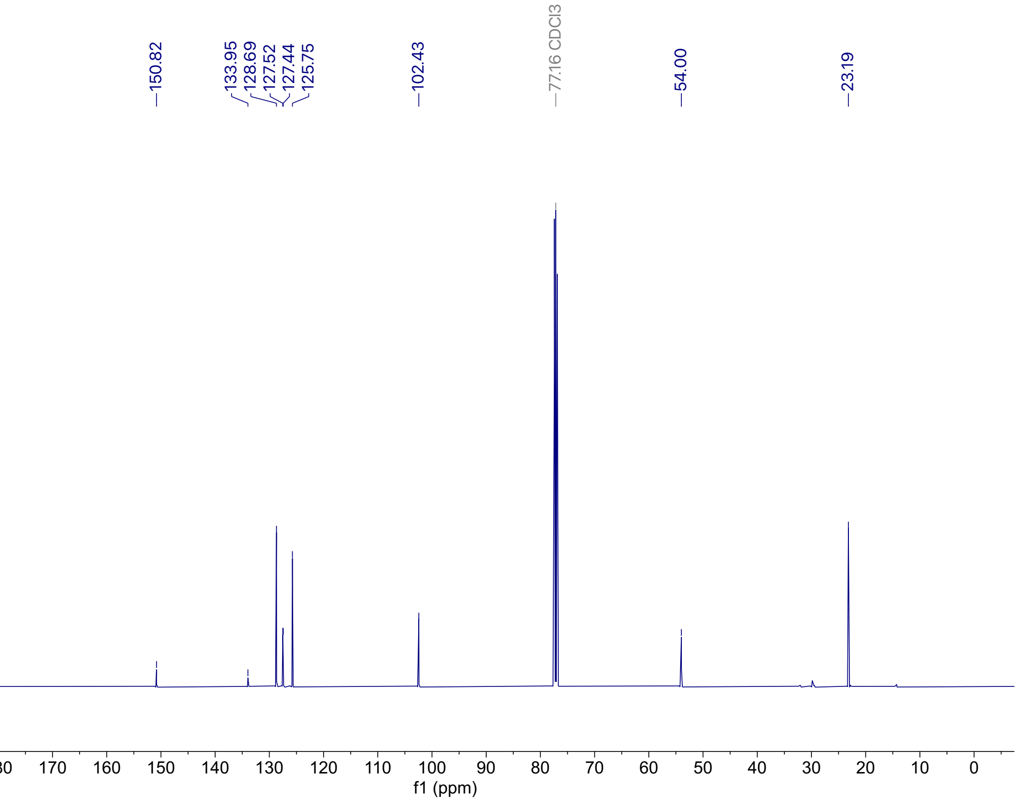

^{13}C NMR spectrum of **44** taken in $CDCl_3$.
